# Supplementary material for: Retinoic acid-induced protein 14 links mechanical forces to Hippo signaling
Source: EMBO Rep. 2024 Aug 19;25(9):18. doi: 10.1038/s44319-024-00228-0 (PMC11387738; doi:10.1038/s44319-024-00228-0)
Supplement: Supplementary file 1 — Appendix [file 44319_2024_228_MOESM1_ESM.pdf]

Appendix

Retinoic acid-induced protein 14 links mechanical forces to Hippo signaling

(Wonyoung Jeong *et al.*)

Contents list

Appendix Figure S1 -- 2

Appendix Figure S2 -- 3

Appendix Figure S3 -- 4

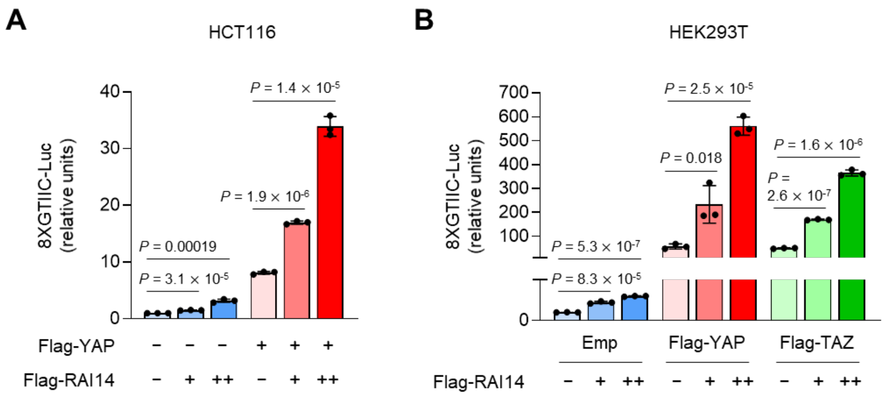

Appendix Figure S1. RAI14 activates YAP/TAZ activity

(A, B) Overexpression of RAI14 activated YAP-reporter activity. HCT116 cells (A) and HEK293T cells (B) were transfected as indicated in the figures.

Data information: In Figures A and B, the error bars indicate ± s.d. of triplicate measurements (biological replicate). Statistical analysis was performed using two-tailed unpaired t-test, and exact *P* values are shown in each figure; $P < 0.05$, statistically significant. Black dots on the graphs indicate individual measurements.

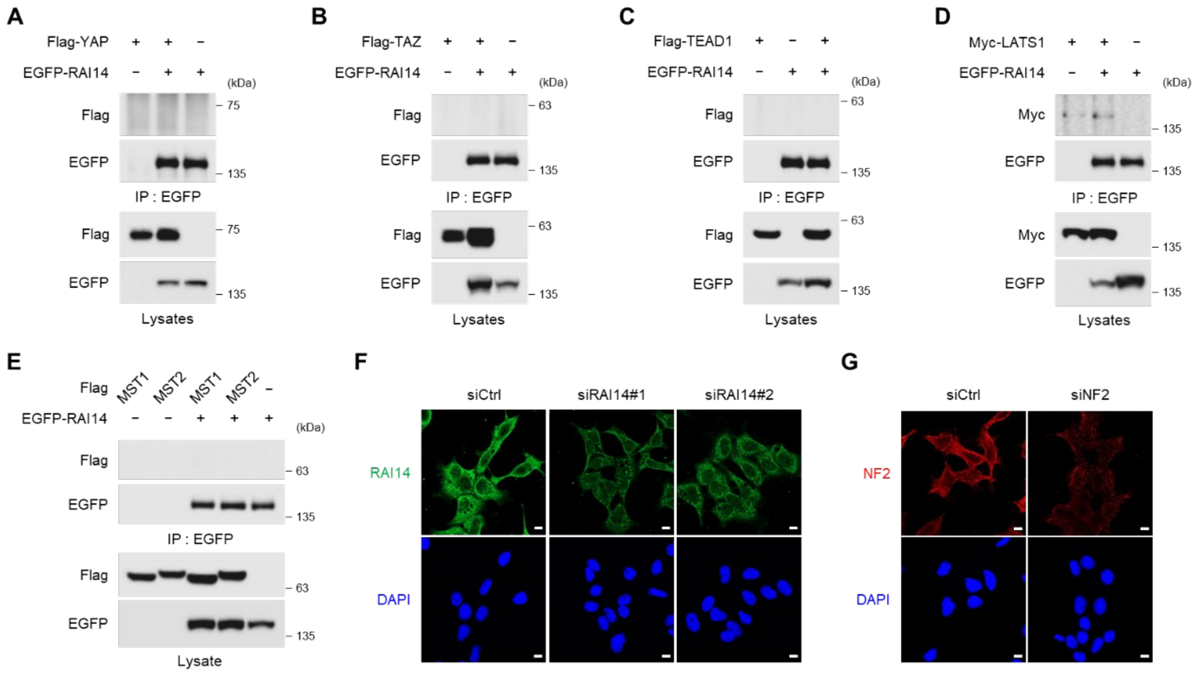

Appendix Figure S2. Interaction with RAI14 and other Hippo components and validation of RAI14 and NF2 antibodies

(A-E) Overexpressed Flag-YAP, Flag-TAZ, Flag-TEAD1, myc-LATS1, and Flag-MST1/2 did not interact with EGFP-RAI14. HEK293T cells were transfected as indicated in the figures.

(F, G) Validation of RAI14 and NF2 antibodies for immunofluorescence assay. HEK293A cells were used. Scale bars: 10 μm.

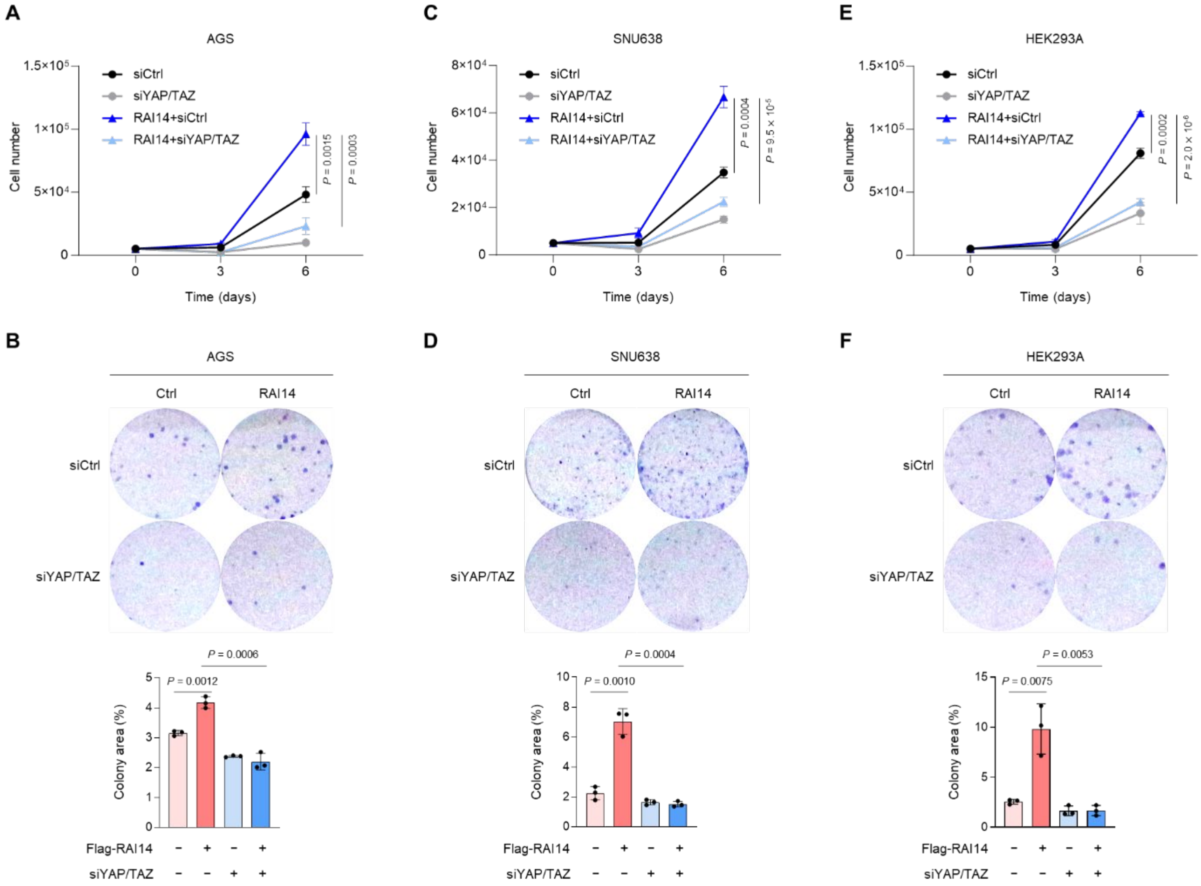

Appendix Figure S3. Effect of RAI14 overexpression and YAP/TAZ knockdown in cell proliferation and growth

(A, C, and E) Overexpressed RAI14 increased cell proliferation ability, but the knockdown of YAP/TAZ attenuated the effect of RAI14 overexpression. AGS (A), SNU638 (C), and HEK293A (E) cells were transfected as indicated in the figure.

(B, D, and F) Overexpressed RAI14 increased colony-forming ability, but the knockdown of YAP/TAZ attenuated the effect of RAI14 overexpression. AGS (B), SNU638 (D), and HEK293A (F) cells were transfected as indicated in the figure. The quantification of "colony area per area of the well" is shown.

Data information: In all Figures, the error bars indicate ± s.d. of triplicate measurements (biological replicate). Statistical analysis was performed using two-tailed unpaired t-test, and exact P values are shown in each figure; $P < 0.05$, statistically significant. Black dots on the graphs indicate individual measurements.
