# Supplementary figures and images for: Retinoic acid-induced protein 14 links mechanical forces to Hippo signaling (part 1 of 3)
Source: EMBO Rep. 2024 Aug 19;25(9):18. doi: 10.1038/s44319-024-00228-0 (PMC11387738; doi:10.1038/s44319-024-00228-0)

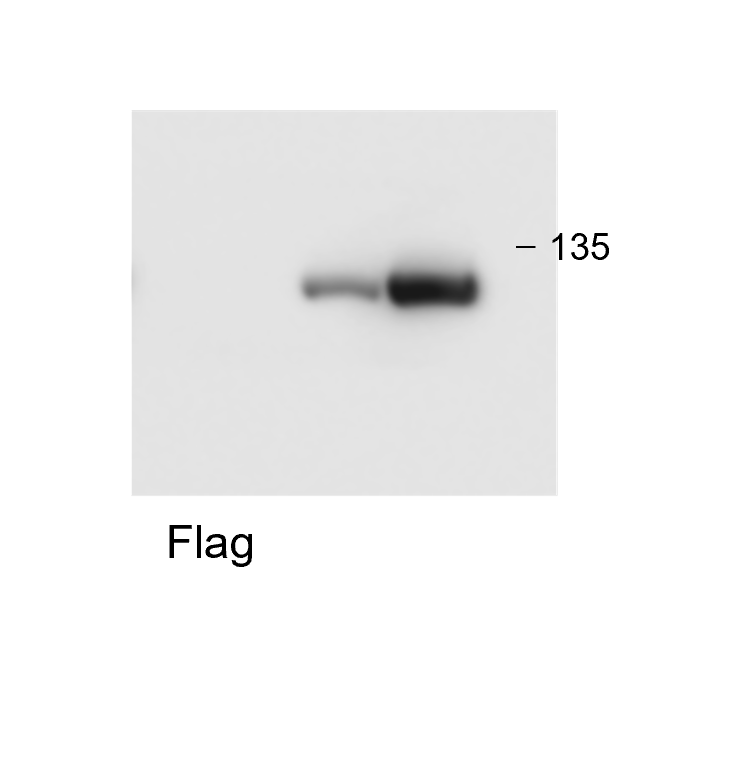

Supplement: Supplementary file 3 — Source data Fig. 1 [file 44319_2024_228_MOESM3_ESM.zip › Figure 1/Figure 1H/Flag.tif]

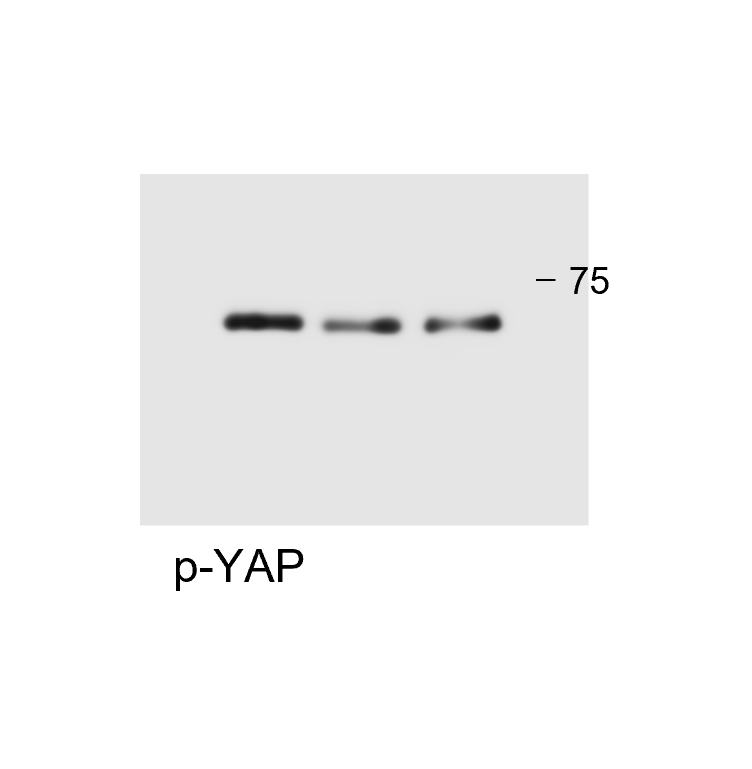

Supplement: Supplementary file 3 — Source data Fig. 1 [file 44319_2024_228_MOESM3_ESM.zip › Figure 1/Figure 1H/p-YAP.tif]

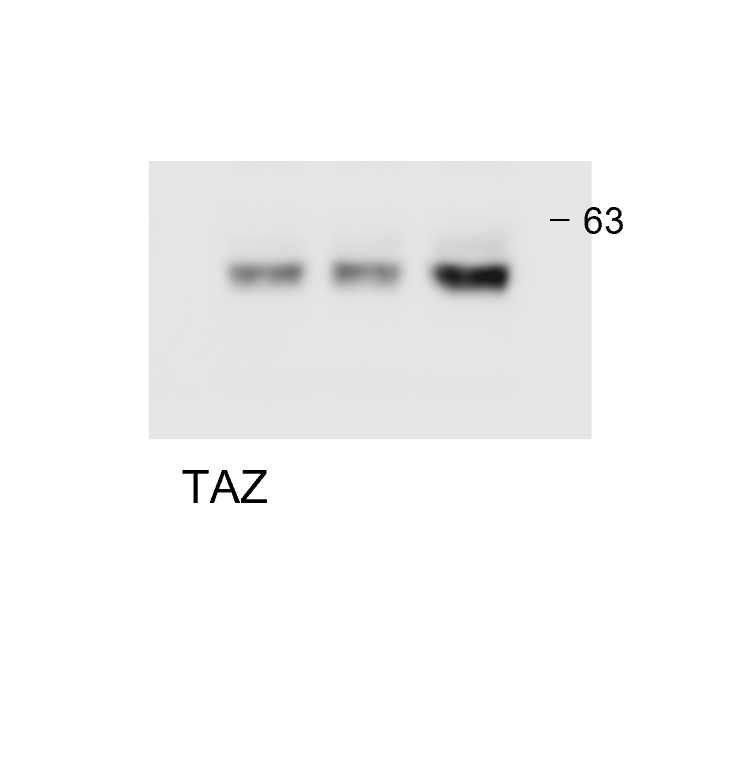

Supplement: Supplementary file 3 — Source data Fig. 1 [file 44319_2024_228_MOESM3_ESM.zip › Figure 1/Figure 1H/TAZ.tif]

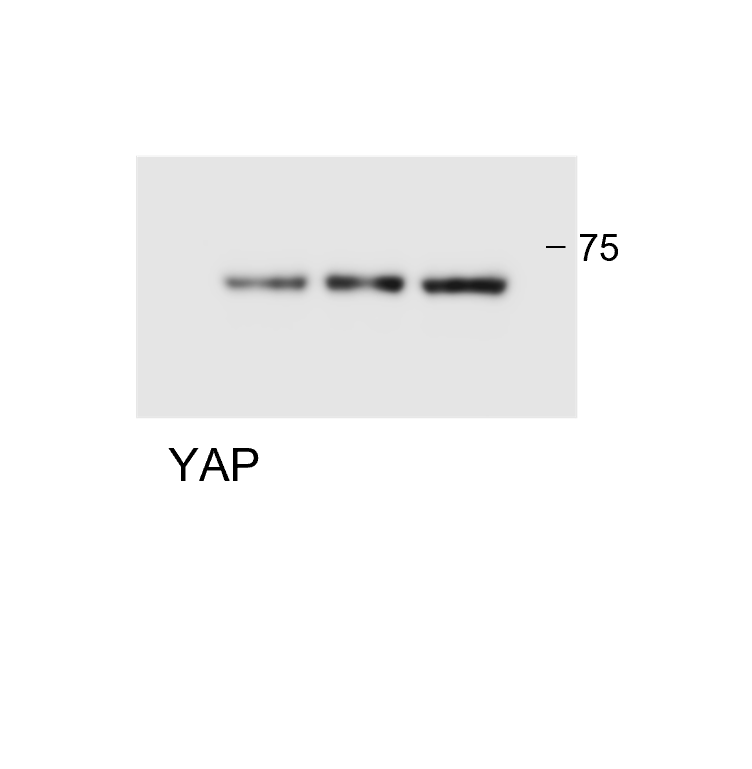

Supplement: Supplementary file 3 — Source data Fig. 1 [file 44319_2024_228_MOESM3_ESM.zip › Figure 1/Figure 1H/YAP.tif]

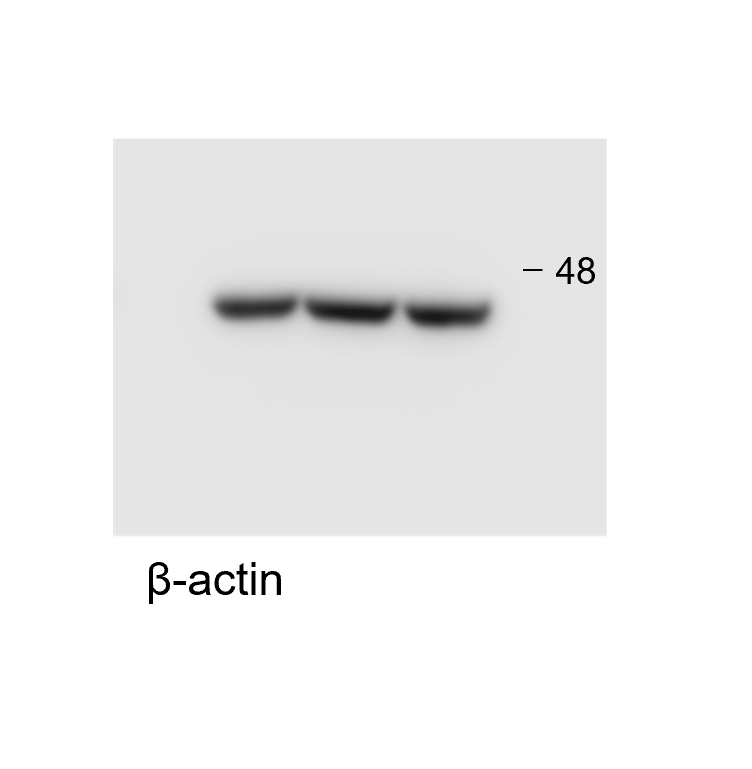

Supplement: Supplementary file 3 — Source data Fig. 1 [file 44319_2024_228_MOESM3_ESM.zip › Figure 1/Figure 1H/ÑΓ-actin.tif]

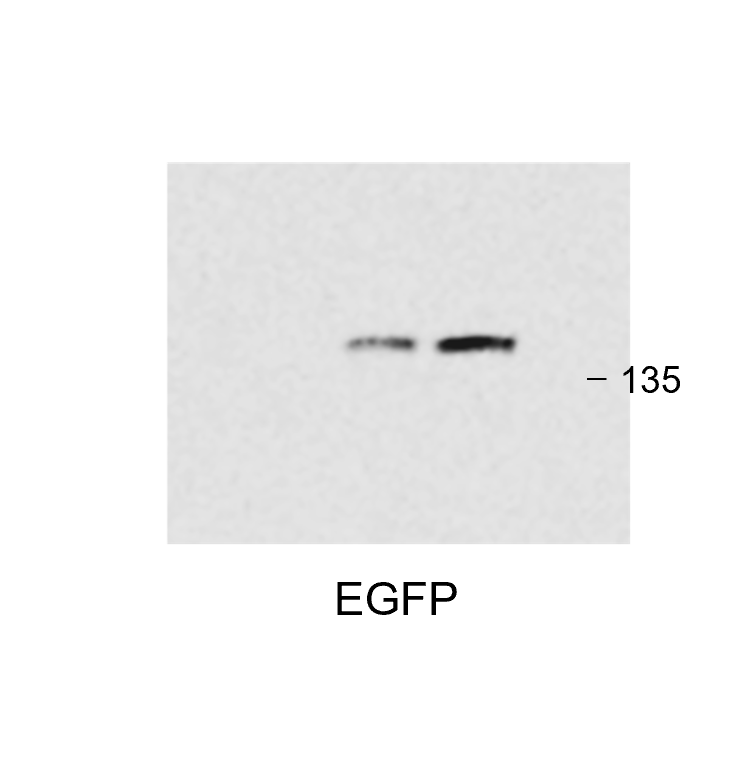

Supplement: Supplementary file 3 — Source data Fig. 1 [file 44319_2024_228_MOESM3_ESM.zip › Figure 1/Figure 1I/EGFP.tif]

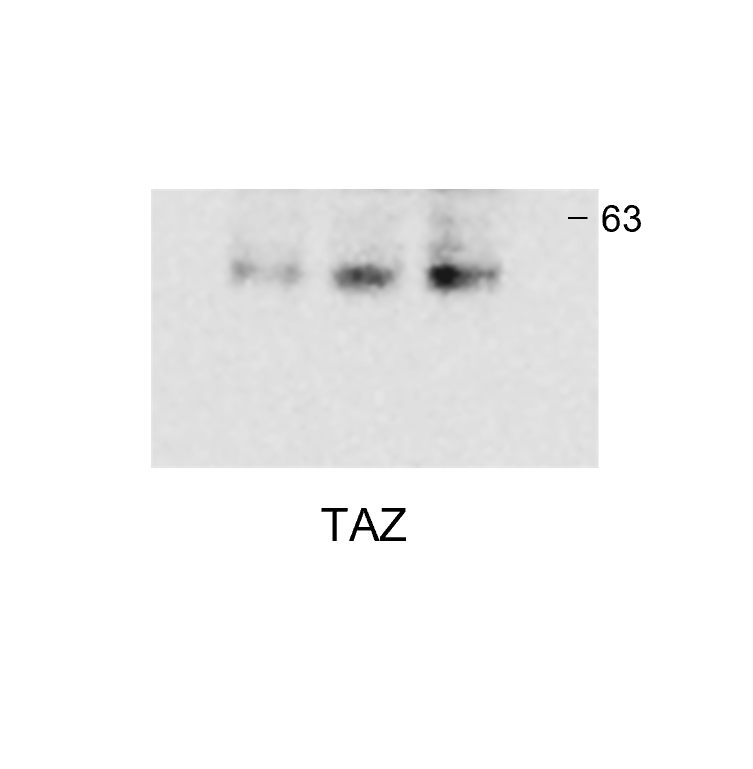

Supplement: Supplementary file 3 — Source data Fig. 1 [file 44319_2024_228_MOESM3_ESM.zip › Figure 1/Figure 1I/TAZ.tif]

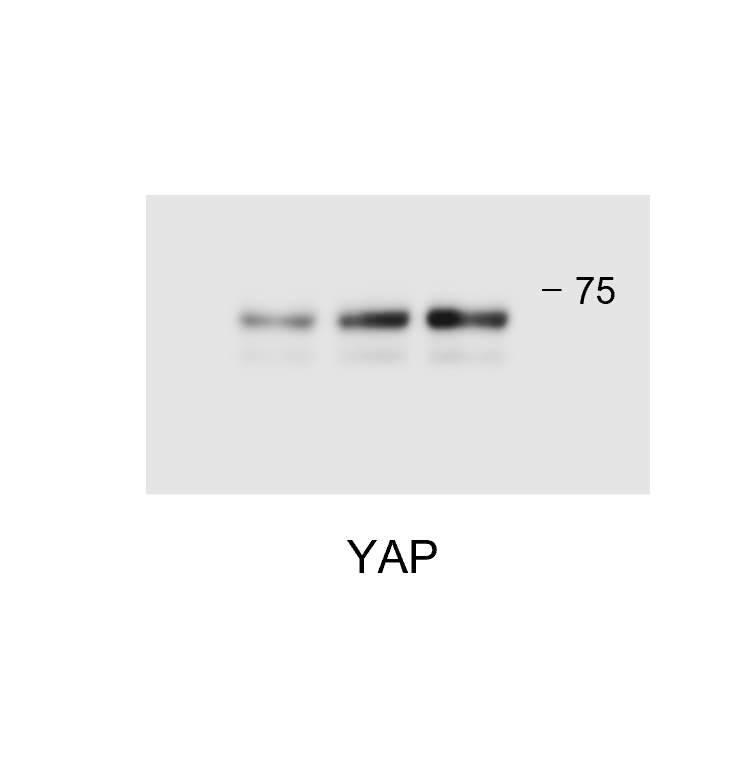

Supplement: Supplementary file 3 — Source data Fig. 1 [file 44319_2024_228_MOESM3_ESM.zip › Figure 1/Figure 1I/YAP.tif]

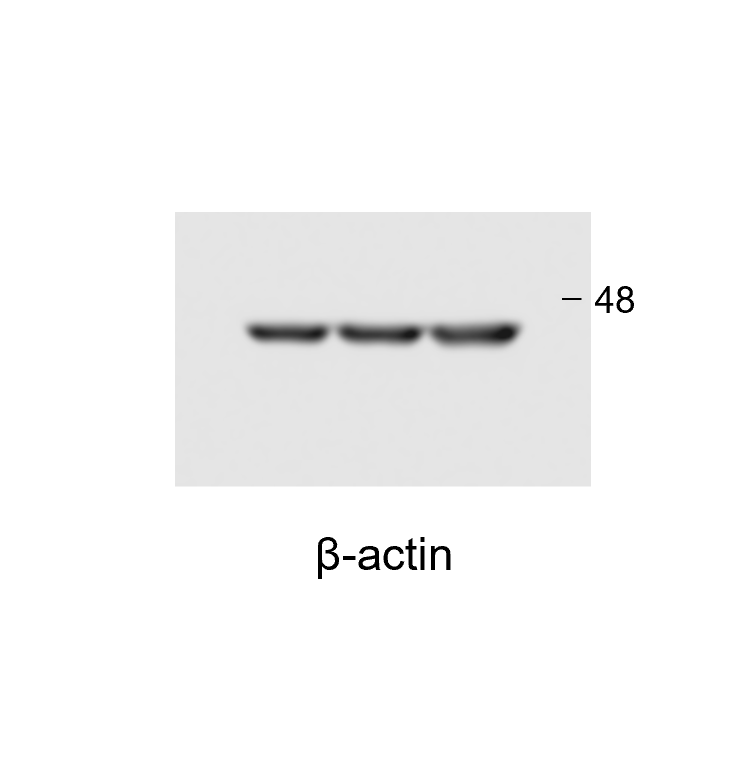

Supplement: Supplementary file 3 — Source data Fig. 1 [file 44319_2024_228_MOESM3_ESM.zip › Figure 1/Figure 1I/ÑΓ-actin.tif]

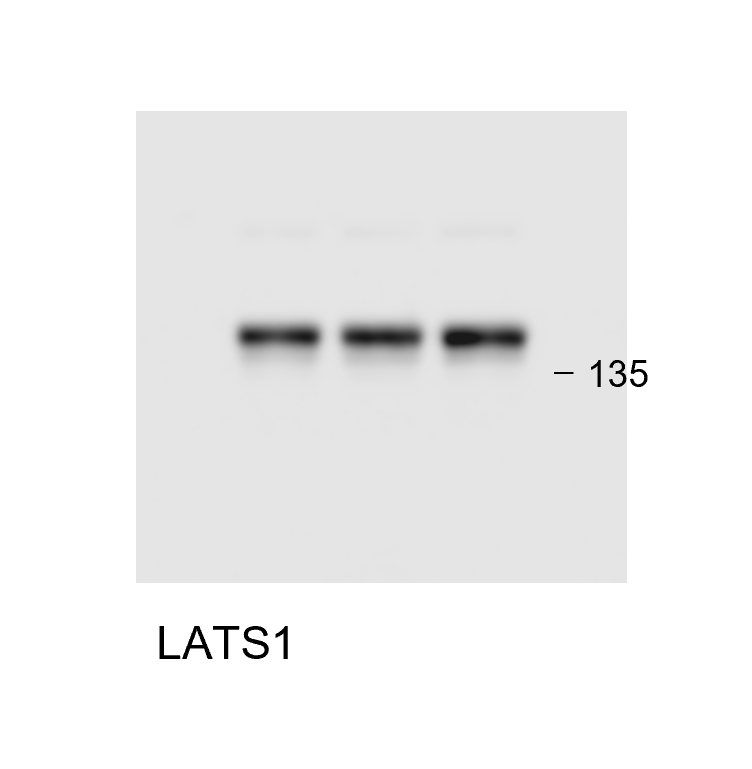

Supplement: Supplementary file 3 — Source data Fig. 1 [file 44319_2024_228_MOESM3_ESM.zip › Figure 1/Figure 1J/LATS1.tif]

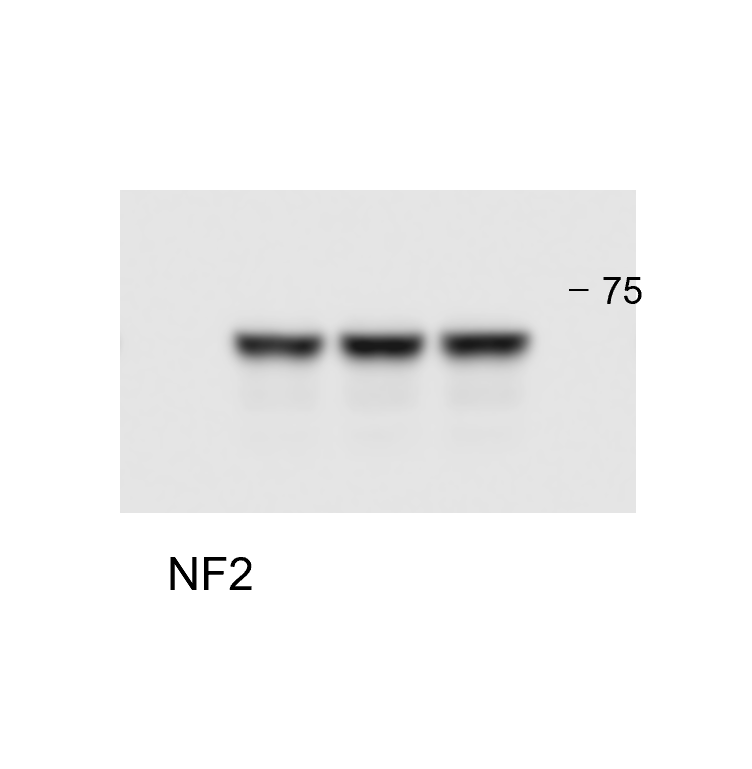

Supplement: Supplementary file 3 — Source data Fig. 1 [file 44319_2024_228_MOESM3_ESM.zip › Figure 1/Figure 1J/NF2.tif]

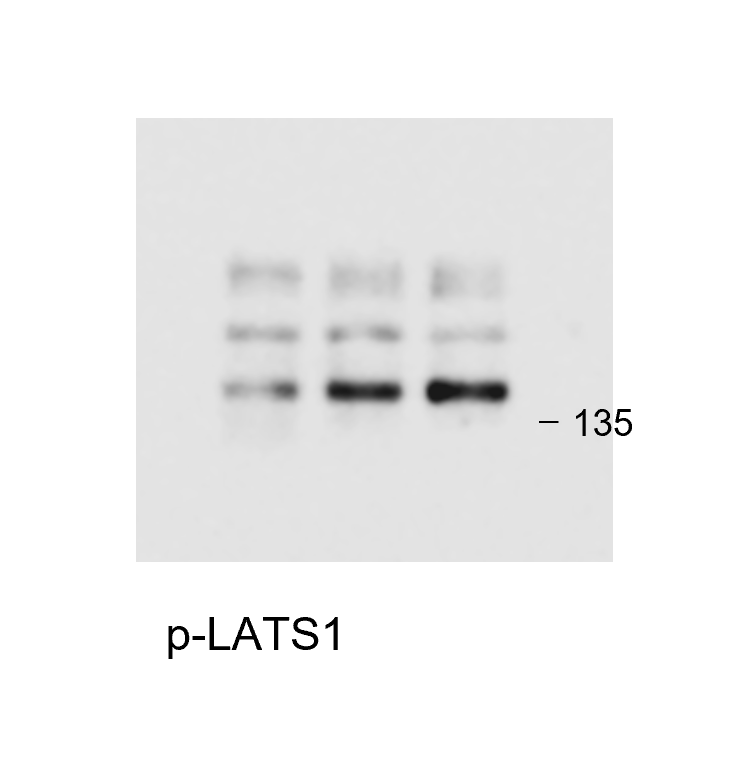

Supplement: Supplementary file 3 — Source data Fig. 1 [file 44319_2024_228_MOESM3_ESM.zip › Figure 1/Figure 1J/p-LATS1.tif]

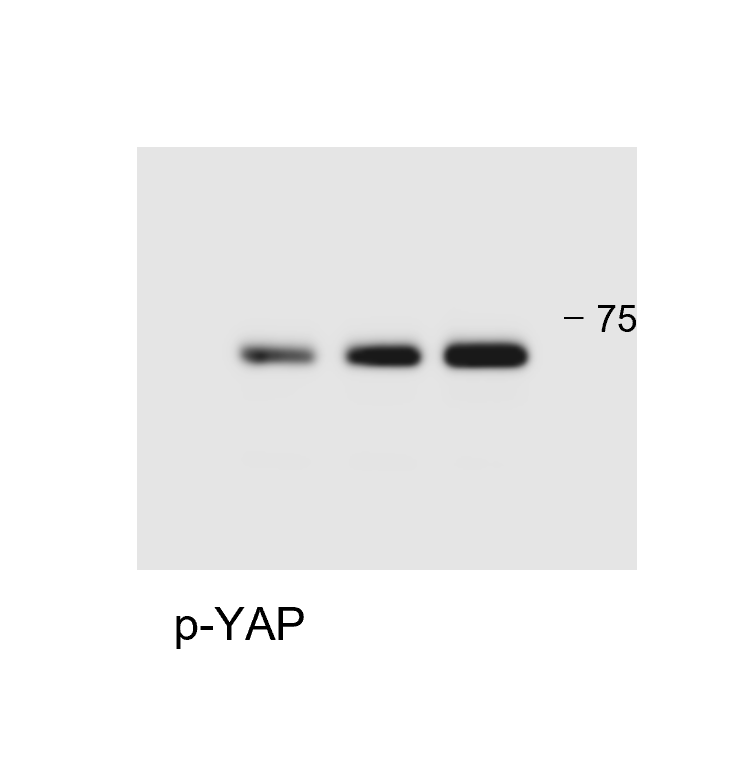

Supplement: Supplementary file 3 — Source data Fig. 1 [file 44319_2024_228_MOESM3_ESM.zip › Figure 1/Figure 1J/p-YAP.tif]

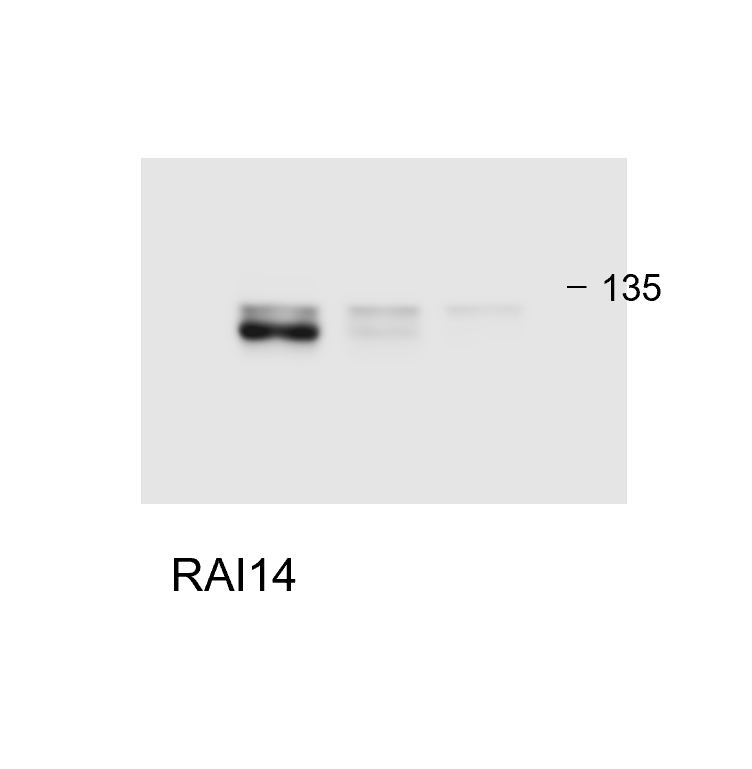

Supplement: Supplementary file 3 — Source data Fig. 1 [file 44319_2024_228_MOESM3_ESM.zip › Figure 1/Figure 1J/RAI14.tif]

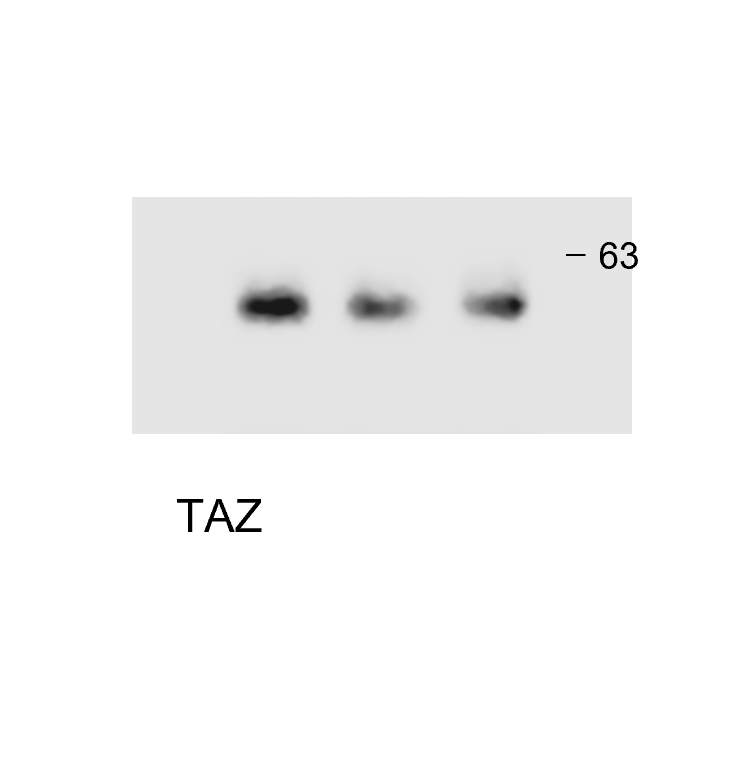

Supplement: Supplementary file 3 — Source data Fig. 1 [file 44319_2024_228_MOESM3_ESM.zip › Figure 1/Figure 1J/TAZ.tif]

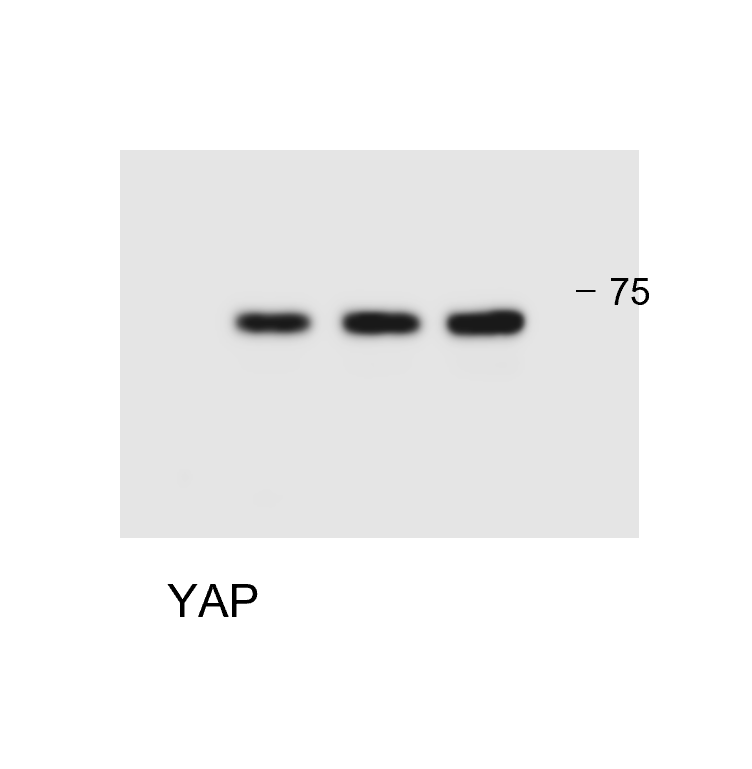

Supplement: Supplementary file 3 — Source data Fig. 1 [file 44319_2024_228_MOESM3_ESM.zip › Figure 1/Figure 1J/YAP.tif]

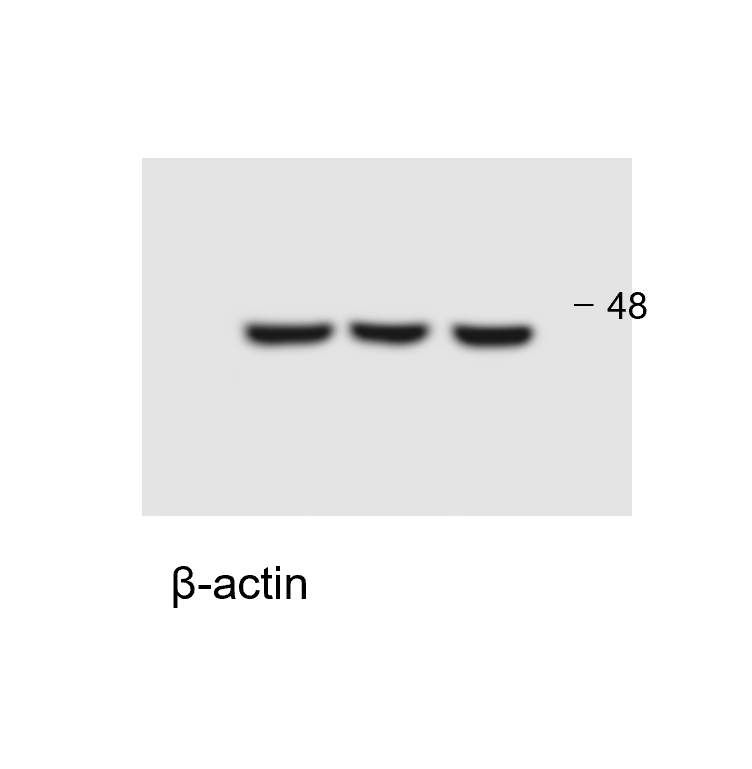

Supplement: Supplementary file 3 — Source data Fig. 1 [file 44319_2024_228_MOESM3_ESM.zip › Figure 1/Figure 1J/ÑΓ-actin.tif]

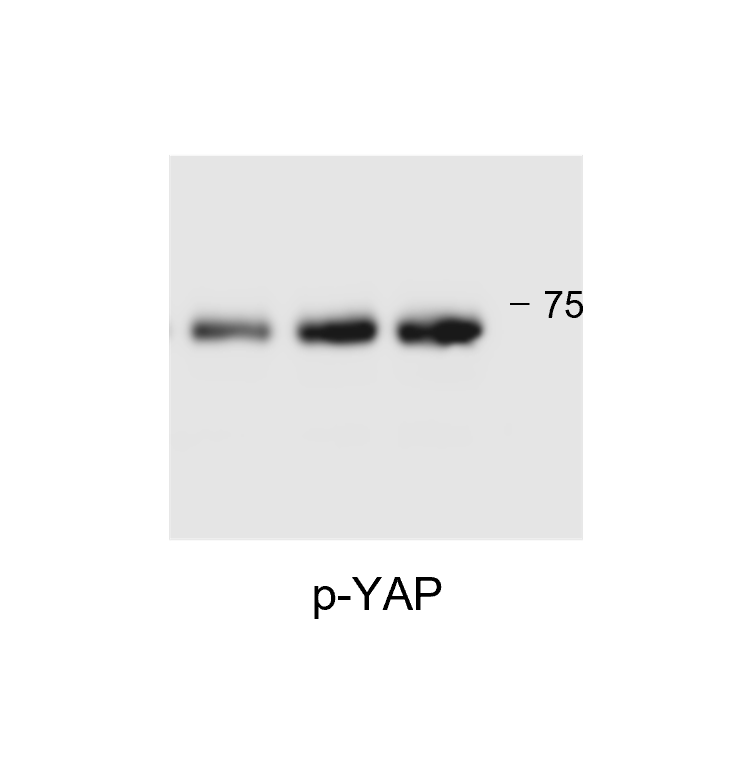

Supplement: Supplementary file 3 — Source data Fig. 1 [file 44319_2024_228_MOESM3_ESM.zip › Figure 1/Figure 1K/p-YAP.tif]

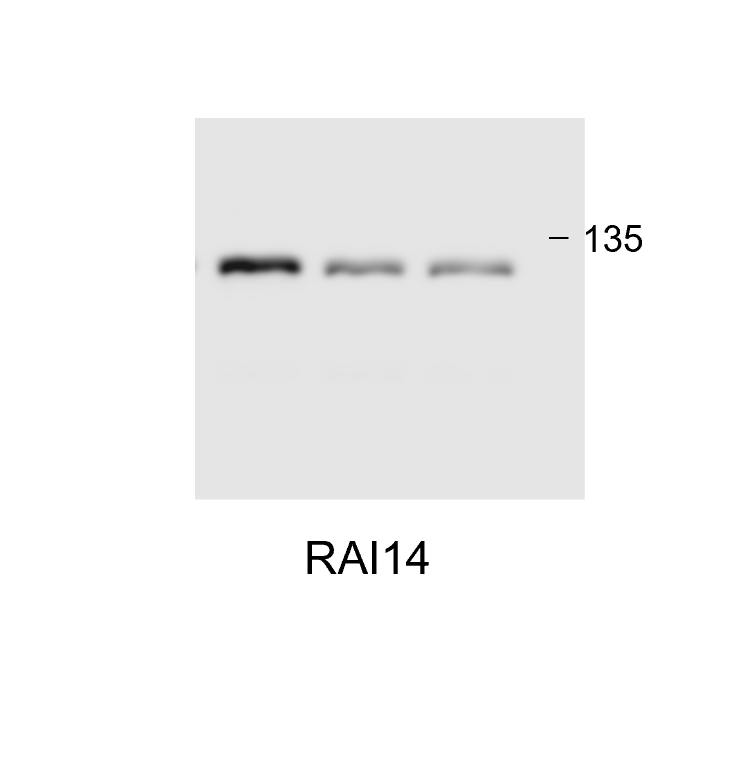

Supplement: Supplementary file 3 — Source data Fig. 1 [file 44319_2024_228_MOESM3_ESM.zip › Figure 1/Figure 1K/RAI14.tif]

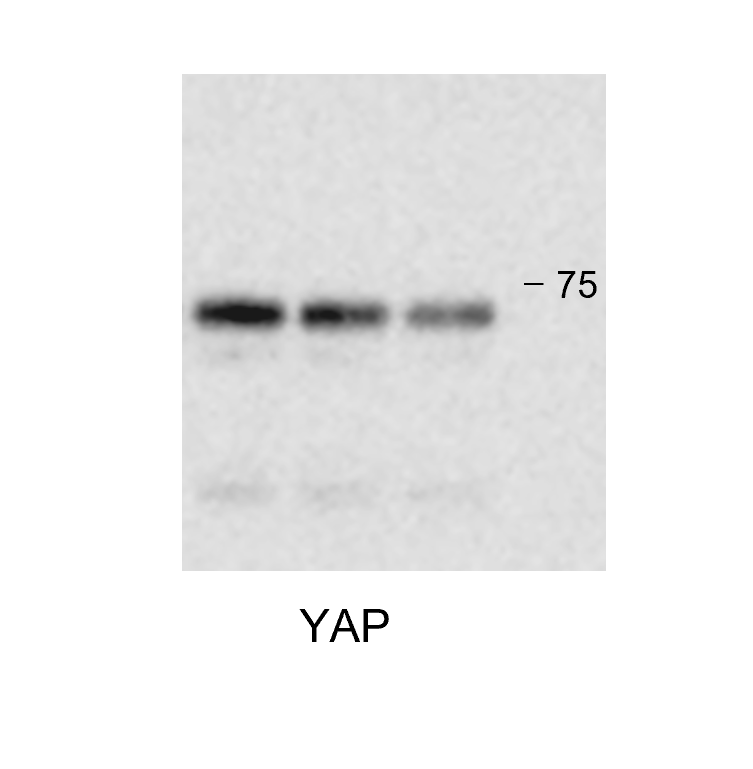

Supplement: Supplementary file 3 — Source data Fig. 1 [file 44319_2024_228_MOESM3_ESM.zip › Figure 1/Figure 1K/YAP.tif]

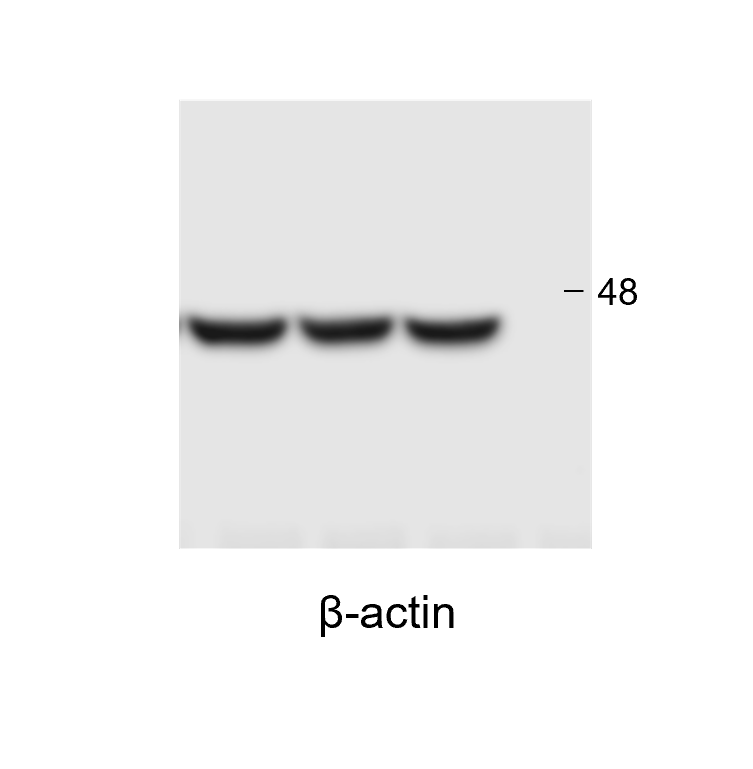

Supplement: Supplementary file 3 — Source data Fig. 1 [file 44319_2024_228_MOESM3_ESM.zip › Figure 1/Figure 1K/ÑΓ-actin.tif]

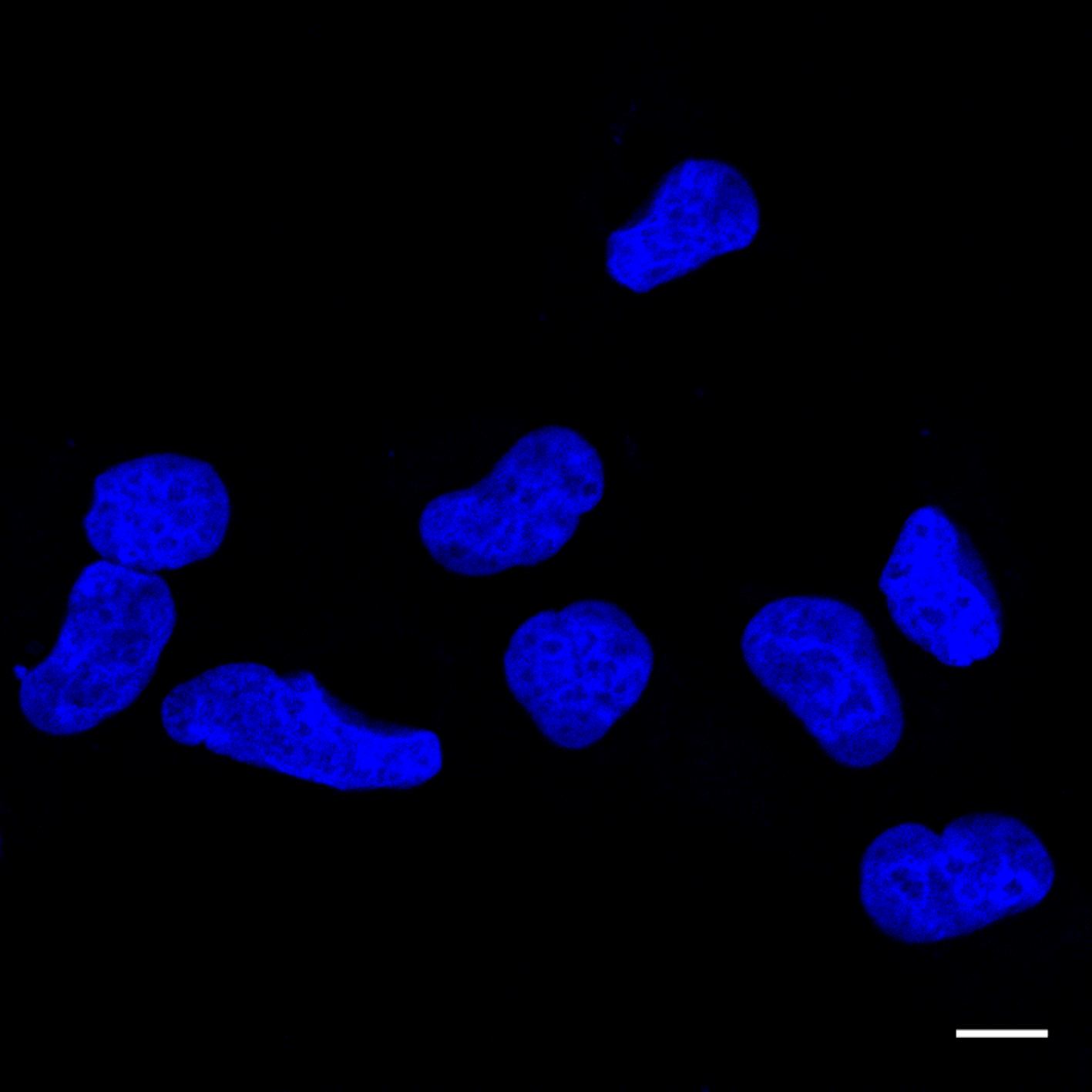

Supplement: Supplementary file 3 — Source data Fig. 1 [file 44319_2024_228_MOESM3_ESM.zip › Figure 1/Figure 1L/DAPI_siCtrl.tif]

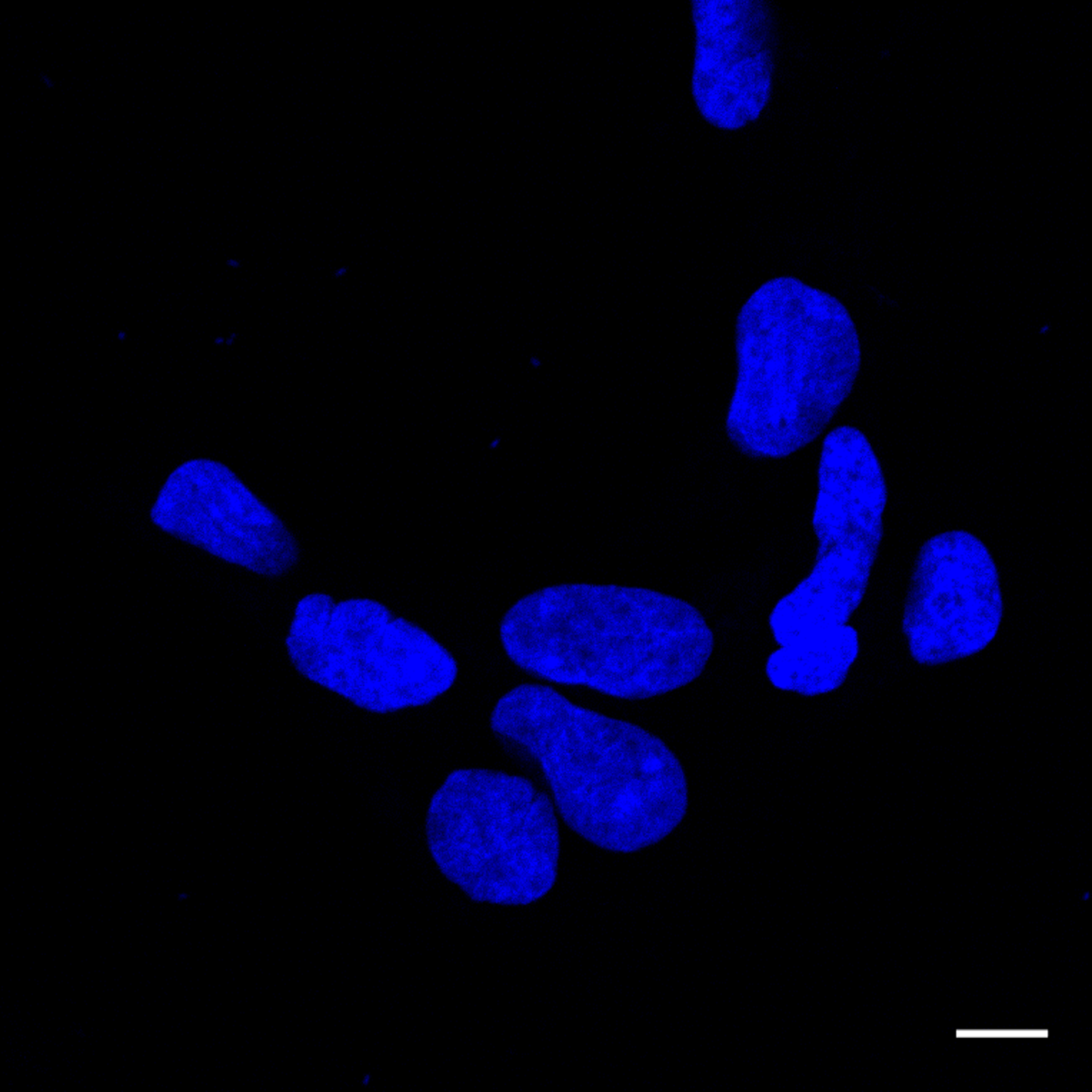

Supplement: Supplementary file 3 — Source data Fig. 1 [file 44319_2024_228_MOESM3_ESM.zip › Figure 1/Figure 1L/DAPI_siRAI14#1.tif]

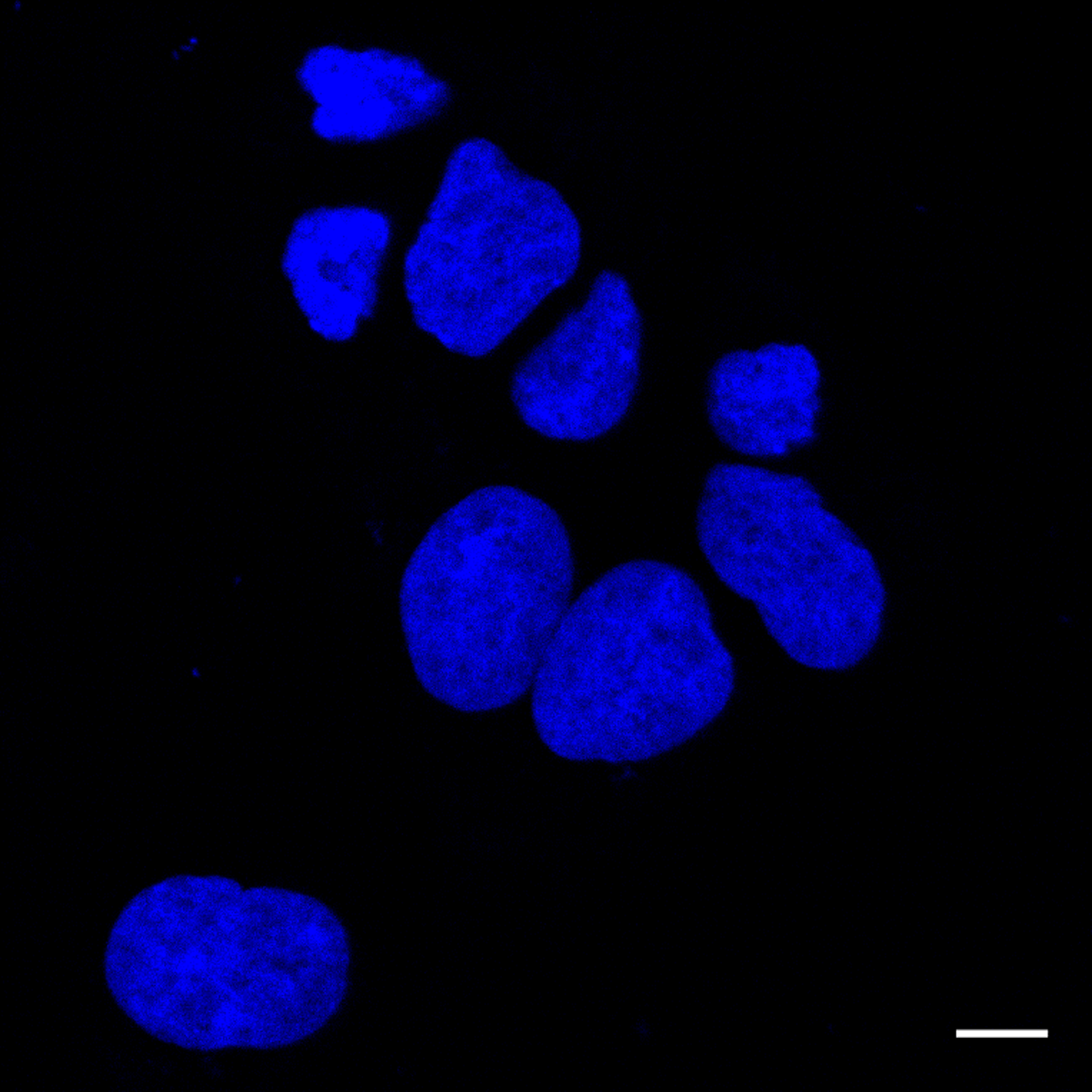

Supplement: Supplementary file 3 — Source data Fig. 1 [file 44319_2024_228_MOESM3_ESM.zip › Figure 1/Figure 1L/DAPI_siRAI14#2.tif]

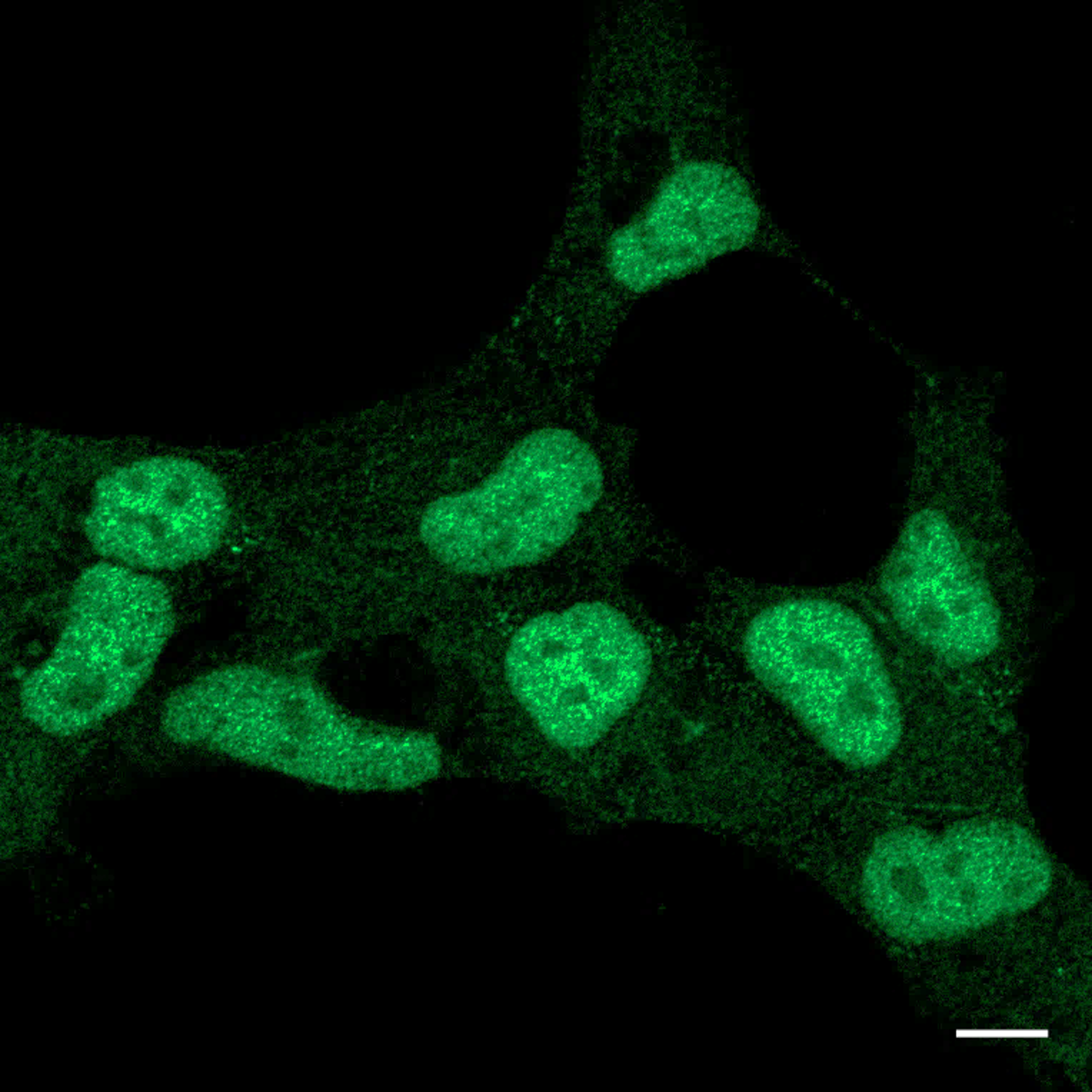

Supplement: Supplementary file 3 — Source data Fig. 1 [file 44319_2024_228_MOESM3_ESM.zip › Figure 1/Figure 1L/YAP_siCtrl.tif]

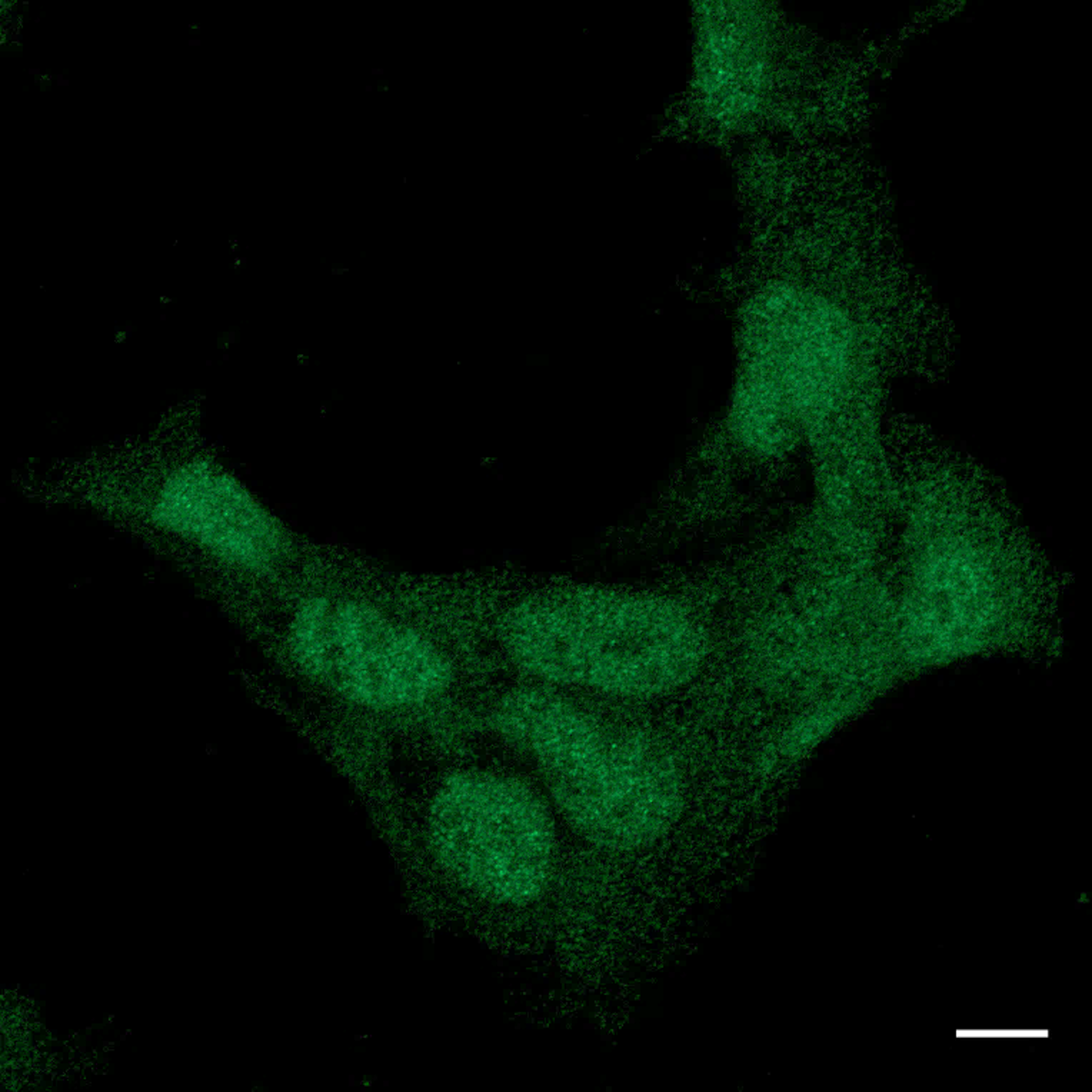

Supplement: Supplementary file 3 — Source data Fig. 1 [file 44319_2024_228_MOESM3_ESM.zip › Figure 1/Figure 1L/YAP_siRAI14#1.tif]

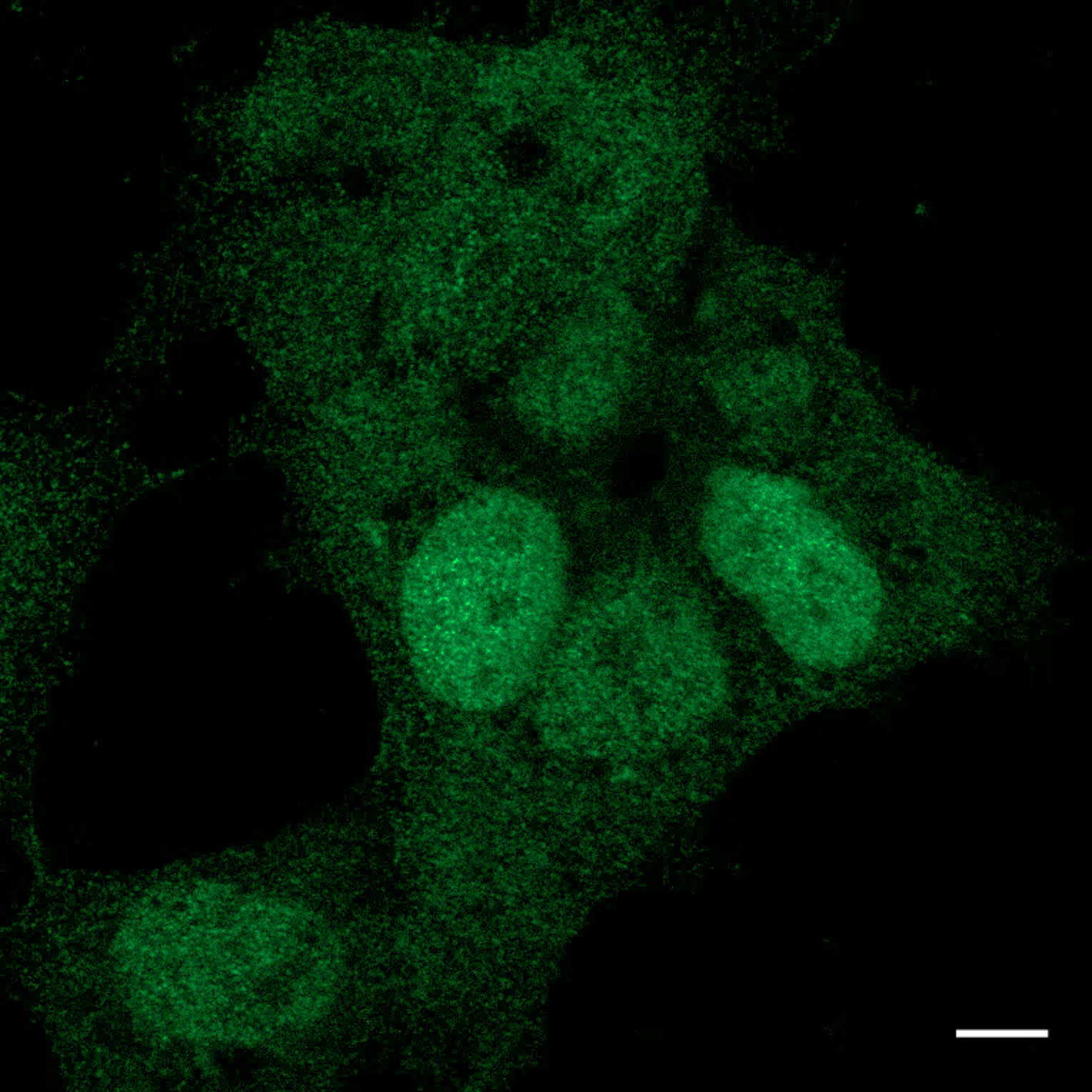

Supplement: Supplementary file 3 — Source data Fig. 1 [file 44319_2024_228_MOESM3_ESM.zip › Figure 1/Figure 1L/YAP_siRAI14#2.tif]

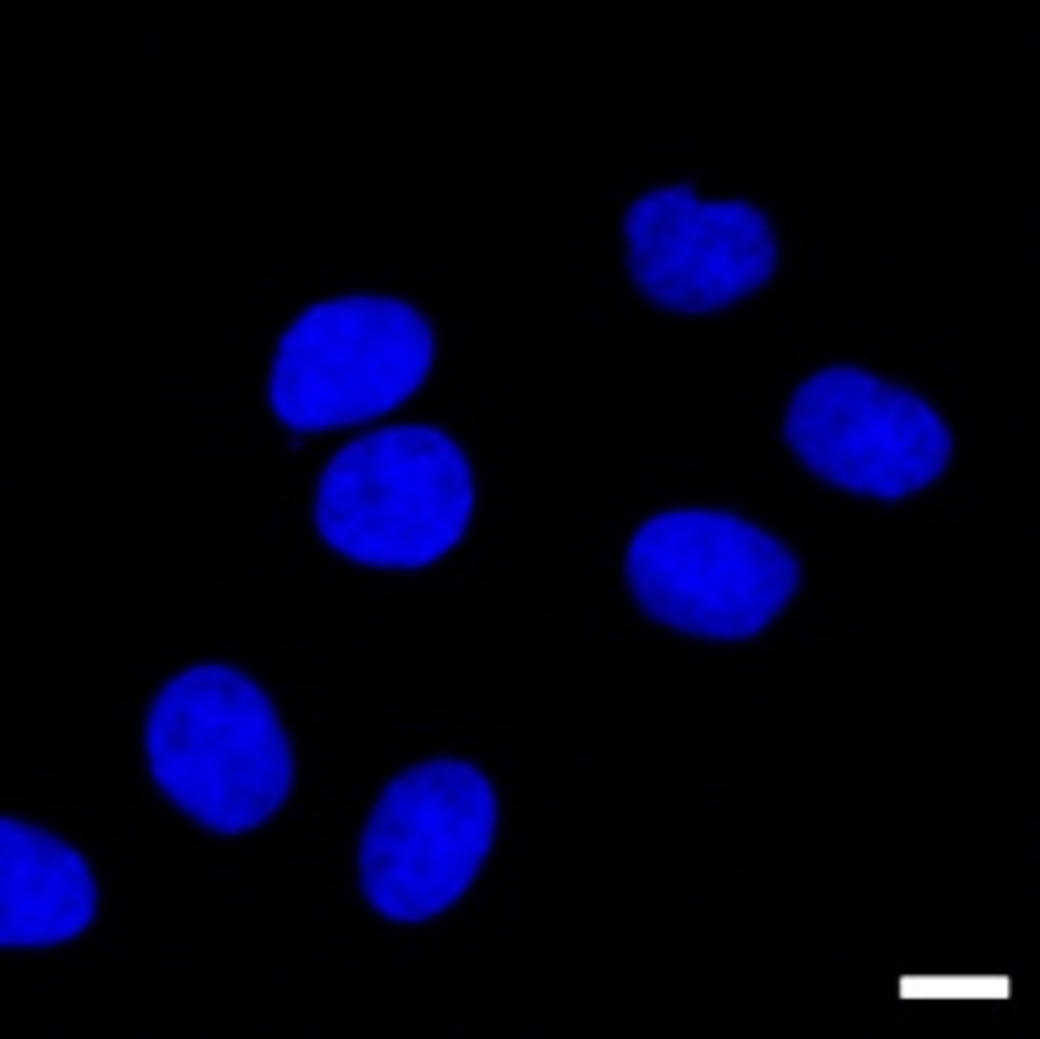

Supplement: Supplementary file 3 — Source data Fig. 1 [file 44319_2024_228_MOESM3_ESM.zip › Figure 1/Figure 1M/DAPI_siCtrl.png]

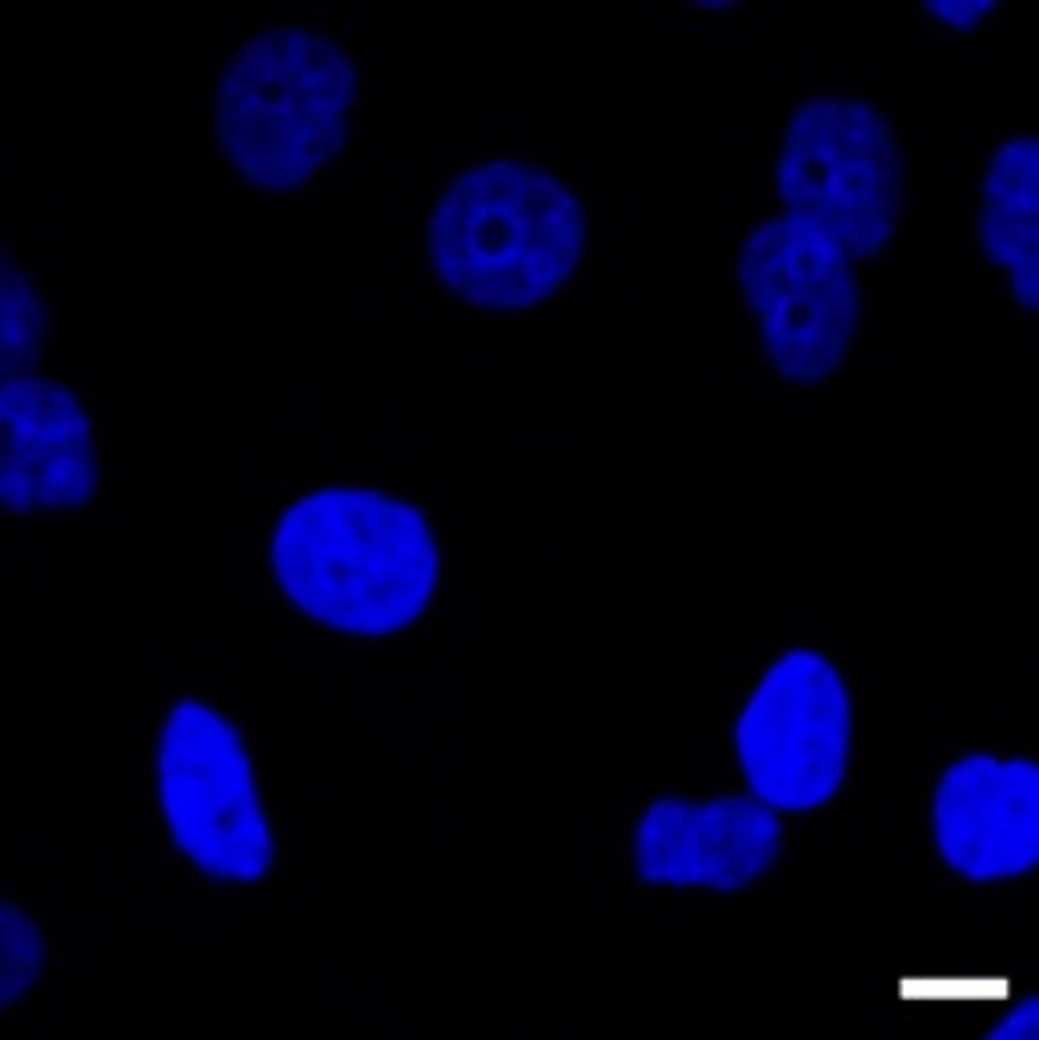

Supplement: Supplementary file 3 — Source data Fig. 1 [file 44319_2024_228_MOESM3_ESM.zip › Figure 1/Figure 1M/DAPI_siRAI14#1.png]

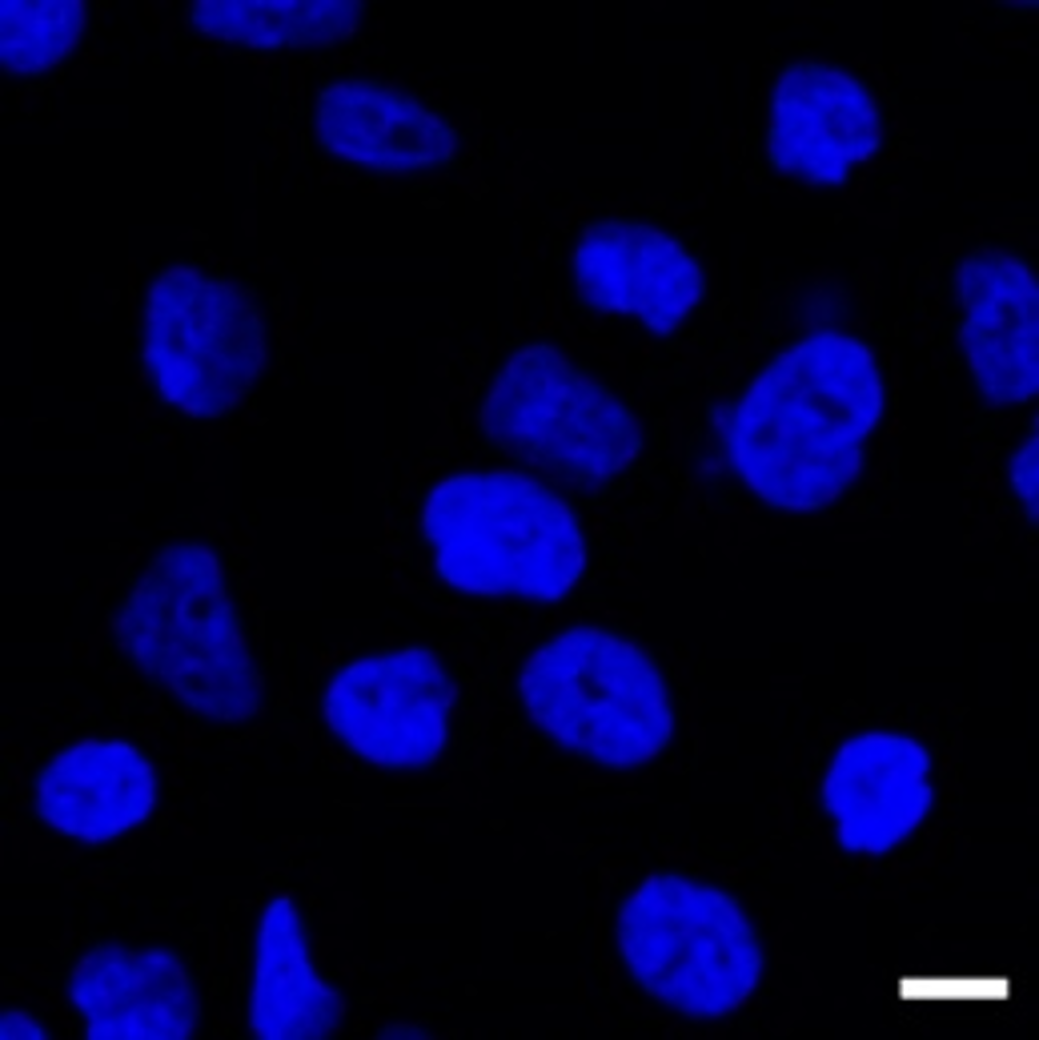

Supplement: Supplementary file 3 — Source data Fig. 1 [file 44319_2024_228_MOESM3_ESM.zip › Figure 1/Figure 1M/DAPI_siRAI14#2.png]

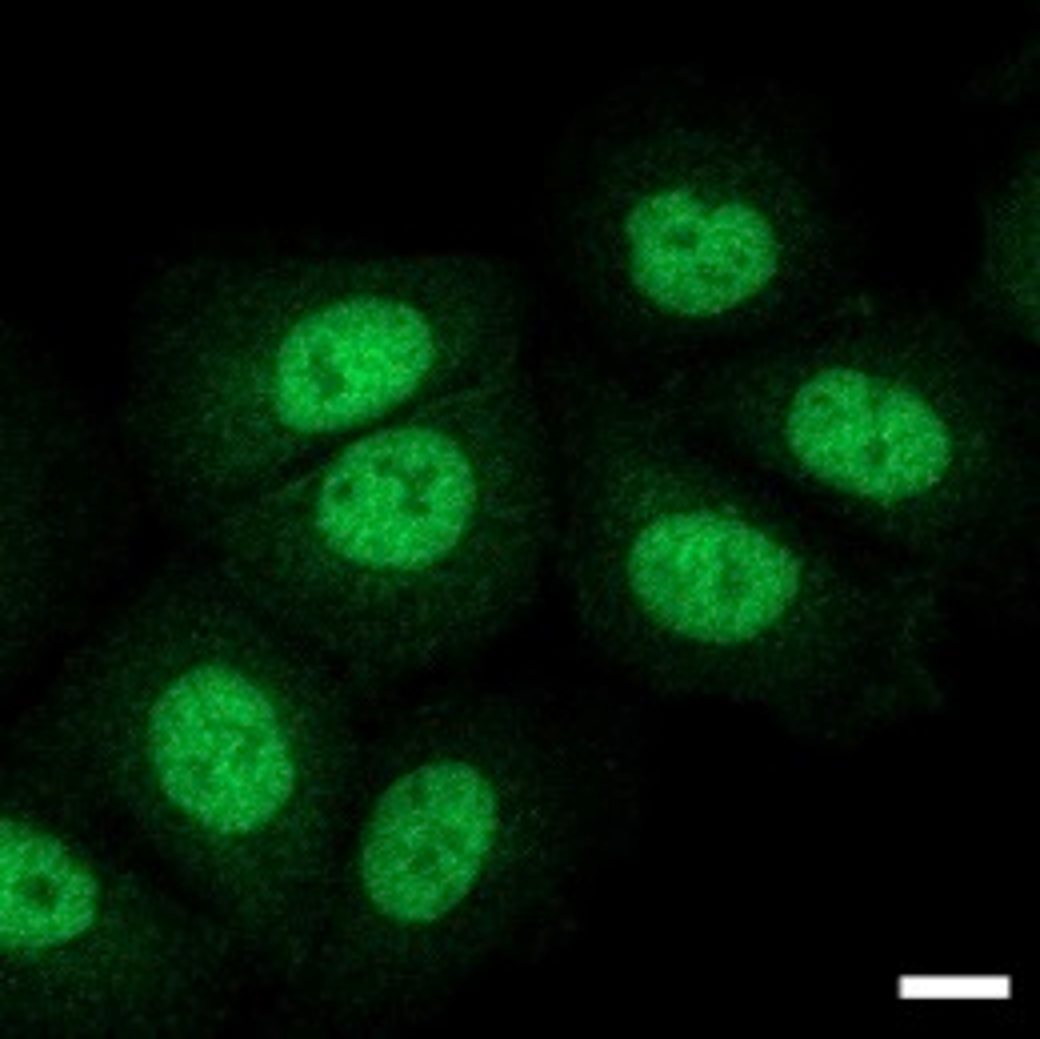

Supplement: Supplementary file 3 — Source data Fig. 1 [file 44319_2024_228_MOESM3_ESM.zip › Figure 1/Figure 1M/YAP_siCtrl.png]

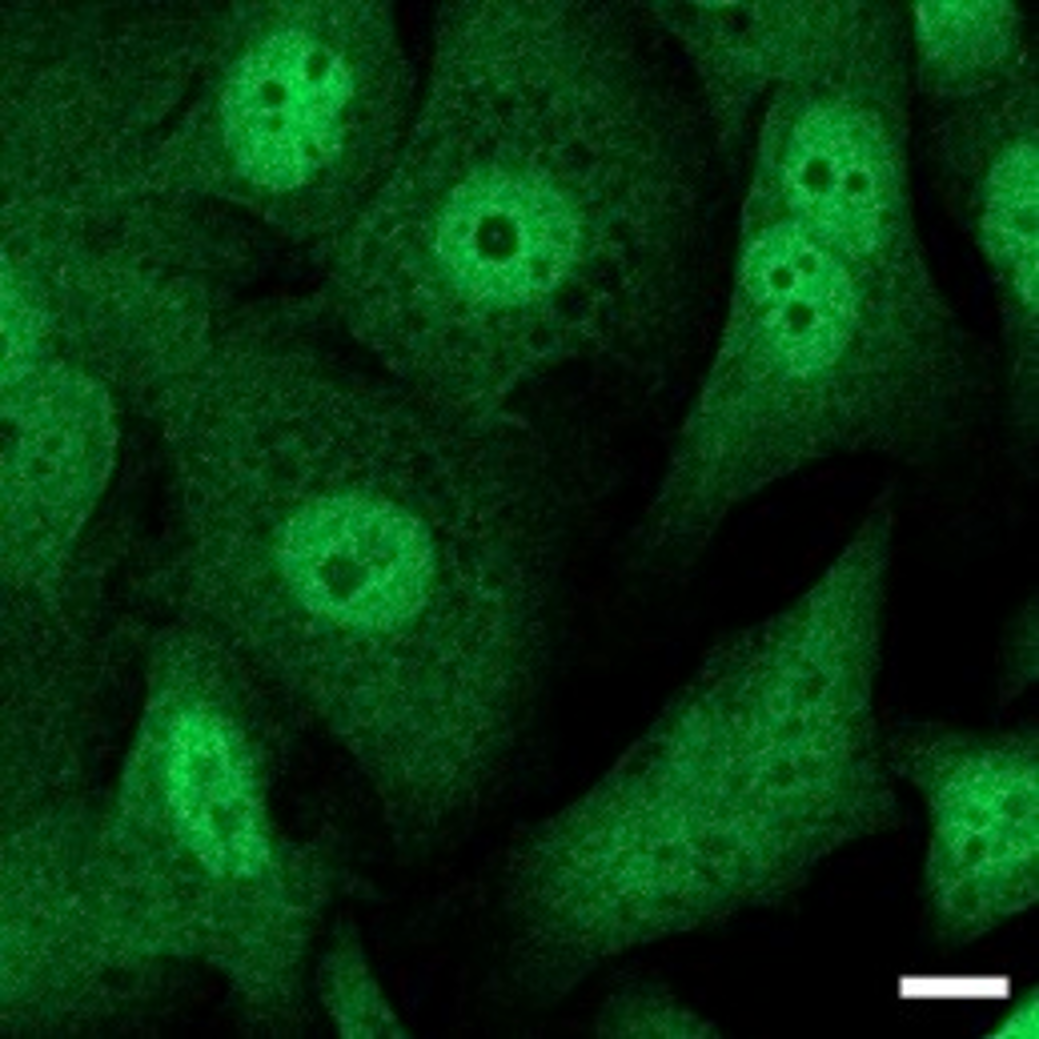

Supplement: Supplementary file 3 — Source data Fig. 1 [file 44319_2024_228_MOESM3_ESM.zip › Figure 1/Figure 1M/YAP_siRAI14#1.png]

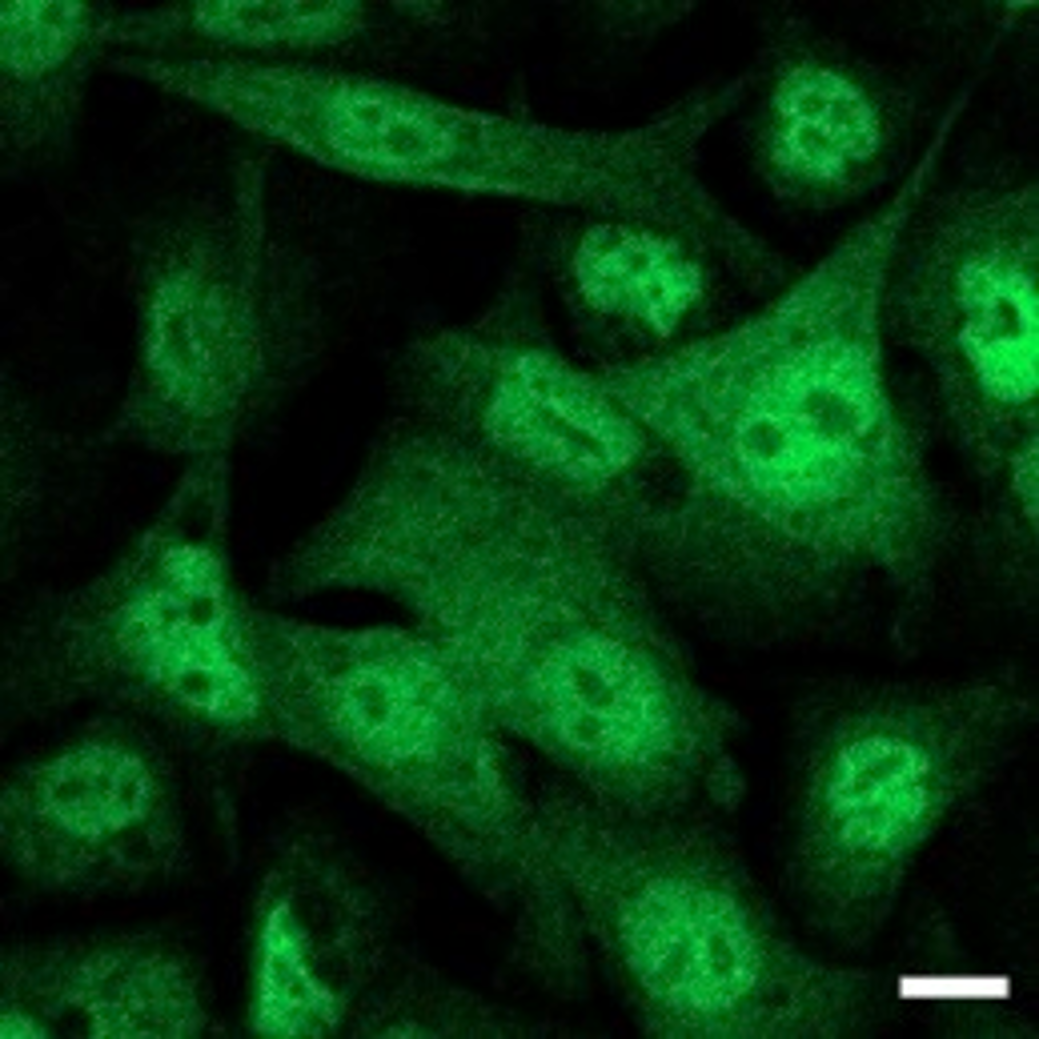

Supplement: Supplementary file 3 — Source data Fig. 1 [file 44319_2024_228_MOESM3_ESM.zip › Figure 1/Figure 1M/YAP_siRAI14#2.png]

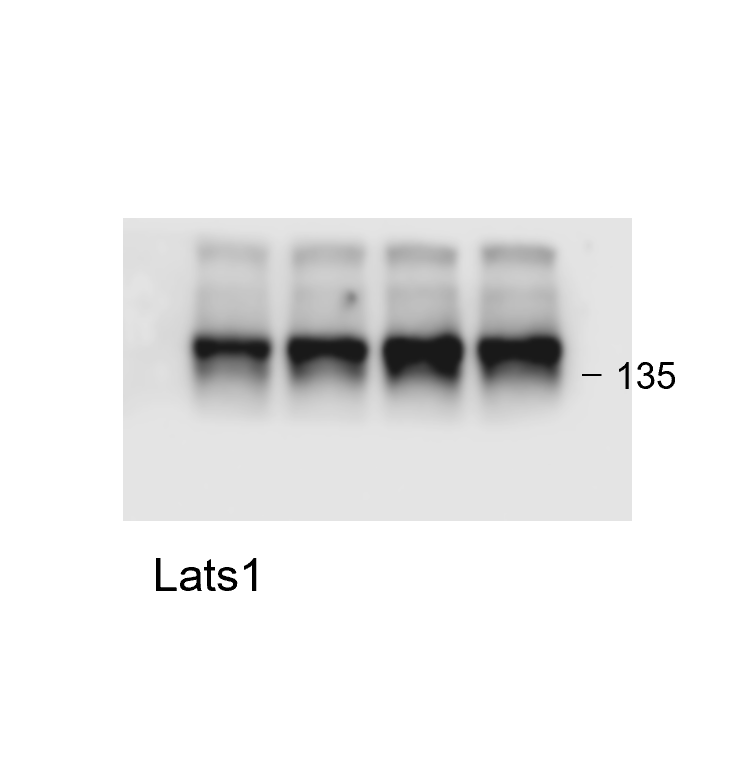

Supplement: Supplementary file 3 — Source data Fig. 1 [file 44319_2024_228_MOESM3_ESM.zip › Figure 1/Figure 1N/Lats1.tif]

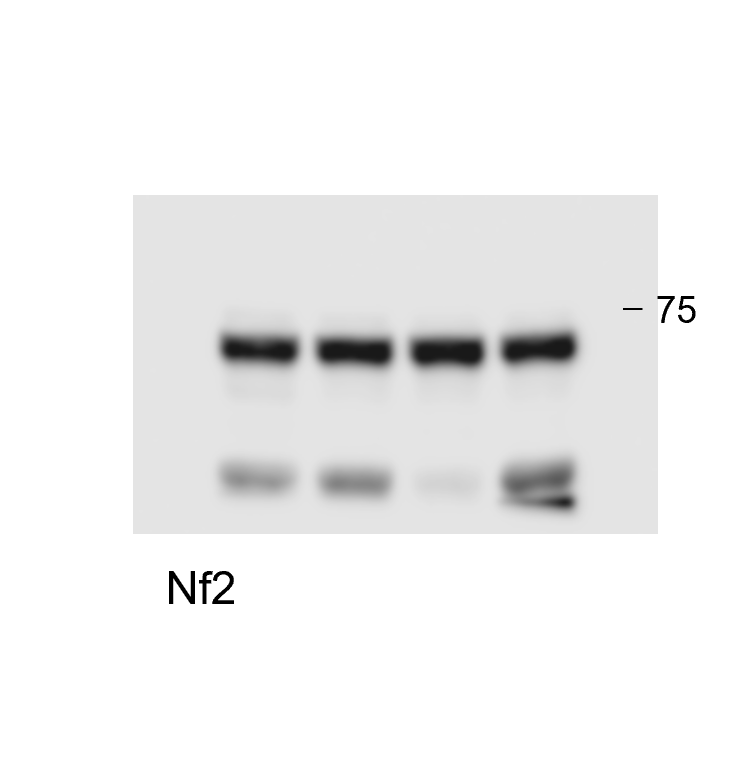

Supplement: Supplementary file 3 — Source data Fig. 1 [file 44319_2024_228_MOESM3_ESM.zip › Figure 1/Figure 1N/Nf2.tif]

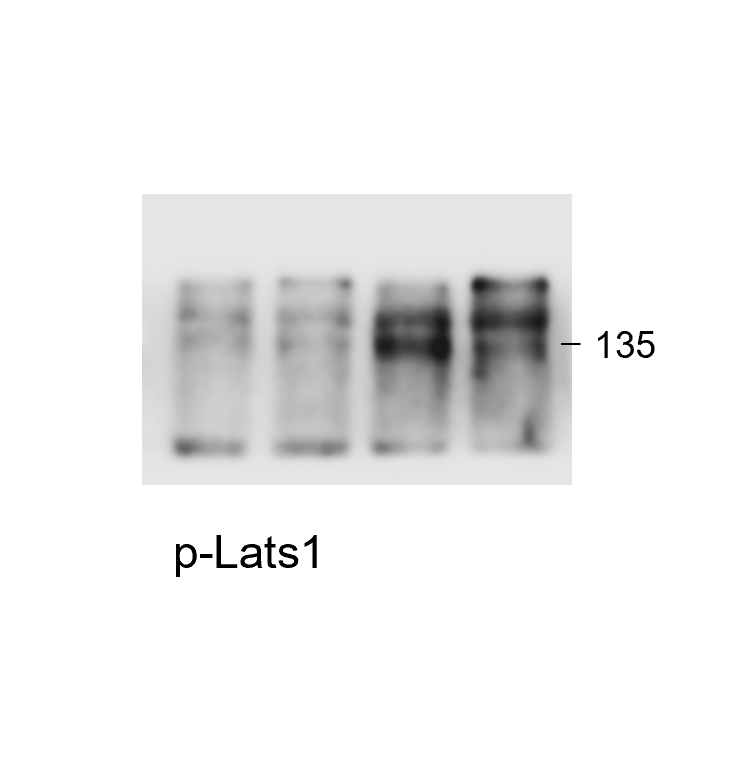

Supplement: Supplementary file 3 — Source data Fig. 1 [file 44319_2024_228_MOESM3_ESM.zip › Figure 1/Figure 1N/p-Lats1.tif]

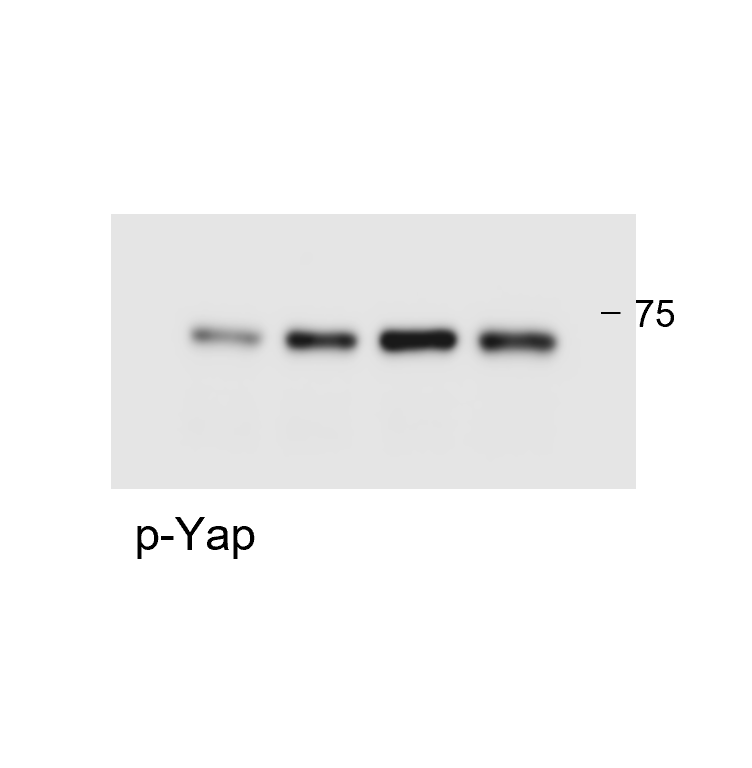

Supplement: Supplementary file 3 — Source data Fig. 1 [file 44319_2024_228_MOESM3_ESM.zip › Figure 1/Figure 1N/p-Yap.tif]

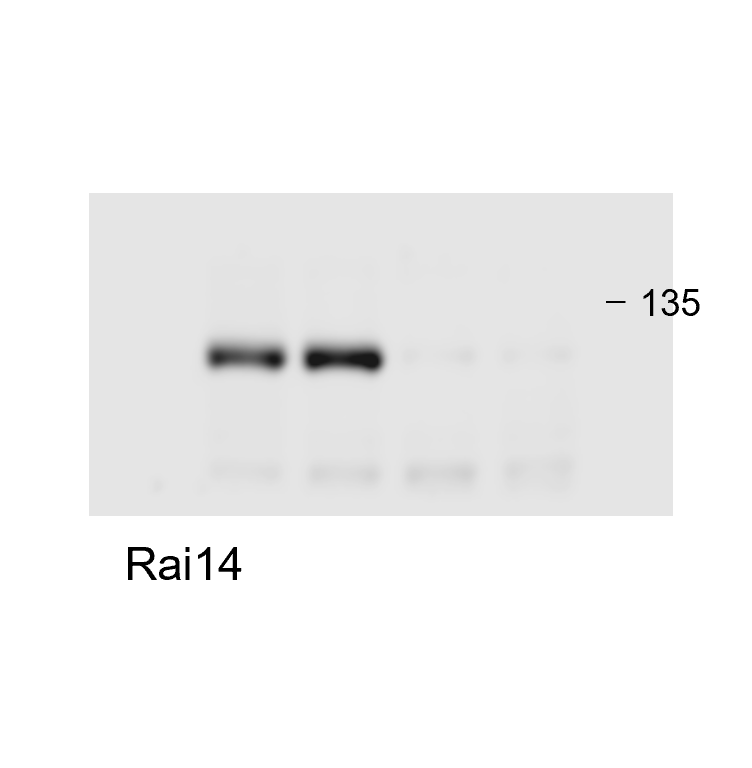

Supplement: Supplementary file 3 — Source data Fig. 1 [file 44319_2024_228_MOESM3_ESM.zip › Figure 1/Figure 1N/Rai14.tif]

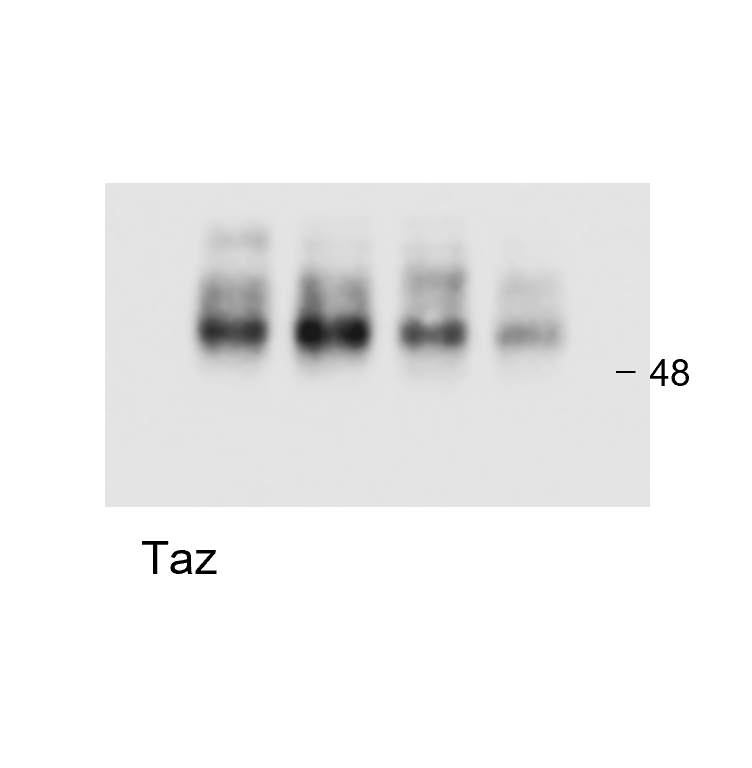

Supplement: Supplementary file 3 — Source data Fig. 1 [file 44319_2024_228_MOESM3_ESM.zip › Figure 1/Figure 1N/Taz.tif]

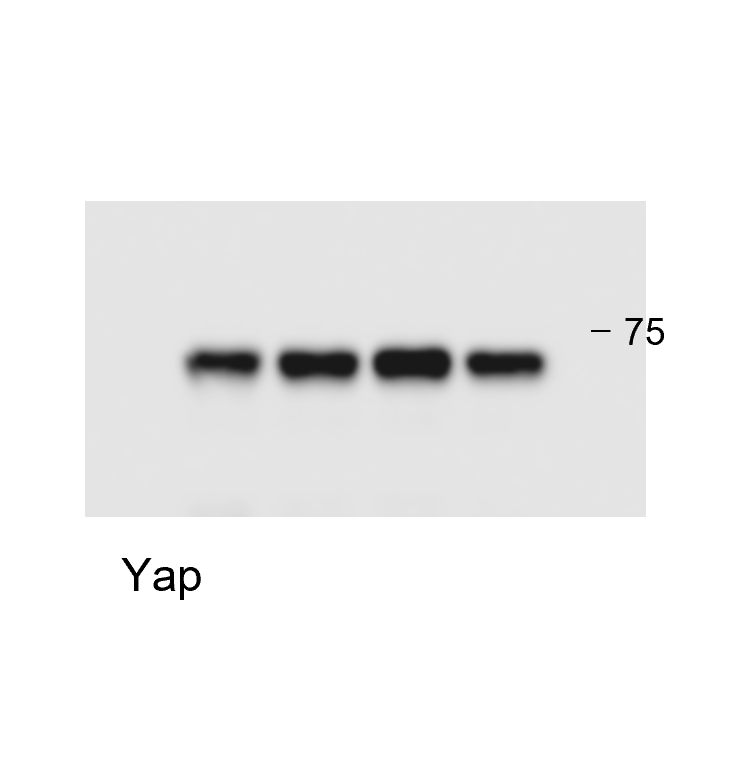

Supplement: Supplementary file 3 — Source data Fig. 1 [file 44319_2024_228_MOESM3_ESM.zip › Figure 1/Figure 1N/Yap.tif]

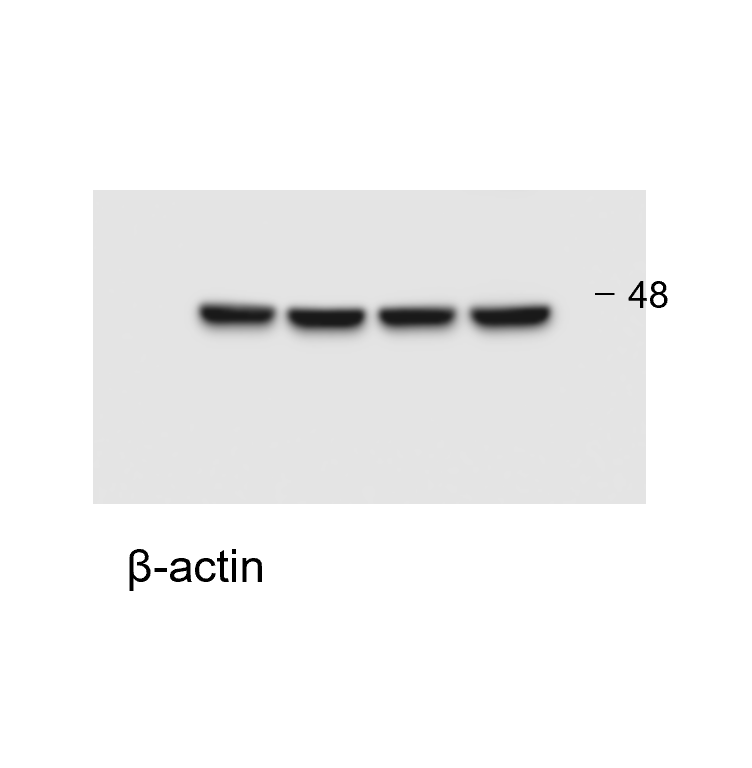

Supplement: Supplementary file 3 — Source data Fig. 1 [file 44319_2024_228_MOESM3_ESM.zip › Figure 1/Figure 1N/ÑΓ-actin.tif]

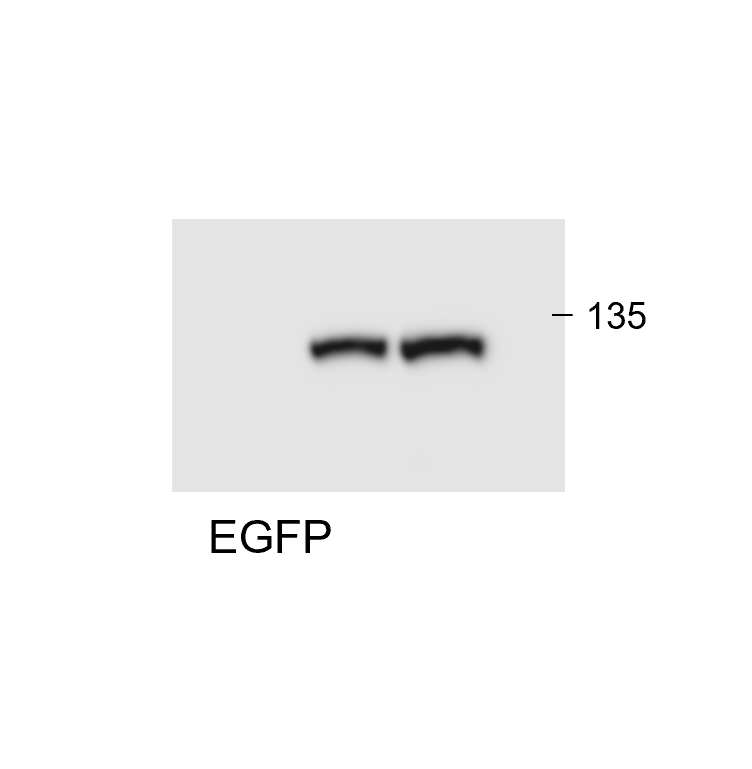

Supplement: Supplementary file 4 — Source data Fig. 2 [file 44319_2024_228_MOESM4_ESM.zip › Figure 2/Figure 2A/EGFP.tif]

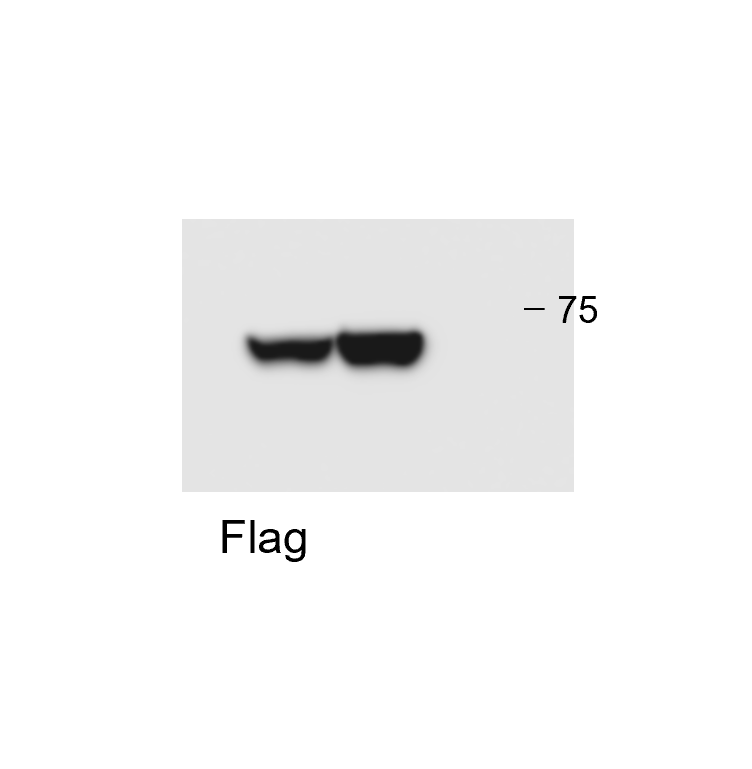

Supplement: Supplementary file 4 — Source data Fig. 2 [file 44319_2024_228_MOESM4_ESM.zip › Figure 2/Figure 2A/Flag.tif]

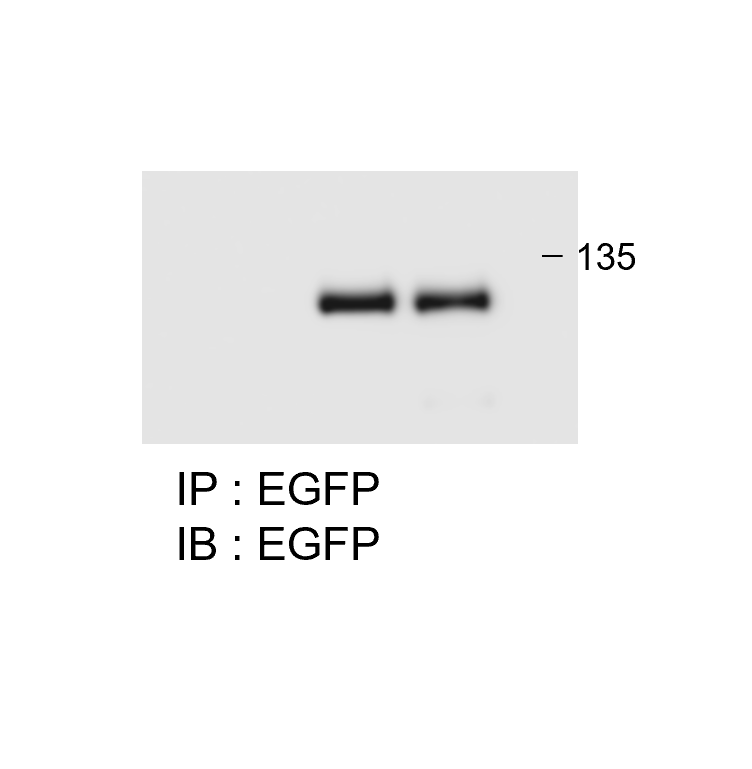

Supplement: Supplementary file 4 — Source data Fig. 2 [file 44319_2024_228_MOESM4_ESM.zip › Figure 2/Figure 2A/IP EGFP, IB EGFP.tif]

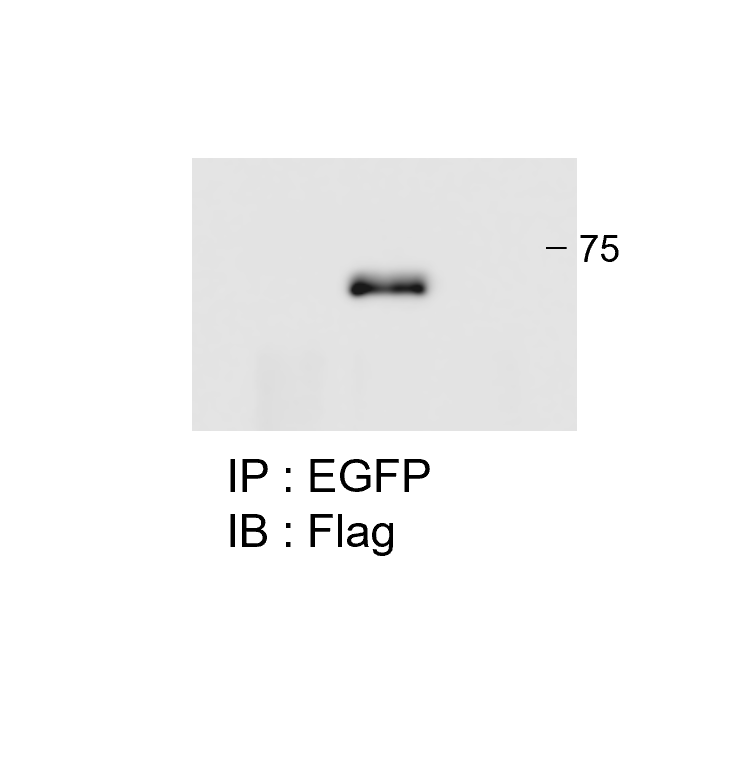

Supplement: Supplementary file 4 — Source data Fig. 2 [file 44319_2024_228_MOESM4_ESM.zip › Figure 2/Figure 2A/IP EGFP, IB Flag.tif]

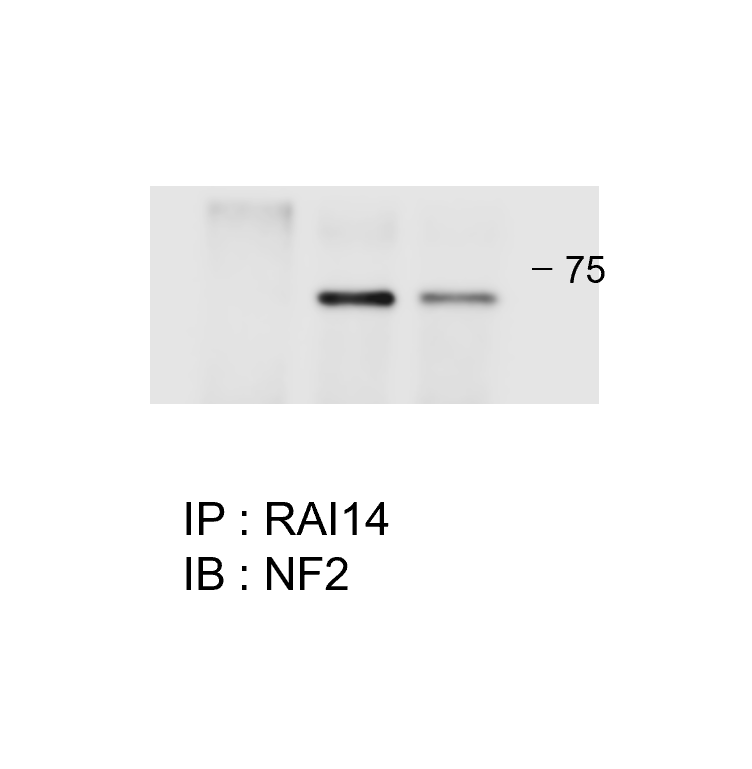

Supplement: Supplementary file 4 — Source data Fig. 2 [file 44319_2024_228_MOESM4_ESM.zip › Figure 2/Figure 2B/IP RAI14, IB NF2.tif]

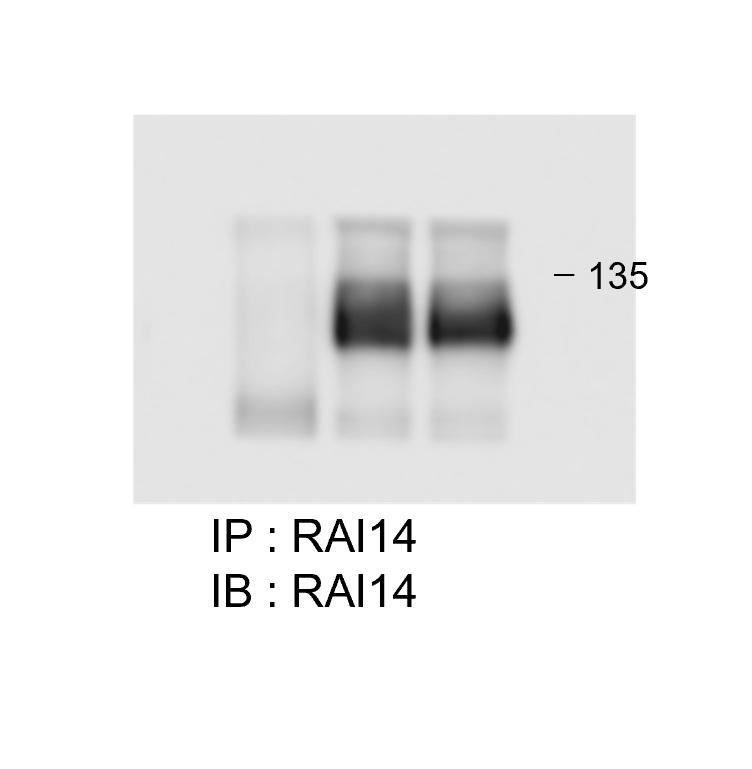

Supplement: Supplementary file 4 — Source data Fig. 2 [file 44319_2024_228_MOESM4_ESM.zip › Figure 2/Figure 2B/IP RAI14, IB RAI14.tif]

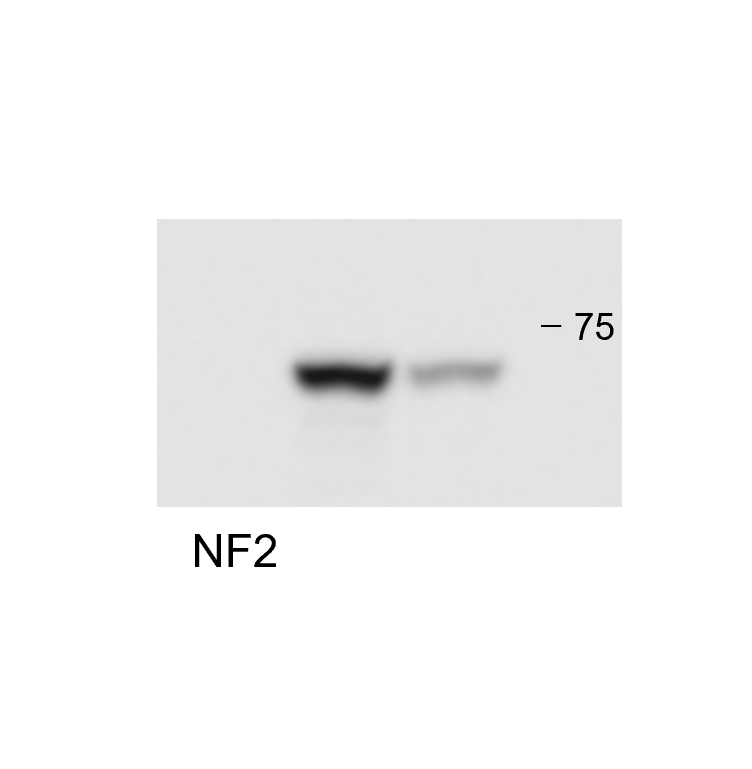

Supplement: Supplementary file 4 — Source data Fig. 2 [file 44319_2024_228_MOESM4_ESM.zip › Figure 2/Figure 2B/NF2.tif]

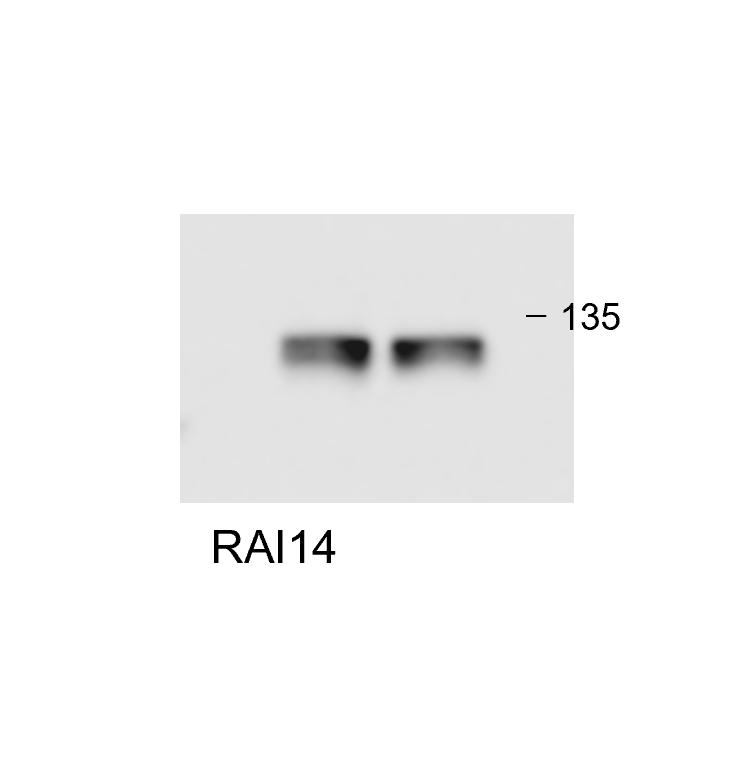

Supplement: Supplementary file 4 — Source data Fig. 2 [file 44319_2024_228_MOESM4_ESM.zip › Figure 2/Figure 2B/RAI14.tif]

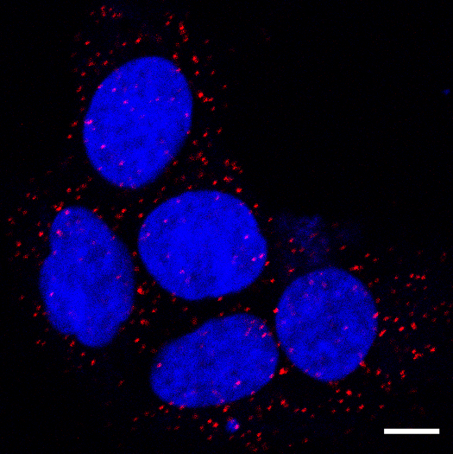

Supplement: Supplementary file 4 — Source data Fig. 2 [file 44319_2024_228_MOESM4_ESM.zip › Figure 2/Figure 2C/NF2, RAI14.tif]

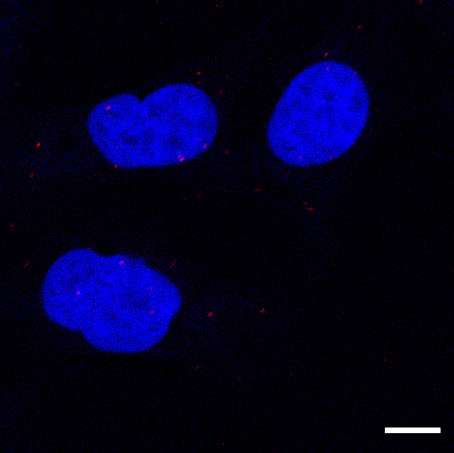

Supplement: Supplementary file 4 — Source data Fig. 2 [file 44319_2024_228_MOESM4_ESM.zip › Figure 2/Figure 2C/NF2.tif]

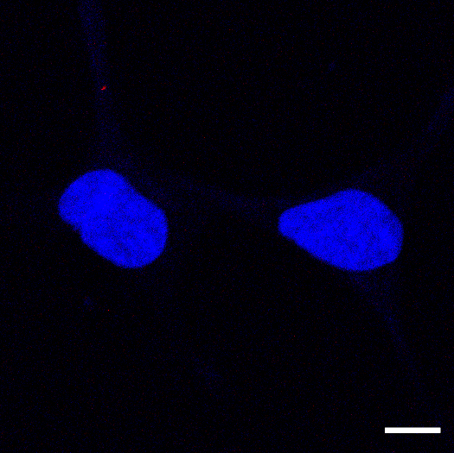

Supplement: Supplementary file 4 — Source data Fig. 2 [file 44319_2024_228_MOESM4_ESM.zip › Figure 2/Figure 2C/no antibody.tif]

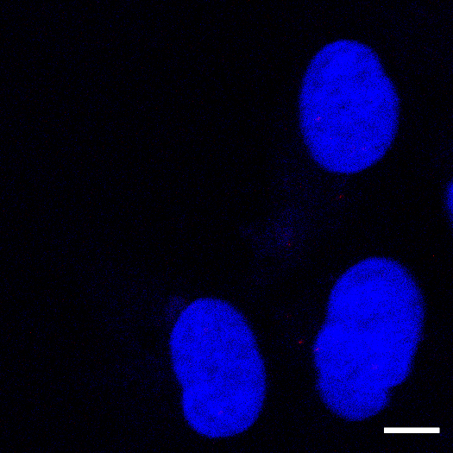

Supplement: Supplementary file 4 — Source data Fig. 2 [file 44319_2024_228_MOESM4_ESM.zip › Figure 2/Figure 2C/RAI14.tif]

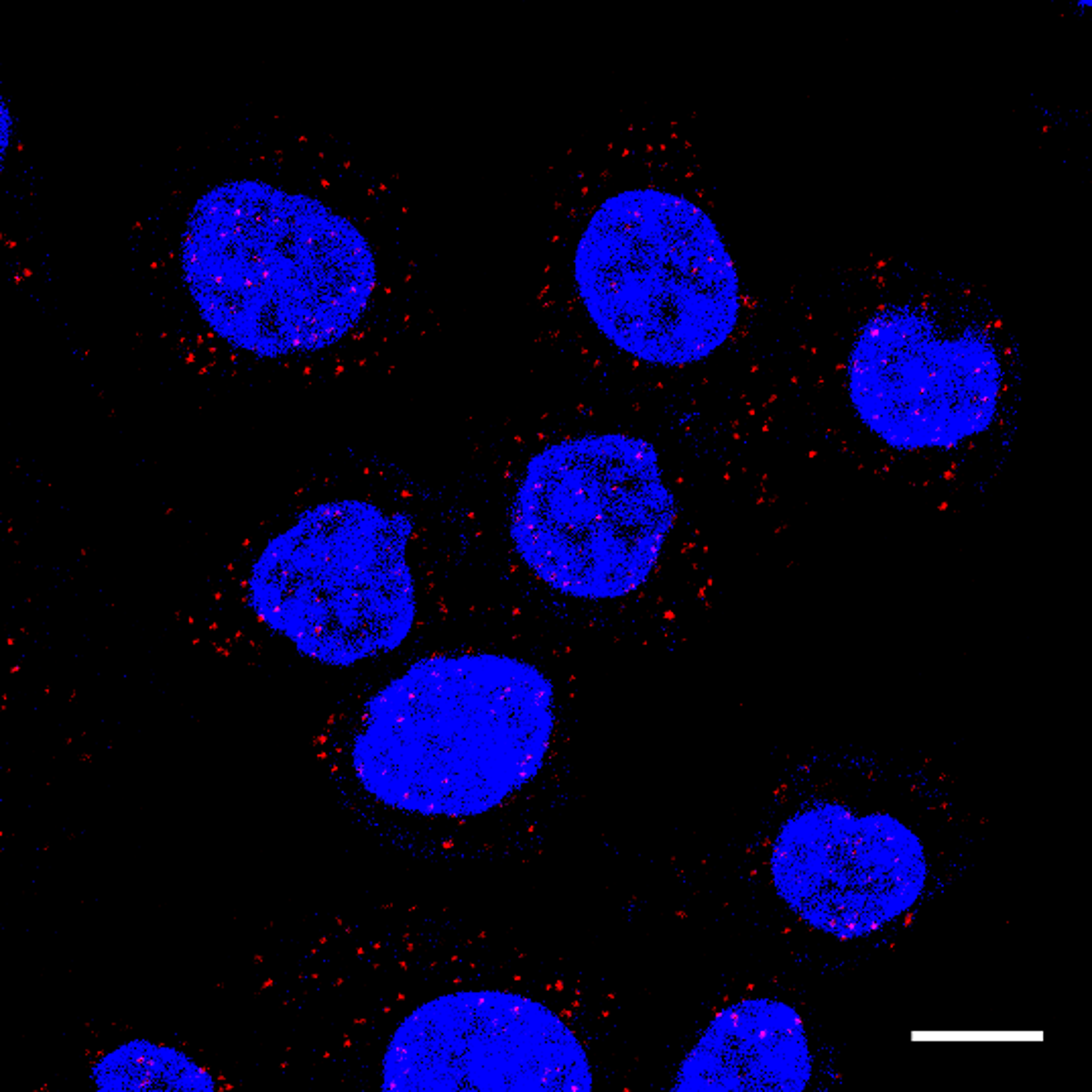

Supplement: Supplementary file 4 — Source data Fig. 2 [file 44319_2024_228_MOESM4_ESM.zip › Figure 2/Figure 2D/NF2, RAI14.tif]

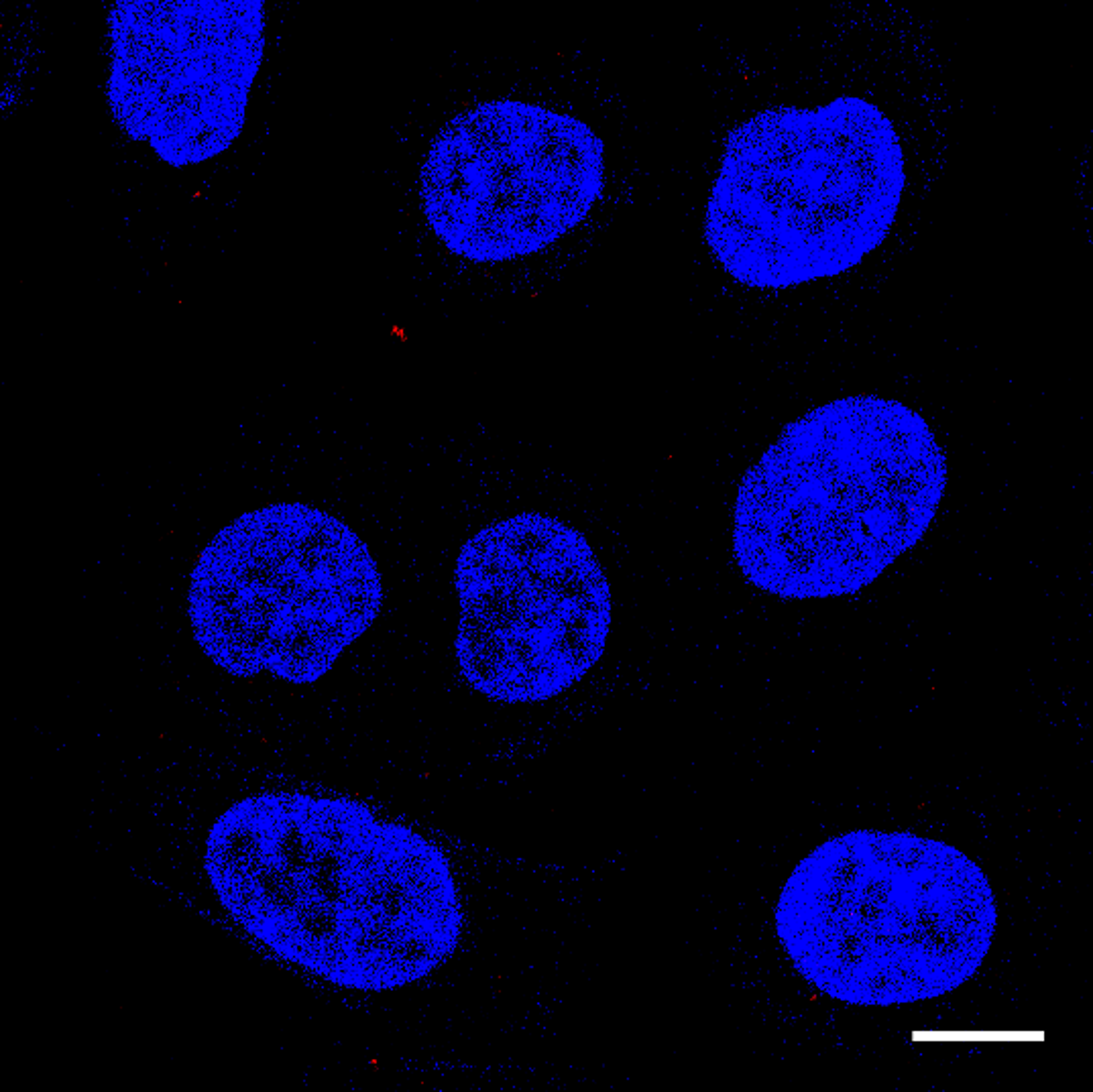

Supplement: Supplementary file 4 — Source data Fig. 2 [file 44319_2024_228_MOESM4_ESM.zip › Figure 2/Figure 2D/NF2.tif]

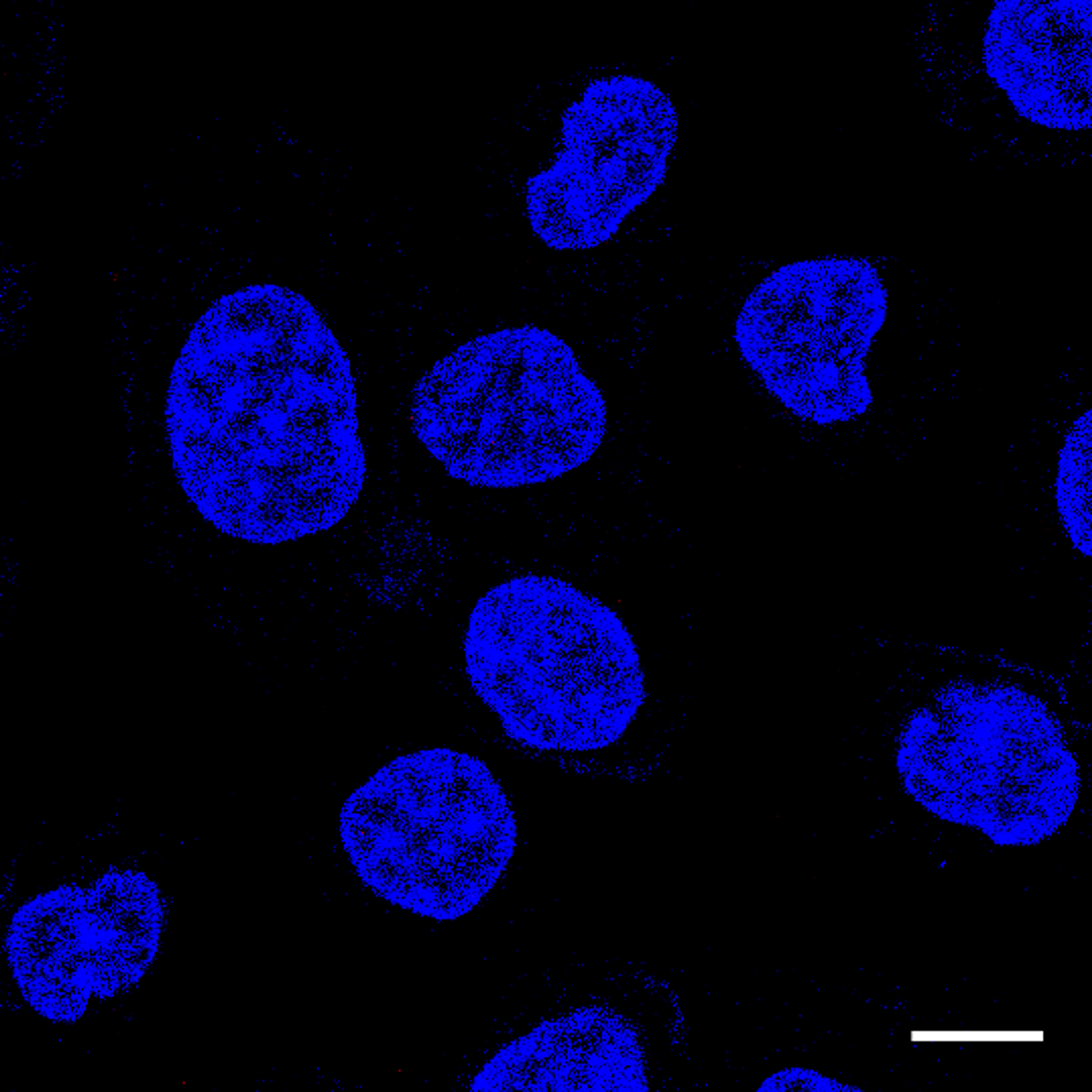

Supplement: Supplementary file 4 — Source data Fig. 2 [file 44319_2024_228_MOESM4_ESM.zip › Figure 2/Figure 2D/no antibody.tif]

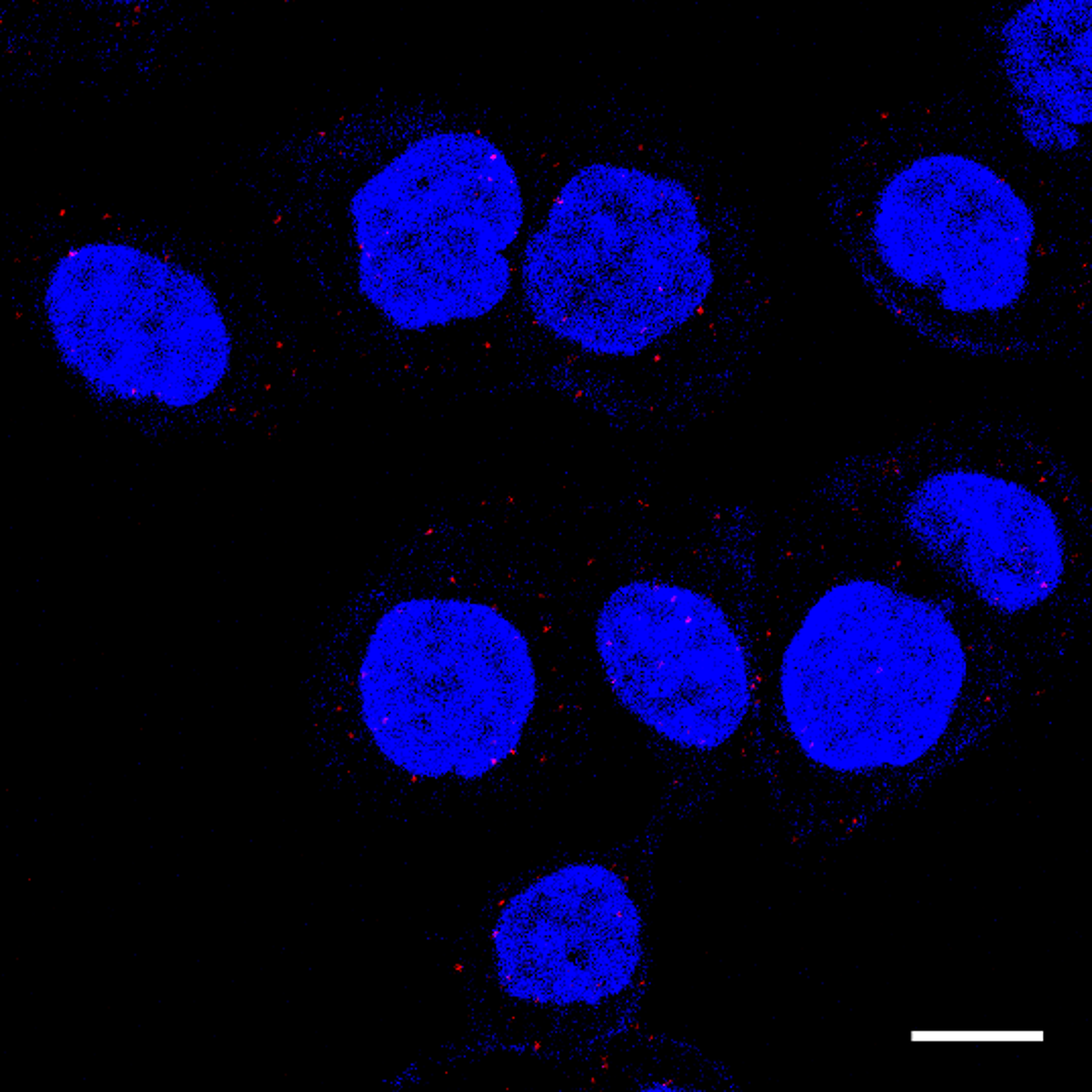

Supplement: Supplementary file 4 — Source data Fig. 2 [file 44319_2024_228_MOESM4_ESM.zip › Figure 2/Figure 2D/RAI14.tif]

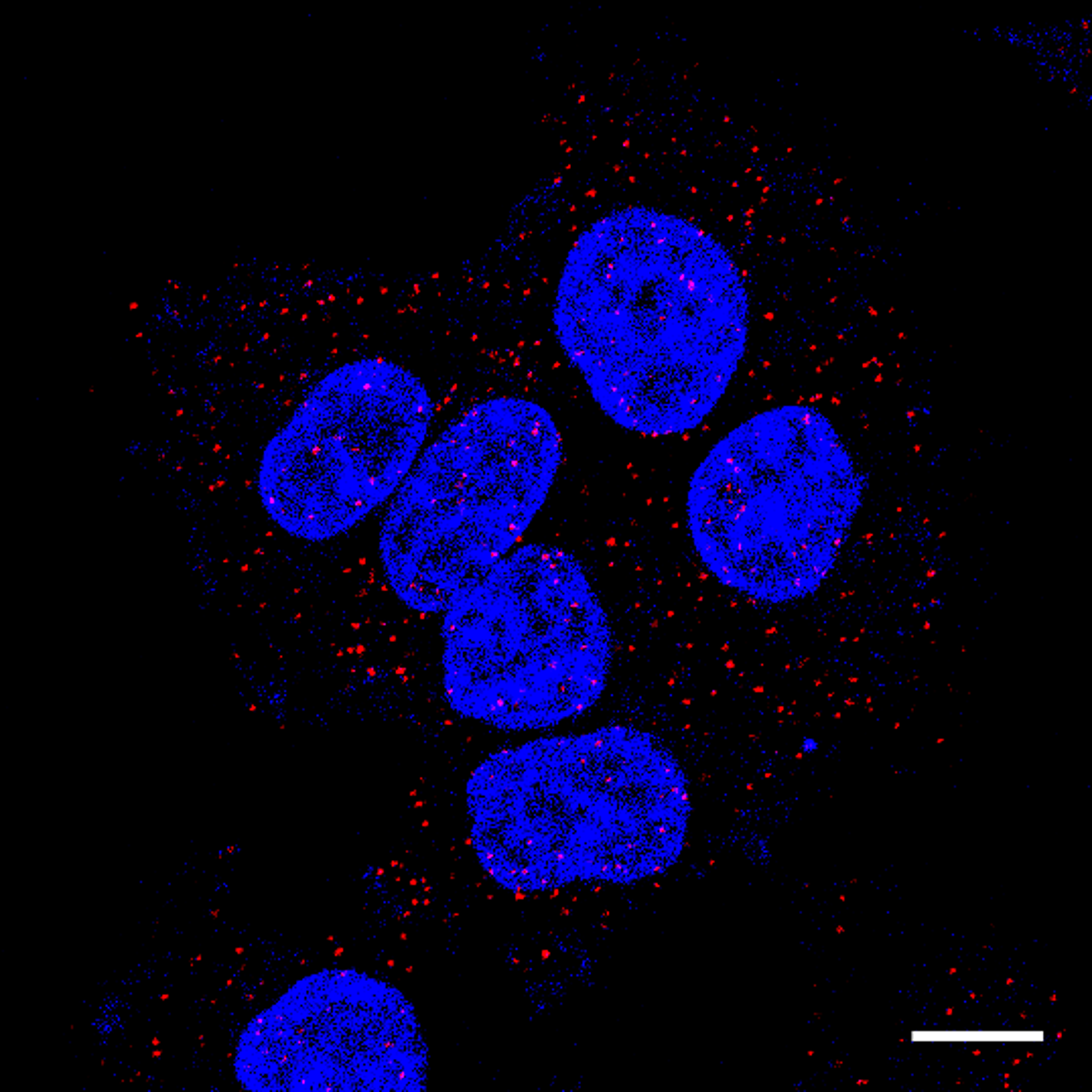

Supplement: Supplementary file 4 — Source data Fig. 2 [file 44319_2024_228_MOESM4_ESM.zip › Figure 2/Figure 2E/NF2, RAI14.tif]

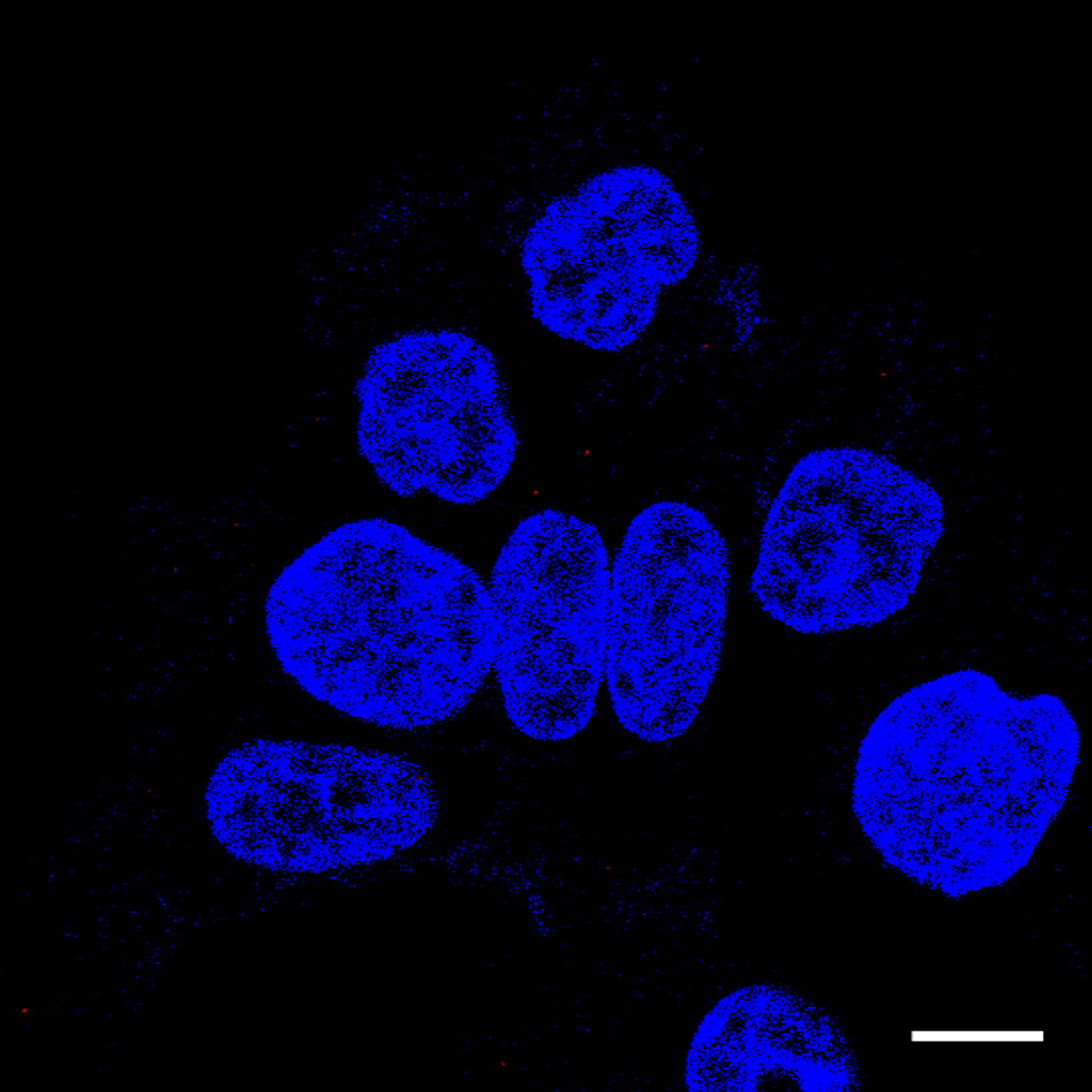

Supplement: Supplementary file 4 — Source data Fig. 2 [file 44319_2024_228_MOESM4_ESM.zip › Figure 2/Figure 2E/NF2.tif]

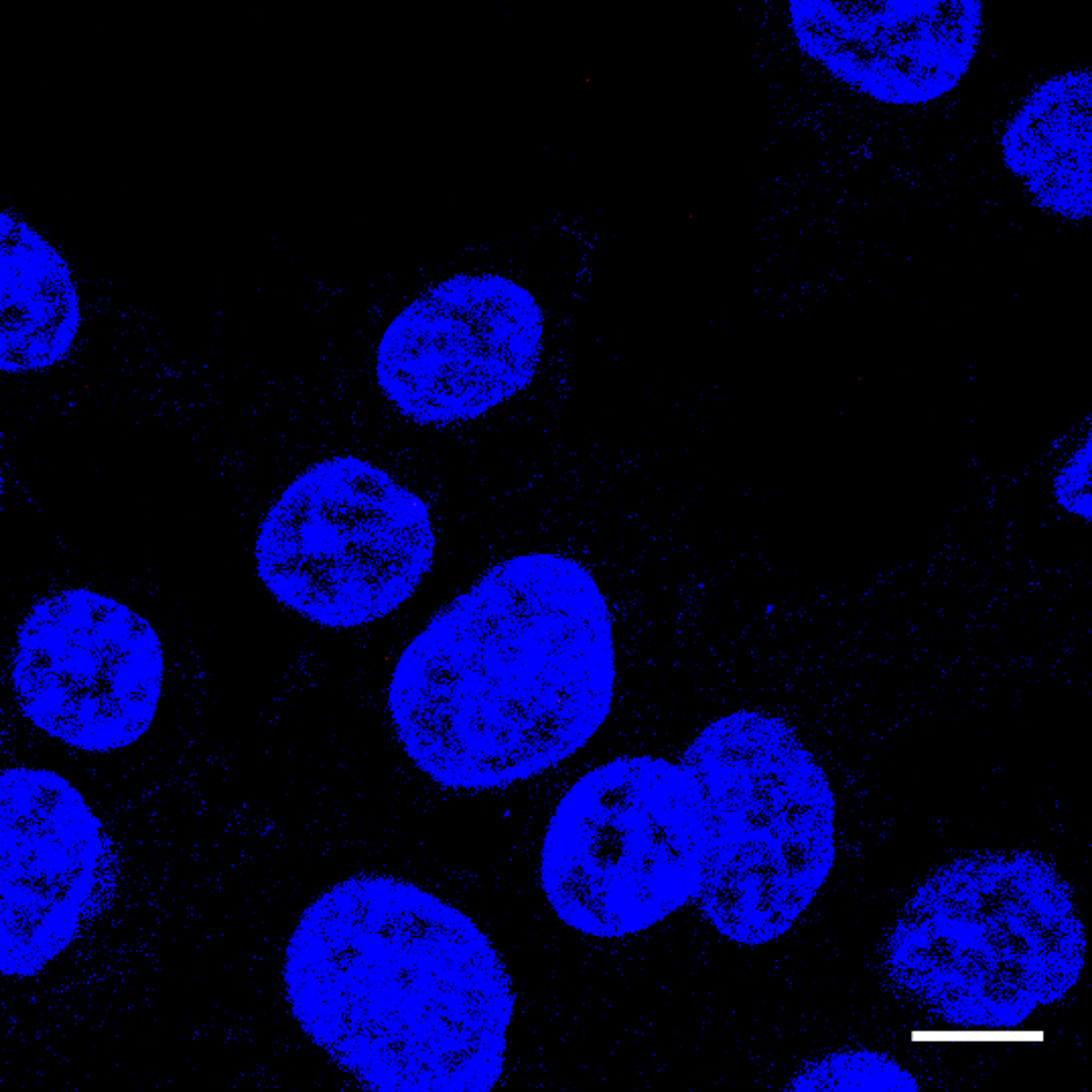

Supplement: Supplementary file 4 — Source data Fig. 2 [file 44319_2024_228_MOESM4_ESM.zip › Figure 2/Figure 2E/no antibody.tif]

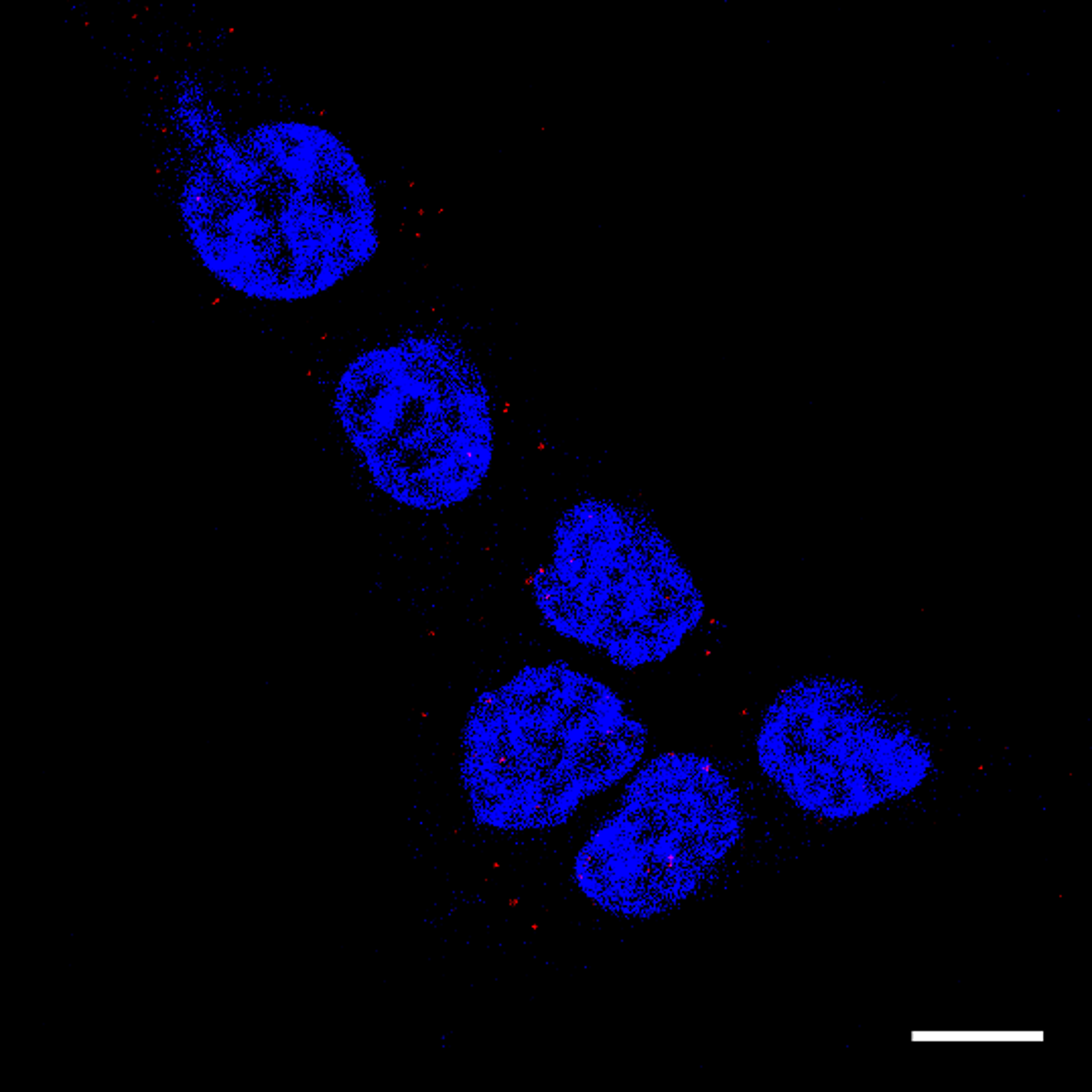

Supplement: Supplementary file 4 — Source data Fig. 2 [file 44319_2024_228_MOESM4_ESM.zip › Figure 2/Figure 2E/RAI14.tif]

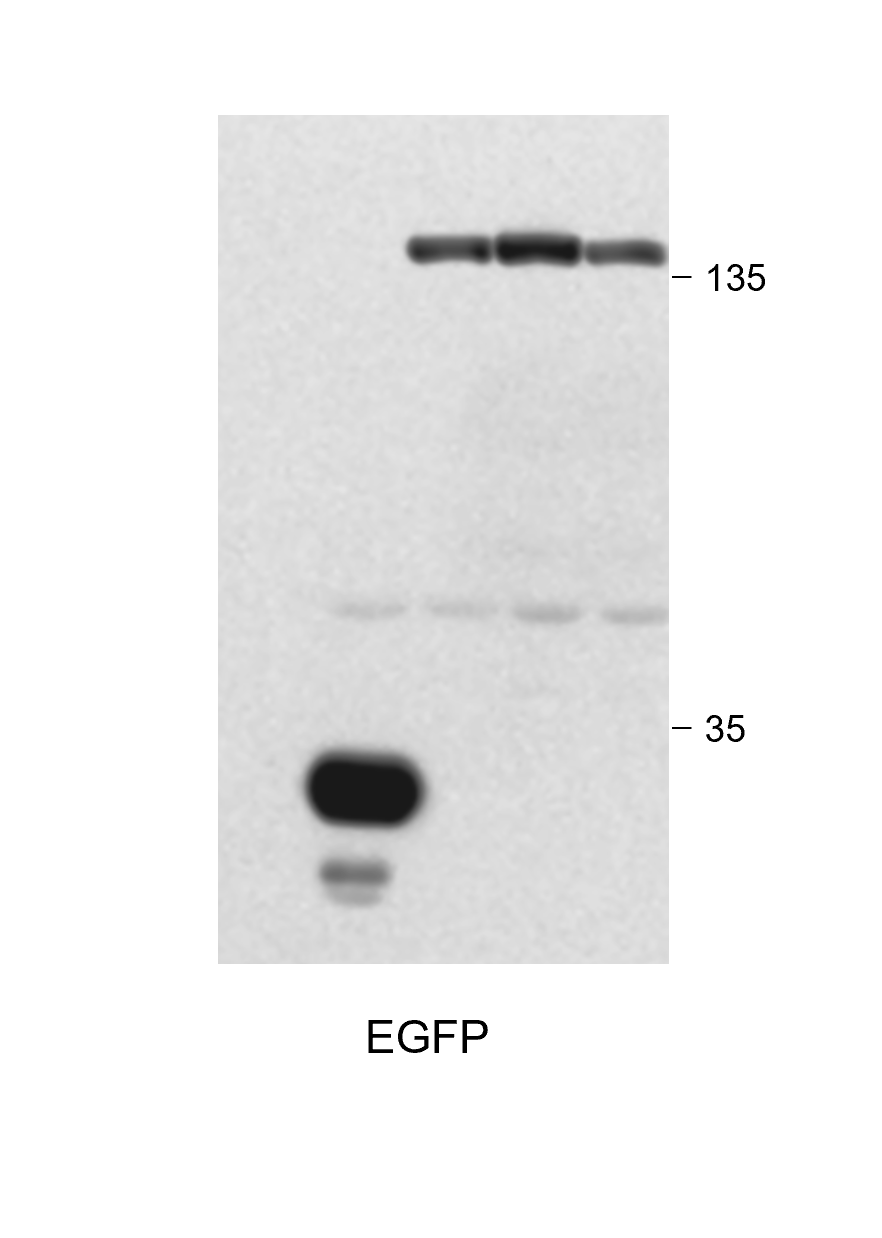

Supplement: Supplementary file 4 — Source data Fig. 2 [file 44319_2024_228_MOESM4_ESM.zip › Figure 2/Figure 2G/EGFP.tif]

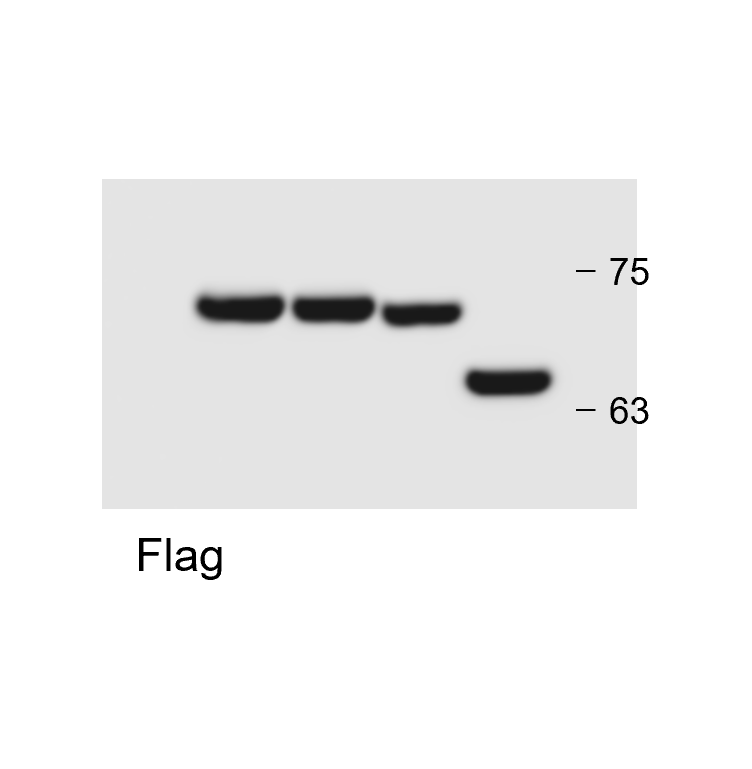

Supplement: Supplementary file 4 — Source data Fig. 2 [file 44319_2024_228_MOESM4_ESM.zip › Figure 2/Figure 2G/Flag.tif]

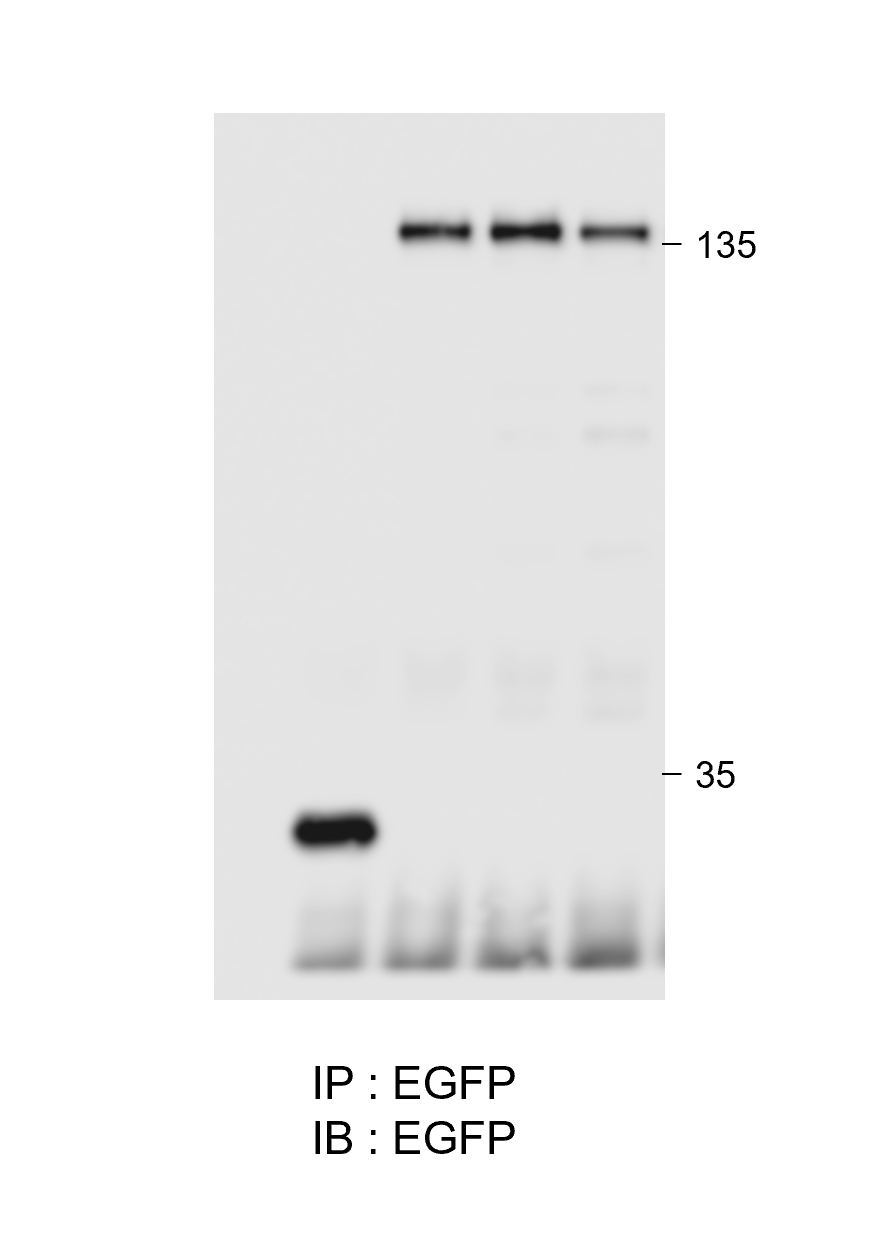

Supplement: Supplementary file 4 — Source data Fig. 2 [file 44319_2024_228_MOESM4_ESM.zip › Figure 2/Figure 2G/IP EGFP, IB EGFP.tif]

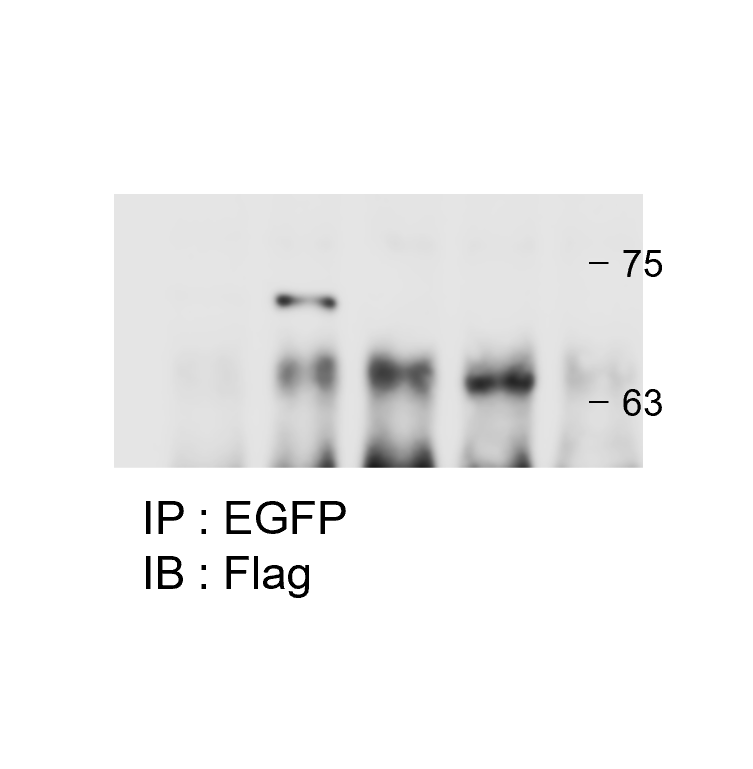

Supplement: Supplementary file 4 — Source data Fig. 2 [file 44319_2024_228_MOESM4_ESM.zip › Figure 2/Figure 2G/IP EGFP, IB Flag.tif]

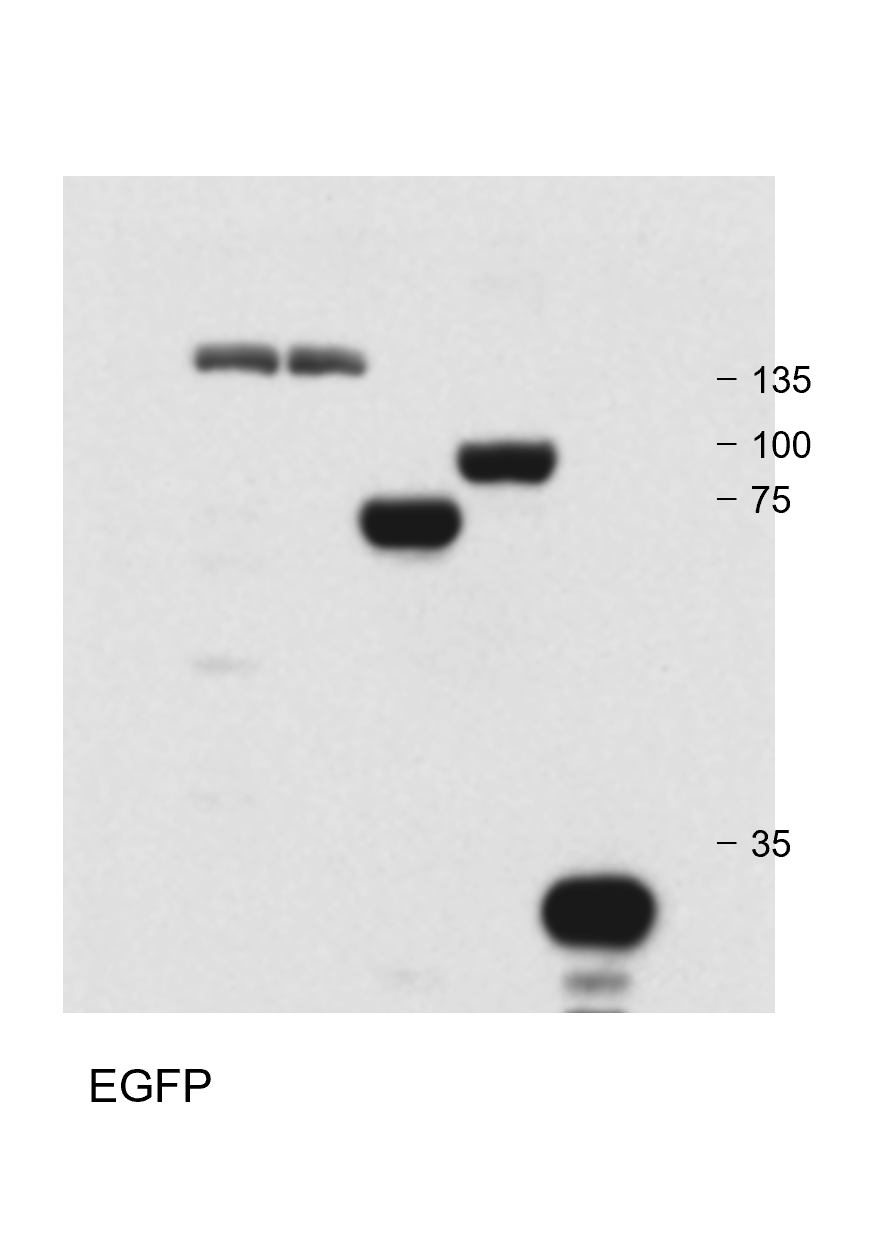

Supplement: Supplementary file 4 — Source data Fig. 2 [file 44319_2024_228_MOESM4_ESM.zip › Figure 2/Figure 2H/EGFP.tif]

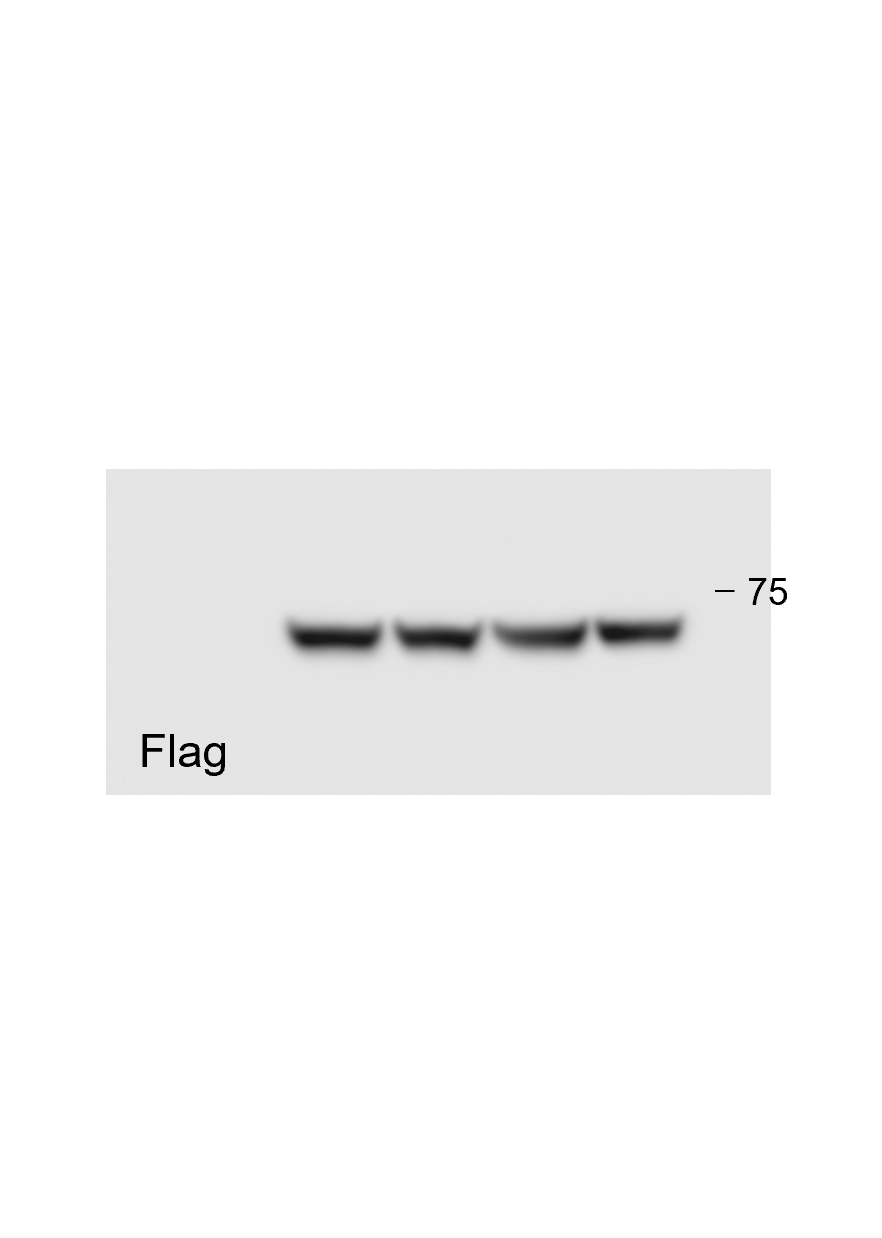

Supplement: Supplementary file 4 — Source data Fig. 2 [file 44319_2024_228_MOESM4_ESM.zip › Figure 2/Figure 2H/Flag.tif]

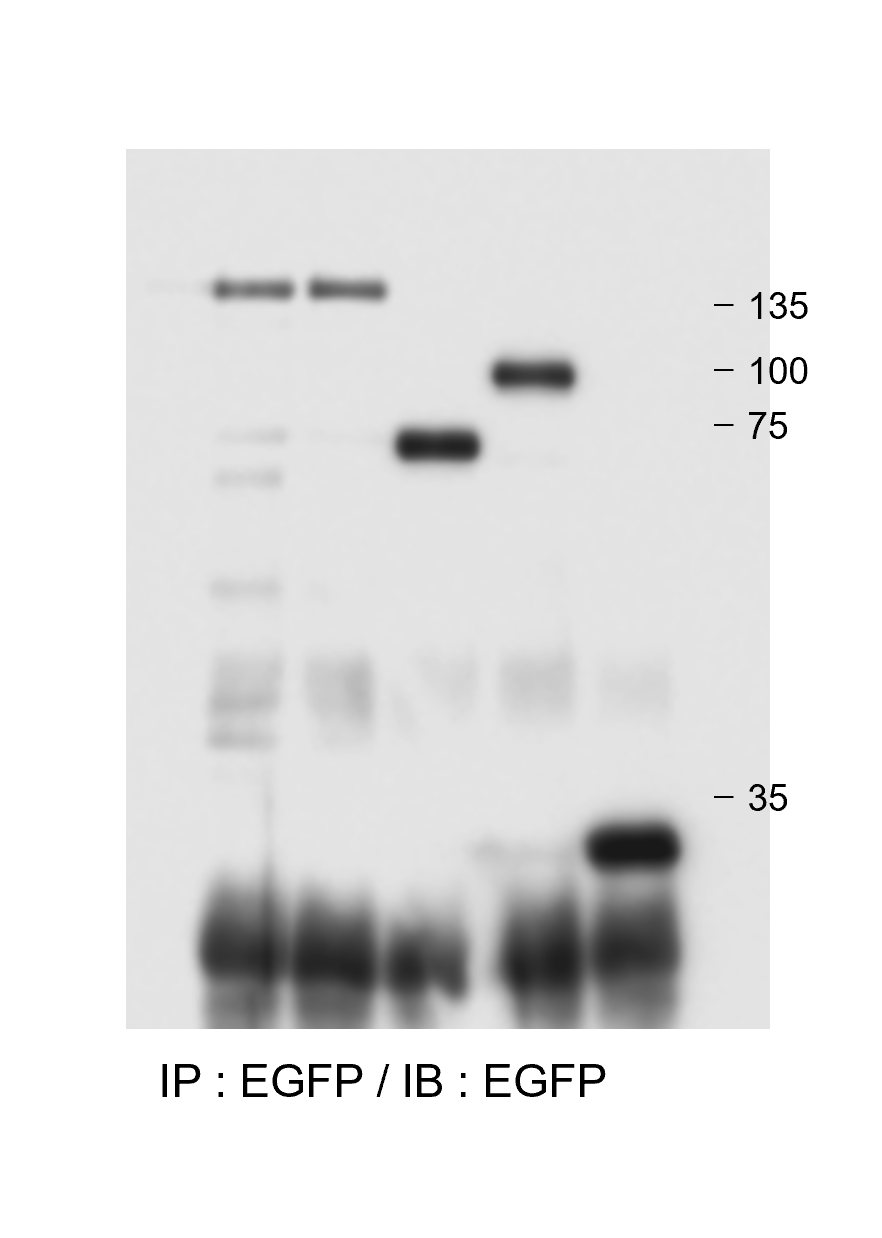

Supplement: Supplementary file 4 — Source data Fig. 2 [file 44319_2024_228_MOESM4_ESM.zip › Figure 2/Figure 2H/IP EGFP, IB EGFP.tif]

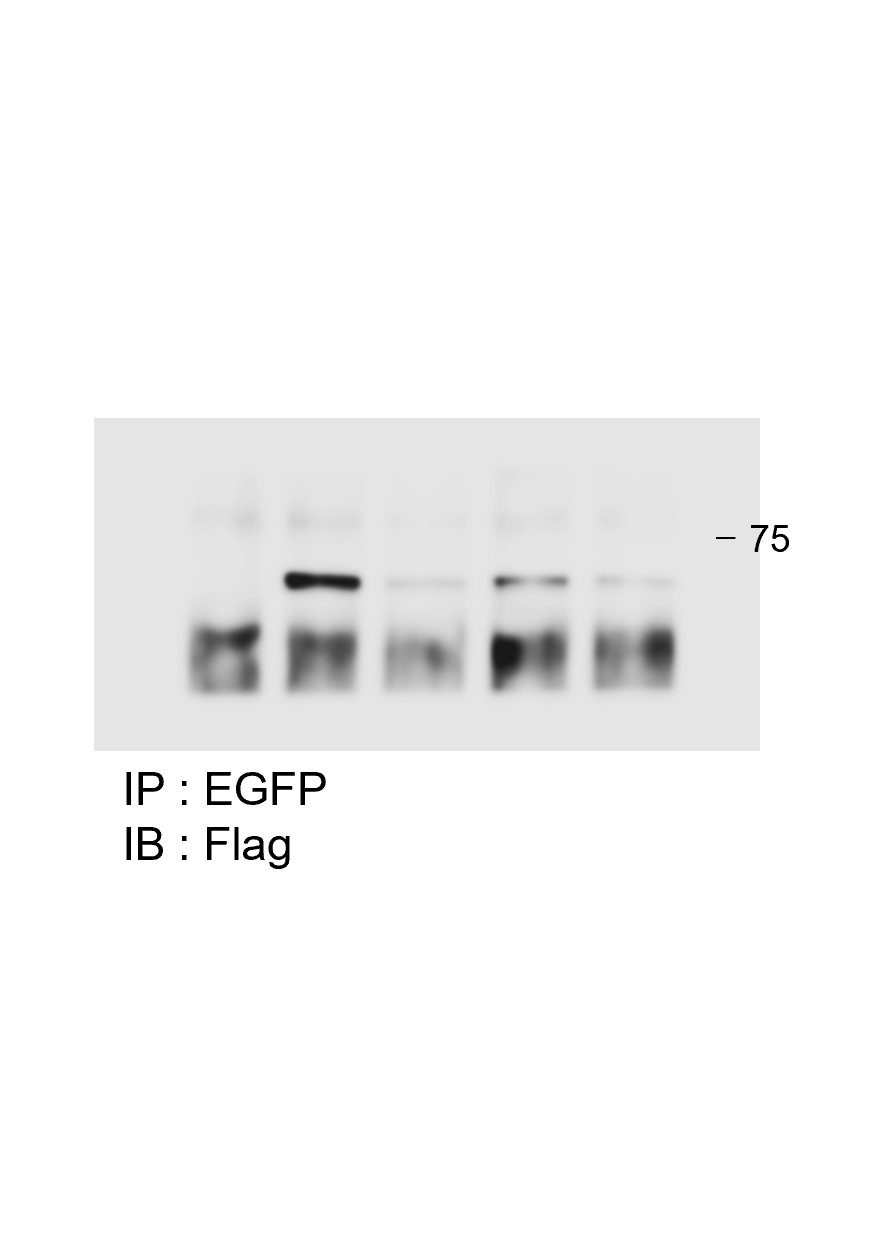

Supplement: Supplementary file 4 — Source data Fig. 2 [file 44319_2024_228_MOESM4_ESM.zip › Figure 2/Figure 2H/IP EGFP, IB Flag.tif]

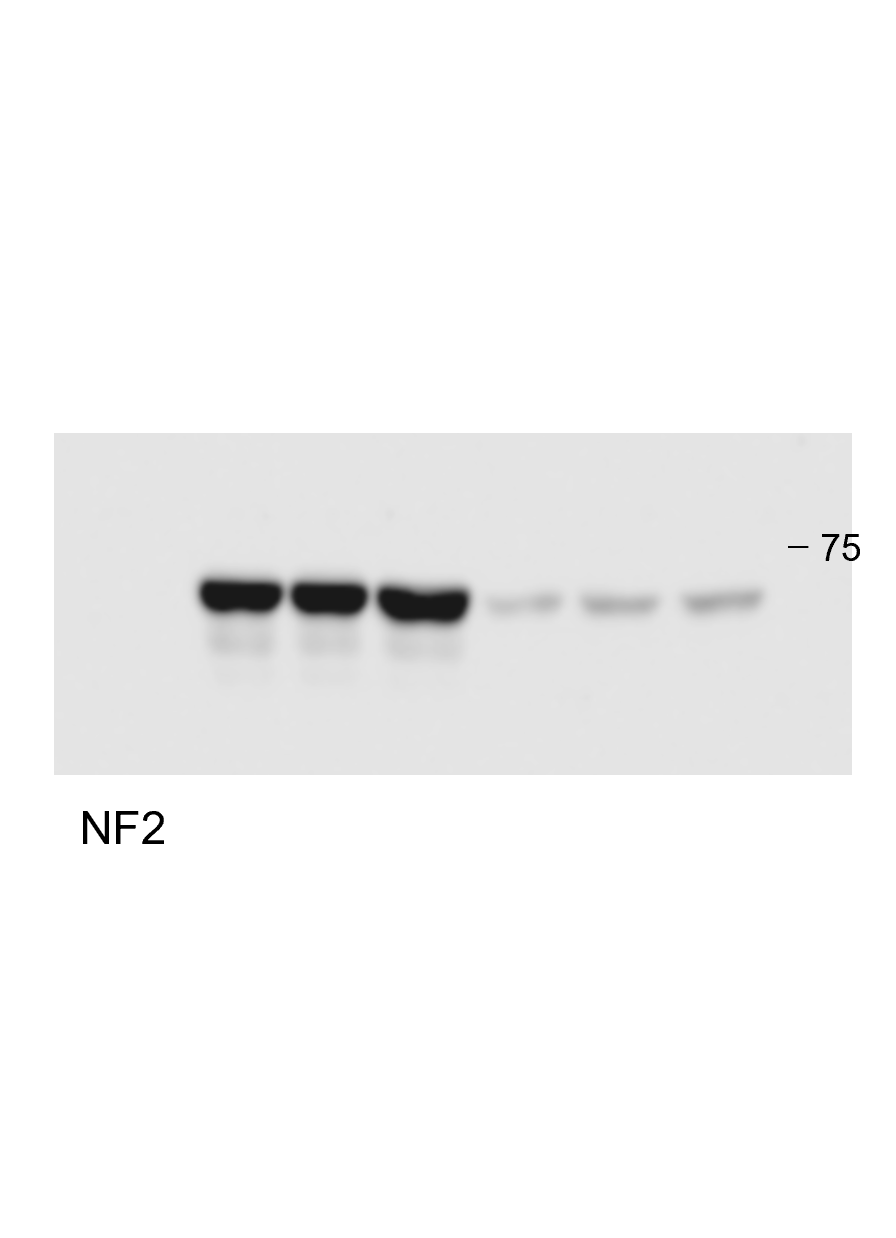

Supplement: Supplementary file 5 — Source data Fig. 3 [file 44319_2024_228_MOESM5_ESM.zip › Figure 3/Figure 3A/NF2.tif]

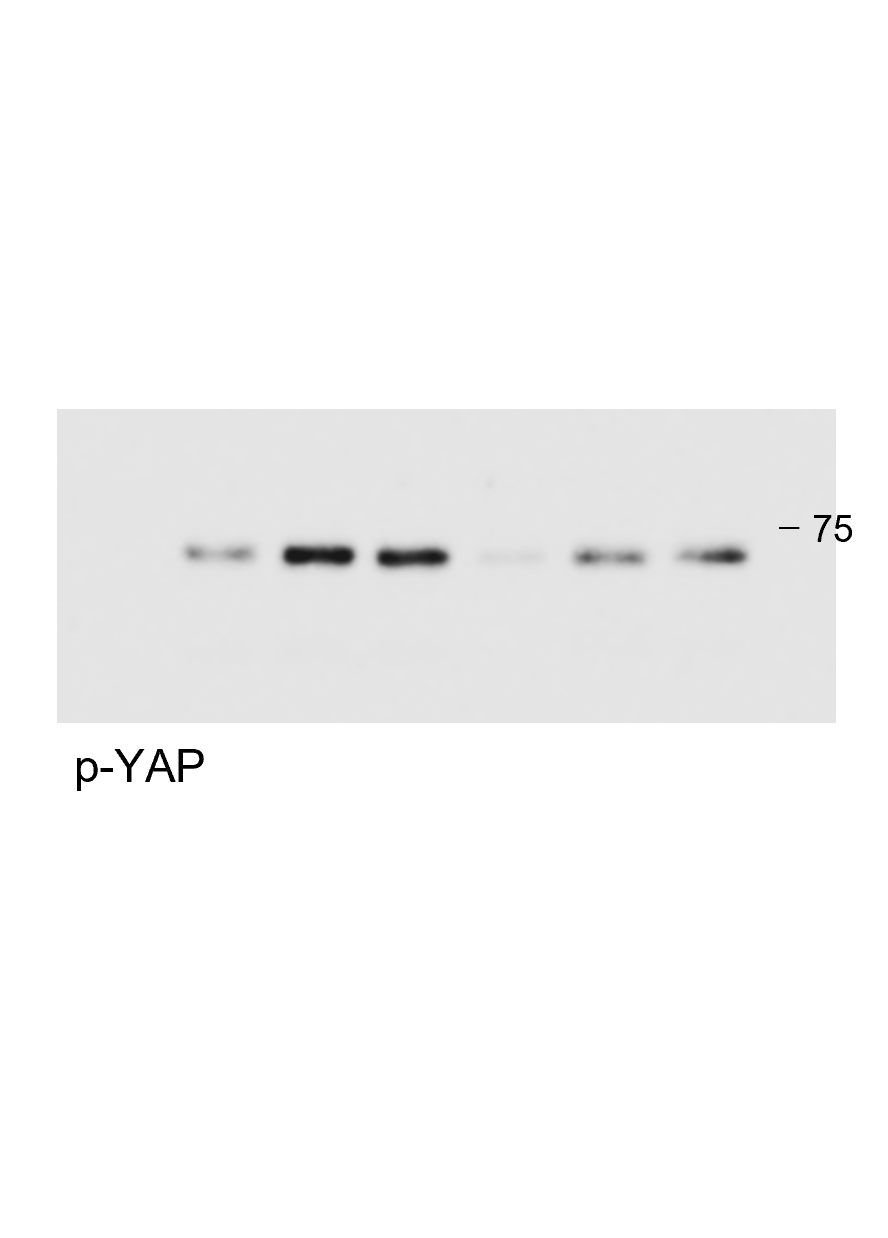

Supplement: Supplementary file 5 — Source data Fig. 3 [file 44319_2024_228_MOESM5_ESM.zip › Figure 3/Figure 3A/p-YAP.tif]

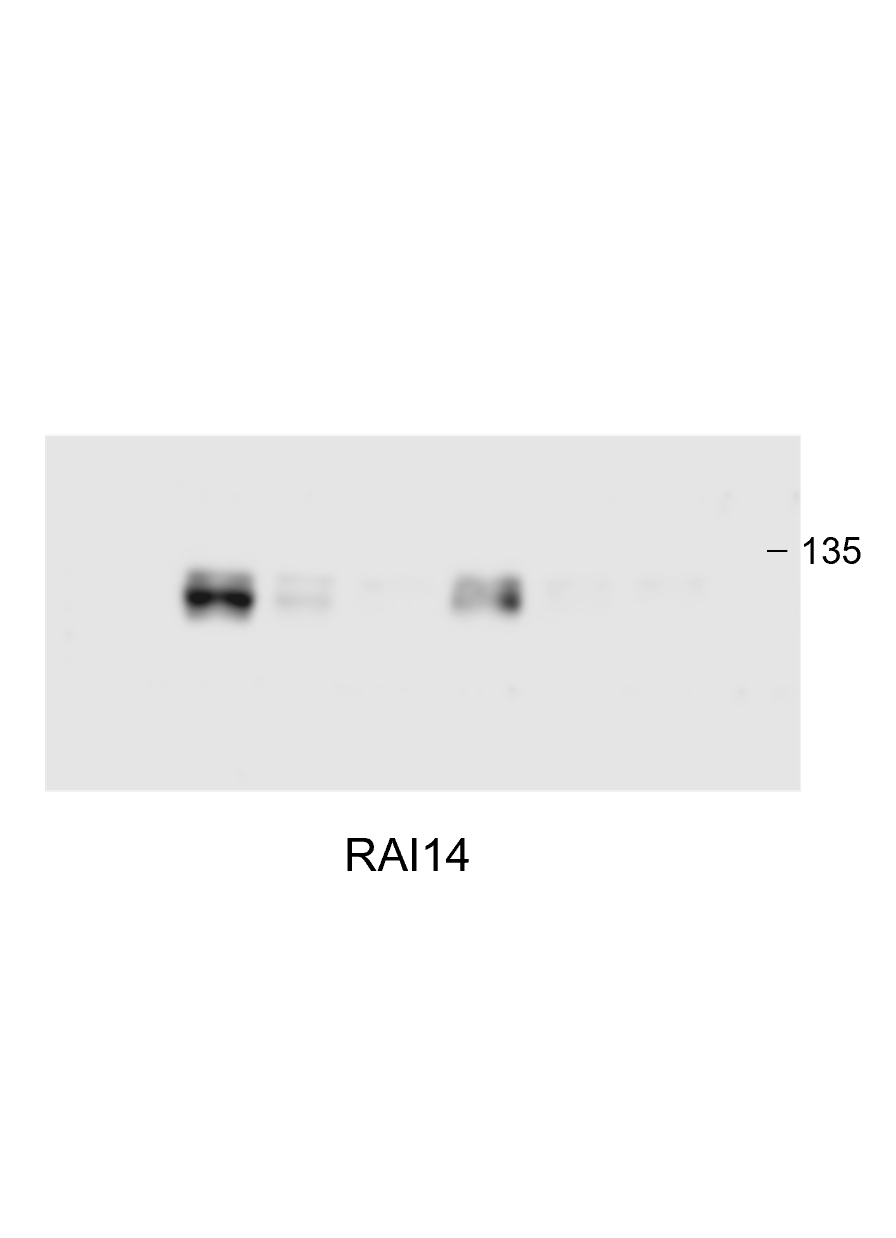

Supplement: Supplementary file 5 — Source data Fig. 3 [file 44319_2024_228_MOESM5_ESM.zip › Figure 3/Figure 3A/RAI14.tif]

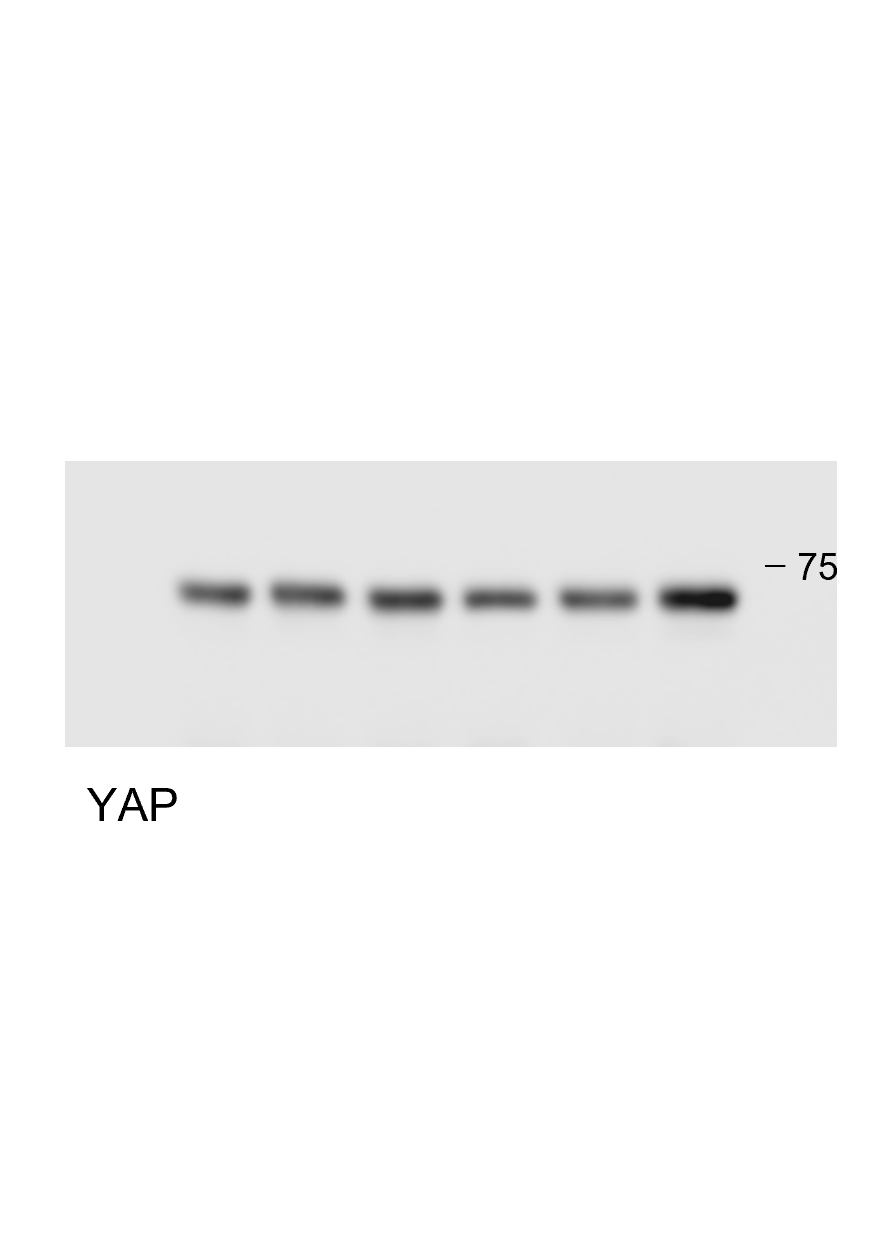

Supplement: Supplementary file 5 — Source data Fig. 3 [file 44319_2024_228_MOESM5_ESM.zip › Figure 3/Figure 3A/YAP.tif]

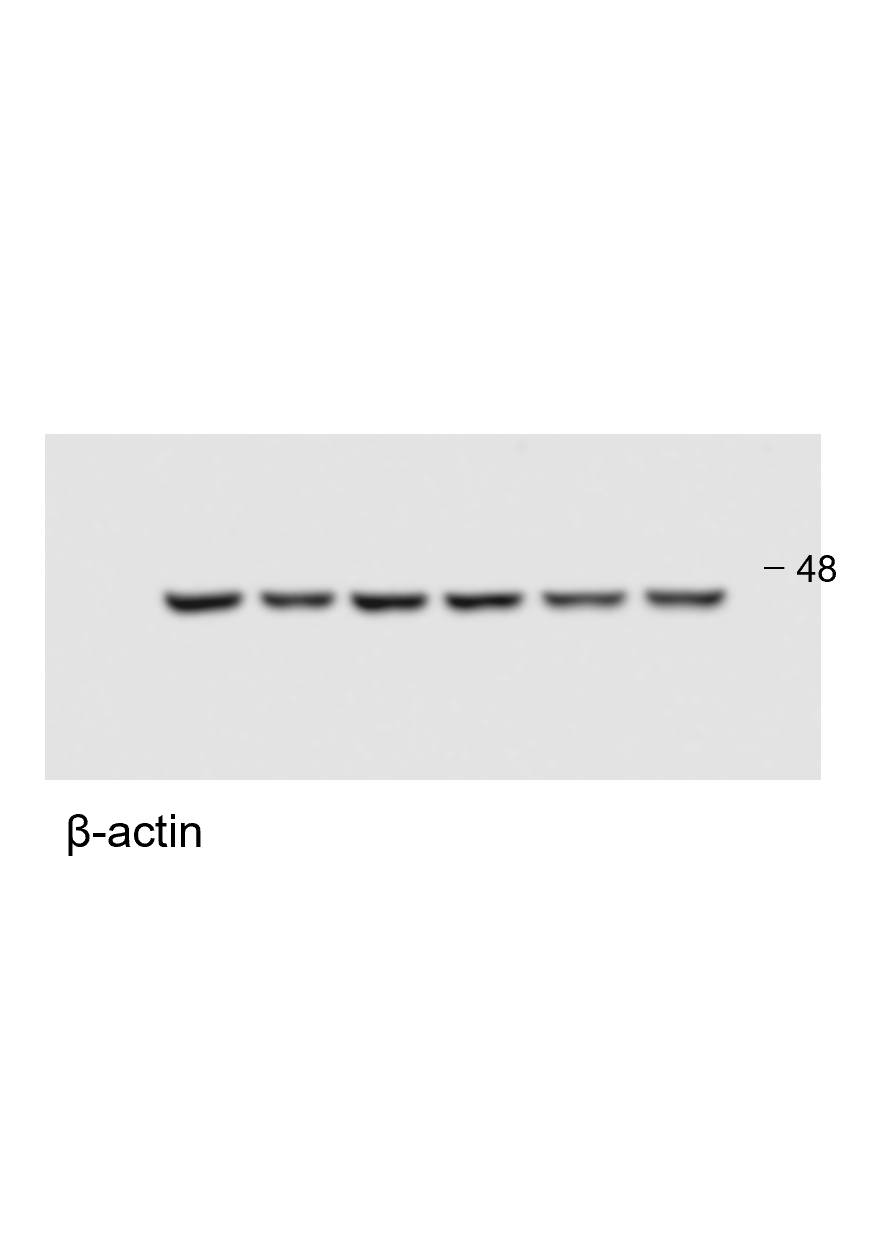

Supplement: Supplementary file 5 — Source data Fig. 3 [file 44319_2024_228_MOESM5_ESM.zip › Figure 3/Figure 3A/ÑΓ-actin.tif]

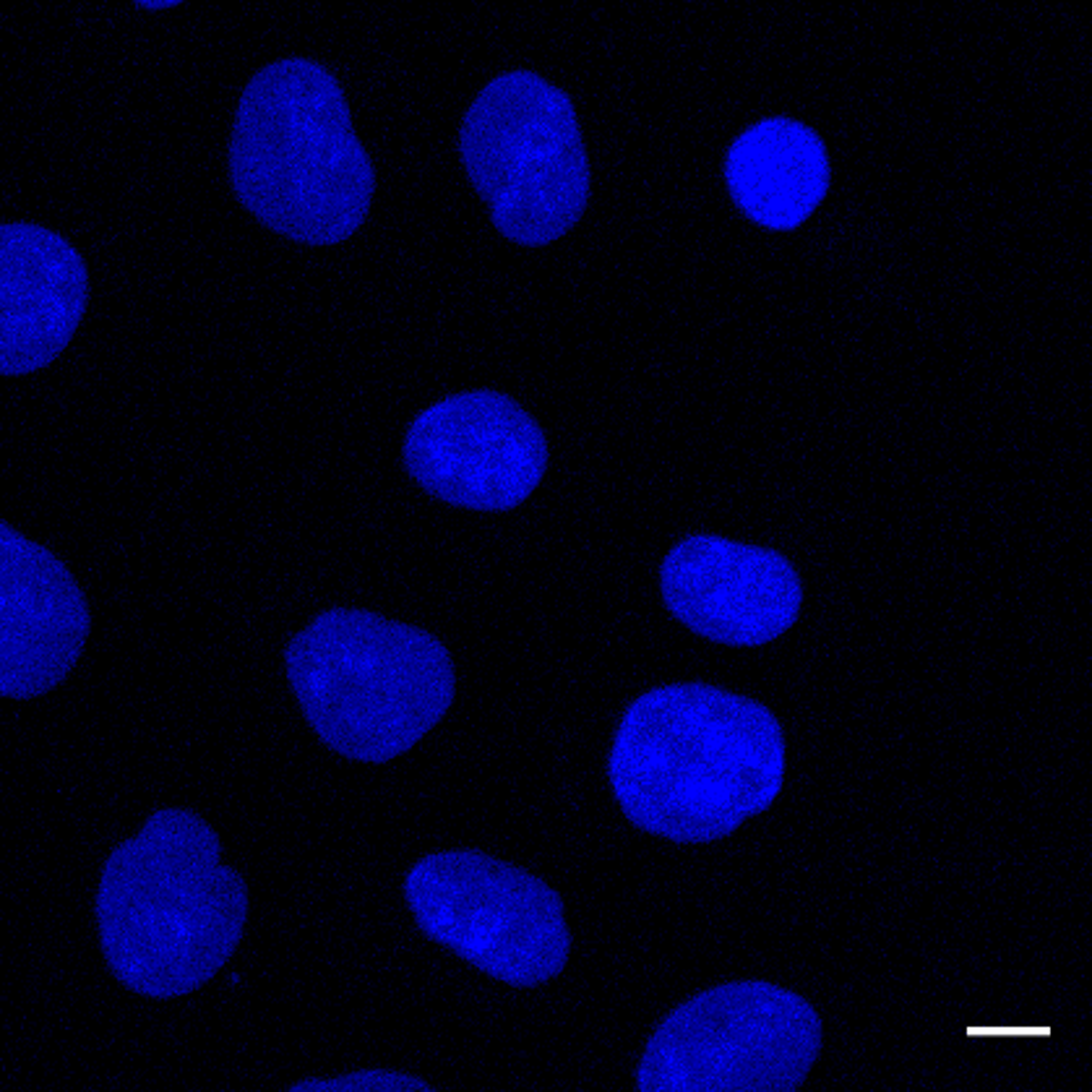

Supplement: Supplementary file 5 — Source data Fig. 3 [file 44319_2024_228_MOESM5_ESM.zip › Figure 3/Figure 3B/DAPI_siCtrl.tif]

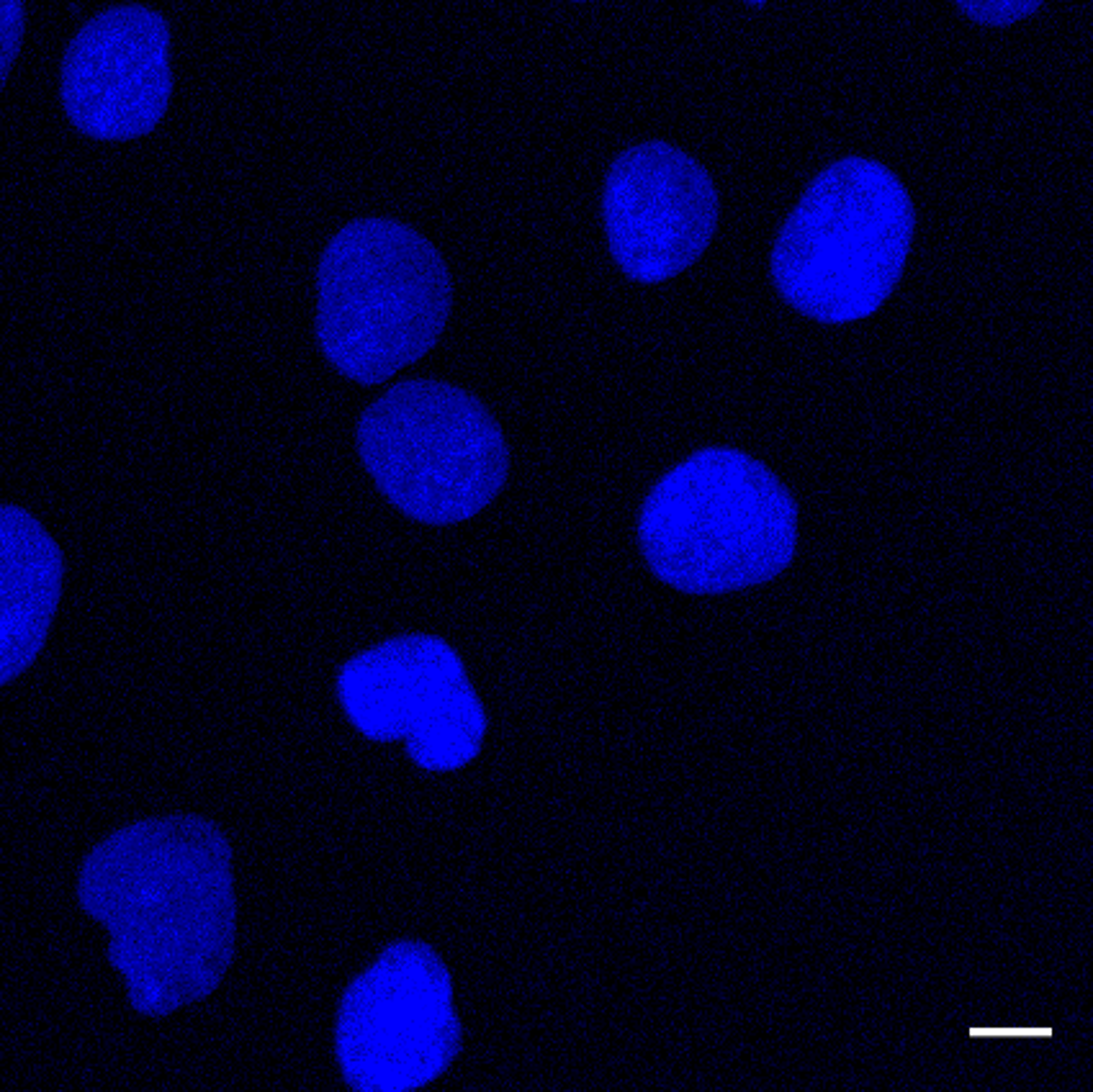

Supplement: Supplementary file 5 — Source data Fig. 3 [file 44319_2024_228_MOESM5_ESM.zip › Figure 3/Figure 3B/DAPI_siNF2&RAI14#1.tif]

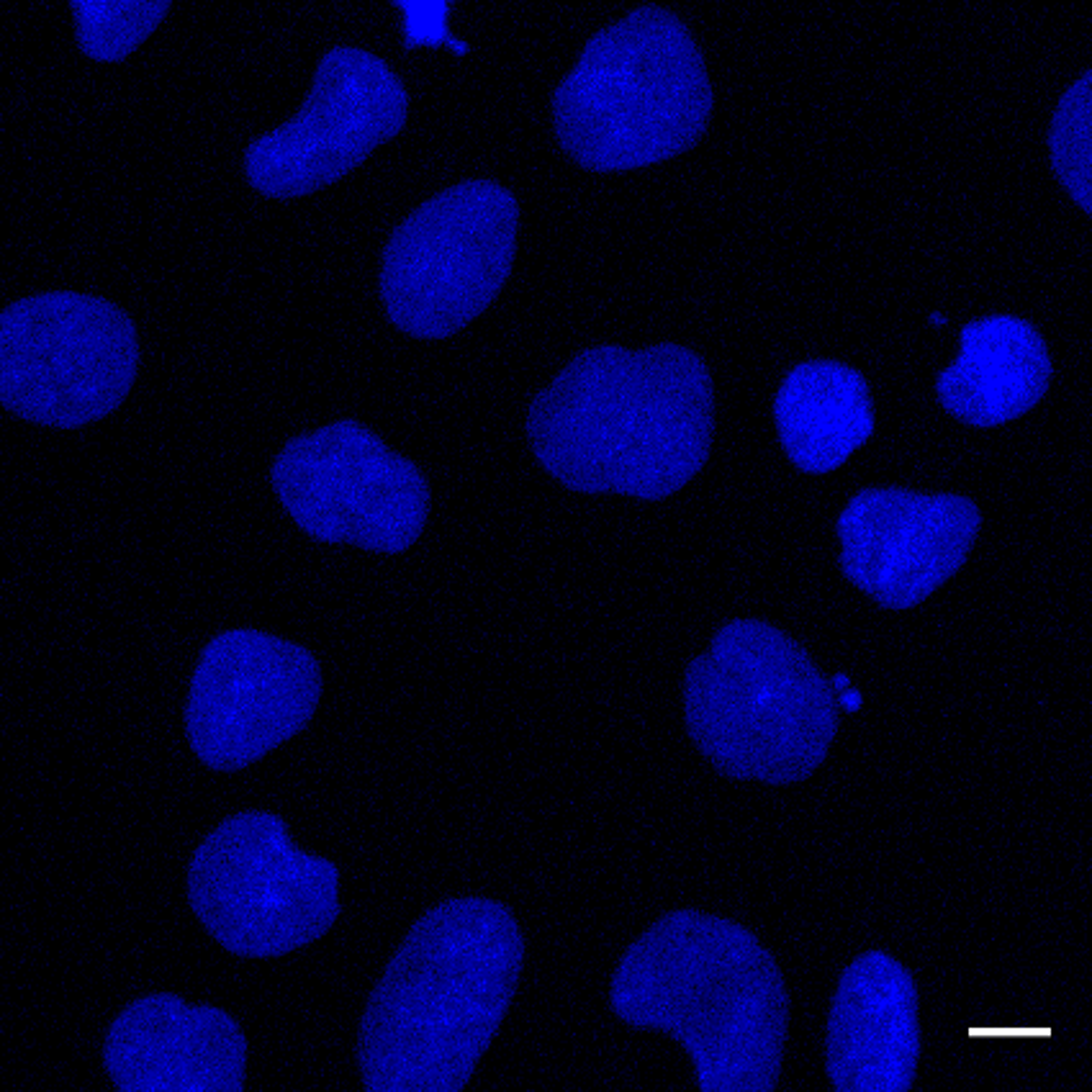

Supplement: Supplementary file 5 — Source data Fig. 3 [file 44319_2024_228_MOESM5_ESM.zip › Figure 3/Figure 3B/DAPI_siNF2&RAI14#2.tif]

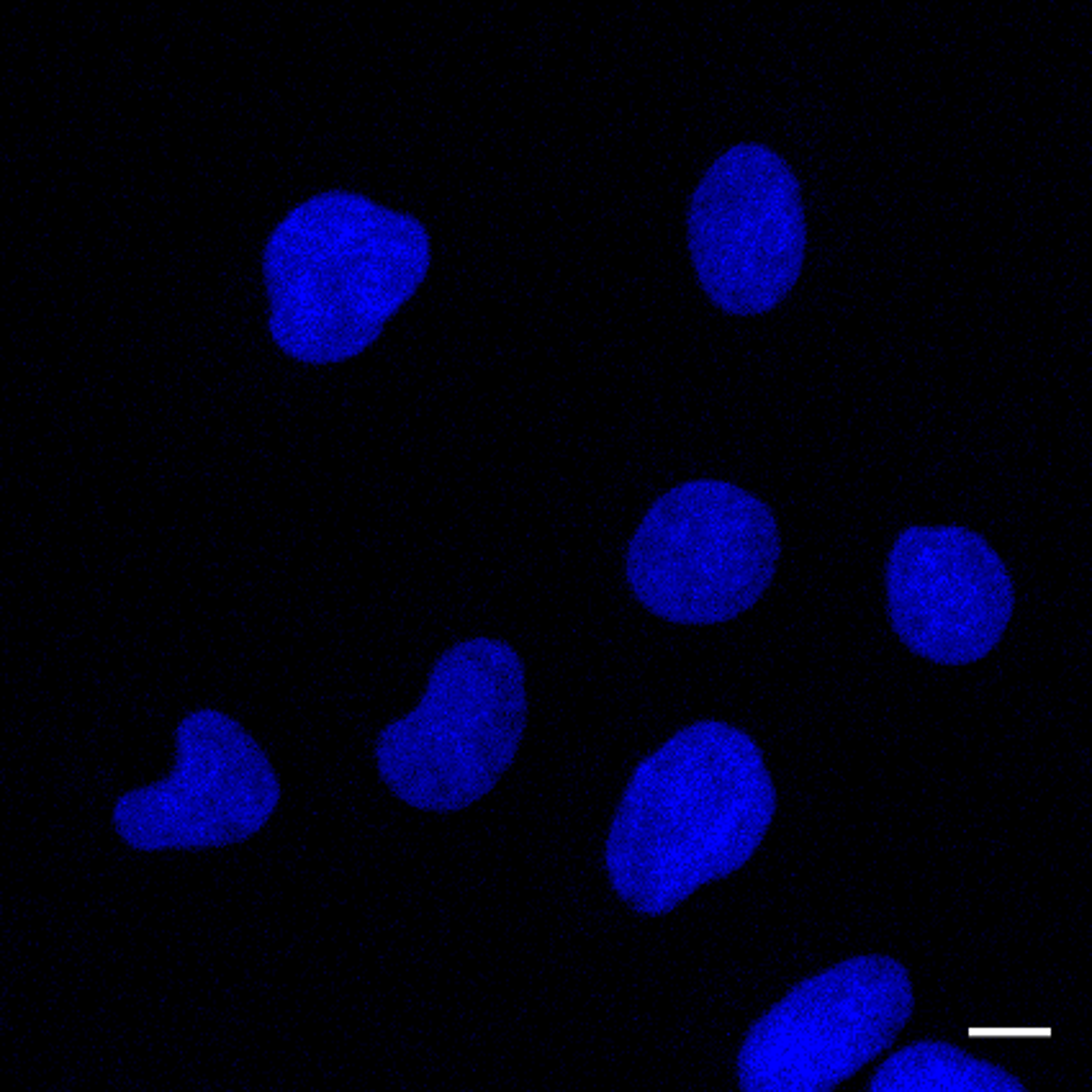

Supplement: Supplementary file 5 — Source data Fig. 3 [file 44319_2024_228_MOESM5_ESM.zip › Figure 3/Figure 3B/DAPI_siNF2.tif]

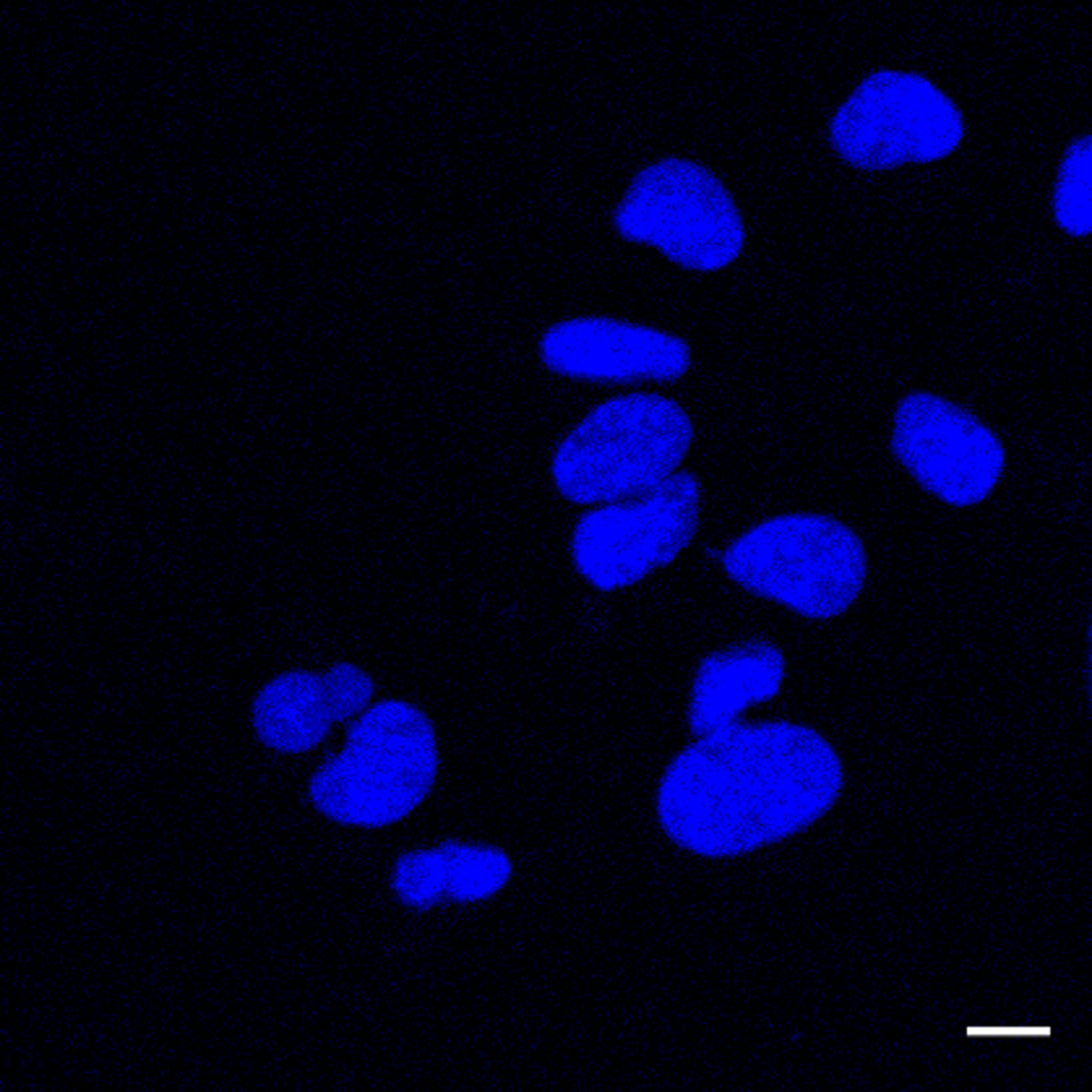

Supplement: Supplementary file 5 — Source data Fig. 3 [file 44319_2024_228_MOESM5_ESM.zip › Figure 3/Figure 3B/DAPI_siRA14#1.tif]

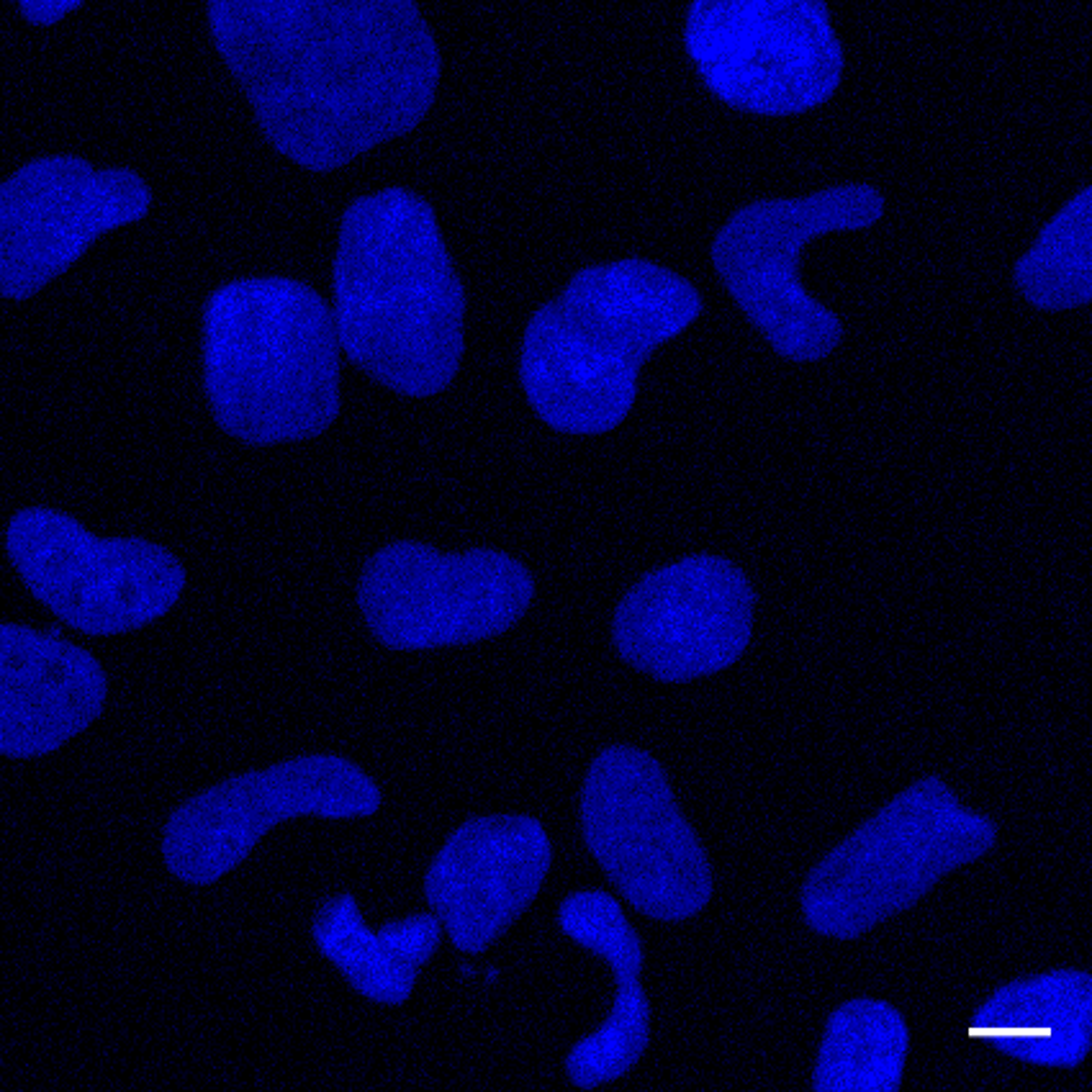

Supplement: Supplementary file 5 — Source data Fig. 3 [file 44319_2024_228_MOESM5_ESM.zip › Figure 3/Figure 3B/DAPI_siRAI14#2.tif]

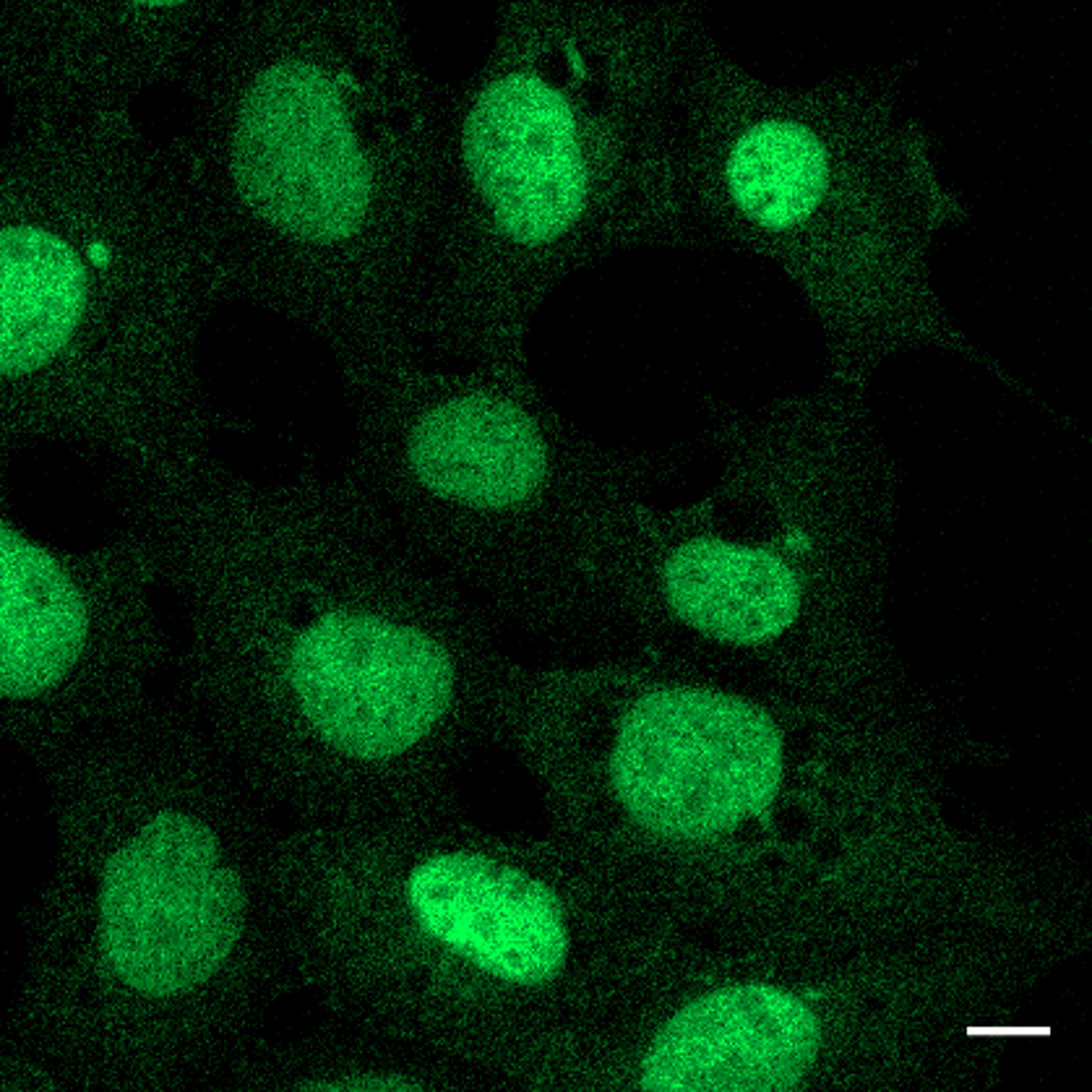

Supplement: Supplementary file 5 — Source data Fig. 3 [file 44319_2024_228_MOESM5_ESM.zip › Figure 3/Figure 3B/YAP_siCtrl.tif]

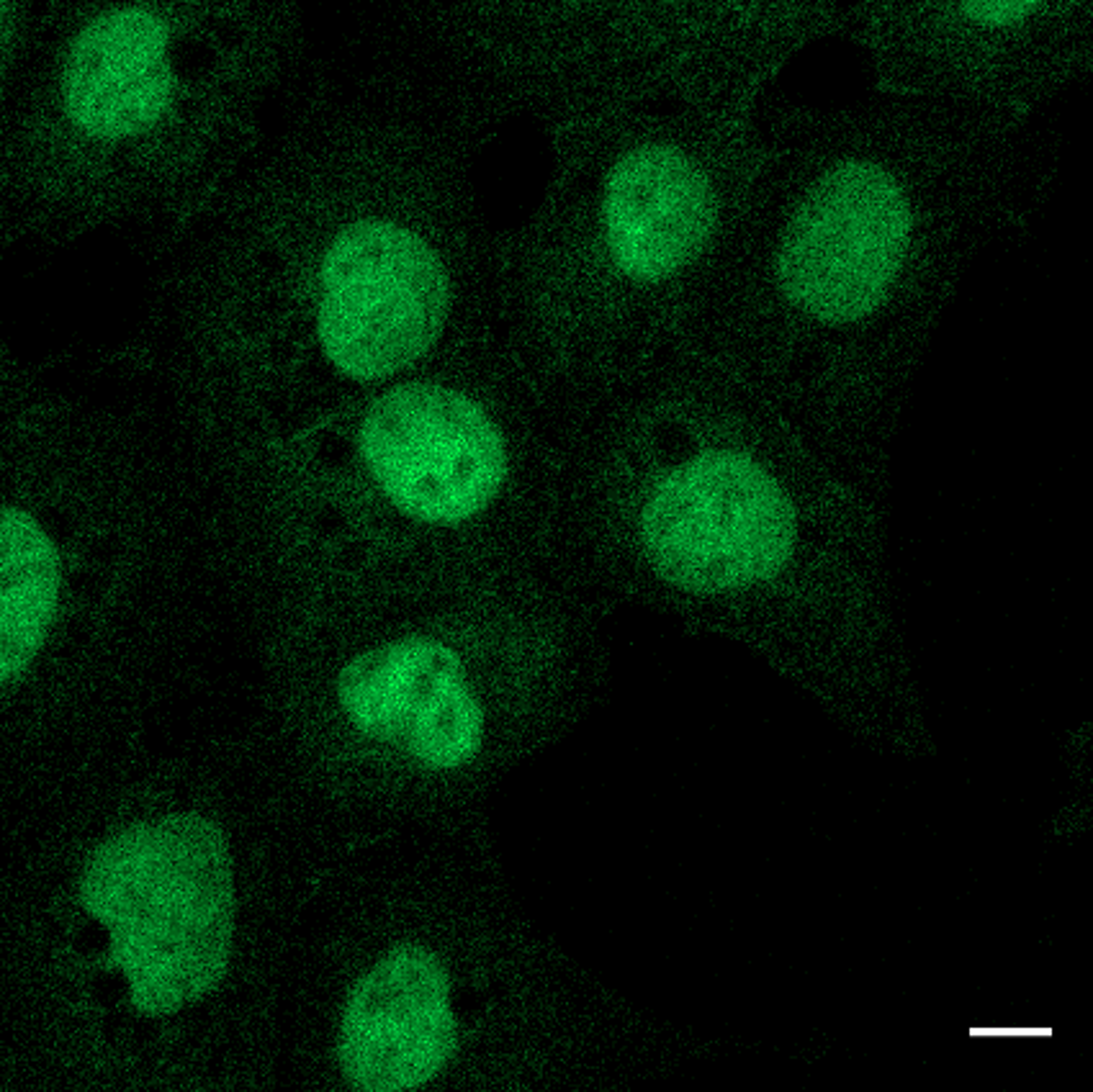

Supplement: Supplementary file 5 — Source data Fig. 3 [file 44319_2024_228_MOESM5_ESM.zip › Figure 3/Figure 3B/YAP_siNF2&RAI14#1.tif]

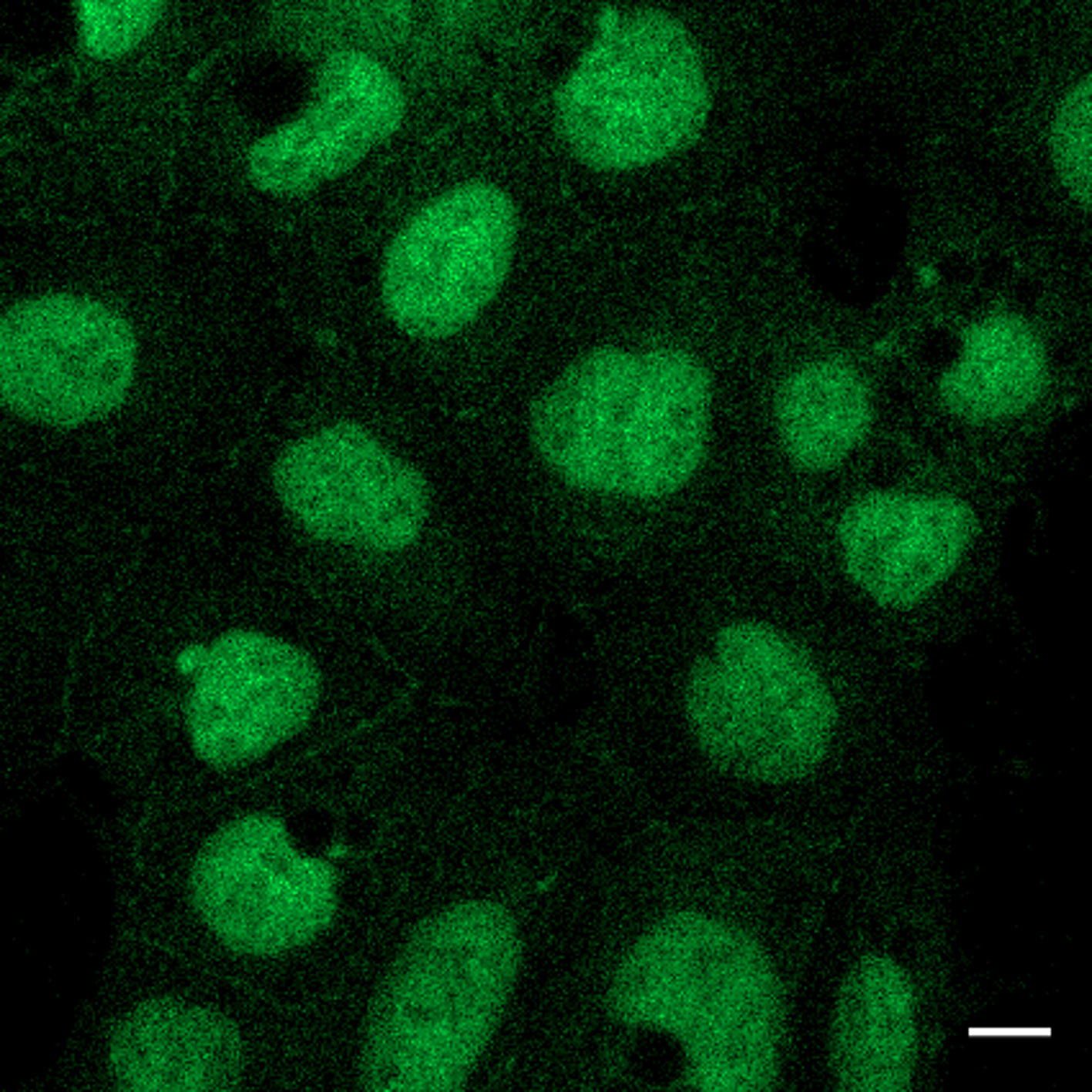

Supplement: Supplementary file 5 — Source data Fig. 3 [file 44319_2024_228_MOESM5_ESM.zip › Figure 3/Figure 3B/YAP_siNF2&RAI14#2.tif]

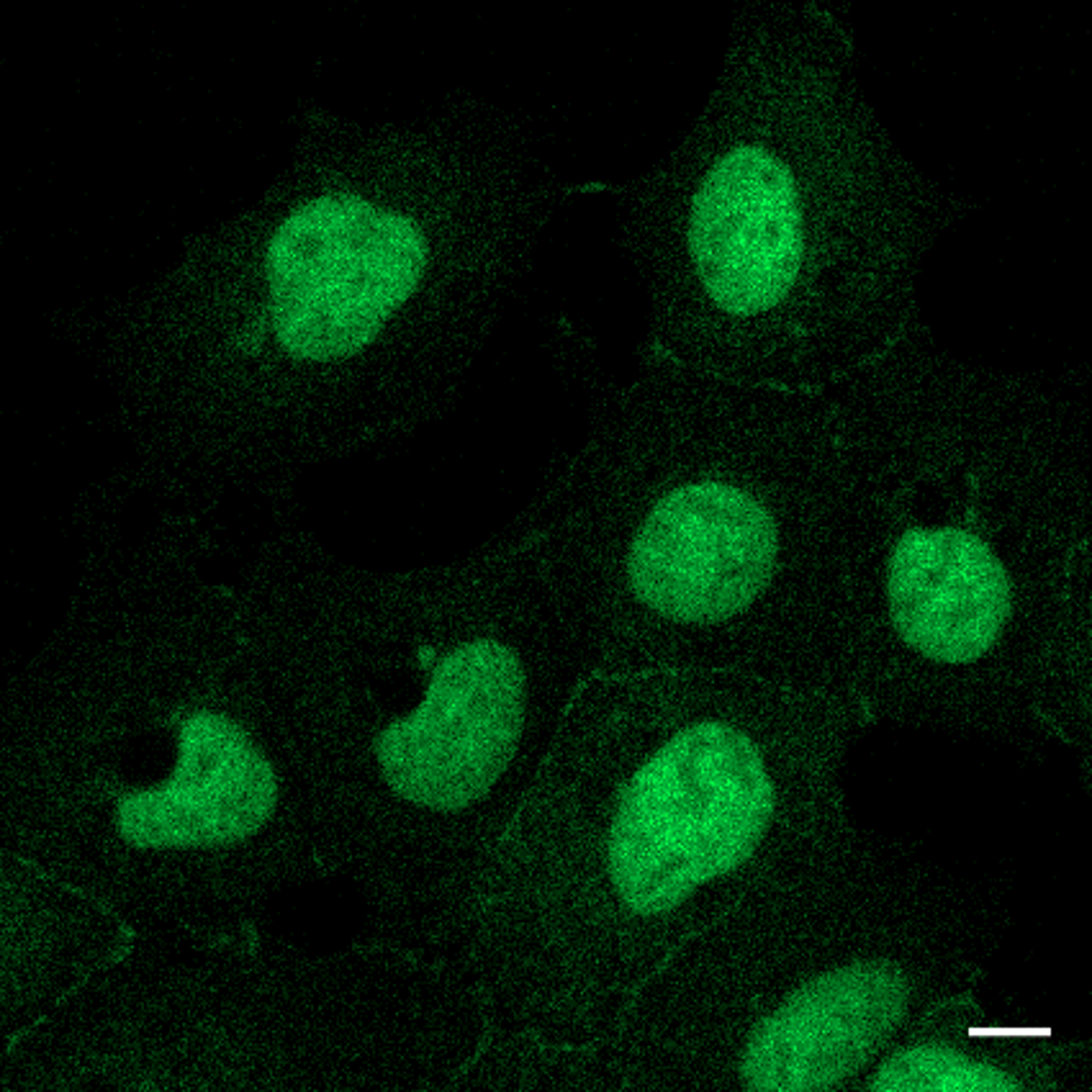

Supplement: Supplementary file 5 — Source data Fig. 3 [file 44319_2024_228_MOESM5_ESM.zip › Figure 3/Figure 3B/YAP_siNF2.tif]

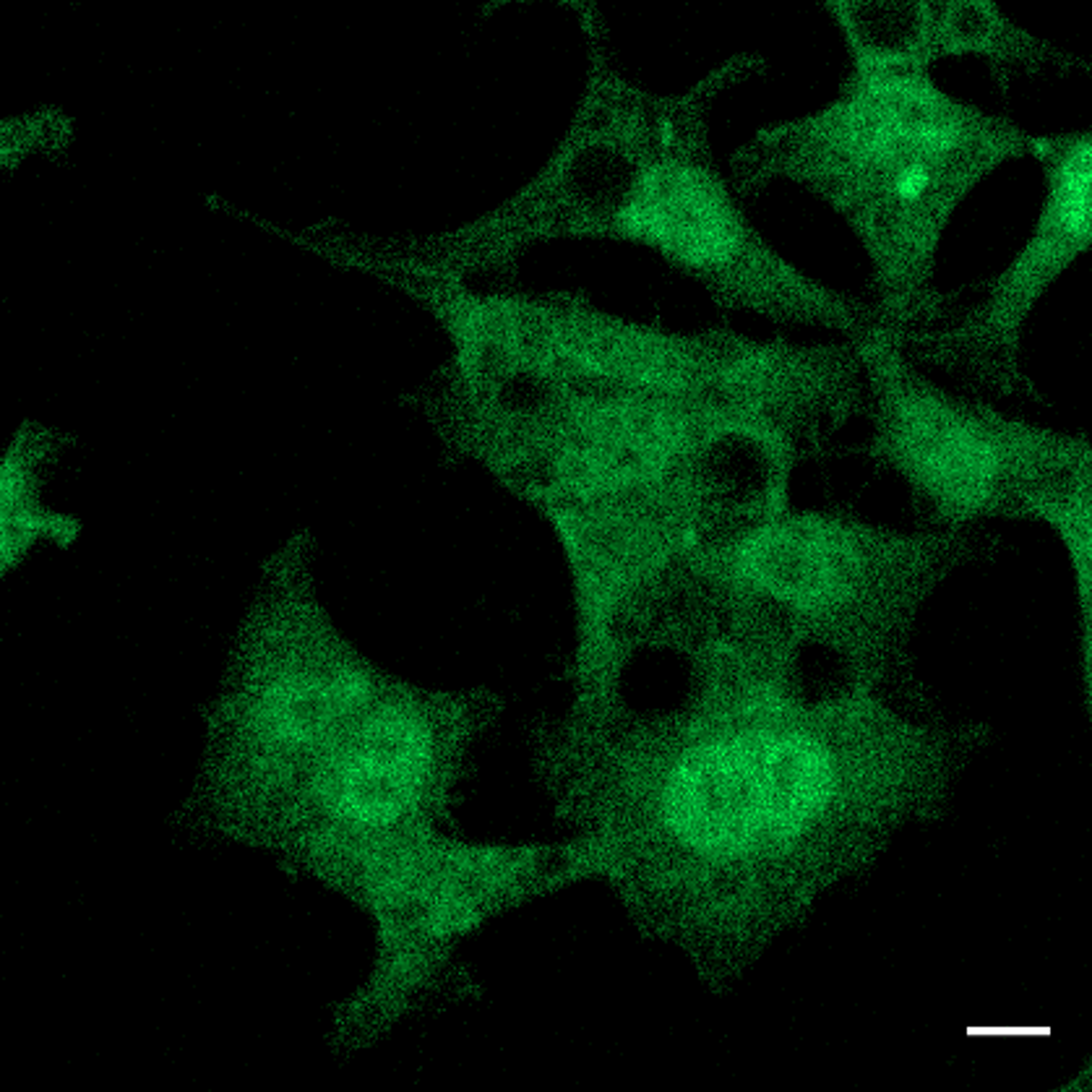

Supplement: Supplementary file 5 — Source data Fig. 3 [file 44319_2024_228_MOESM5_ESM.zip › Figure 3/Figure 3B/YAP_siRAI14#1.tif]

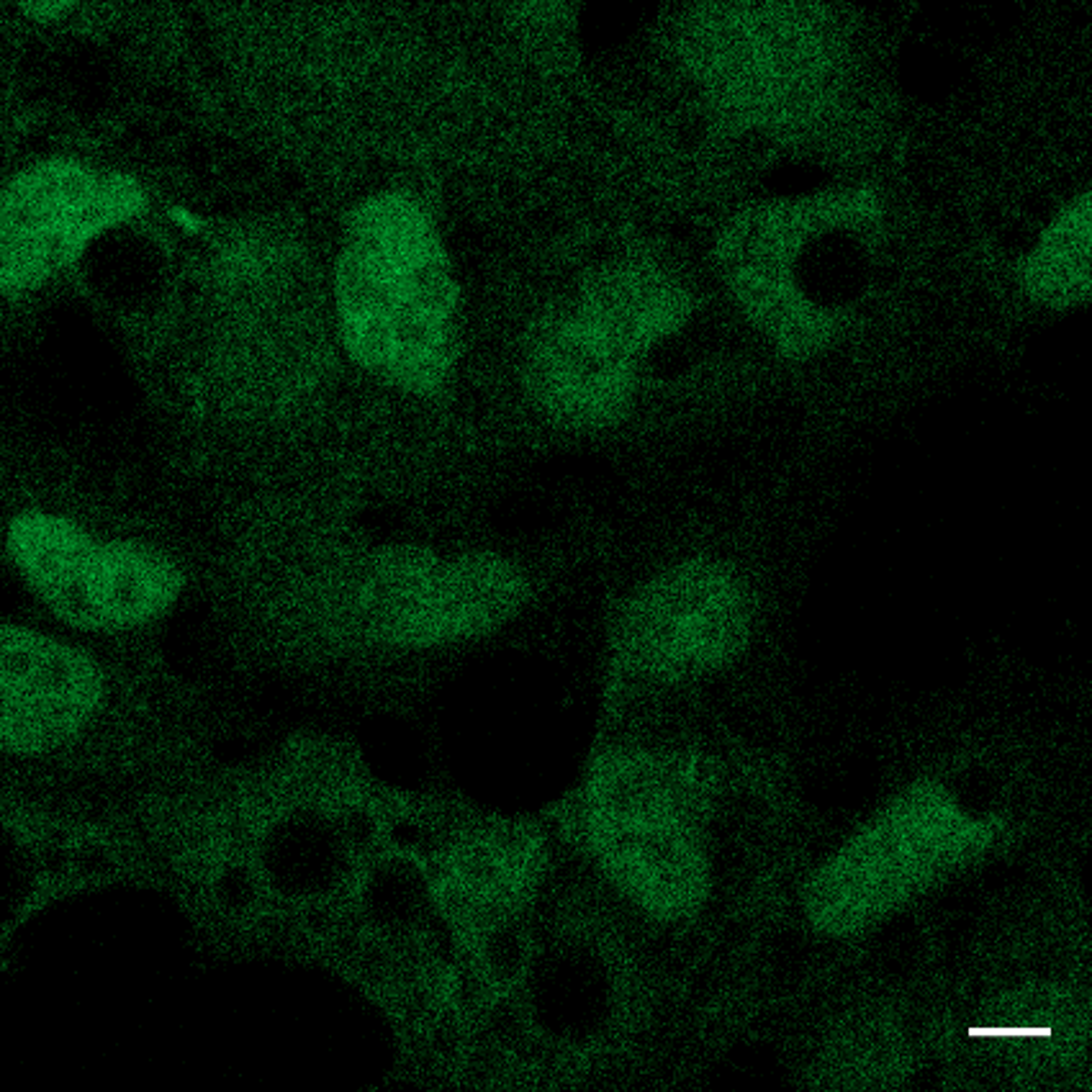

Supplement: Supplementary file 5 — Source data Fig. 3 [file 44319_2024_228_MOESM5_ESM.zip › Figure 3/Figure 3B/YAP_siRAI14#2.tif]

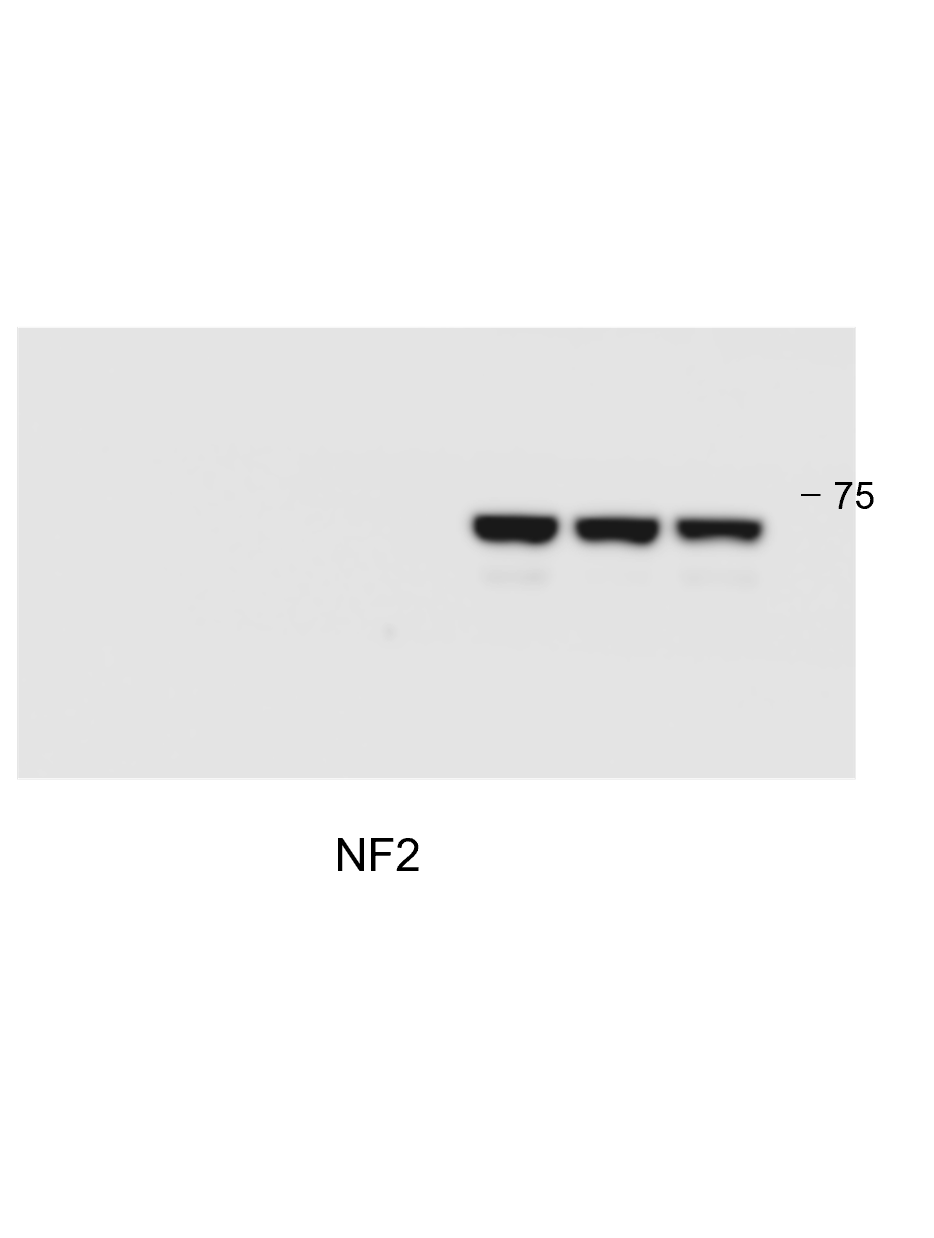

Supplement: Supplementary file 5 — Source data Fig. 3 [file 44319_2024_228_MOESM5_ESM.zip › Figure 3/Figure 3D/NF2.tif]

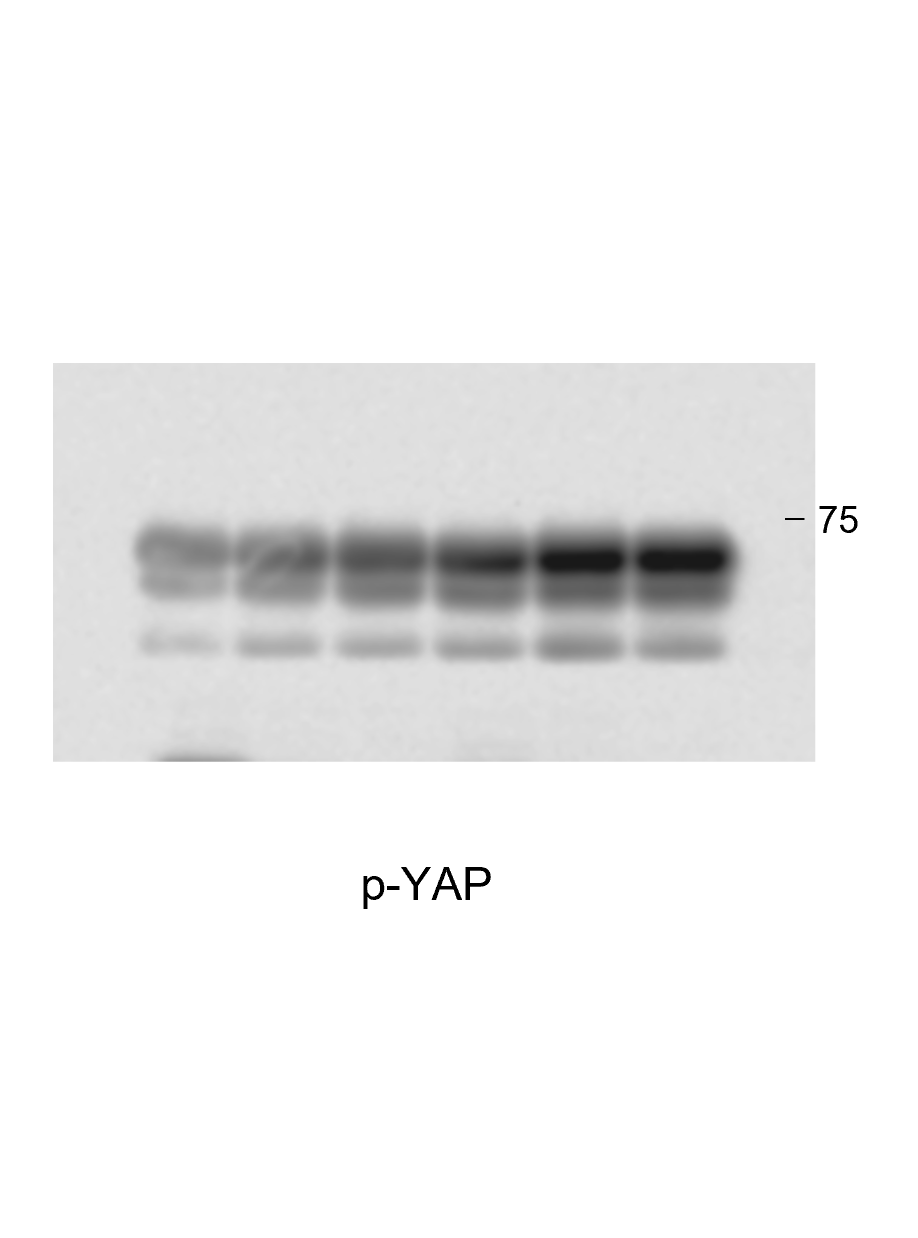

Supplement: Supplementary file 5 — Source data Fig. 3 [file 44319_2024_228_MOESM5_ESM.zip › Figure 3/Figure 3D/p-YAP.tif]

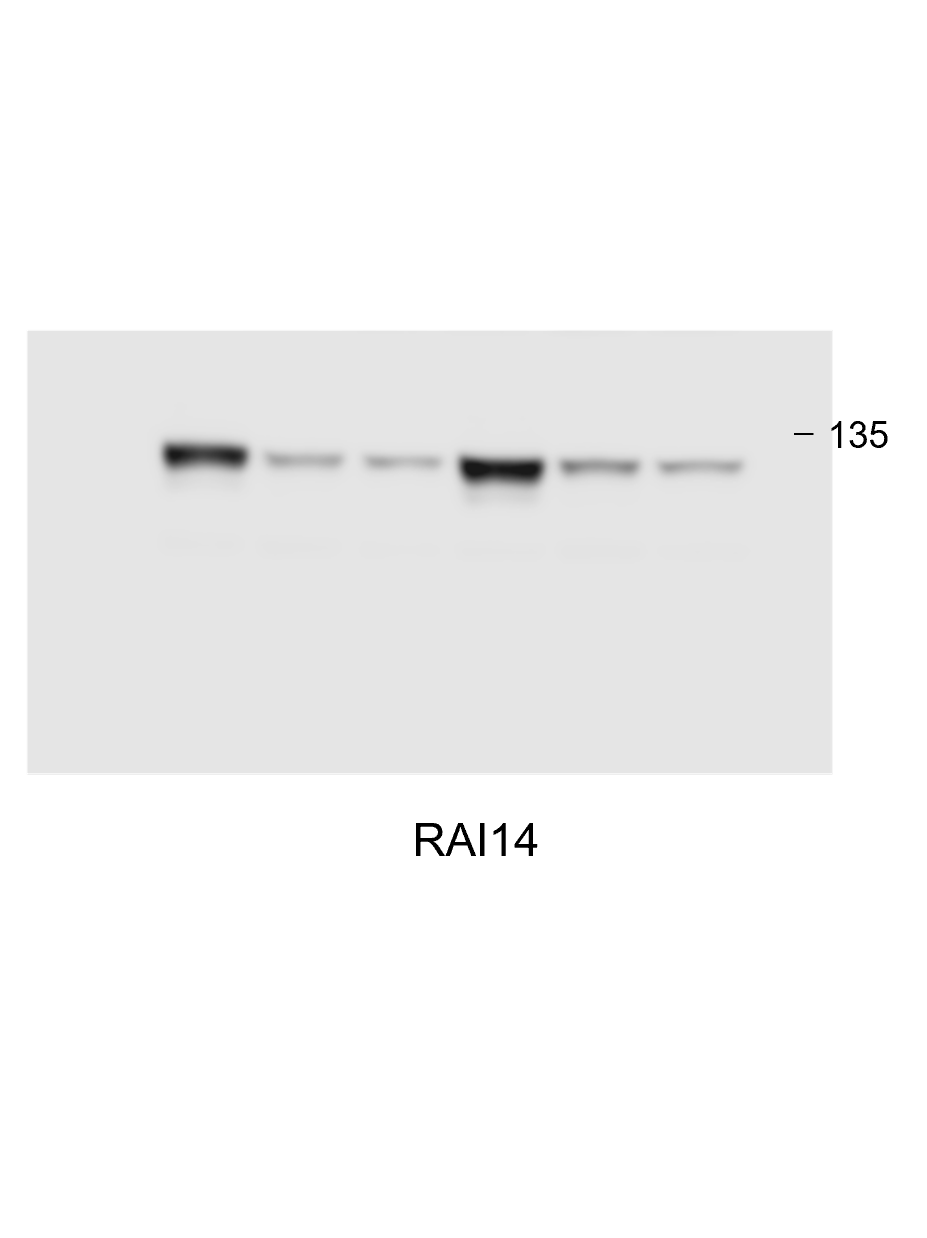

Supplement: Supplementary file 5 — Source data Fig. 3 [file 44319_2024_228_MOESM5_ESM.zip › Figure 3/Figure 3D/RAI14.tif]

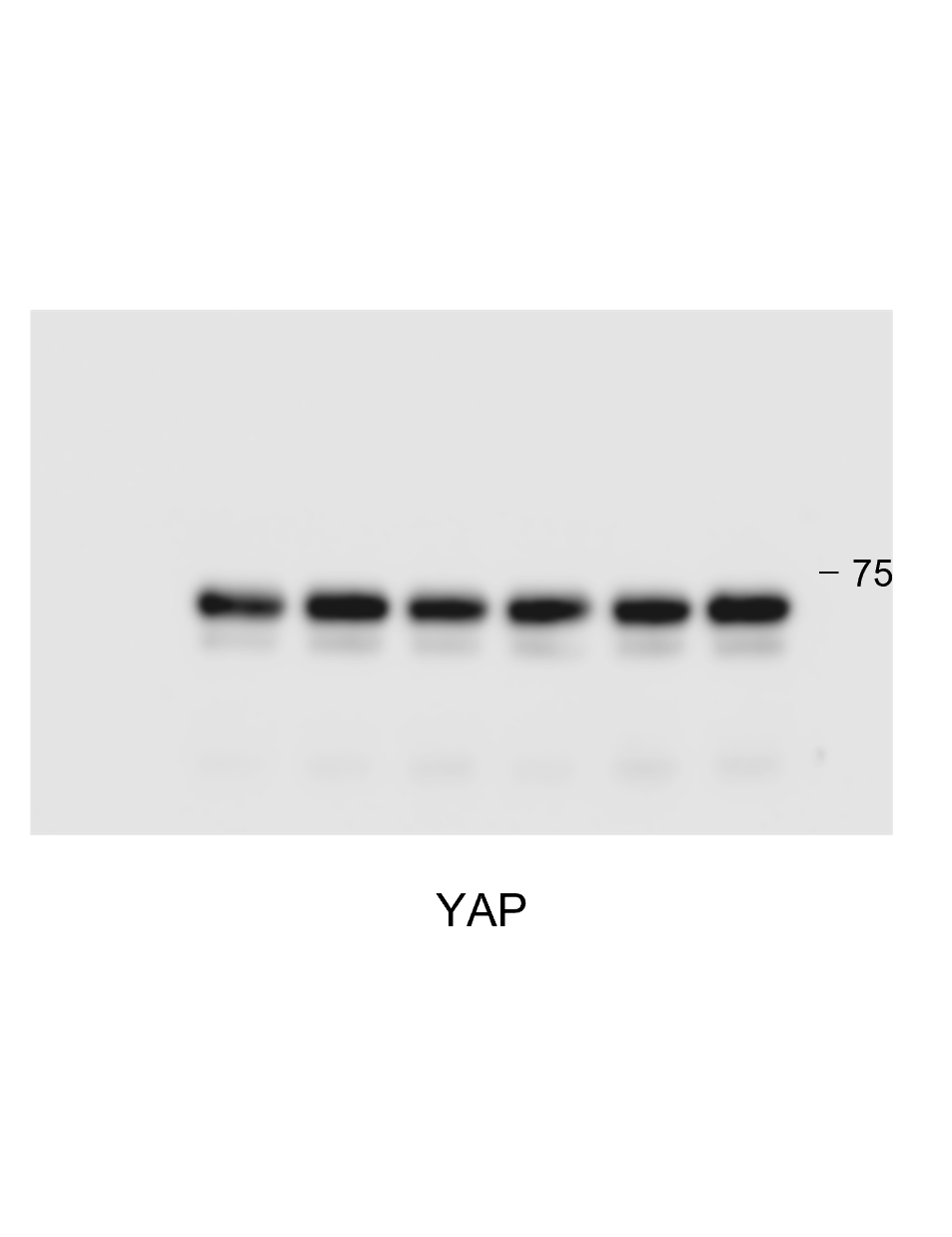

Supplement: Supplementary file 5 — Source data Fig. 3 [file 44319_2024_228_MOESM5_ESM.zip › Figure 3/Figure 3D/YAP.tif]

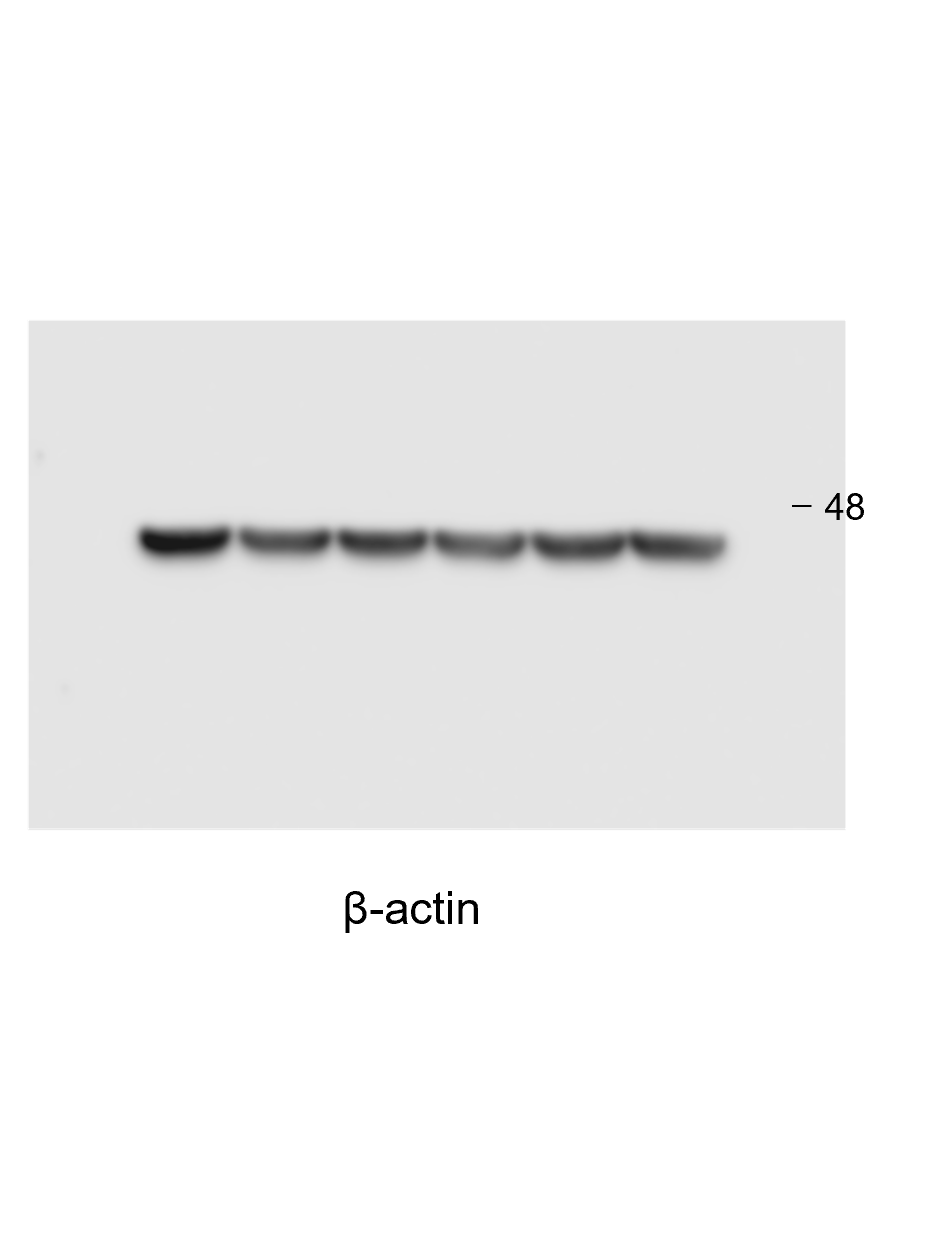

Supplement: Supplementary file 5 — Source data Fig. 3 [file 44319_2024_228_MOESM5_ESM.zip › Figure 3/Figure 3D/ÑΓ-actin.tif]

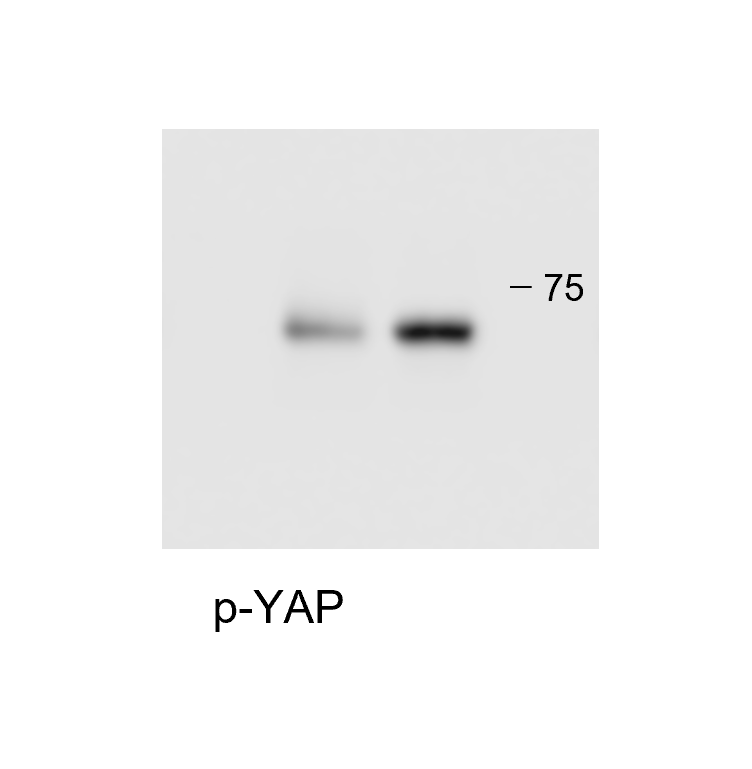

Supplement: Supplementary file 6 — Source data Fig. 4 [file 44319_2024_228_MOESM6_ESM.zip › Figure 4/Figure 4A/p-YAP.tif]

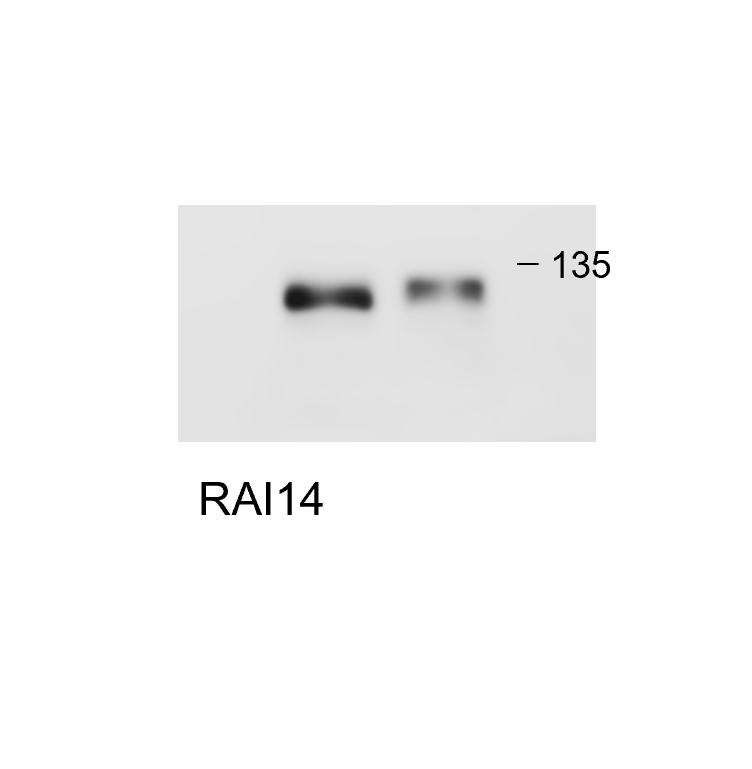

Supplement: Supplementary file 6 — Source data Fig. 4 [file 44319_2024_228_MOESM6_ESM.zip › Figure 4/Figure 4A/RAI14.tif]

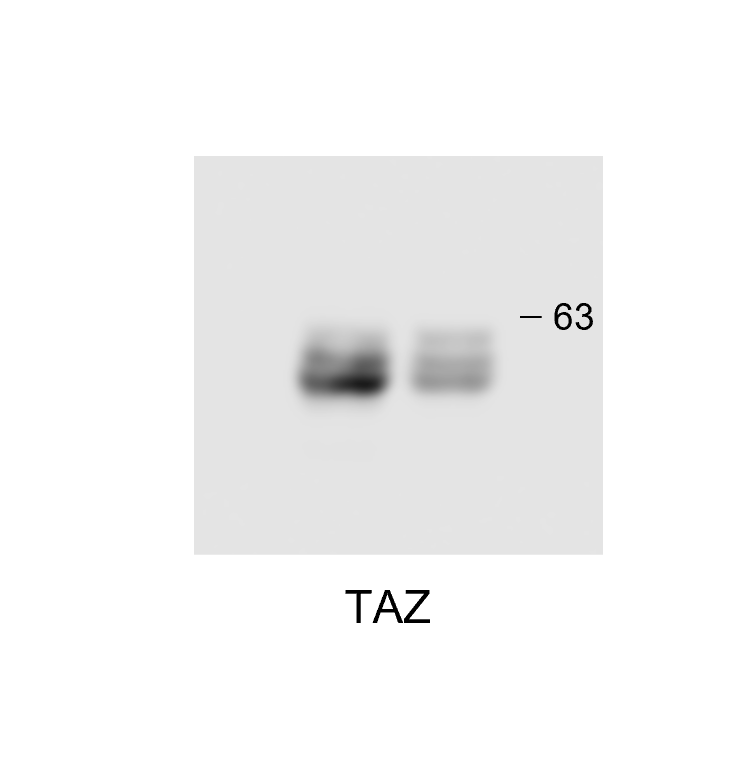

Supplement: Supplementary file 6 — Source data Fig. 4 [file 44319_2024_228_MOESM6_ESM.zip › Figure 4/Figure 4A/TAZ.tif]

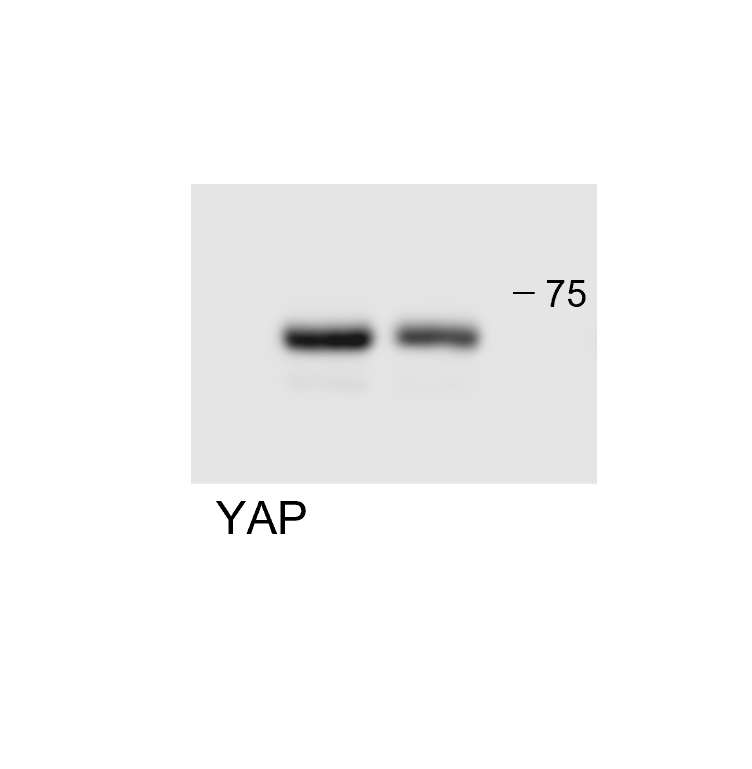

Supplement: Supplementary file 6 — Source data Fig. 4 [file 44319_2024_228_MOESM6_ESM.zip › Figure 4/Figure 4A/YAP.tif]

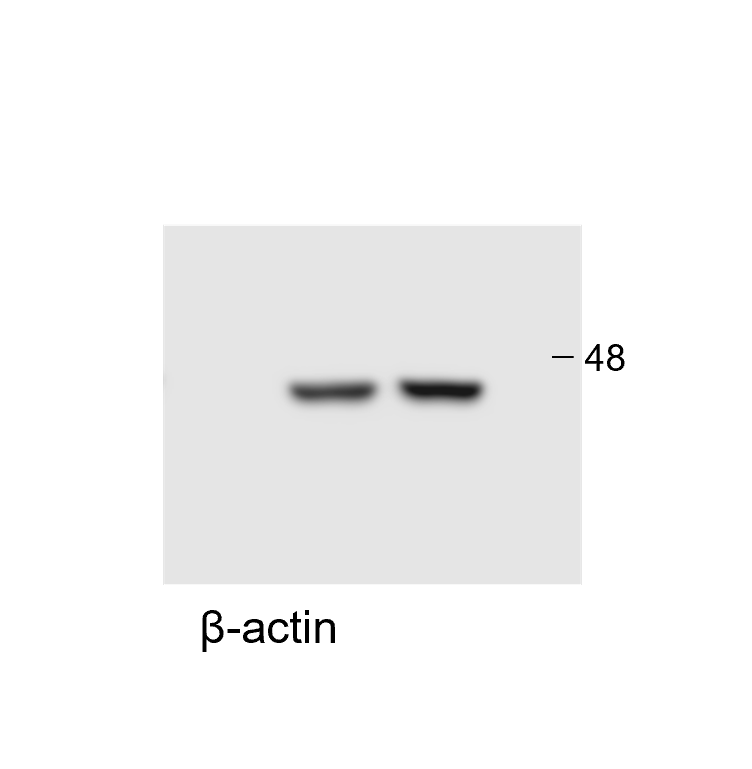

Supplement: Supplementary file 6 — Source data Fig. 4 [file 44319_2024_228_MOESM6_ESM.zip › Figure 4/Figure 4A/ÑΓ-actin.tif]

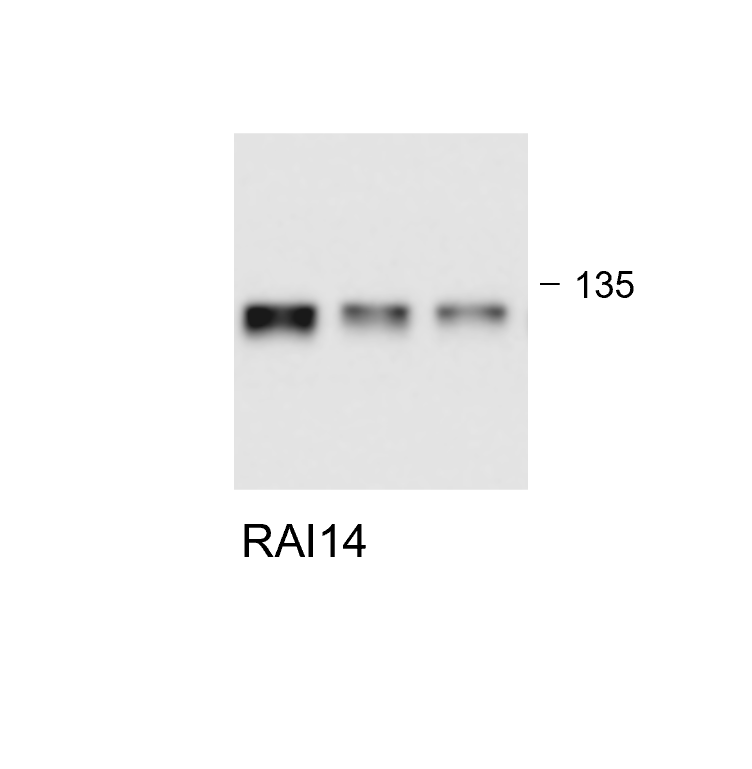

Supplement: Supplementary file 6 — Source data Fig. 4 [file 44319_2024_228_MOESM6_ESM.zip › Figure 4/Figure 4B/RAI14.tif]

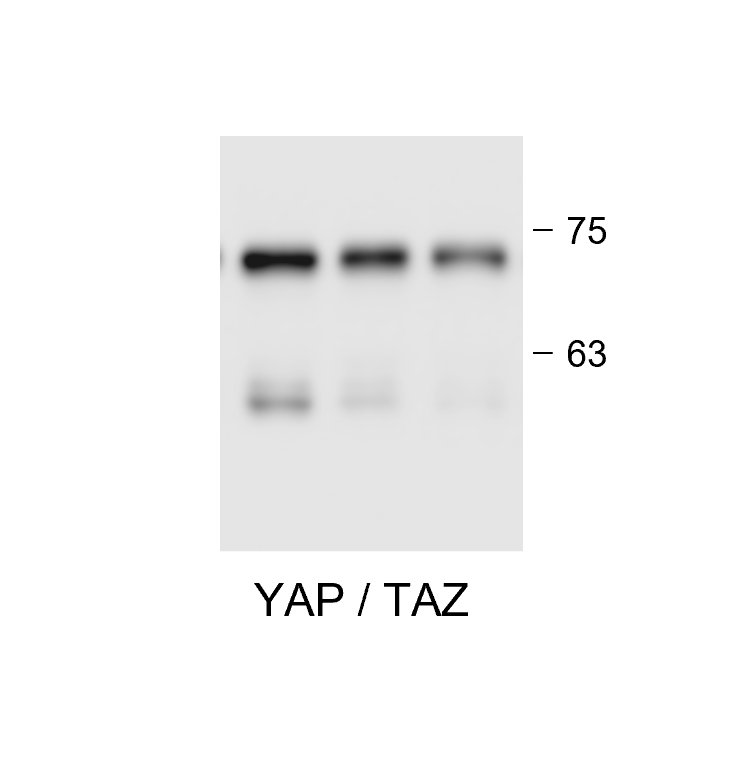

Supplement: Supplementary file 6 — Source data Fig. 4 [file 44319_2024_228_MOESM6_ESM.zip › Figure 4/Figure 4B/YAP&TAZ.tif]

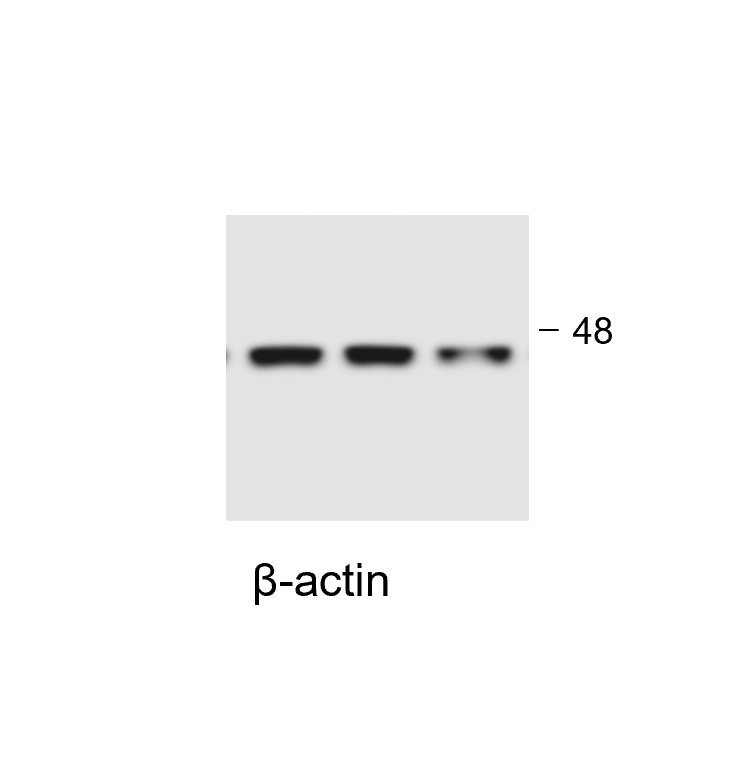

Supplement: Supplementary file 6 — Source data Fig. 4 [file 44319_2024_228_MOESM6_ESM.zip › Figure 4/Figure 4B/ÑΓ-actin.tif]

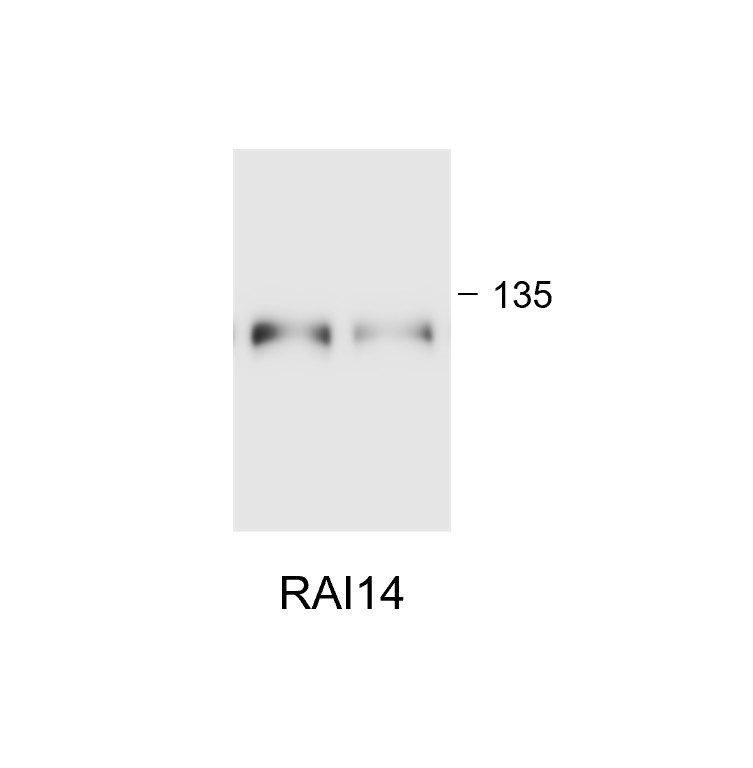

Supplement: Supplementary file 6 — Source data Fig. 4 [file 44319_2024_228_MOESM6_ESM.zip › Figure 4/Figure 4C/RAI14.tif]
