# Supplementary figures and images for: Retinoic acid-induced protein 14 links mechanical forces to Hippo signaling (part 2 of 3)
Source: EMBO Rep. 2024 Aug 19;25(9):18. doi: 10.1038/s44319-024-00228-0 (PMC11387738; doi:10.1038/s44319-024-00228-0)

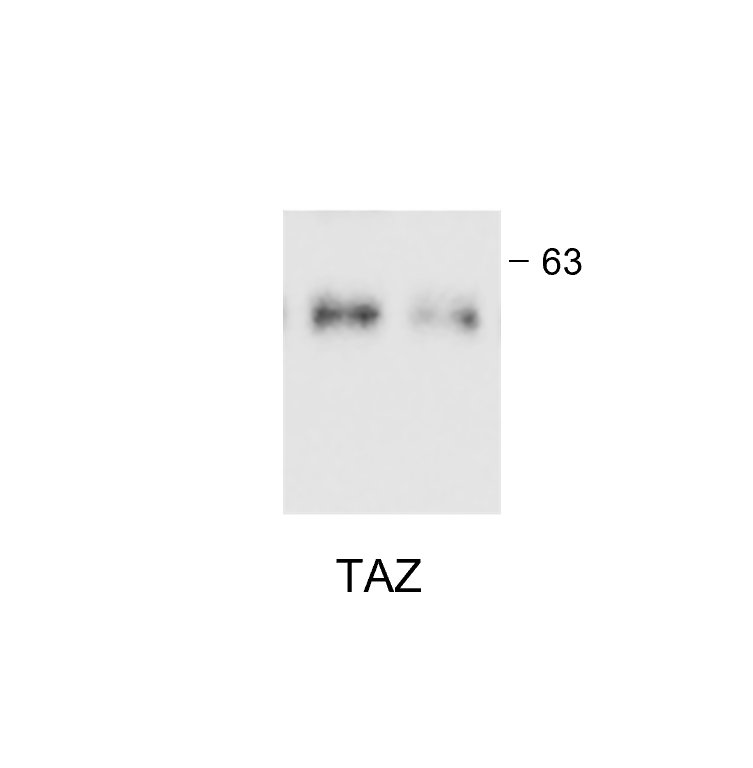

Supplement: Supplementary file 6 — Source data Fig. 4 [file 44319_2024_228_MOESM6_ESM.zip › Figure 4/Figure 4C/TAZ.tif]

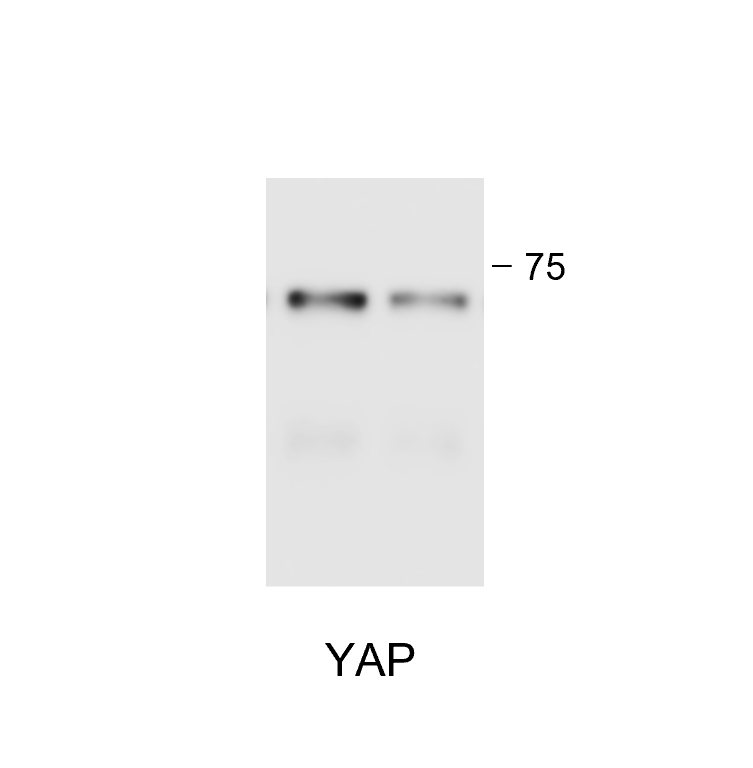

Supplement: Supplementary file 6 — Source data Fig. 4 [file 44319_2024_228_MOESM6_ESM.zip › Figure 4/Figure 4C/YAP.tif]

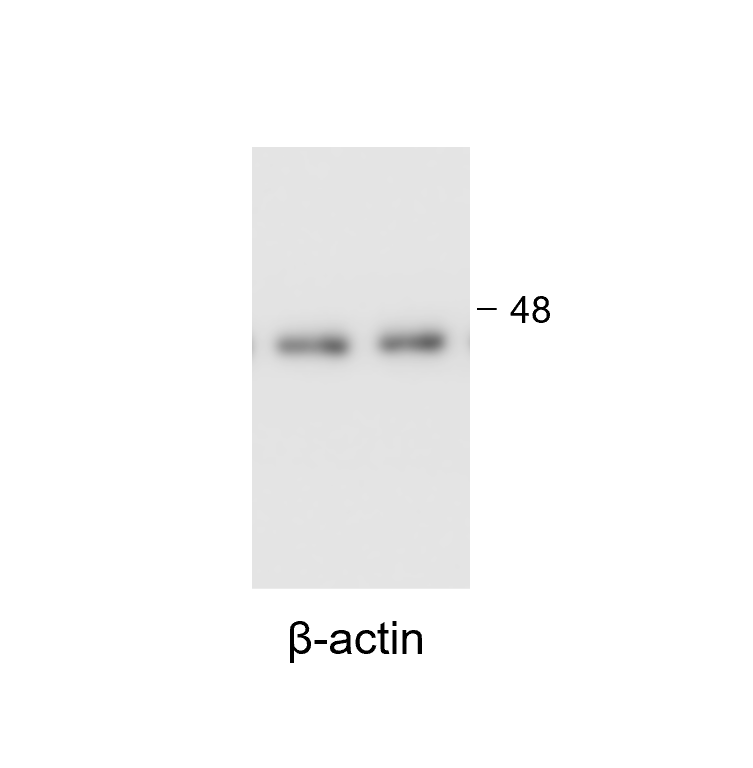

Supplement: Supplementary file 6 — Source data Fig. 4 [file 44319_2024_228_MOESM6_ESM.zip › Figure 4/Figure 4C/ÑΓ-actin.tif]

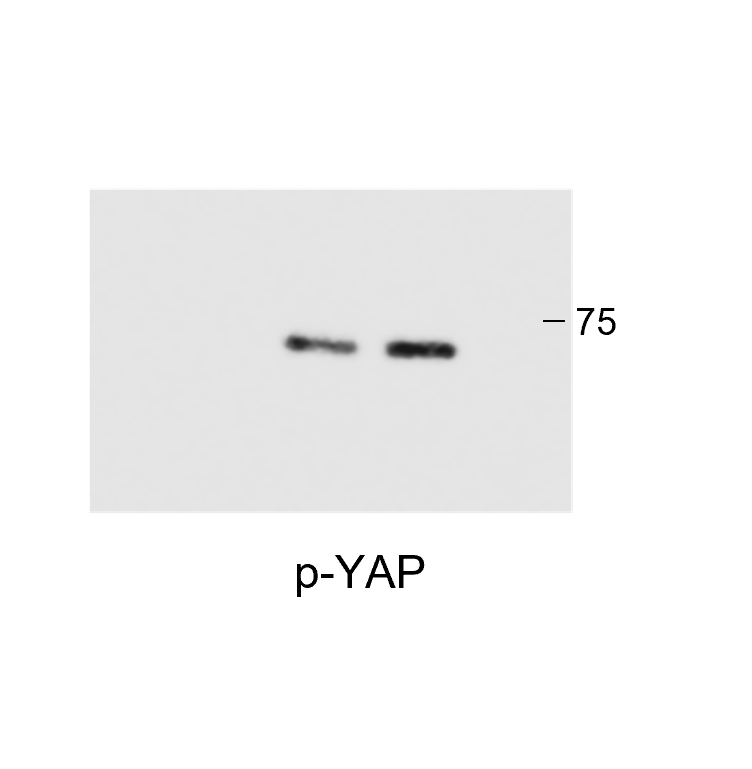

Supplement: Supplementary file 6 — Source data Fig. 4 [file 44319_2024_228_MOESM6_ESM.zip › Figure 4/Figure 4D/p-YAP.tif]

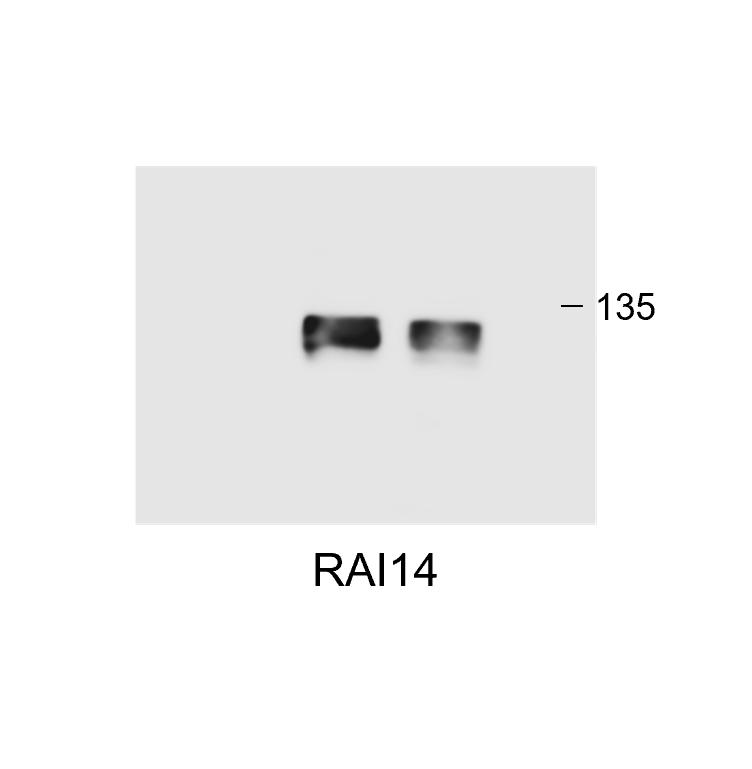

Supplement: Supplementary file 6 — Source data Fig. 4 [file 44319_2024_228_MOESM6_ESM.zip › Figure 4/Figure 4D/RAI14.tif]

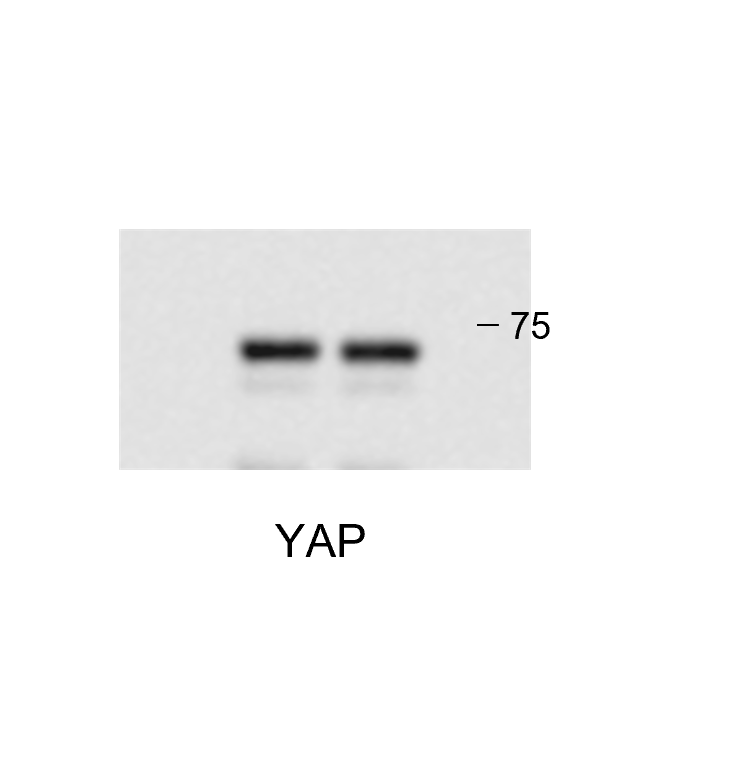

Supplement: Supplementary file 6 — Source data Fig. 4 [file 44319_2024_228_MOESM6_ESM.zip › Figure 4/Figure 4D/YAP.tif]

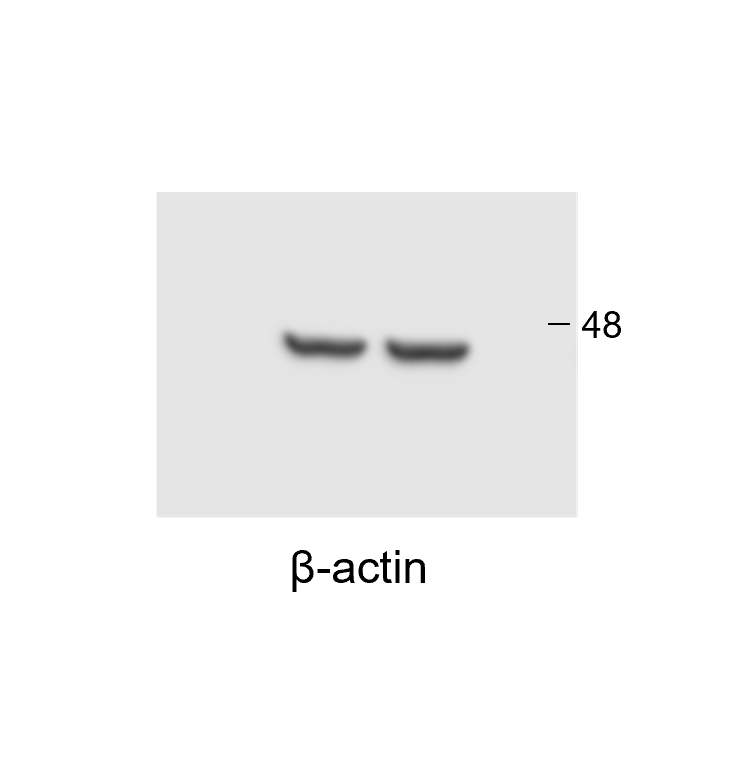

Supplement: Supplementary file 6 — Source data Fig. 4 [file 44319_2024_228_MOESM6_ESM.zip › Figure 4/Figure 4D/ÑΓ-actin.tif]

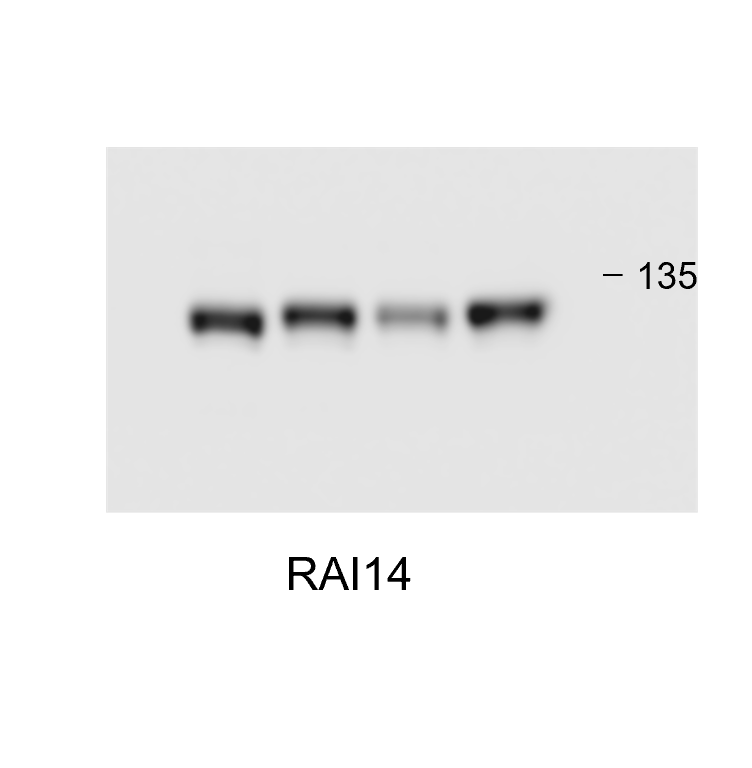

Supplement: Supplementary file 6 — Source data Fig. 4 [file 44319_2024_228_MOESM6_ESM.zip › Figure 4/Figure 4E/RAI14.tif]

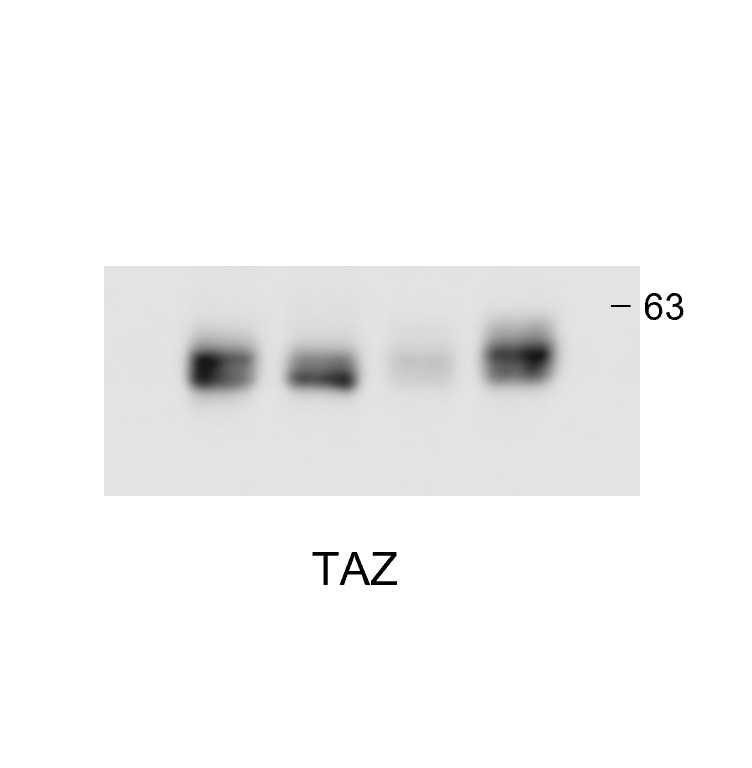

Supplement: Supplementary file 6 — Source data Fig. 4 [file 44319_2024_228_MOESM6_ESM.zip › Figure 4/Figure 4E/TAZ.tif]

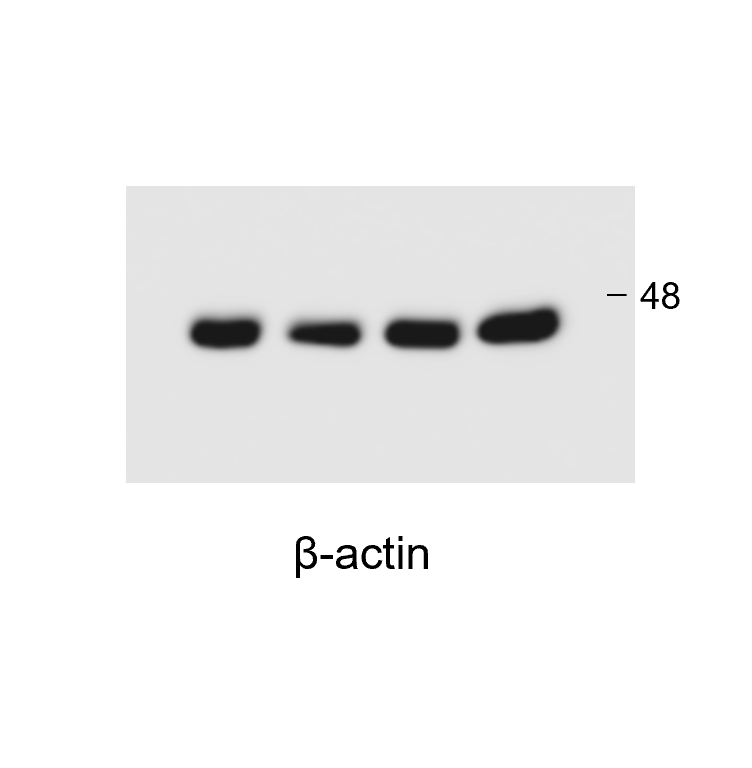

Supplement: Supplementary file 6 — Source data Fig. 4 [file 44319_2024_228_MOESM6_ESM.zip › Figure 4/Figure 4E/ÑΓ-actin.tif]

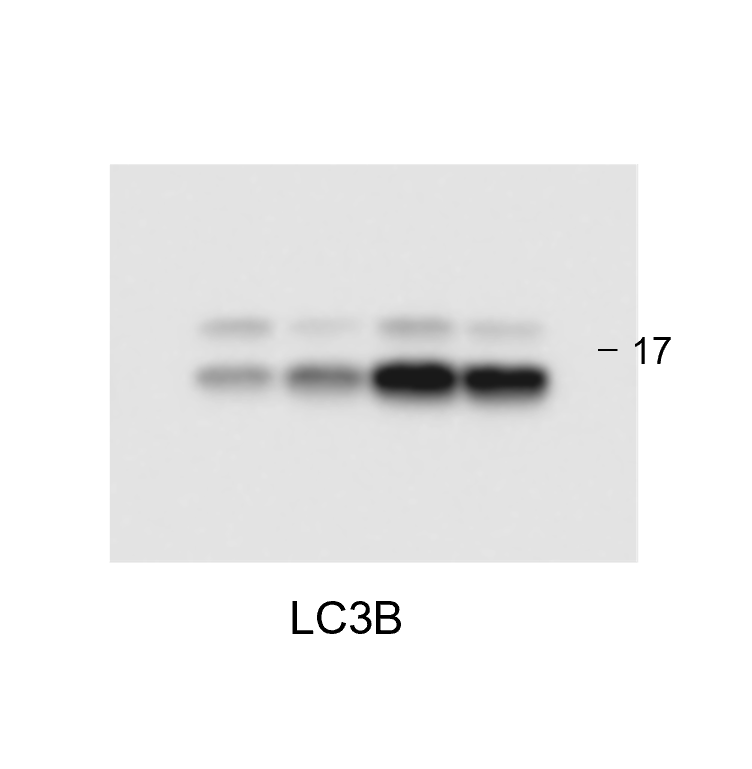

Supplement: Supplementary file 6 — Source data Fig. 4 [file 44319_2024_228_MOESM6_ESM.zip › Figure 4/Figure 4F/LC3B.tif]

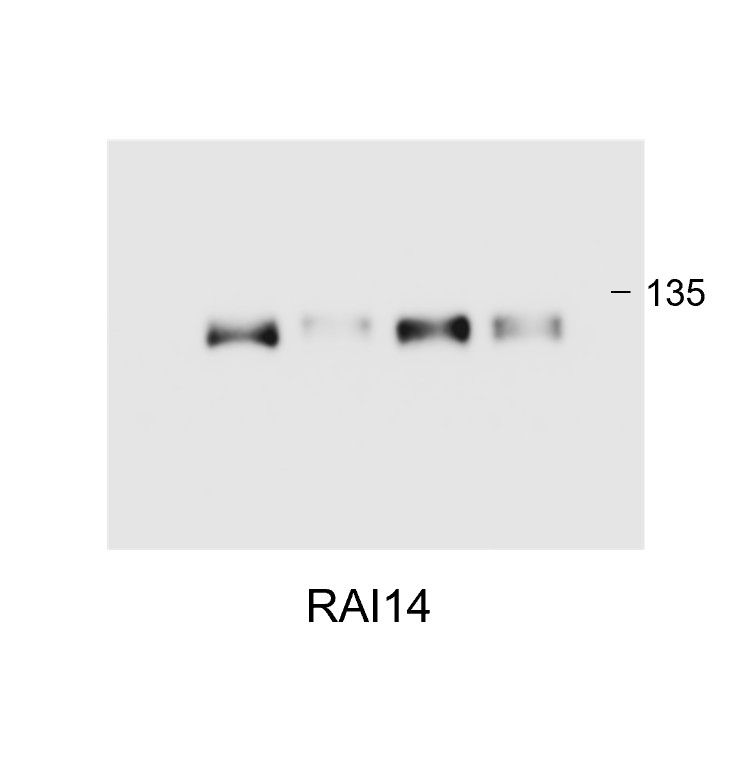

Supplement: Supplementary file 6 — Source data Fig. 4 [file 44319_2024_228_MOESM6_ESM.zip › Figure 4/Figure 4F/RAI14.tif]

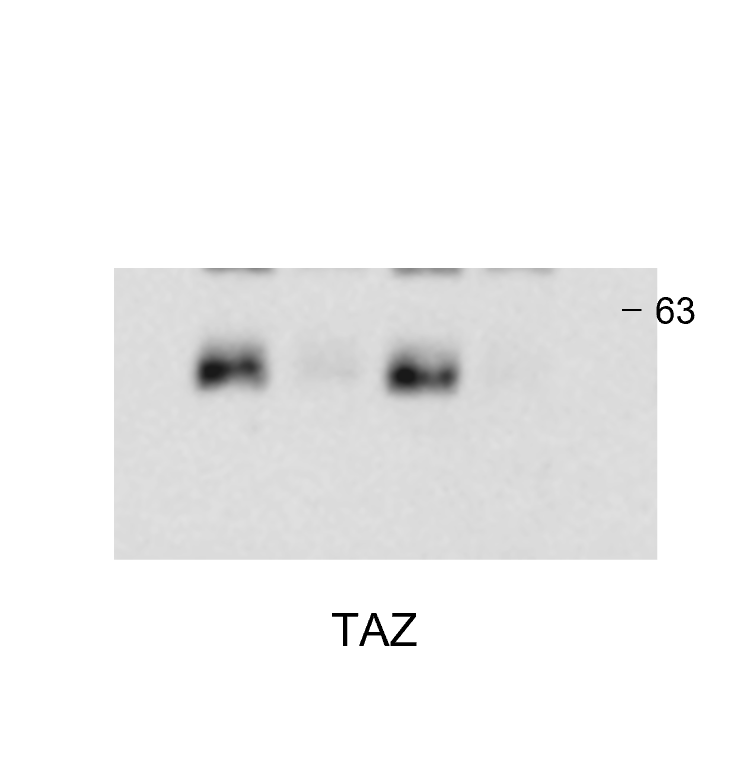

Supplement: Supplementary file 6 — Source data Fig. 4 [file 44319_2024_228_MOESM6_ESM.zip › Figure 4/Figure 4F/TAZ.tif]

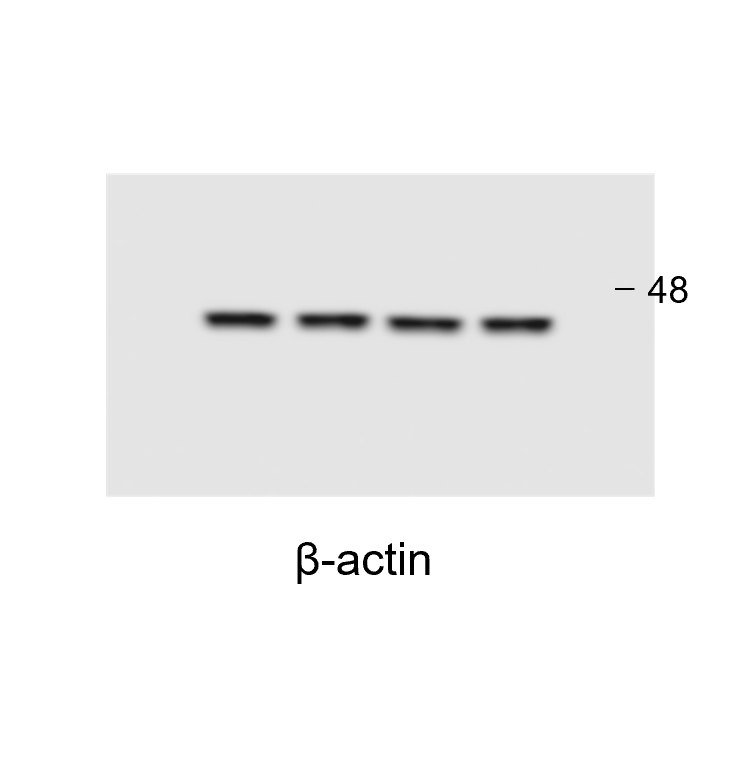

Supplement: Supplementary file 6 — Source data Fig. 4 [file 44319_2024_228_MOESM6_ESM.zip › Figure 4/Figure 4F/ÑΓ-actin.tif]

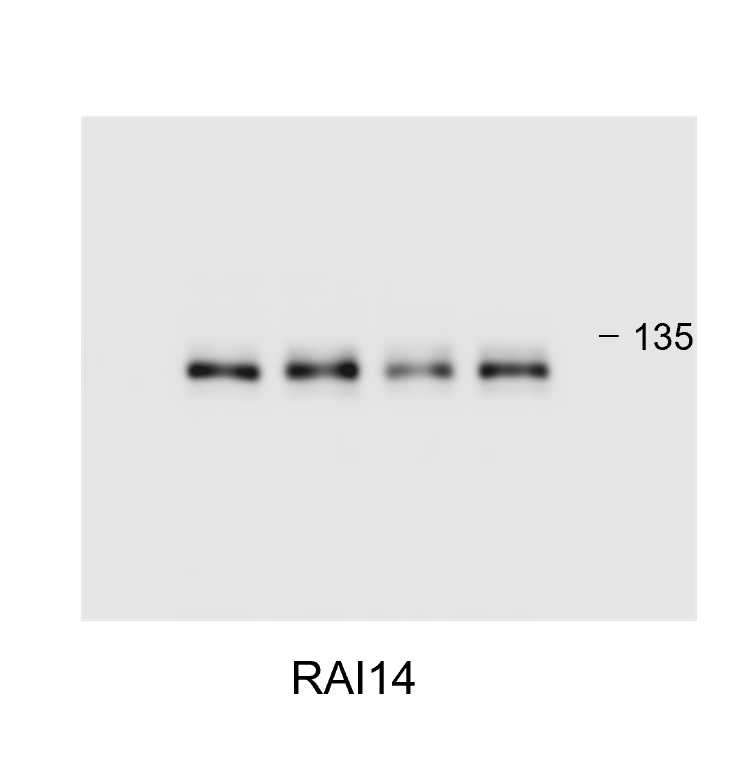

Supplement: Supplementary file 6 — Source data Fig. 4 [file 44319_2024_228_MOESM6_ESM.zip › Figure 4/Figure 4G/RAI14.tif]

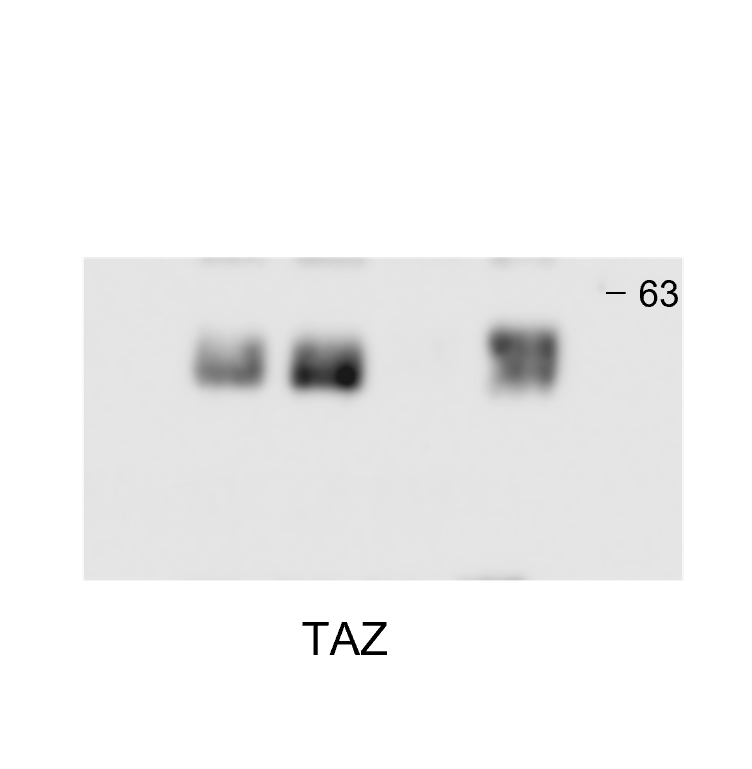

Supplement: Supplementary file 6 — Source data Fig. 4 [file 44319_2024_228_MOESM6_ESM.zip › Figure 4/Figure 4G/TAZ.tif]

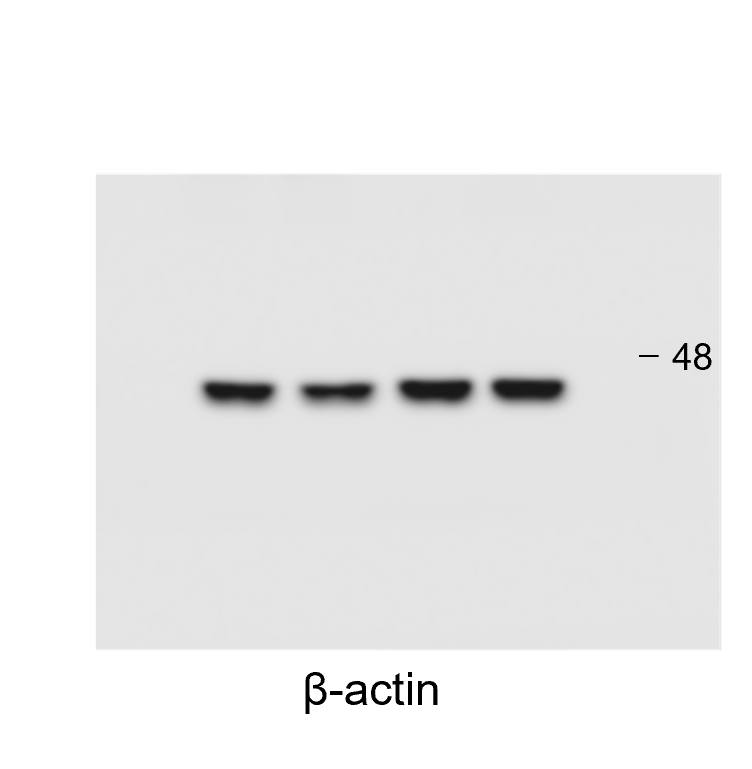

Supplement: Supplementary file 6 — Source data Fig. 4 [file 44319_2024_228_MOESM6_ESM.zip › Figure 4/Figure 4G/ÑΓ-actin.tif]

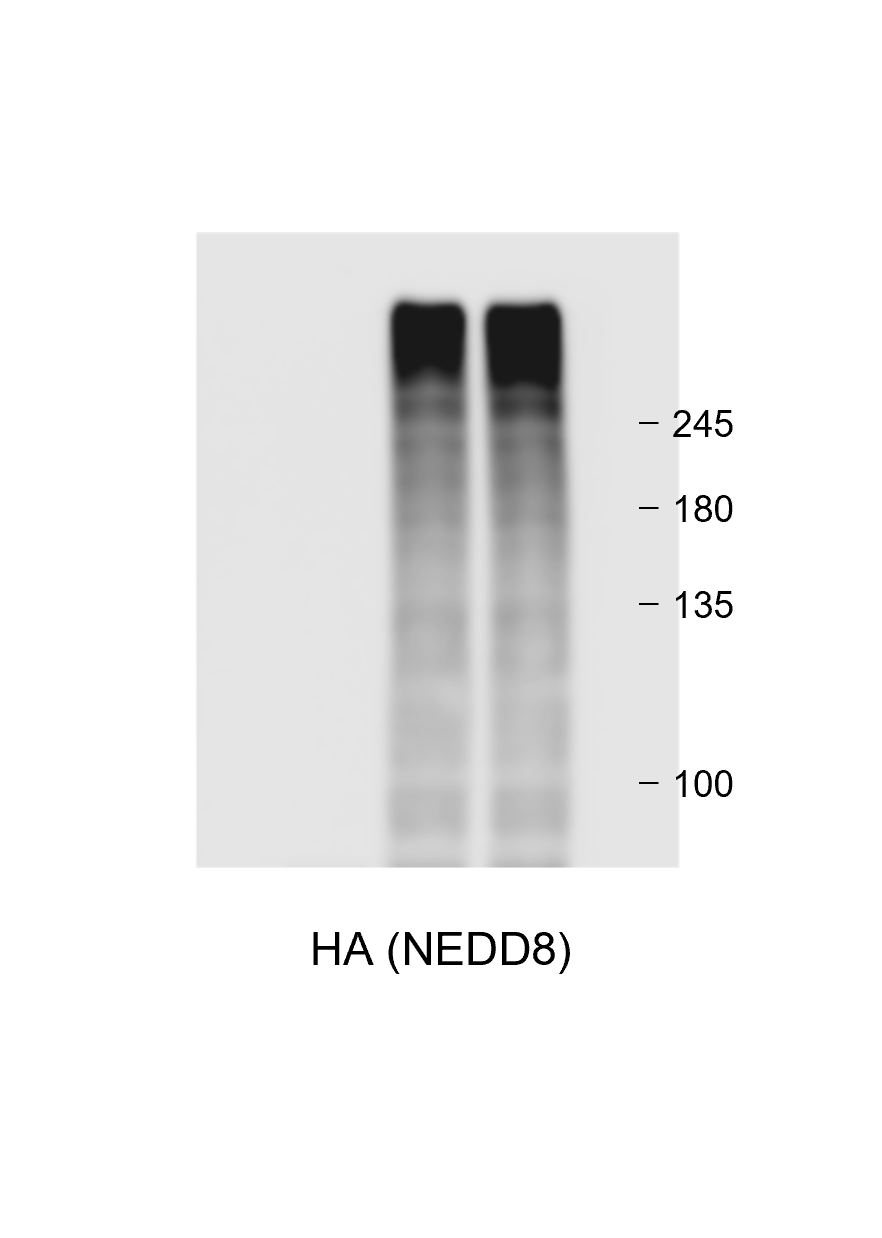

Supplement: Supplementary file 6 — Source data Fig. 4 [file 44319_2024_228_MOESM6_ESM.zip › Figure 4/Figure 4H/HA(NEDD8).tif]

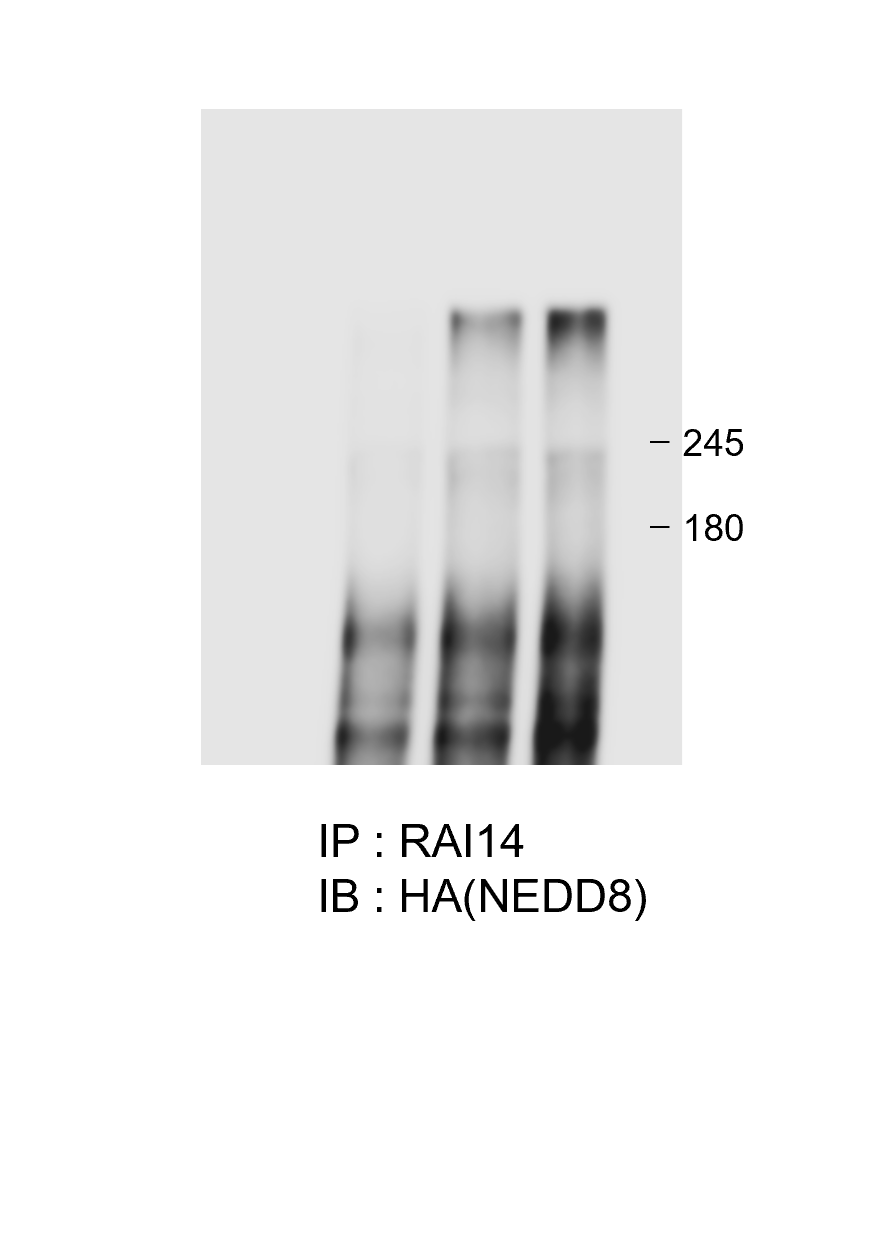

Supplement: Supplementary file 6 — Source data Fig. 4 [file 44319_2024_228_MOESM6_ESM.zip › Figure 4/Figure 4H/IP RAI14, IB HA(NEDD8).tif]

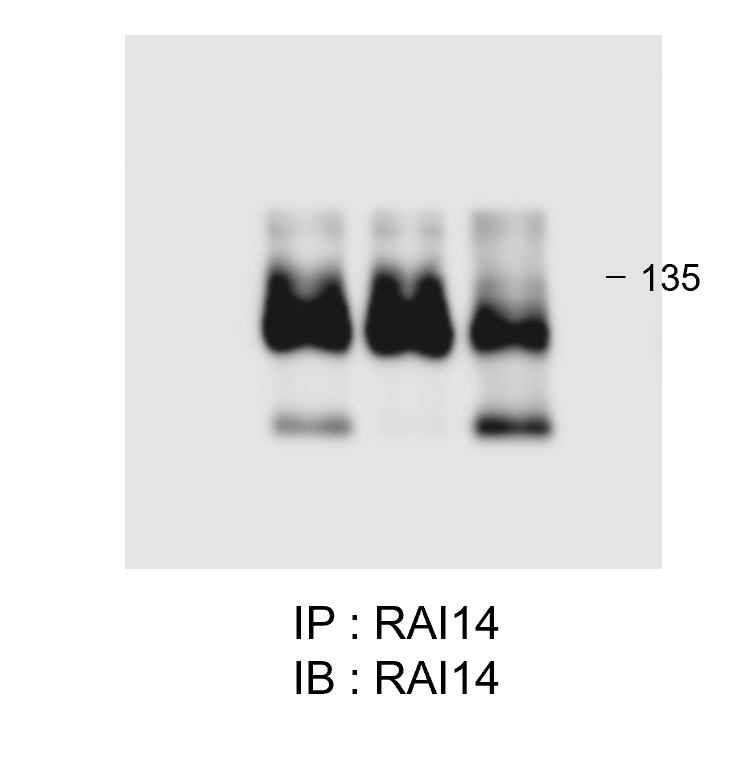

Supplement: Supplementary file 6 — Source data Fig. 4 [file 44319_2024_228_MOESM6_ESM.zip › Figure 4/Figure 4H/IP RAI14, IB RAI14.tif]

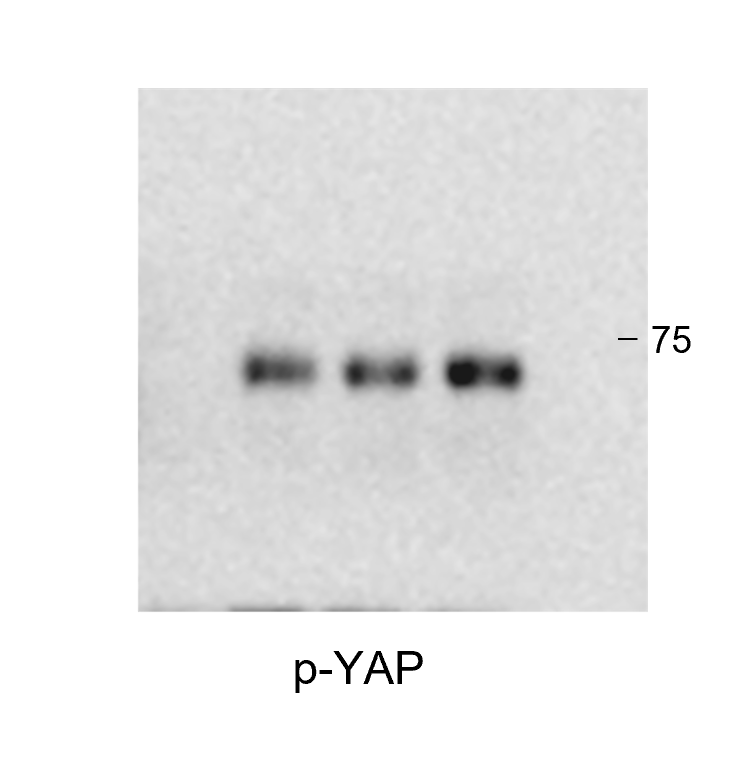

Supplement: Supplementary file 6 — Source data Fig. 4 [file 44319_2024_228_MOESM6_ESM.zip › Figure 4/Figure 4H/p-YAP.tif]

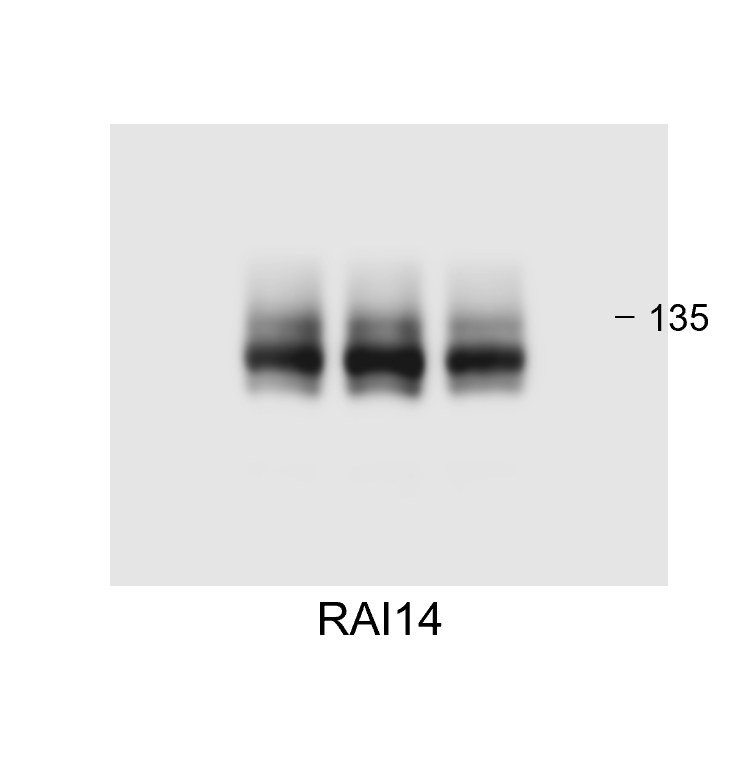

Supplement: Supplementary file 6 — Source data Fig. 4 [file 44319_2024_228_MOESM6_ESM.zip › Figure 4/Figure 4H/RAI14.tif]

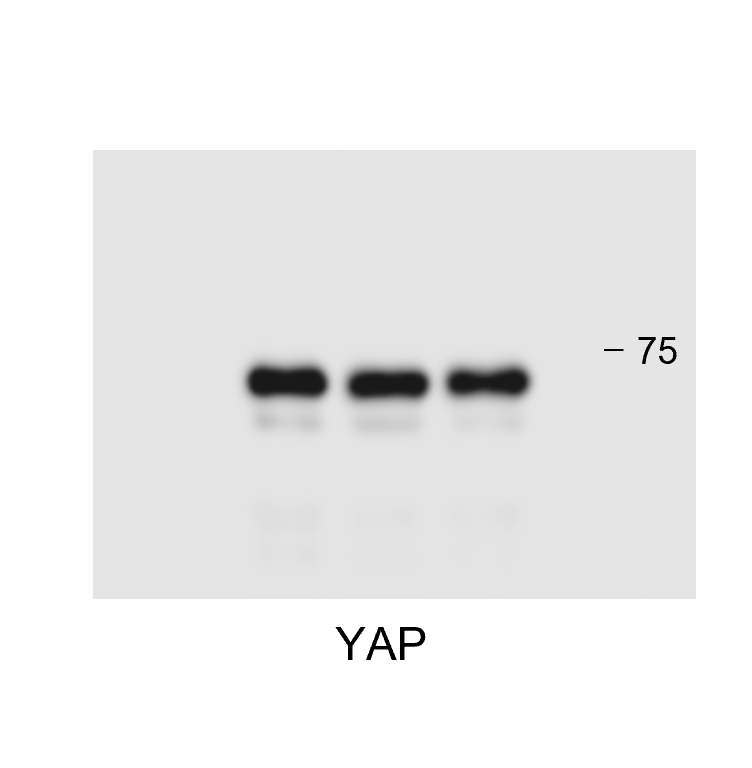

Supplement: Supplementary file 6 — Source data Fig. 4 [file 44319_2024_228_MOESM6_ESM.zip › Figure 4/Figure 4H/YAP.tif]

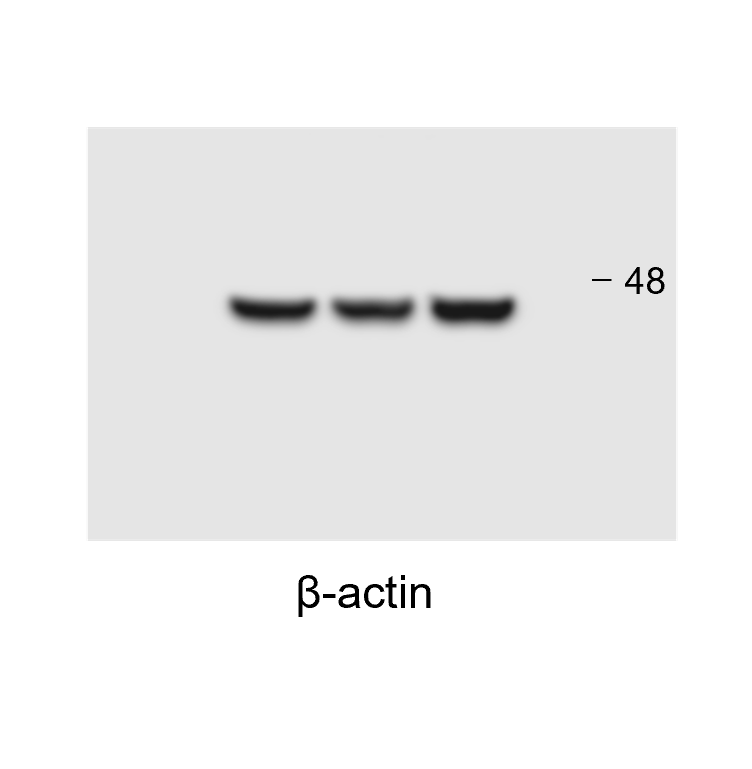

Supplement: Supplementary file 6 — Source data Fig. 4 [file 44319_2024_228_MOESM6_ESM.zip › Figure 4/Figure 4H/ÑΓ-actin.tif]

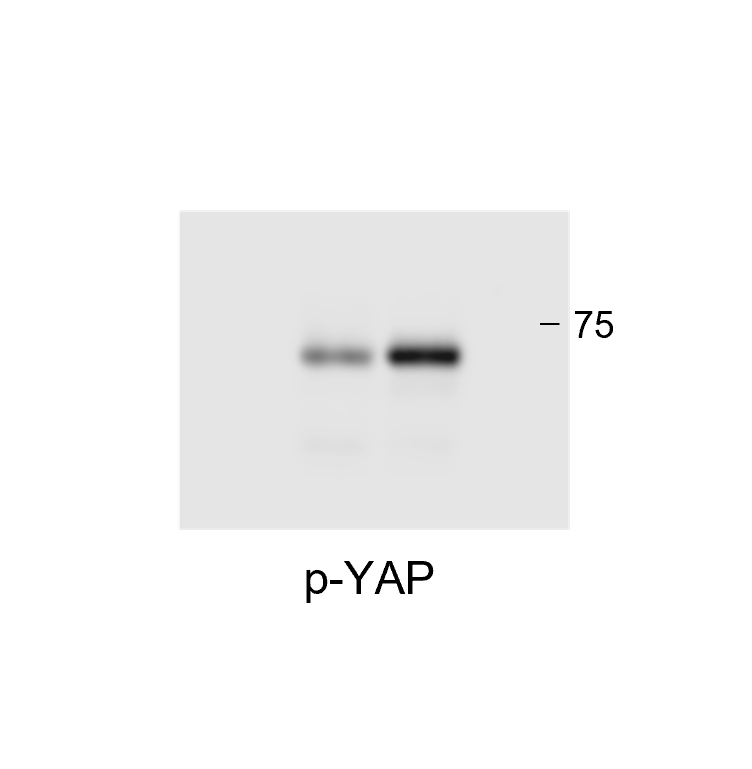

Supplement: Supplementary file 6 — Source data Fig. 4 [file 44319_2024_228_MOESM6_ESM.zip › Figure 4/Figure 4I/p-YAP.tif]

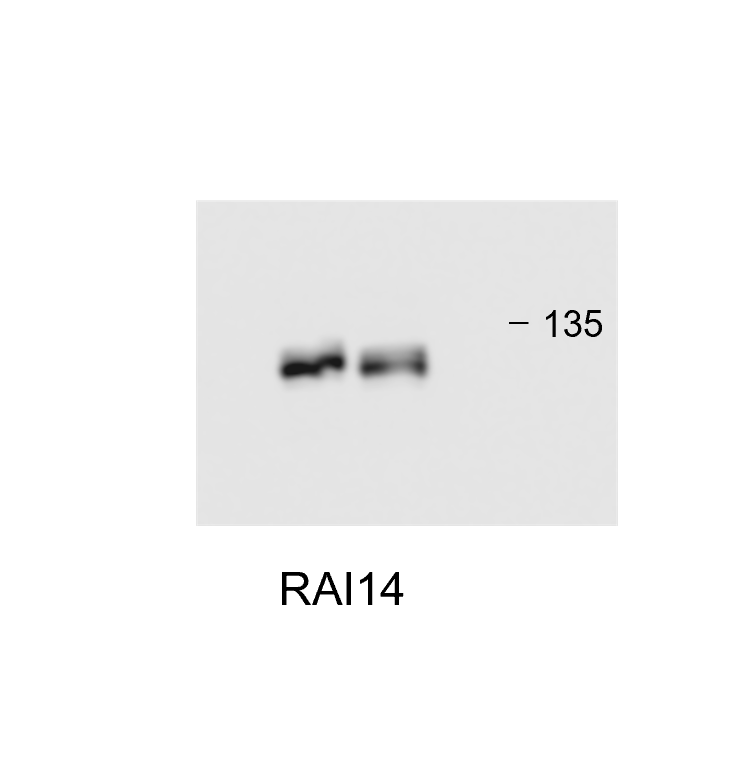

Supplement: Supplementary file 6 — Source data Fig. 4 [file 44319_2024_228_MOESM6_ESM.zip › Figure 4/Figure 4I/RAI14.tif]

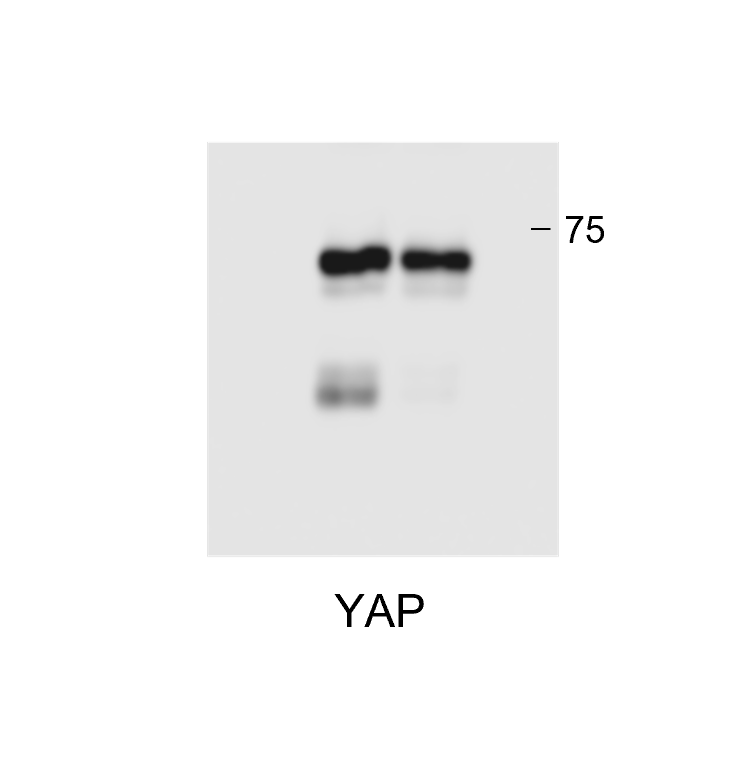

Supplement: Supplementary file 6 — Source data Fig. 4 [file 44319_2024_228_MOESM6_ESM.zip › Figure 4/Figure 4I/YAP.tif]

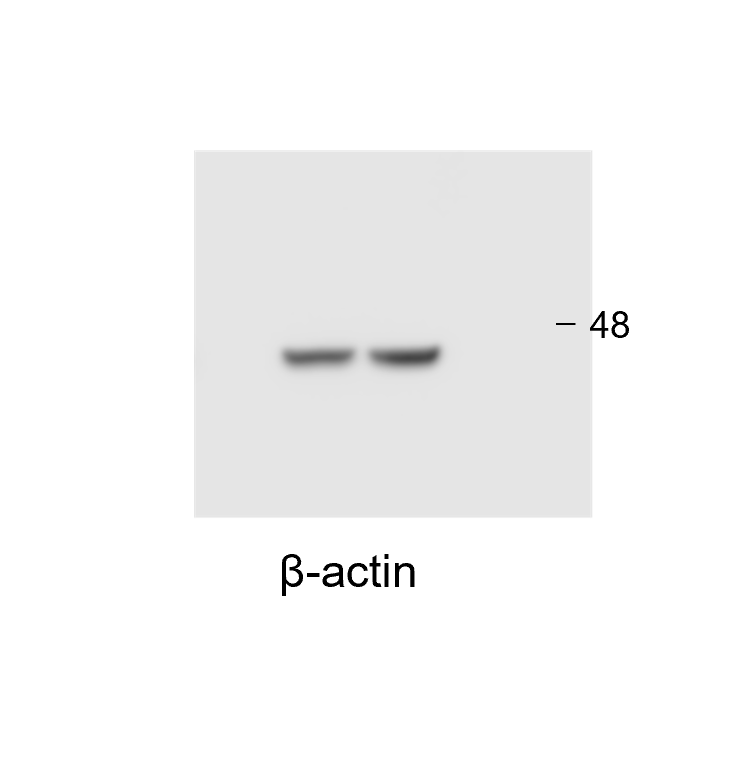

Supplement: Supplementary file 6 — Source data Fig. 4 [file 44319_2024_228_MOESM6_ESM.zip › Figure 4/Figure 4I/ÑΓ-actin.tif]

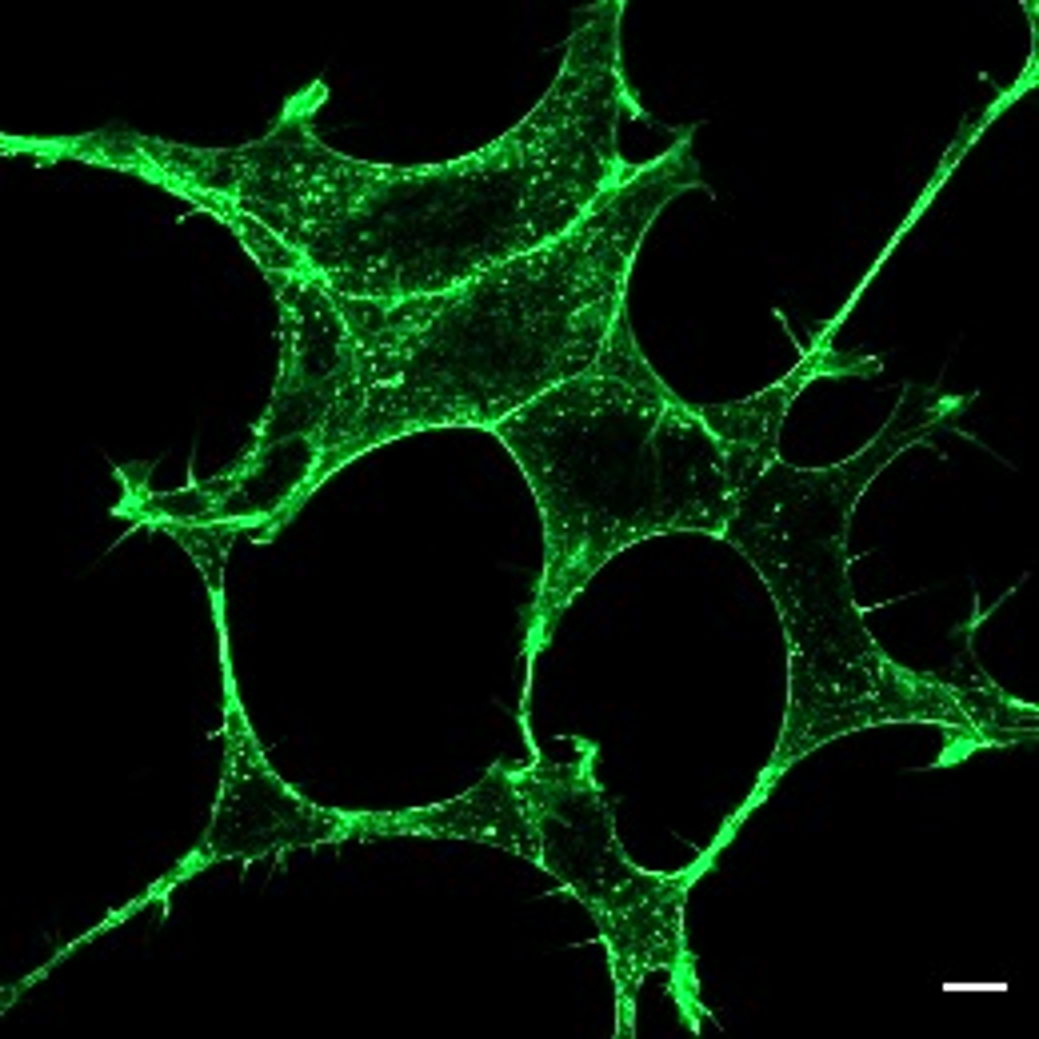

Supplement: Supplementary file 6 — Source data Fig. 4 [file 44319_2024_228_MOESM6_ESM.zip › Figure 4/Figure 4J/F-actin, serum -.tif]

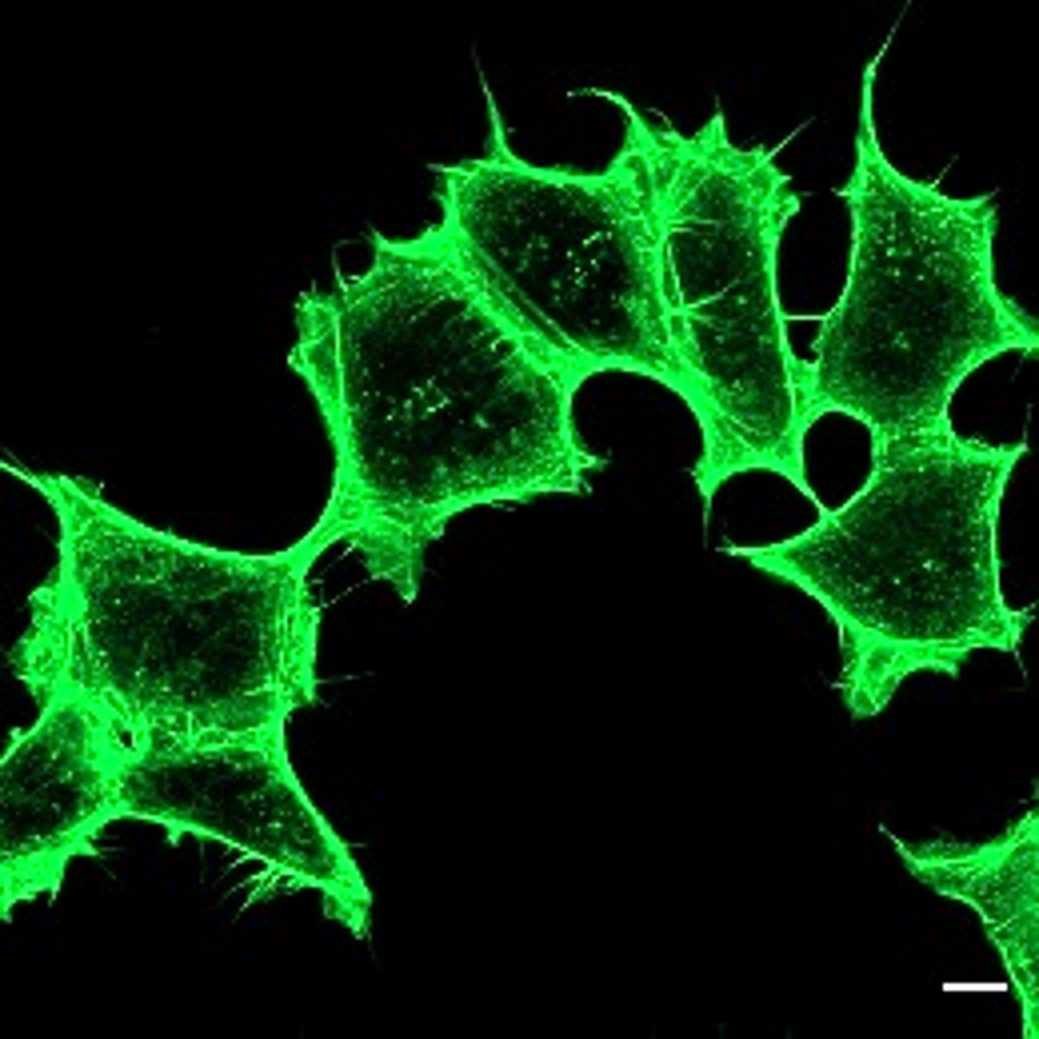

Supplement: Supplementary file 6 — Source data Fig. 4 [file 44319_2024_228_MOESM6_ESM.zip › Figure 4/Figure 4J/F-actin, serum +.tif]

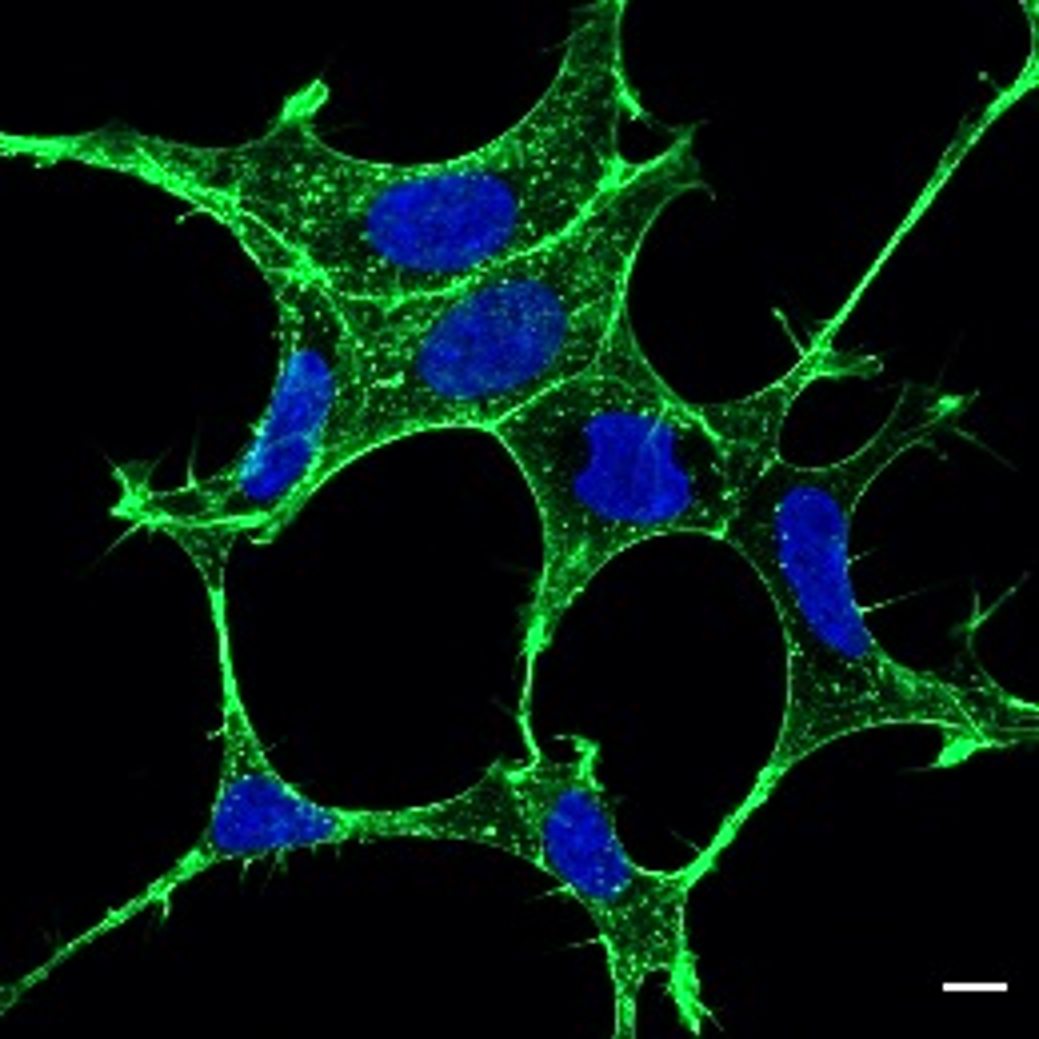

Supplement: Supplementary file 6 — Source data Fig. 4 [file 44319_2024_228_MOESM6_ESM.zip › Figure 4/Figure 4J/F-actin+DAPI, serum -.tif]

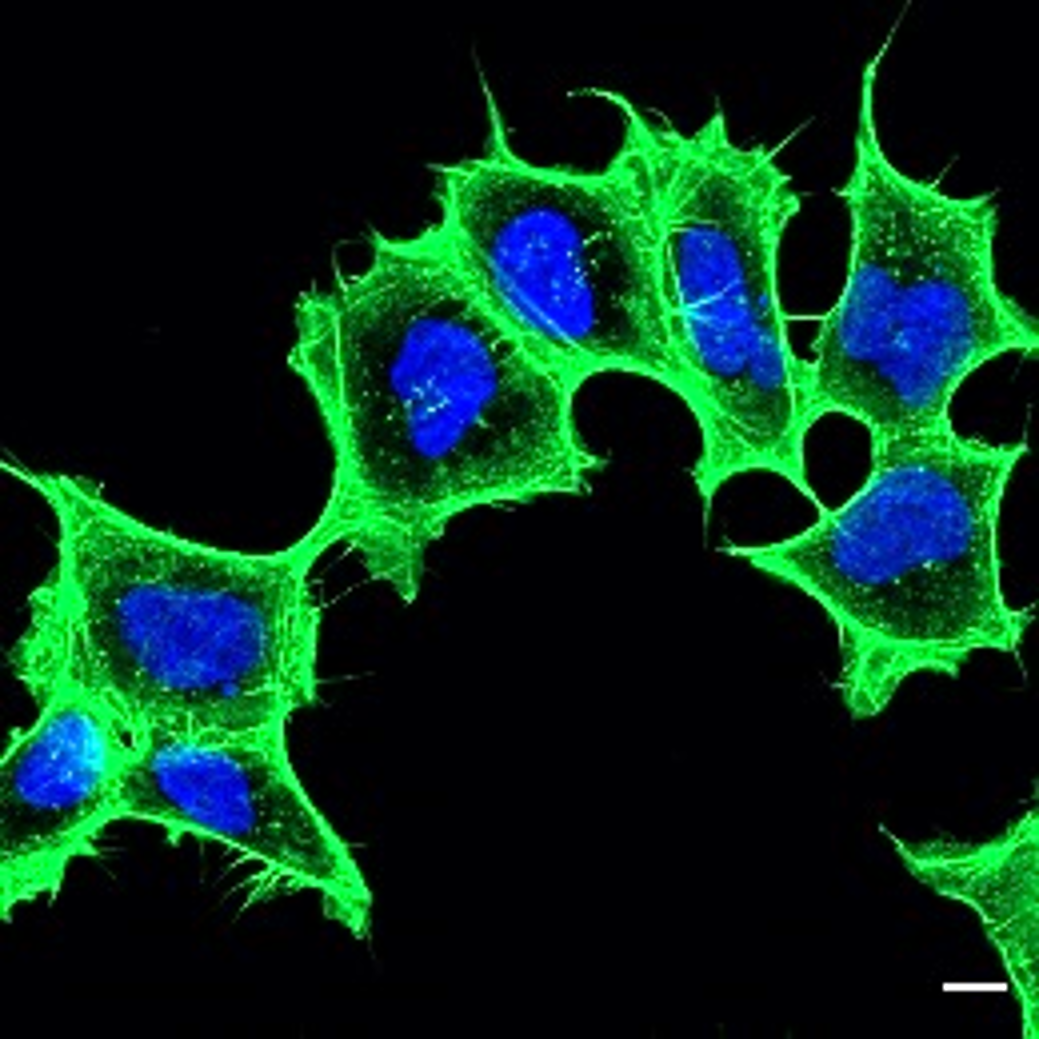

Supplement: Supplementary file 6 — Source data Fig. 4 [file 44319_2024_228_MOESM6_ESM.zip › Figure 4/Figure 4J/F-actin+DAPI, serum +.tif]

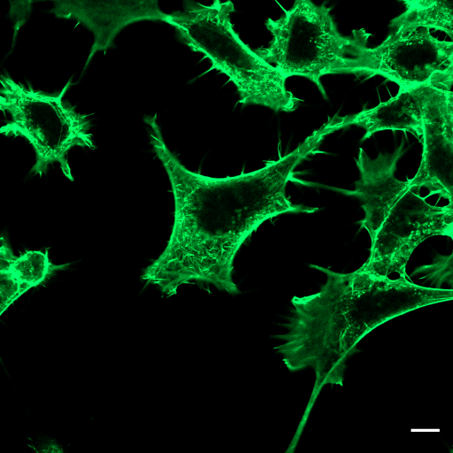

Supplement: Supplementary file 7 — Source data Fig. 5 [file 44319_2024_228_MOESM7_ESM.zip › Figure 5/Figure 5A/F-actin.tif]

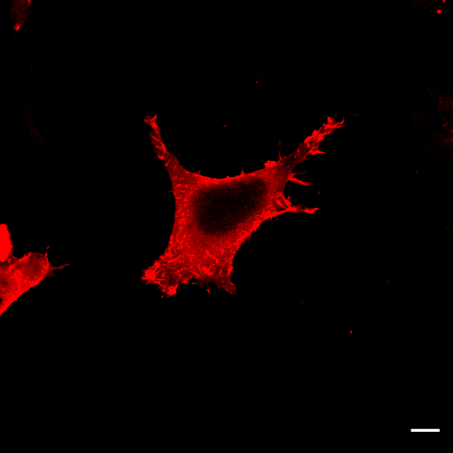

Supplement: Supplementary file 7 — Source data Fig. 5 [file 44319_2024_228_MOESM7_ESM.zip › Figure 5/Figure 5A/Flag-NF2.tif]

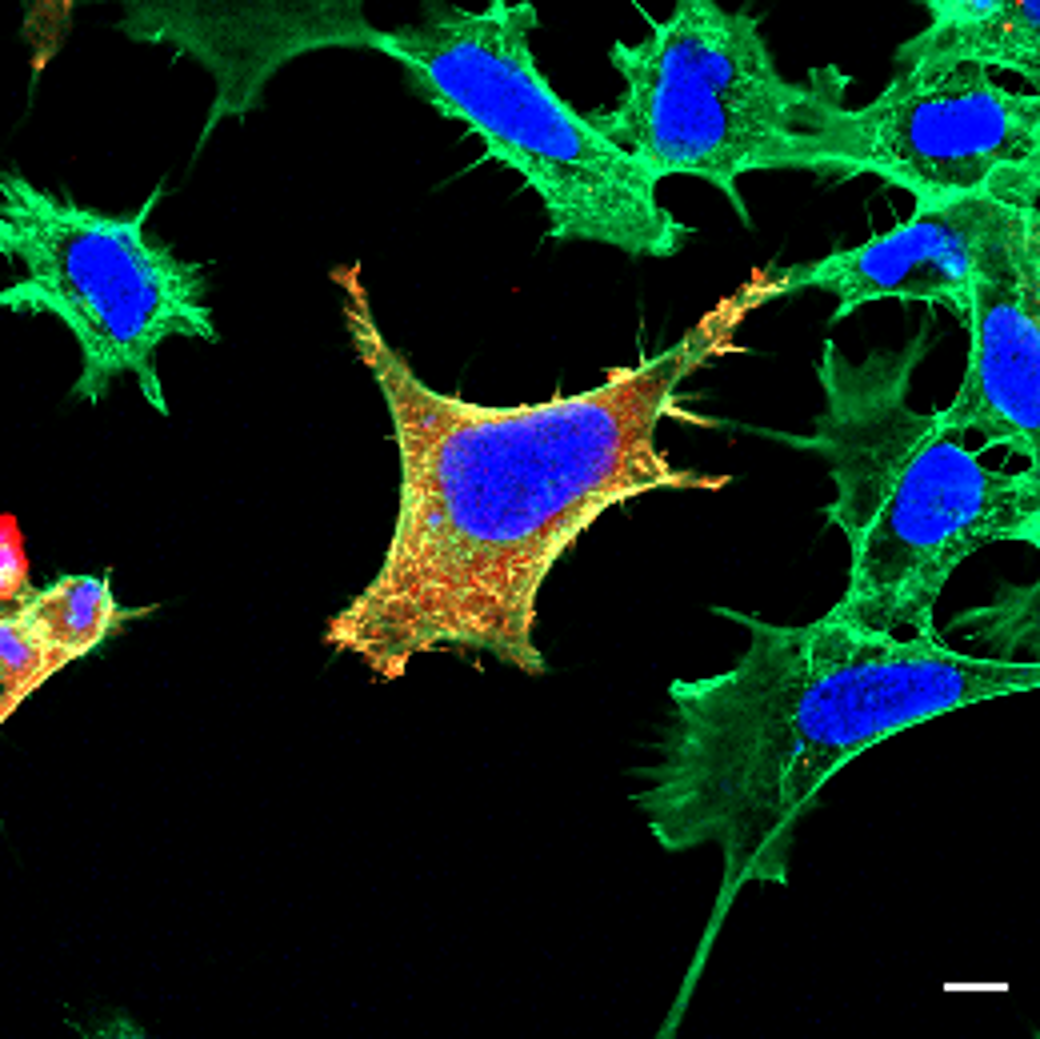

Supplement: Supplementary file 7 — Source data Fig. 5 [file 44319_2024_228_MOESM7_ESM.zip › Figure 5/Figure 5A/Merge.tif]

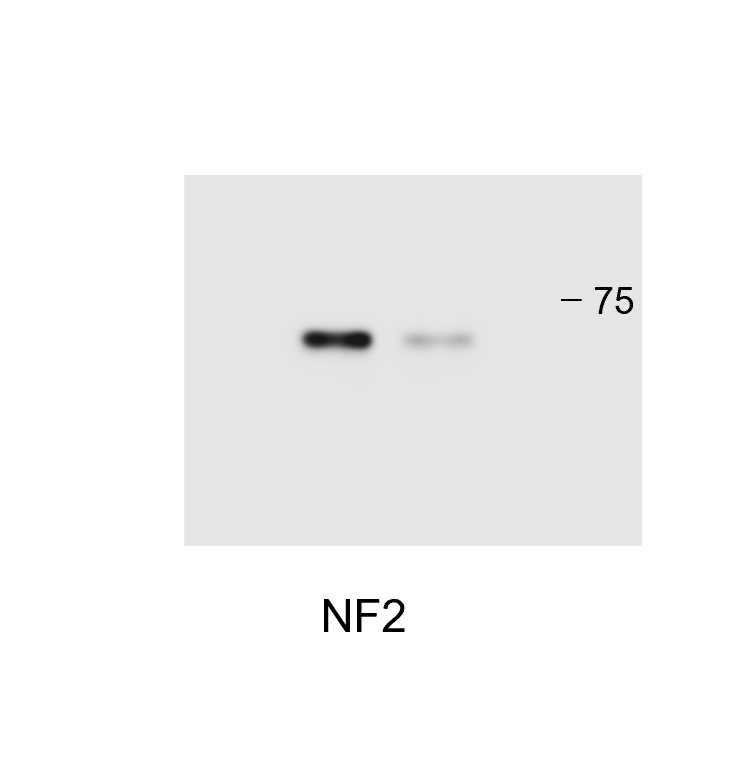

Supplement: Supplementary file 7 — Source data Fig. 5 [file 44319_2024_228_MOESM7_ESM.zip › Figure 5/Figure 5B/NF2.tif]

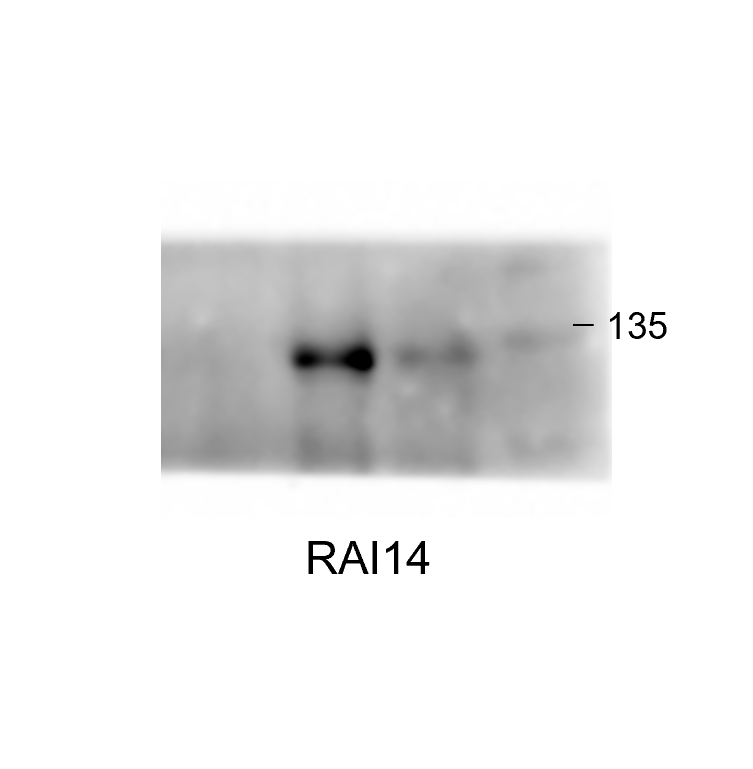

Supplement: Supplementary file 7 — Source data Fig. 5 [file 44319_2024_228_MOESM7_ESM.zip › Figure 5/Figure 5B/RAI14.tif]

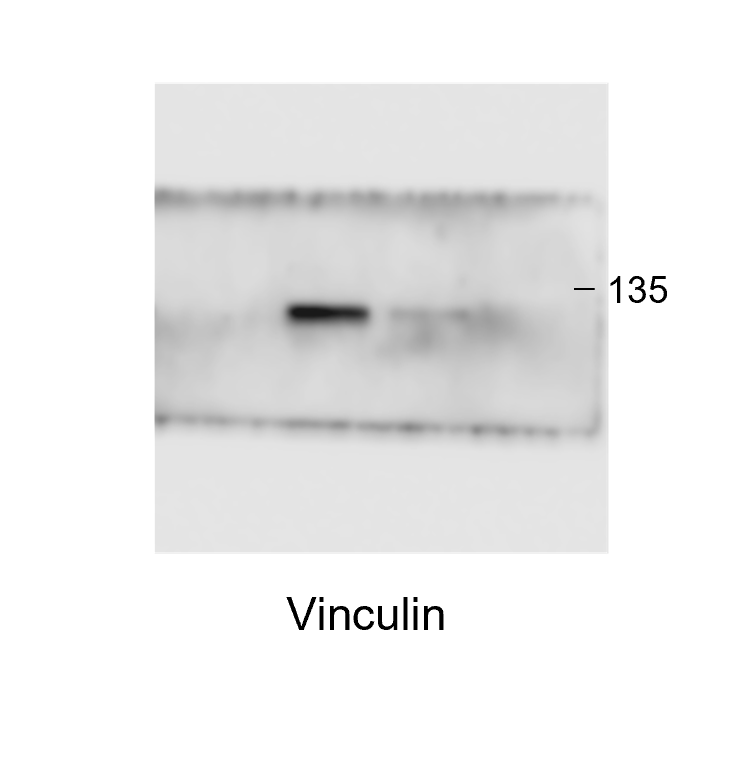

Supplement: Supplementary file 7 — Source data Fig. 5 [file 44319_2024_228_MOESM7_ESM.zip › Figure 5/Figure 5B/Vinculin.tif]

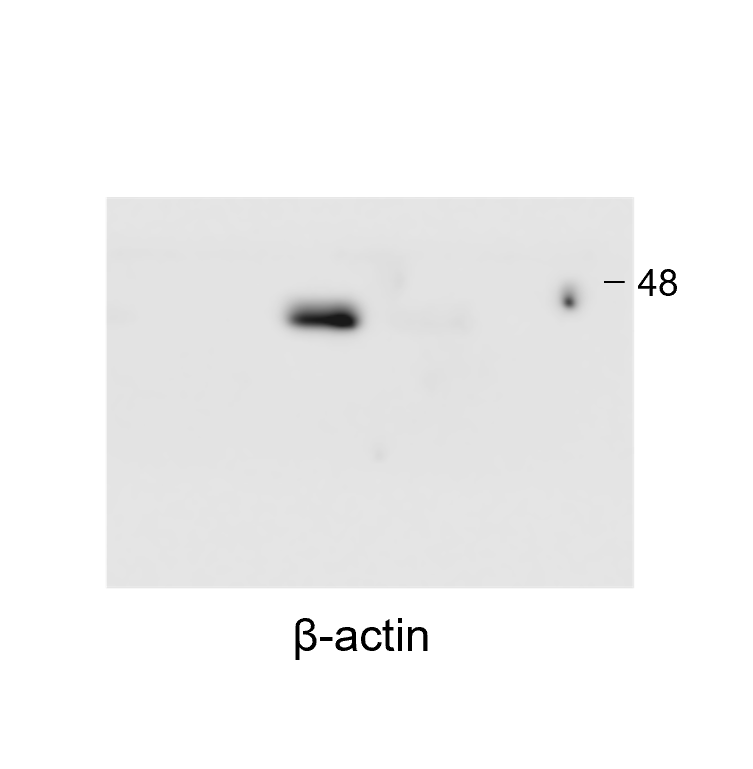

Supplement: Supplementary file 7 — Source data Fig. 5 [file 44319_2024_228_MOESM7_ESM.zip › Figure 5/Figure 5B/ÑΓ-actin.tif]

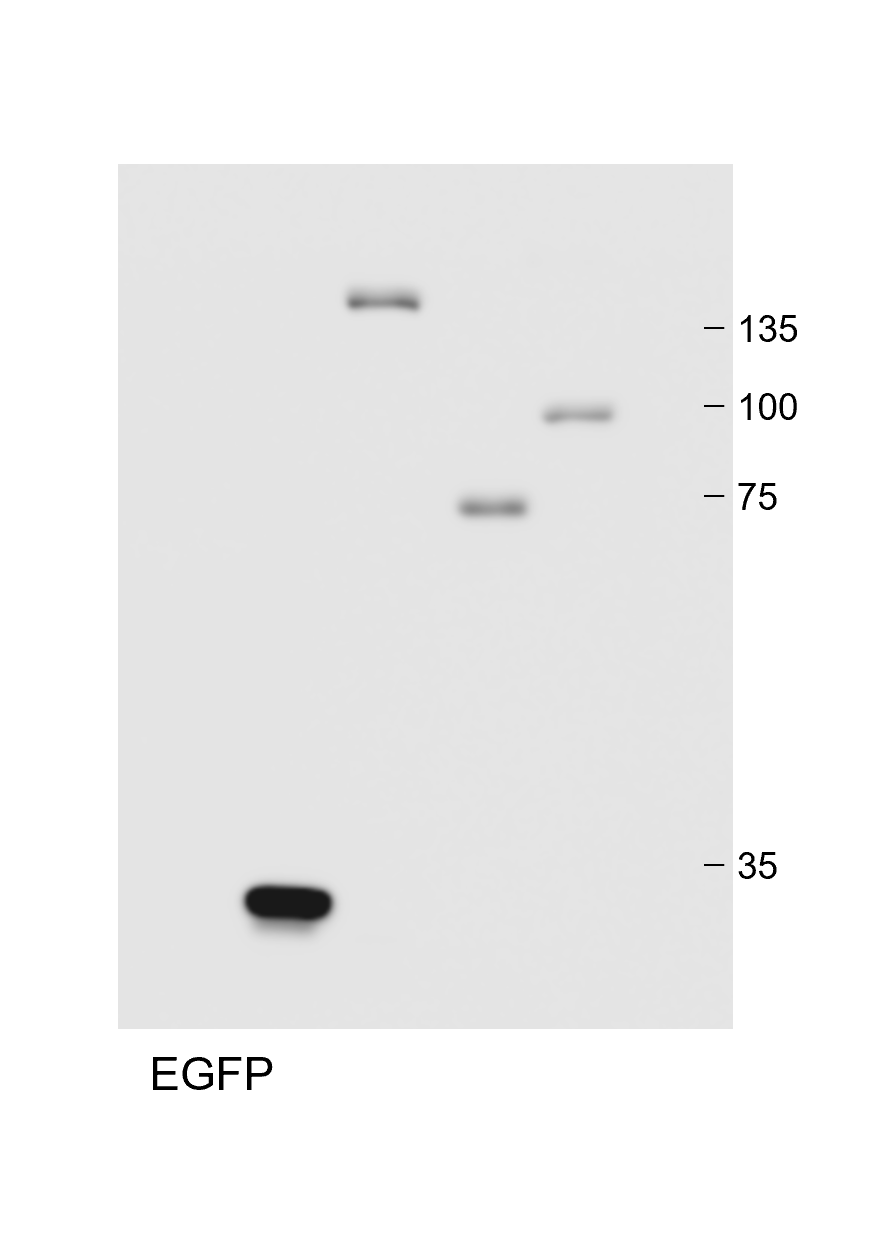

Supplement: Supplementary file 7 — Source data Fig. 5 [file 44319_2024_228_MOESM7_ESM.zip › Figure 5/Figure 5C/EGFP.tif]

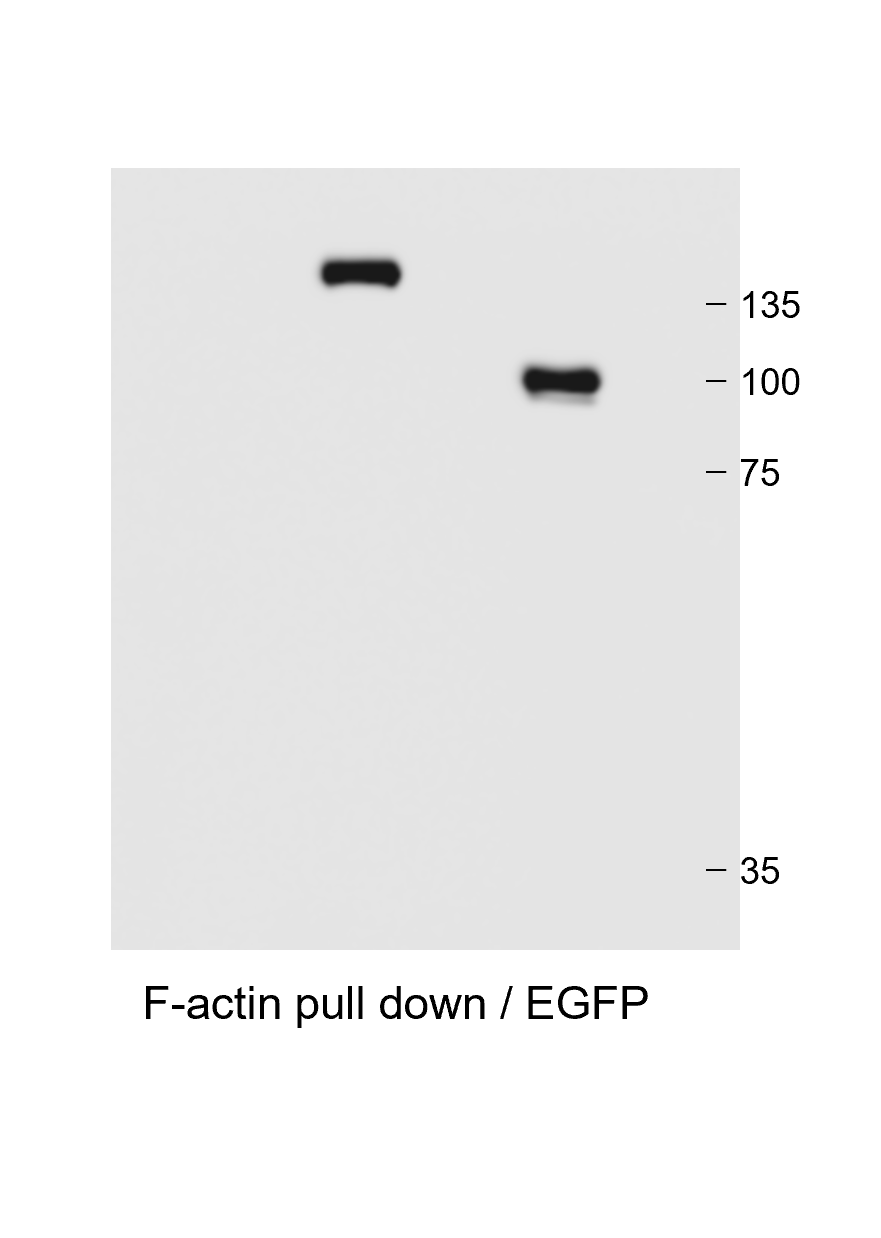

Supplement: Supplementary file 7 — Source data Fig. 5 [file 44319_2024_228_MOESM7_ESM.zip › Figure 5/Figure 5C/F-actin pull down, EGFP.tif]

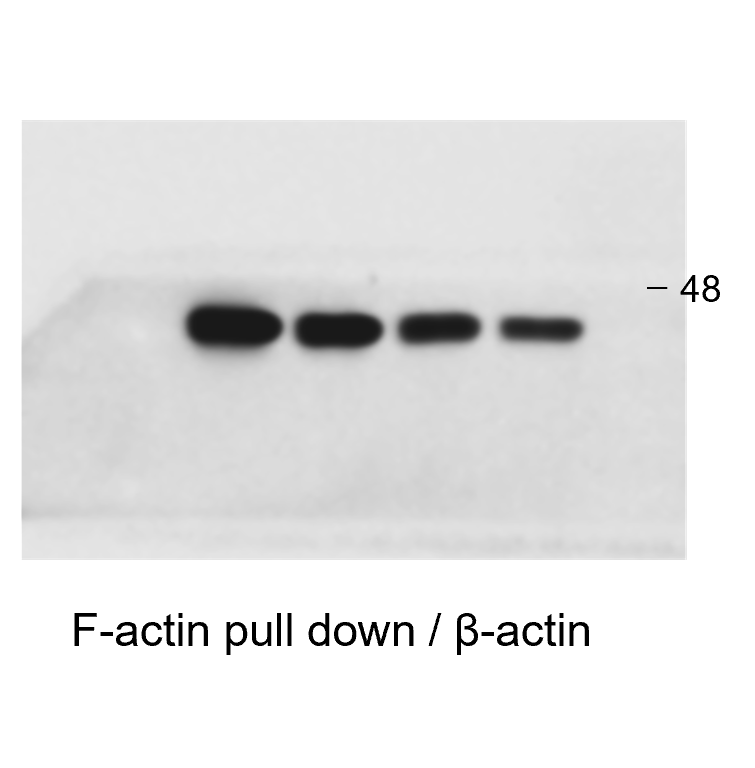

Supplement: Supplementary file 7 — Source data Fig. 5 [file 44319_2024_228_MOESM7_ESM.zip › Figure 5/Figure 5C/F-actin pull down, ÑΓ-actin.tif]

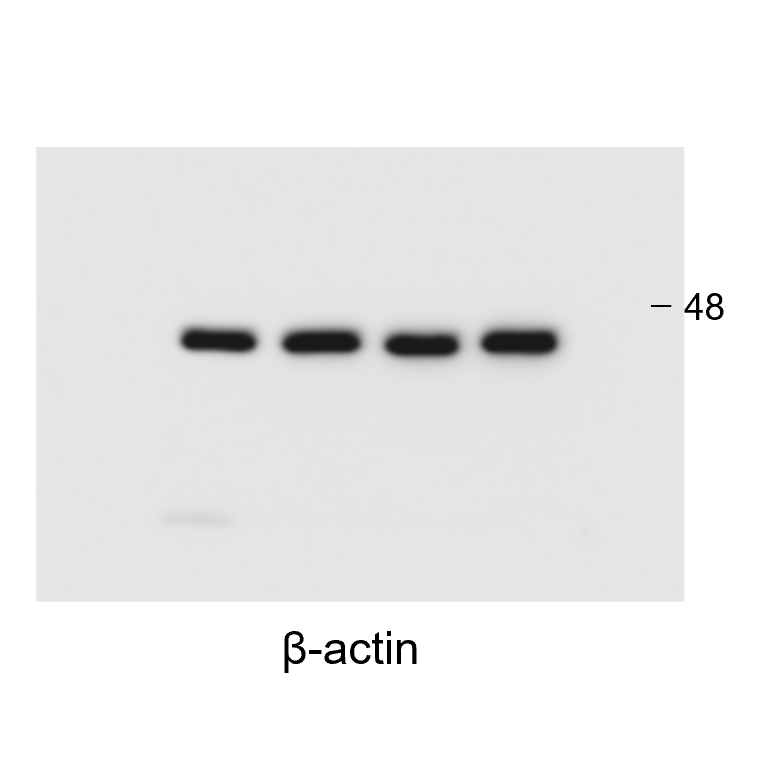

Supplement: Supplementary file 7 — Source data Fig. 5 [file 44319_2024_228_MOESM7_ESM.zip › Figure 5/Figure 5C/ÑΓ-actin.tif]

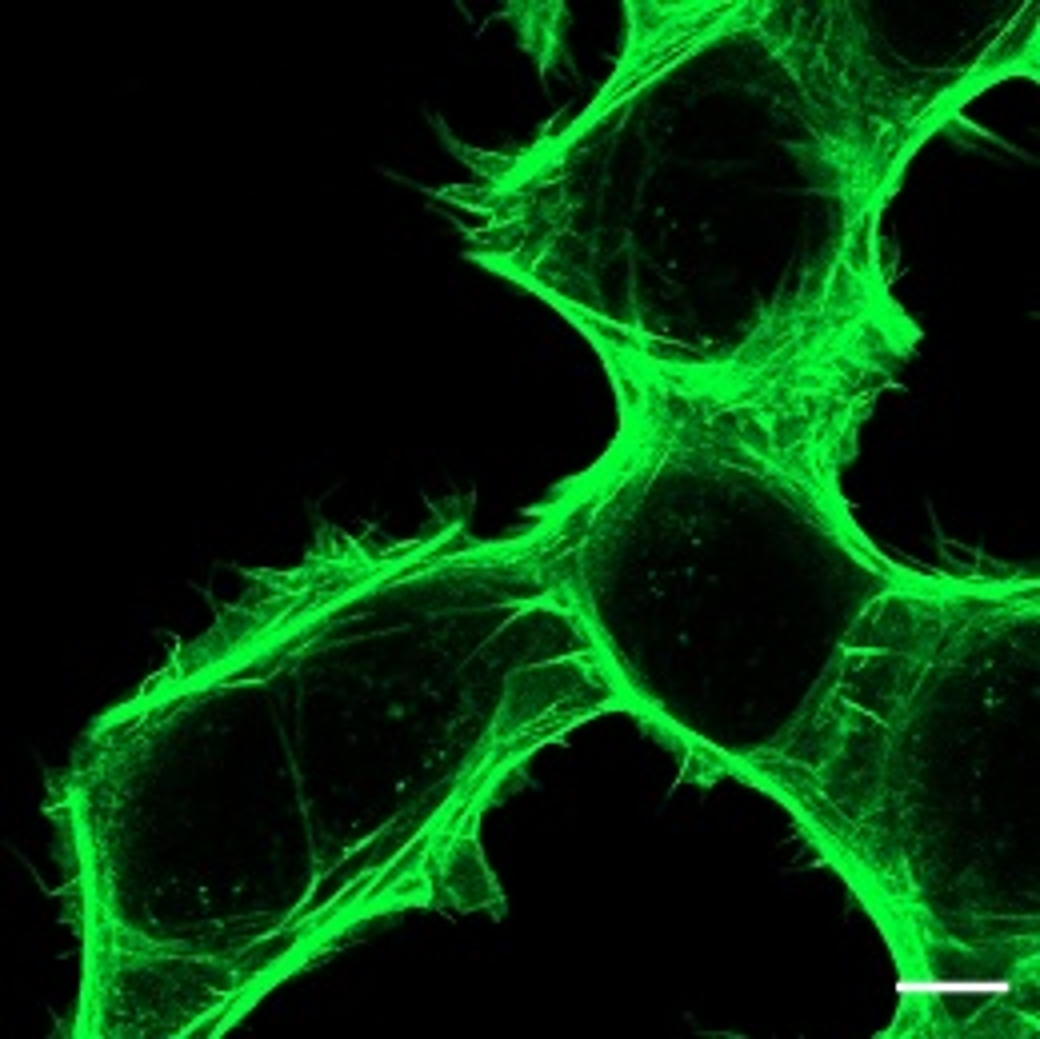

Supplement: Supplementary file 7 — Source data Fig. 5 [file 44319_2024_228_MOESM7_ESM.zip › Figure 5/Figure 5D/F-actin.tif]

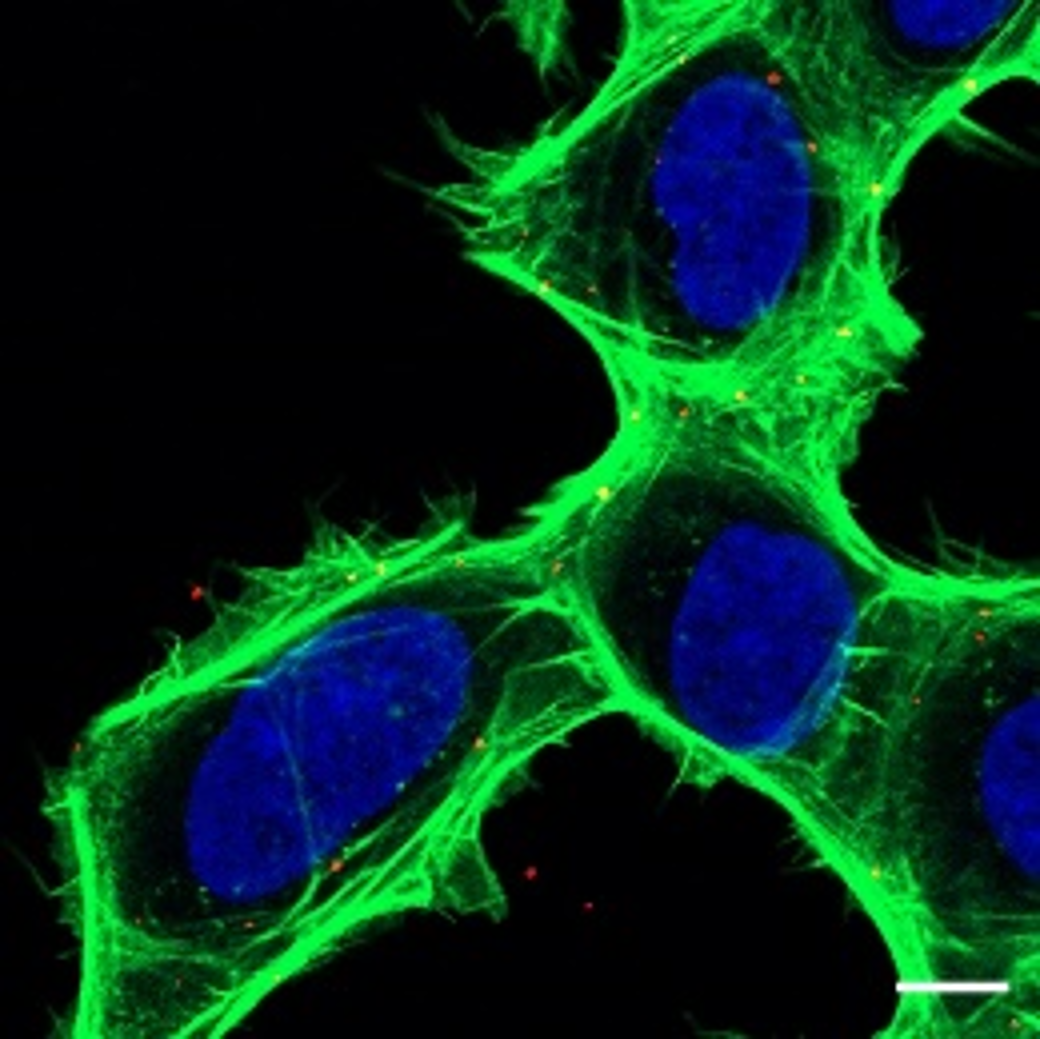

Supplement: Supplementary file 7 — Source data Fig. 5 [file 44319_2024_228_MOESM7_ESM.zip › Figure 5/Figure 5D/Merge.tif]

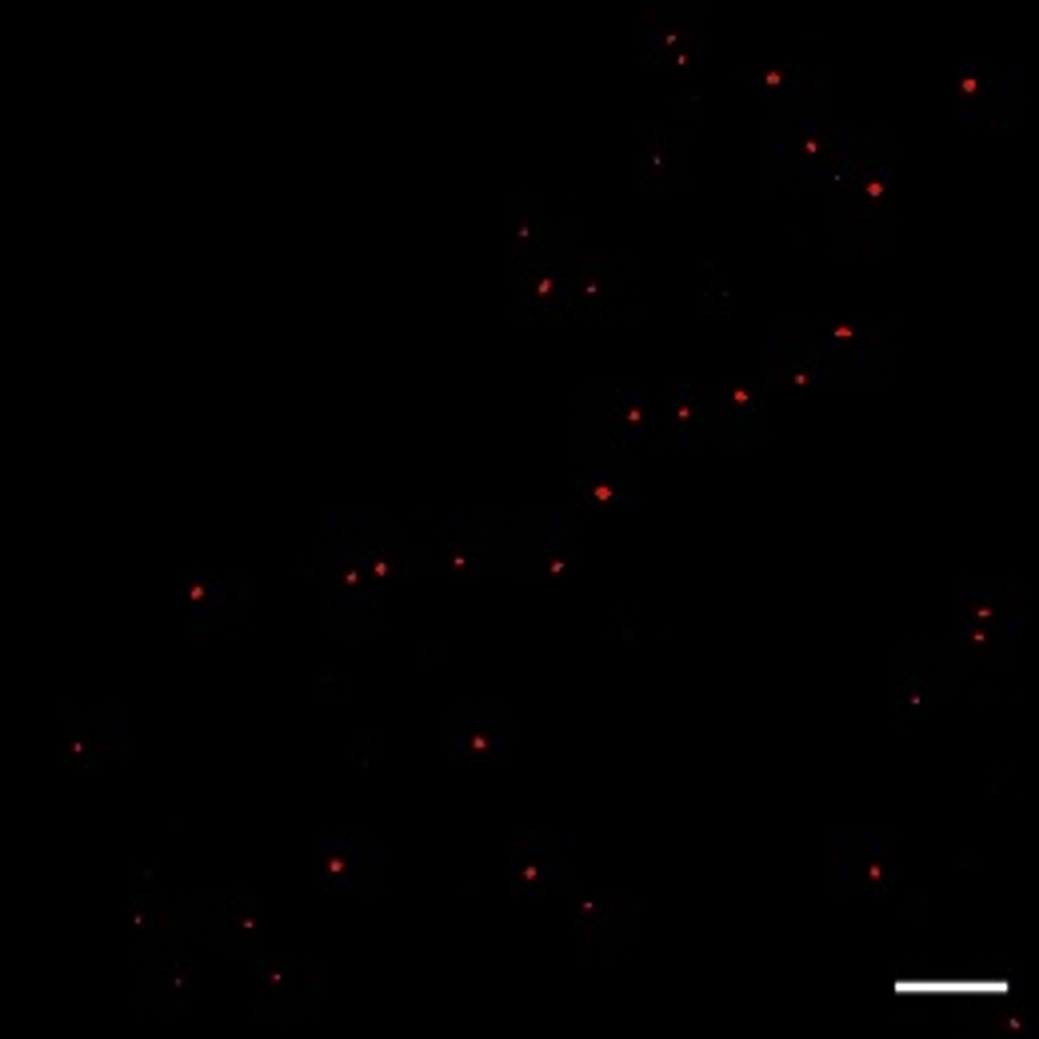

Supplement: Supplementary file 7 — Source data Fig. 5 [file 44319_2024_228_MOESM7_ESM.zip › Figure 5/Figure 5D/PLA(NF2, RAI14).tif]

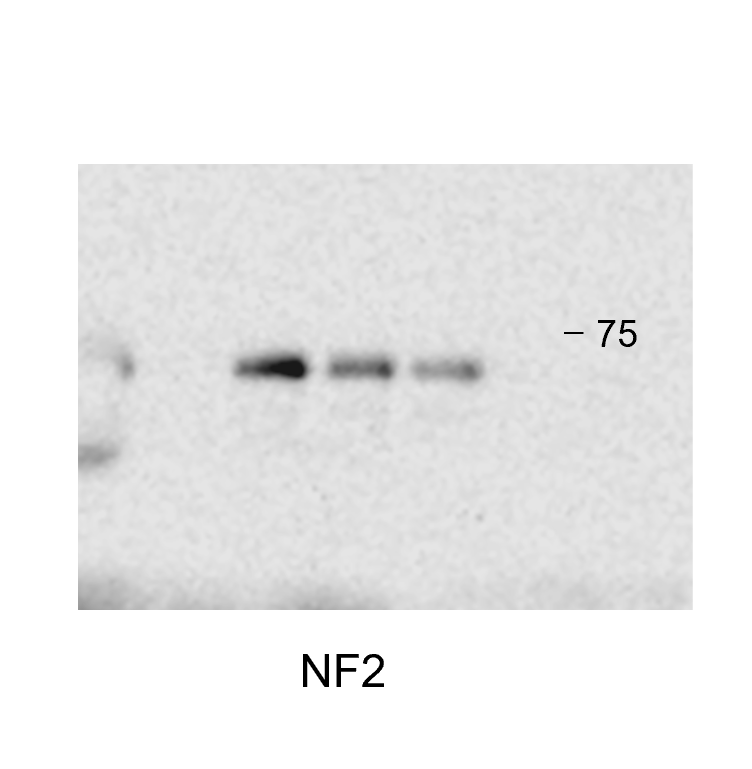

Supplement: Supplementary file 7 — Source data Fig. 5 [file 44319_2024_228_MOESM7_ESM.zip › Figure 5/Figure 5E/NF2.tif]

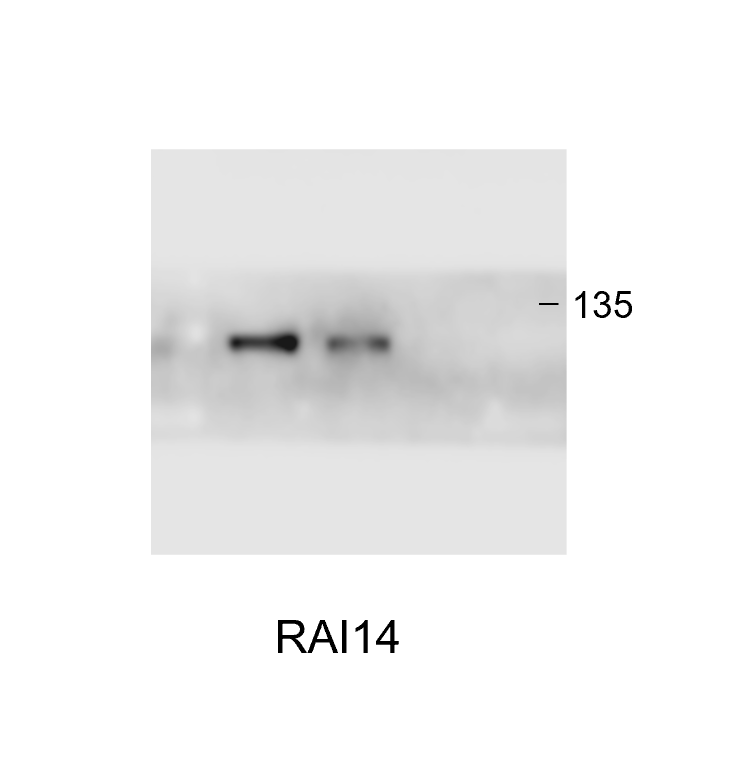

Supplement: Supplementary file 7 — Source data Fig. 5 [file 44319_2024_228_MOESM7_ESM.zip › Figure 5/Figure 5E/RAI14.tif]

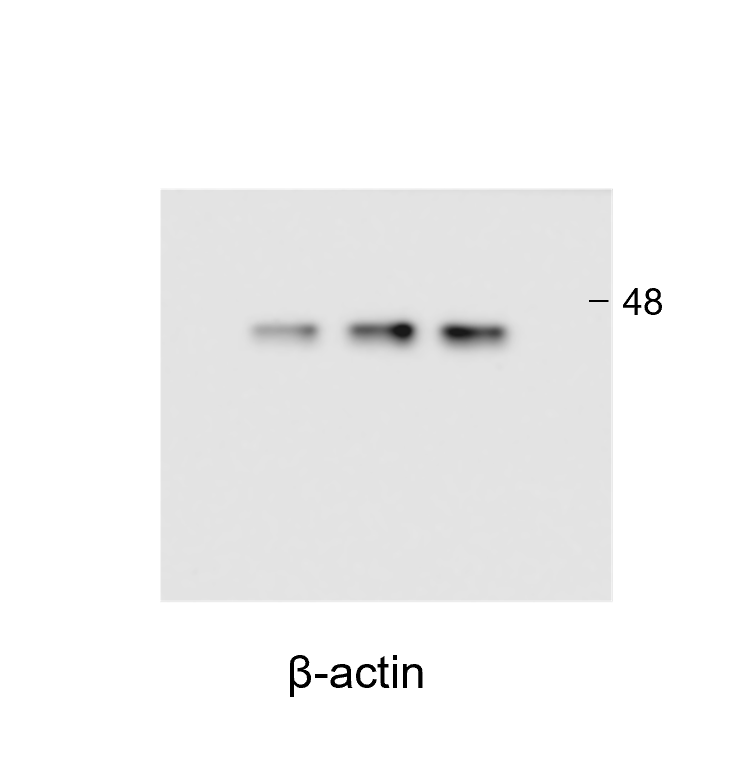

Supplement: Supplementary file 7 — Source data Fig. 5 [file 44319_2024_228_MOESM7_ESM.zip › Figure 5/Figure 5E/ÑΓ-actin.tif]

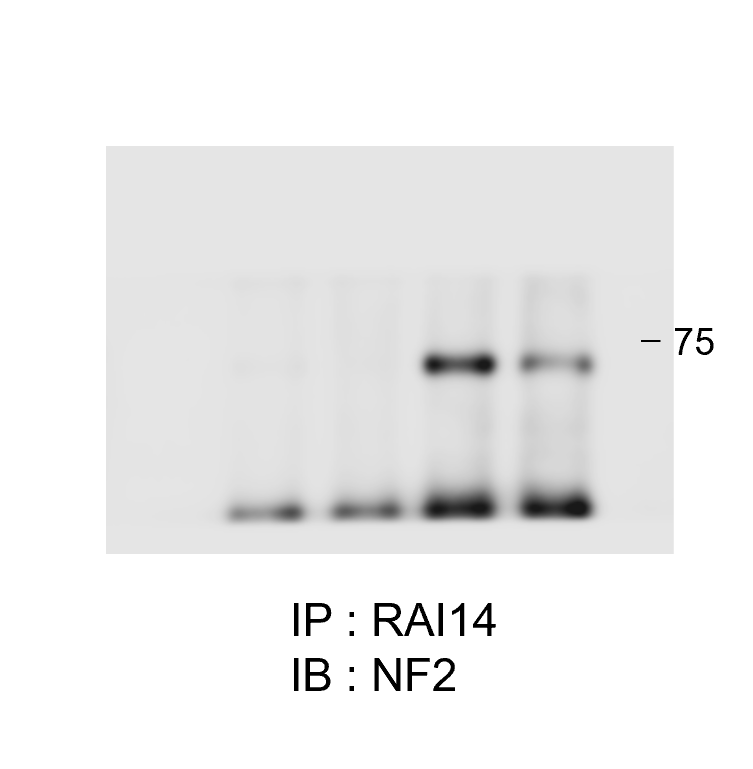

Supplement: Supplementary file 8 — Source data Fig. 6 [file 44319_2024_228_MOESM8_ESM.zip › Figure 6/Figure 6B/IP RAI14, IB NF2.tif]

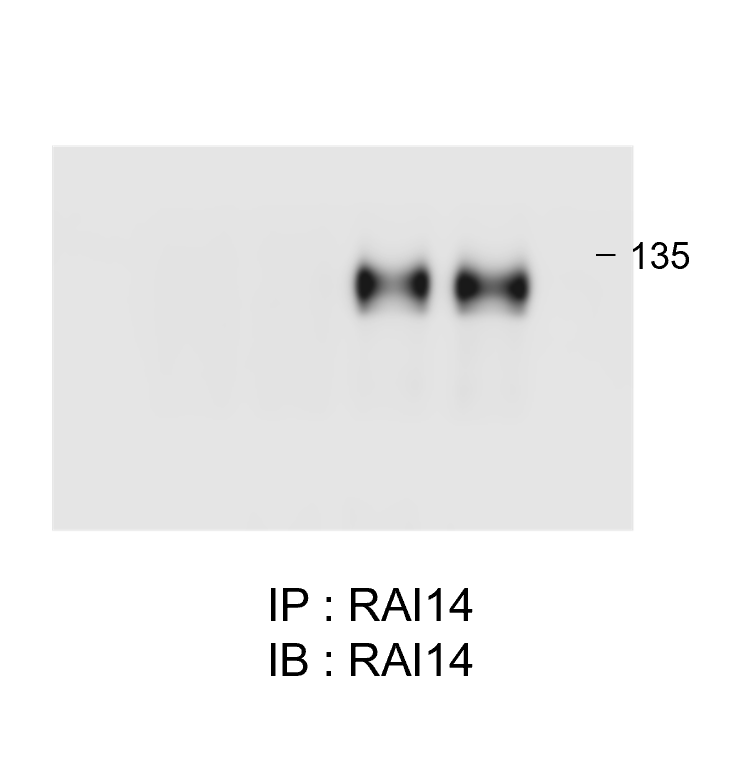

Supplement: Supplementary file 8 — Source data Fig. 6 [file 44319_2024_228_MOESM8_ESM.zip › Figure 6/Figure 6B/IP RAI14, IB RAI14.tif]

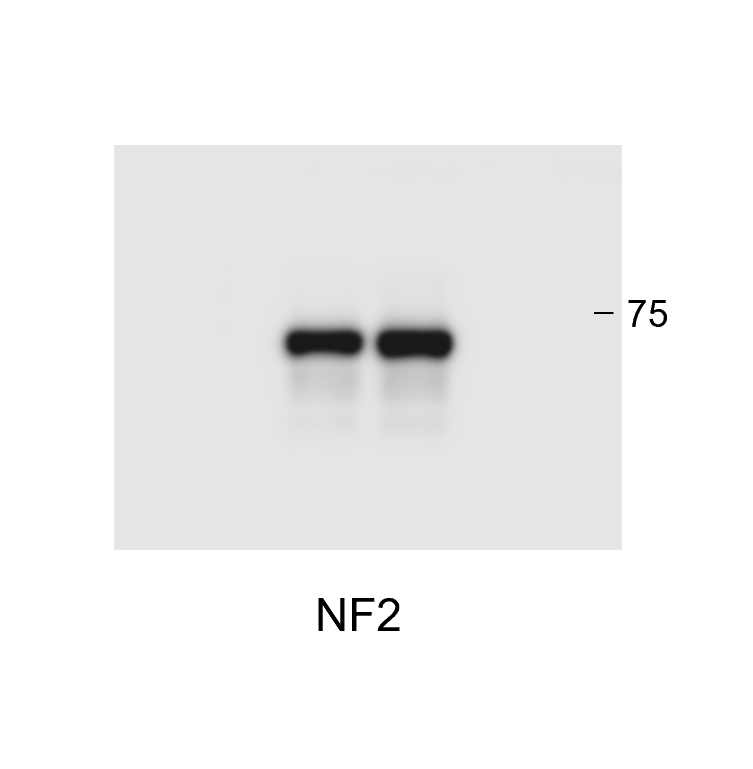

Supplement: Supplementary file 8 — Source data Fig. 6 [file 44319_2024_228_MOESM8_ESM.zip › Figure 6/Figure 6B/NF2.tif]

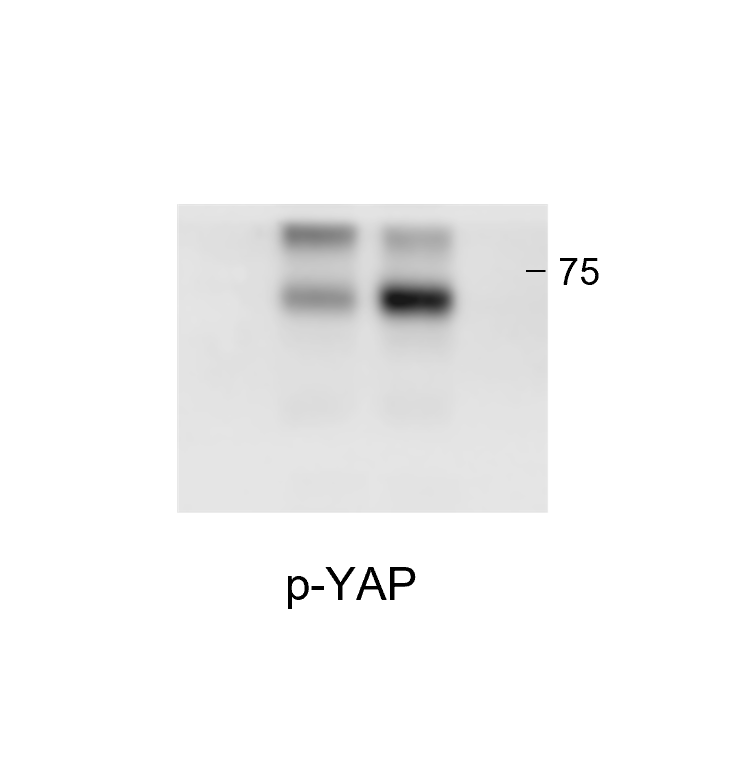

Supplement: Supplementary file 8 — Source data Fig. 6 [file 44319_2024_228_MOESM8_ESM.zip › Figure 6/Figure 6B/p-YAP.tif]

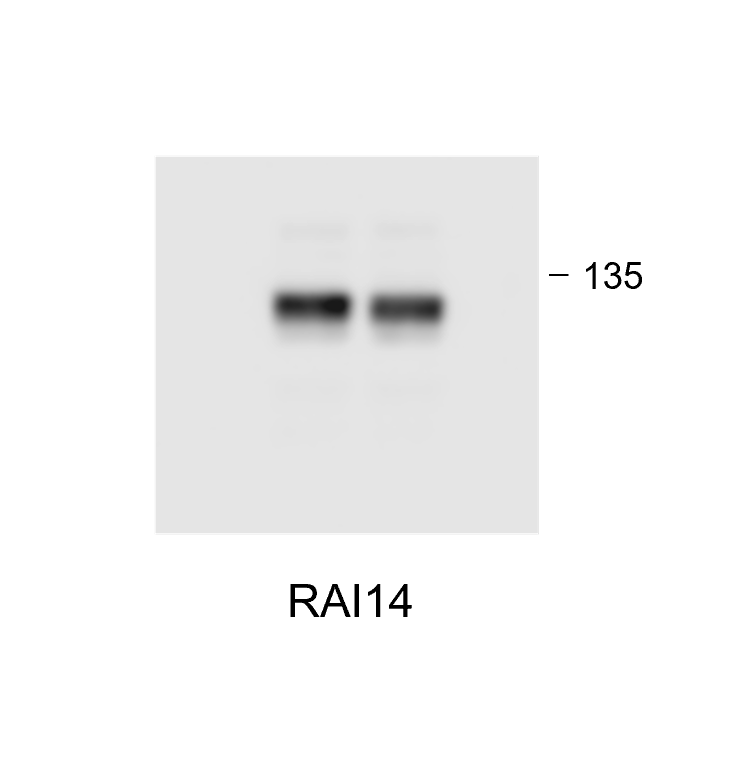

Supplement: Supplementary file 8 — Source data Fig. 6 [file 44319_2024_228_MOESM8_ESM.zip › Figure 6/Figure 6B/RAI14.tif]

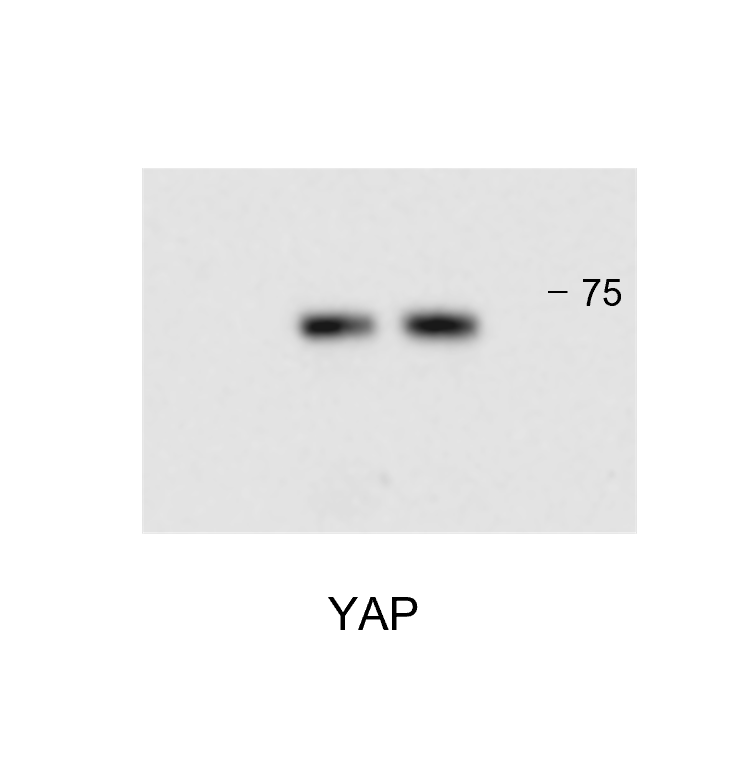

Supplement: Supplementary file 8 — Source data Fig. 6 [file 44319_2024_228_MOESM8_ESM.zip › Figure 6/Figure 6B/YAP.tif]

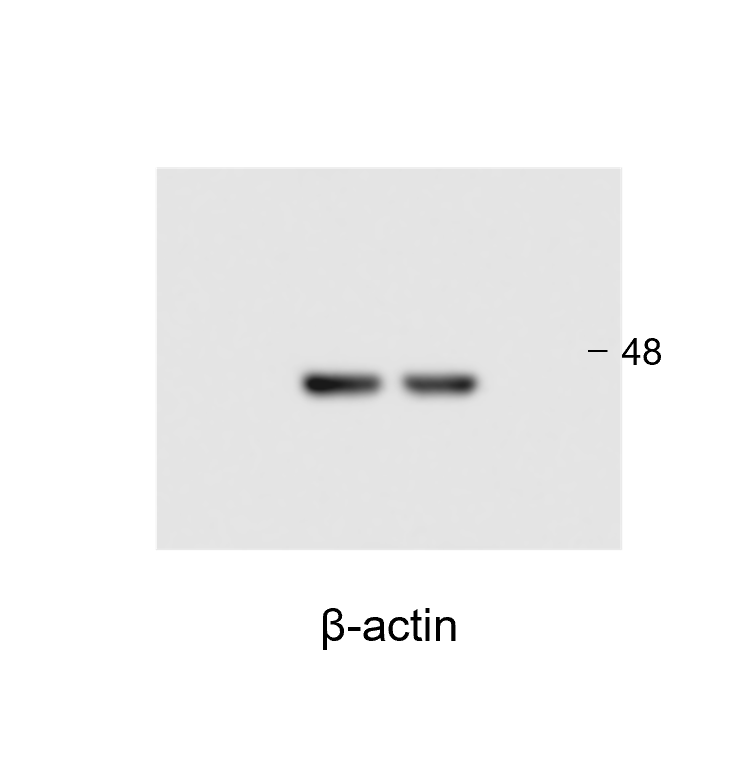

Supplement: Supplementary file 8 — Source data Fig. 6 [file 44319_2024_228_MOESM8_ESM.zip › Figure 6/Figure 6B/ÑΓ-actin.tif]

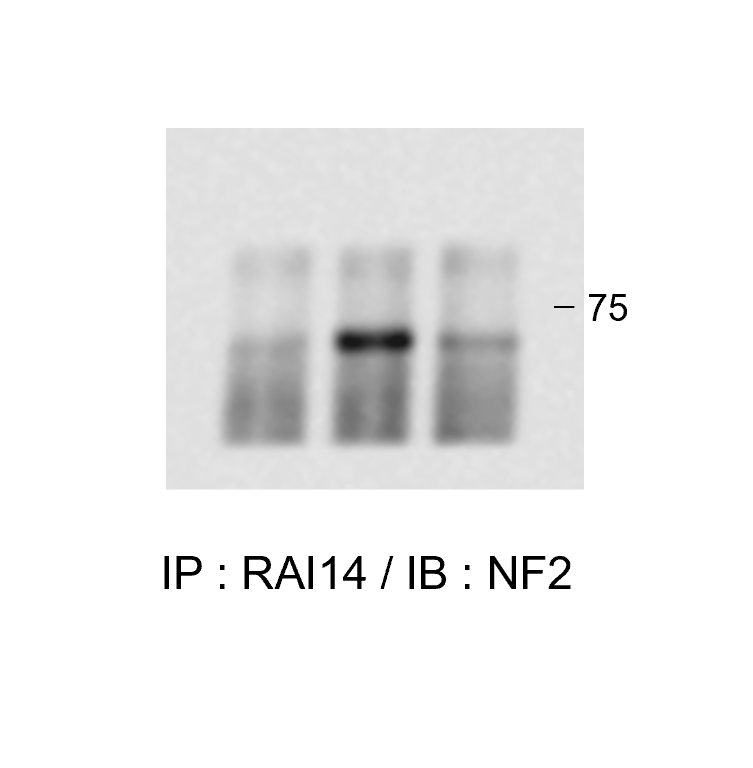

Supplement: Supplementary file 8 — Source data Fig. 6 [file 44319_2024_228_MOESM8_ESM.zip › Figure 6/Figure 6C/IP RAI14, IB NF2.tif]

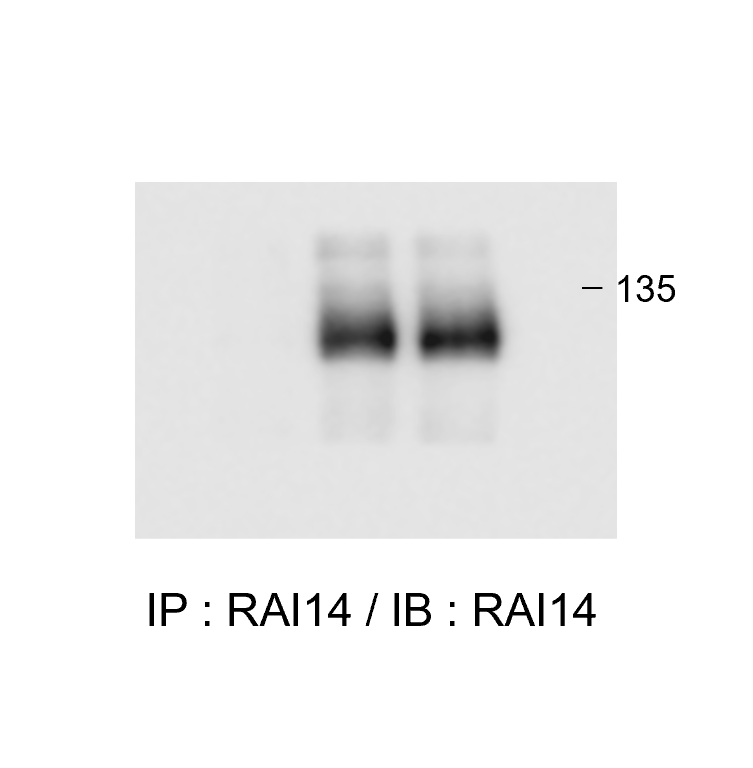

Supplement: Supplementary file 8 — Source data Fig. 6 [file 44319_2024_228_MOESM8_ESM.zip › Figure 6/Figure 6C/IP RAI14, IB RAI14.tif]

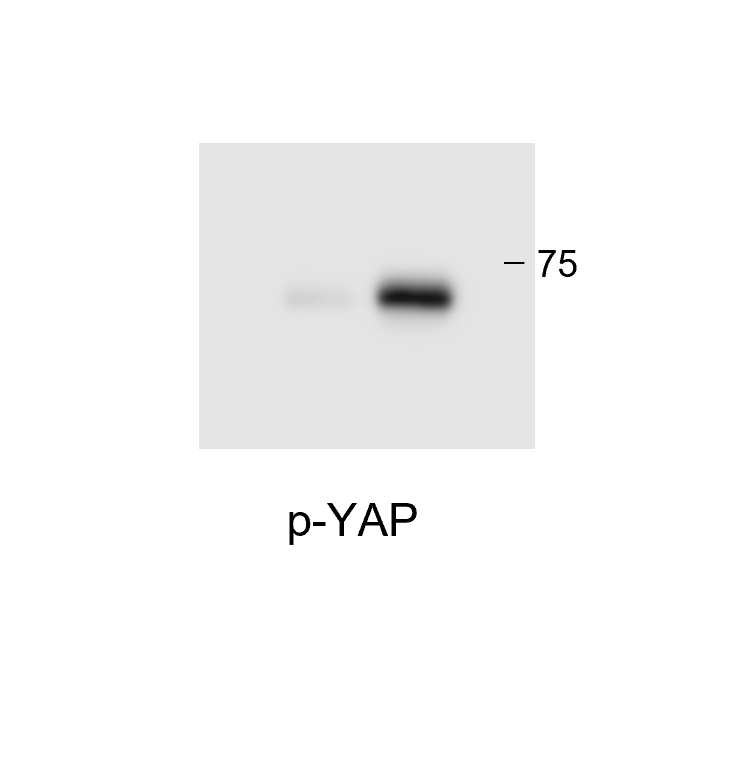

Supplement: Supplementary file 8 — Source data Fig. 6 [file 44319_2024_228_MOESM8_ESM.zip › Figure 6/Figure 6C/p-YAP.tif]

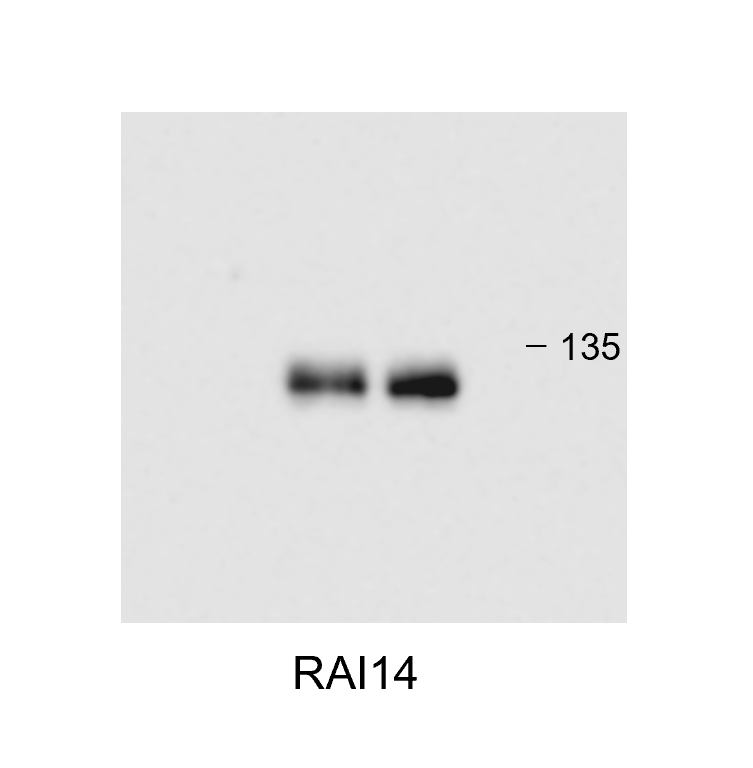

Supplement: Supplementary file 8 — Source data Fig. 6 [file 44319_2024_228_MOESM8_ESM.zip › Figure 6/Figure 6C/RAI14.tif]

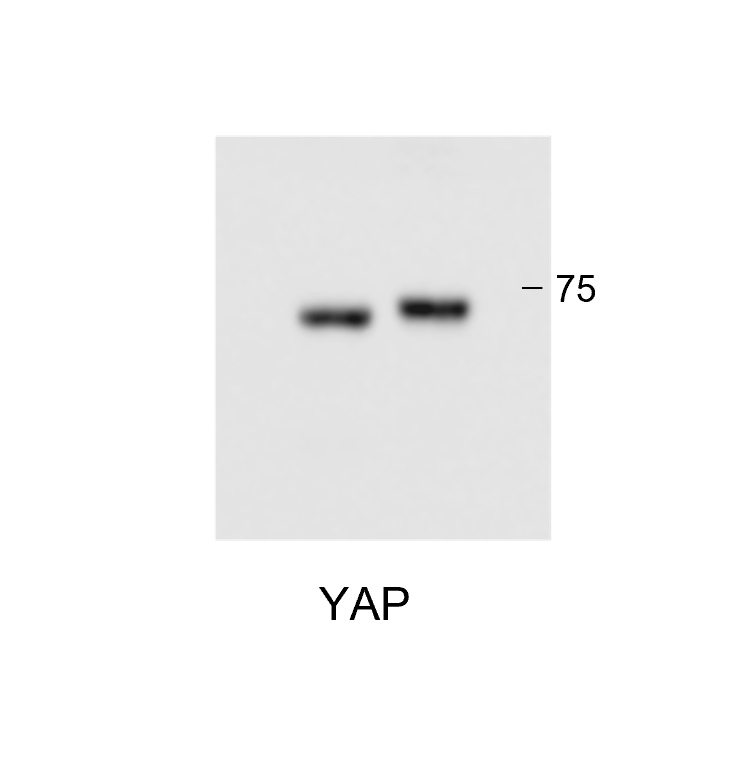

Supplement: Supplementary file 8 — Source data Fig. 6 [file 44319_2024_228_MOESM8_ESM.zip › Figure 6/Figure 6C/YAP.tif]

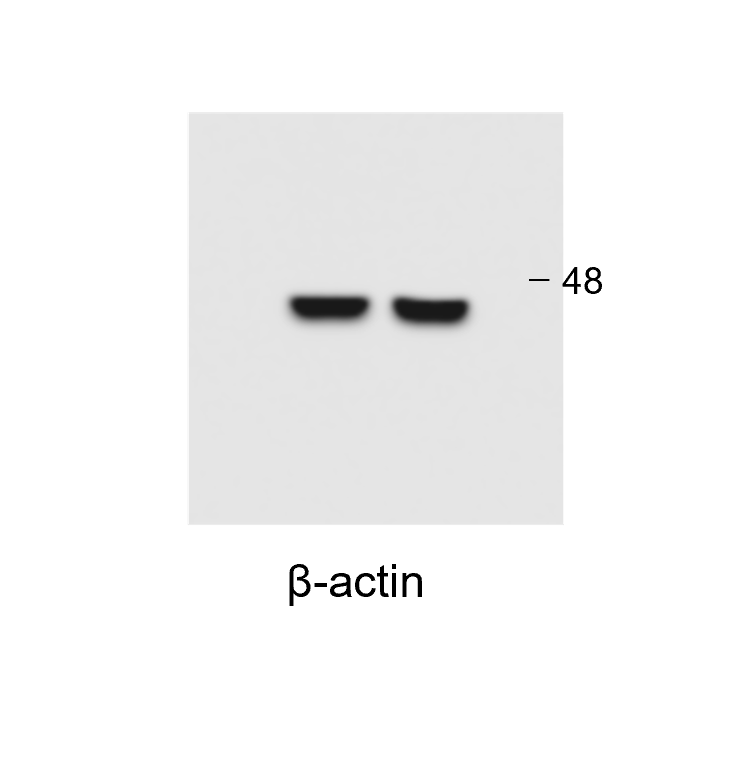

Supplement: Supplementary file 8 — Source data Fig. 6 [file 44319_2024_228_MOESM8_ESM.zip › Figure 6/Figure 6C/ÑΓ-actin.tif]

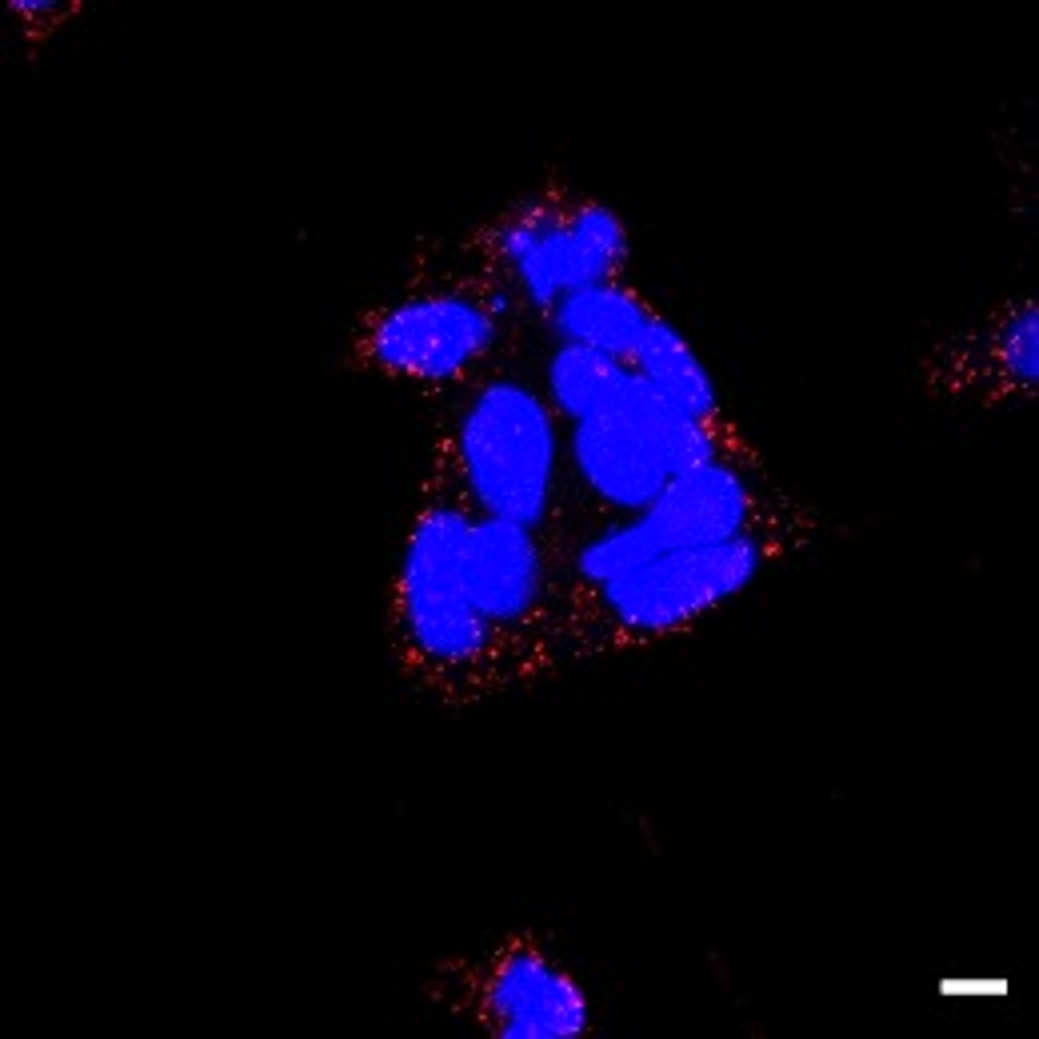

Supplement: Supplementary file 8 — Source data Fig. 6 [file 44319_2024_228_MOESM8_ESM.zip › Figure 6/Figure 6D/1 kPa.tif]

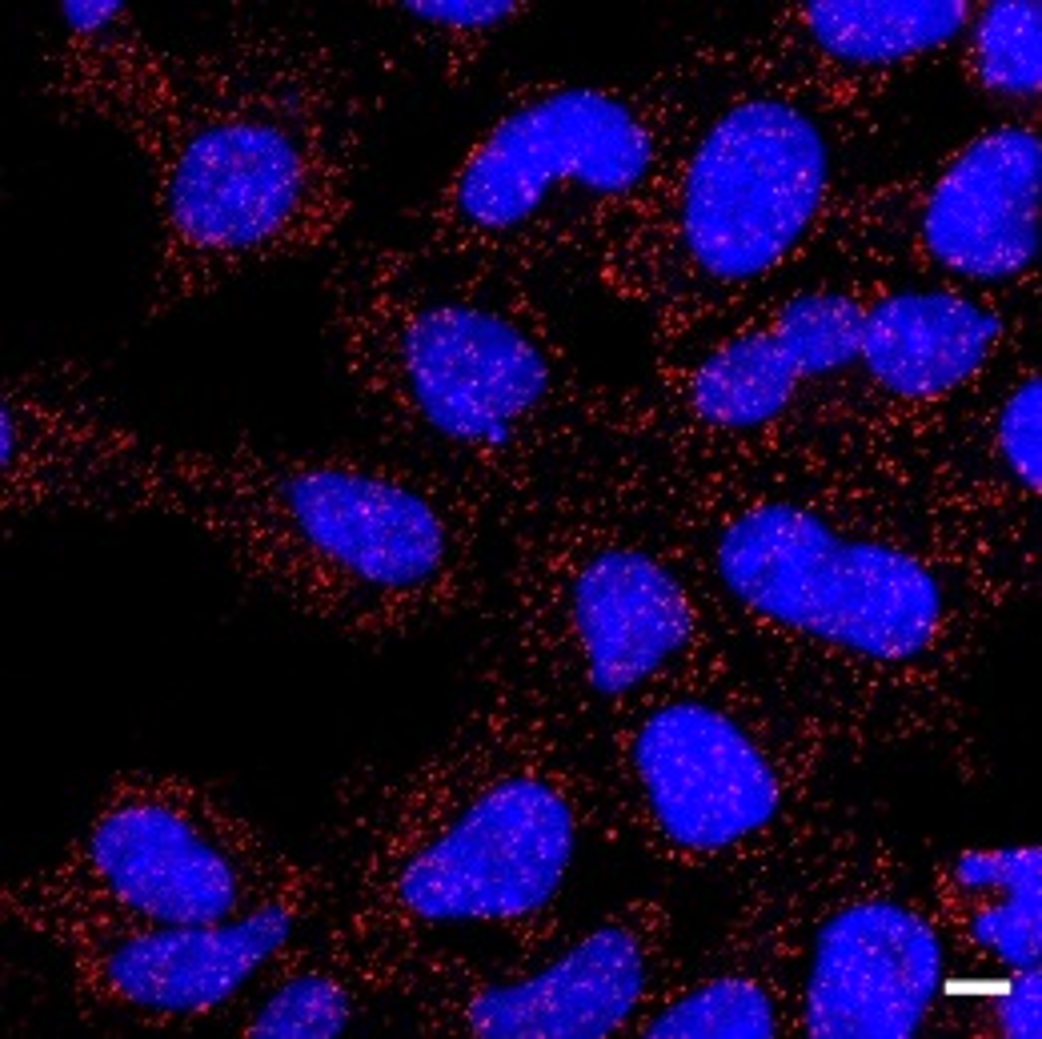

Supplement: Supplementary file 8 — Source data Fig. 6 [file 44319_2024_228_MOESM8_ESM.zip › Figure 6/Figure 6D/50 kPa.tif]

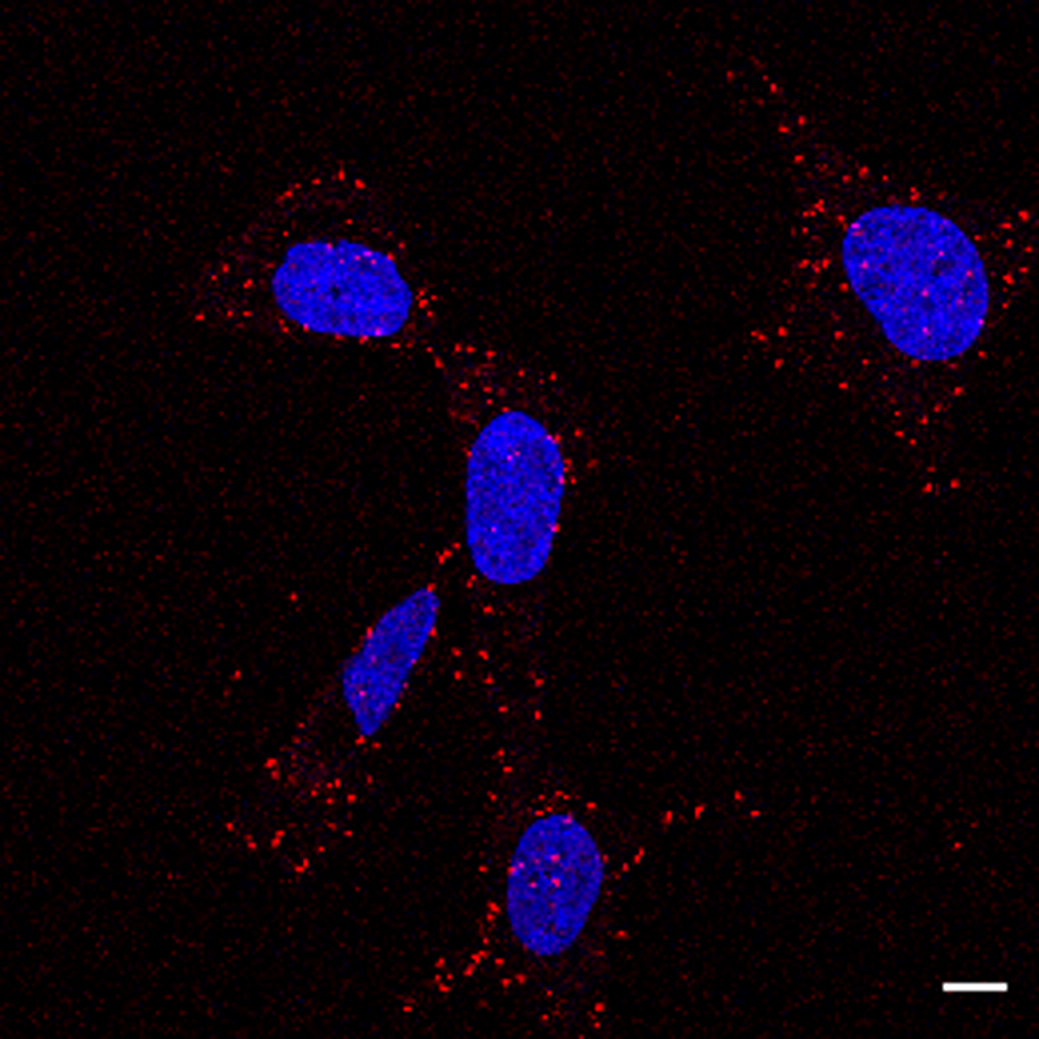

Supplement: Supplementary file 8 — Source data Fig. 6 [file 44319_2024_228_MOESM8_ESM.zip › Figure 6/Figure 6E/DMSO.tif]

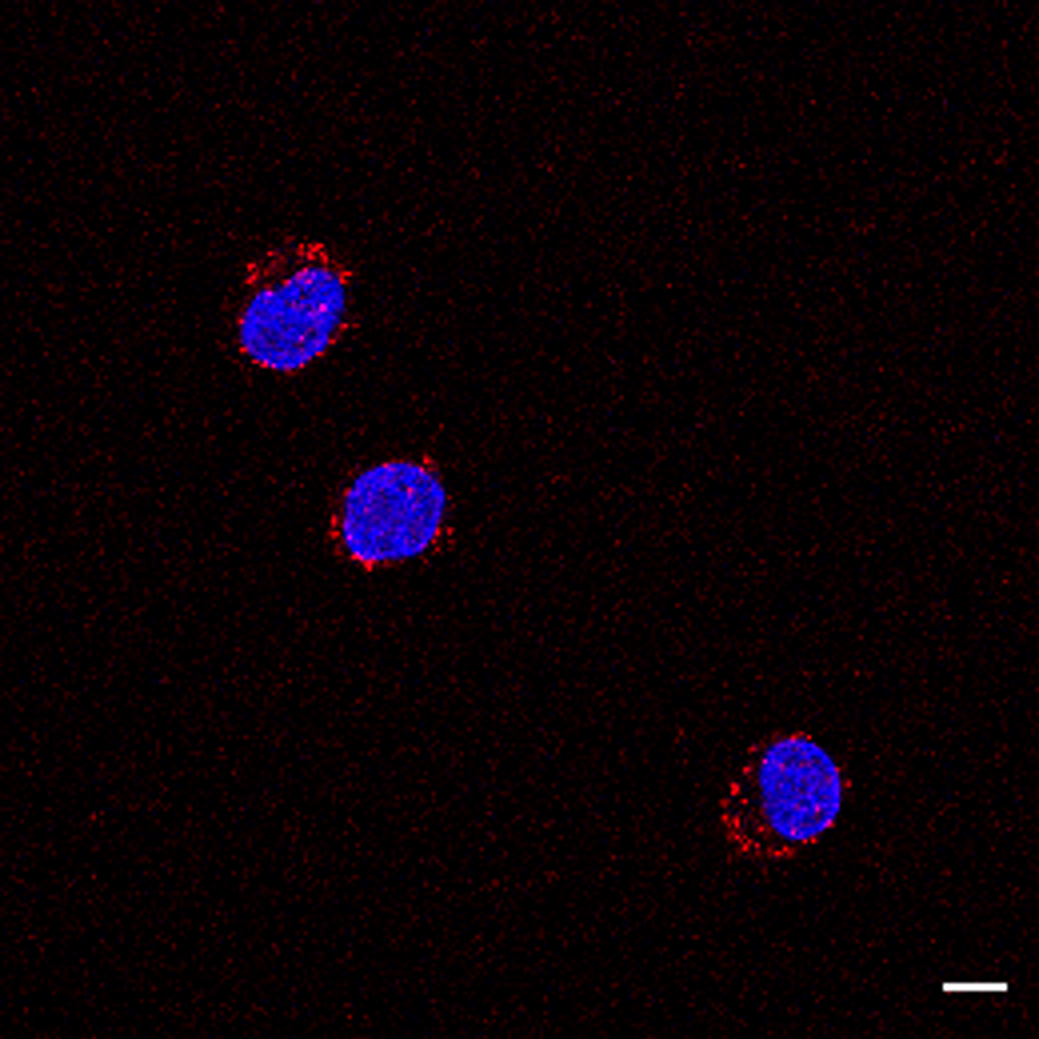

Supplement: Supplementary file 8 — Source data Fig. 6 [file 44319_2024_228_MOESM8_ESM.zip › Figure 6/Figure 6E/Lat. B(1h).tif]

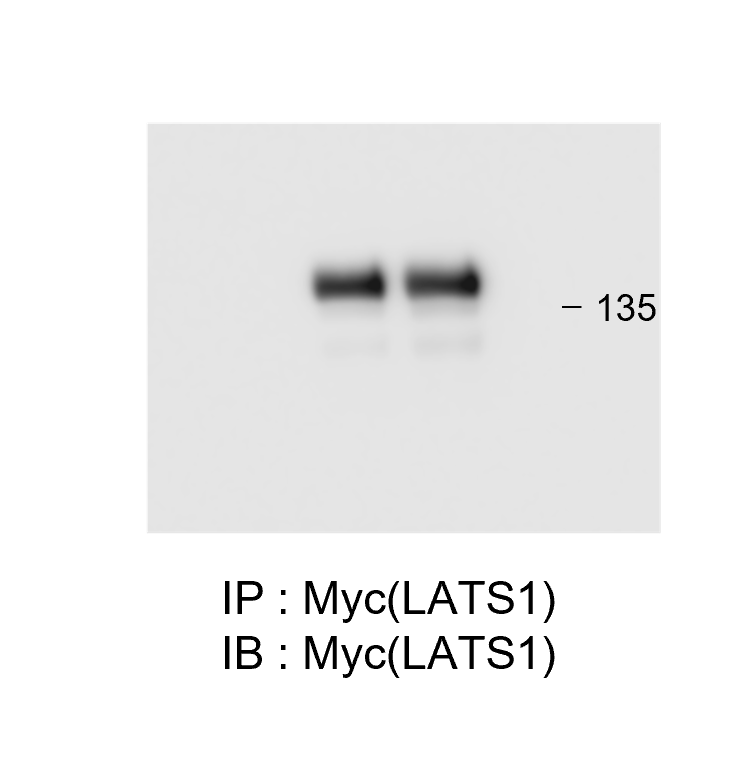

Supplement: Supplementary file 8 — Source data Fig. 6 [file 44319_2024_228_MOESM8_ESM.zip › Figure 6/Figure 6F/IP Myc(LATS1), IB Myc(LATS1).tif]

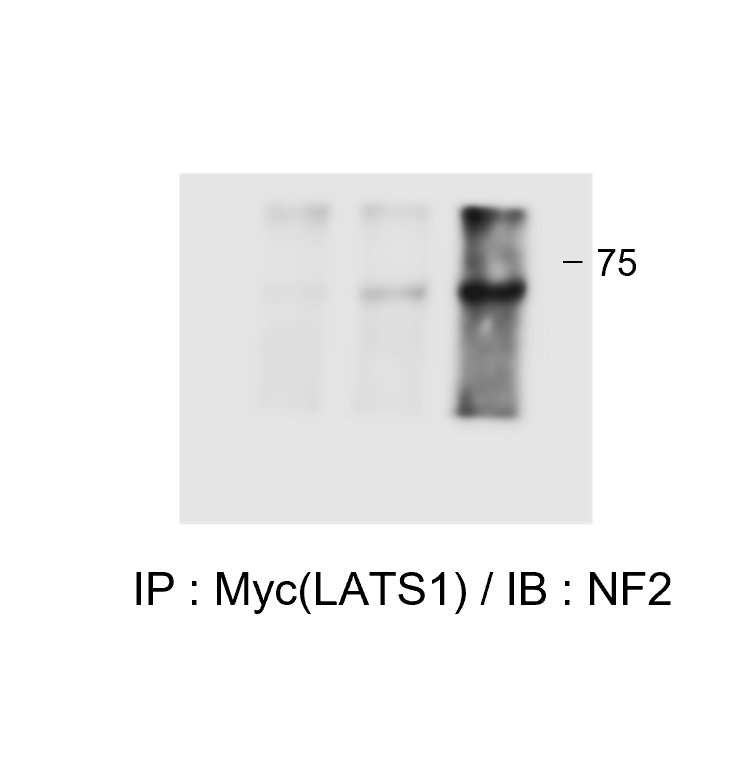

Supplement: Supplementary file 8 — Source data Fig. 6 [file 44319_2024_228_MOESM8_ESM.zip › Figure 6/Figure 6F/IP Myc(LATS1), IB NF2.tif]

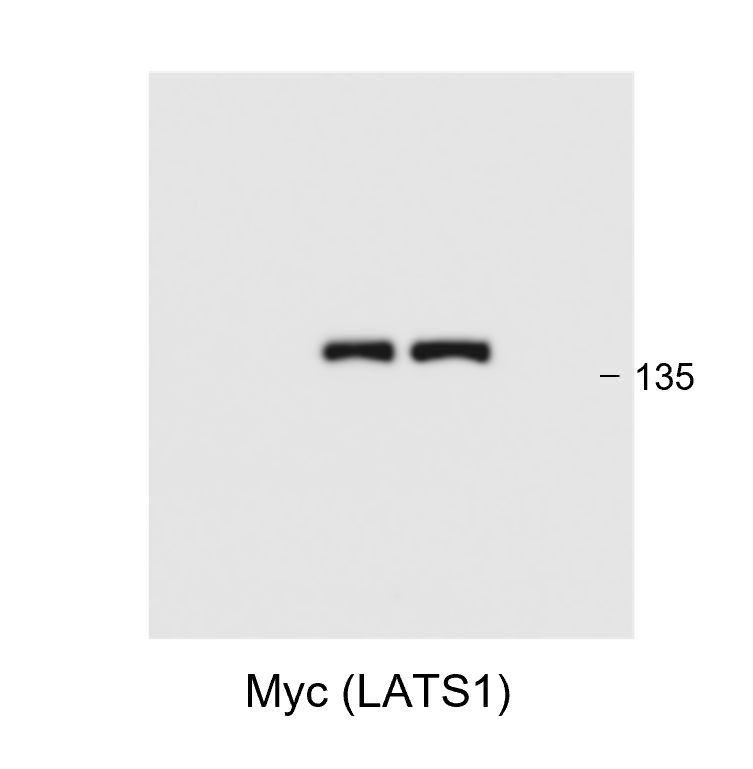

Supplement: Supplementary file 8 — Source data Fig. 6 [file 44319_2024_228_MOESM8_ESM.zip › Figure 6/Figure 6F/Myc(LATS1).tif]

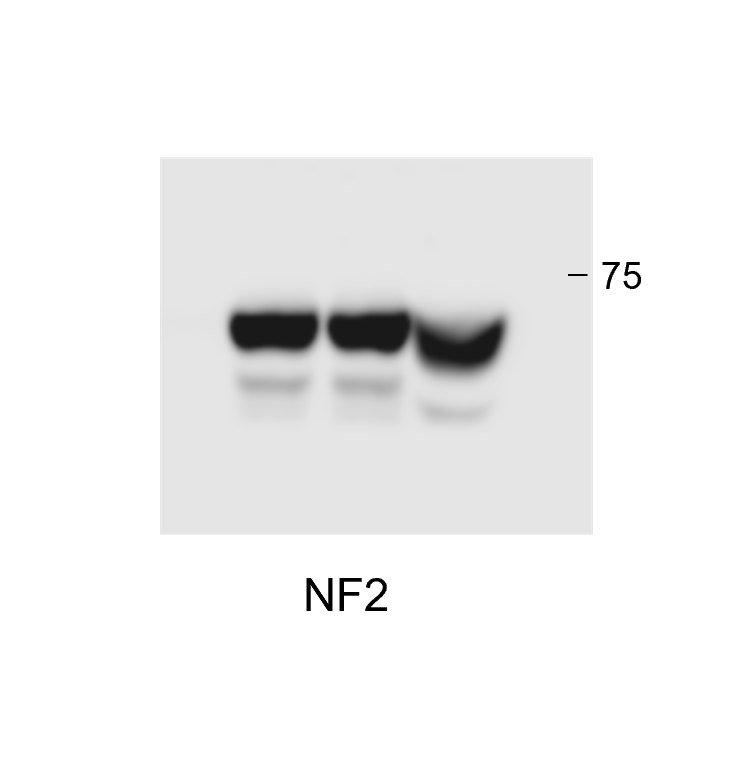

Supplement: Supplementary file 8 — Source data Fig. 6 [file 44319_2024_228_MOESM8_ESM.zip › Figure 6/Figure 6F/NF2.tif]

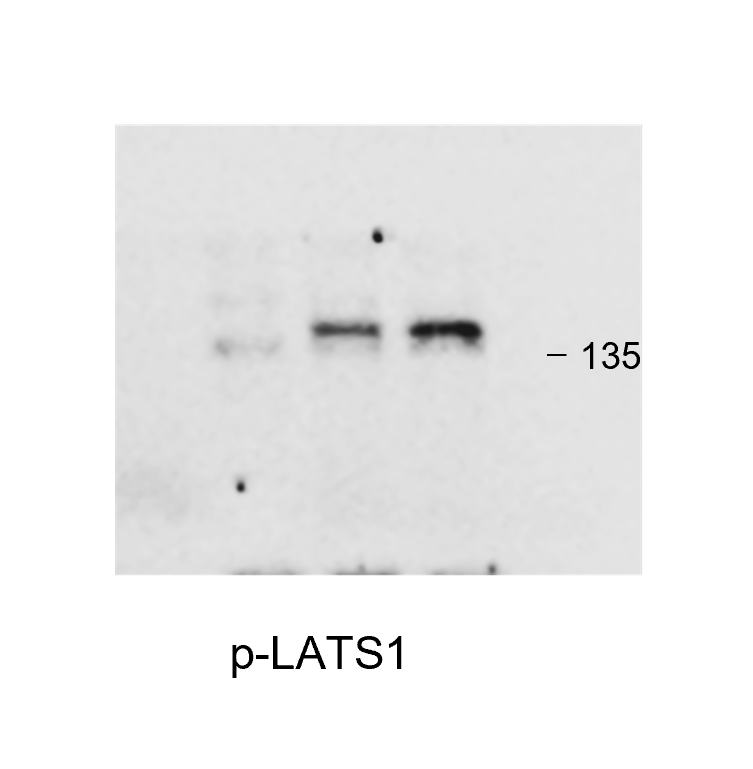

Supplement: Supplementary file 8 — Source data Fig. 6 [file 44319_2024_228_MOESM8_ESM.zip › Figure 6/Figure 6F/p-LATS1.tif]

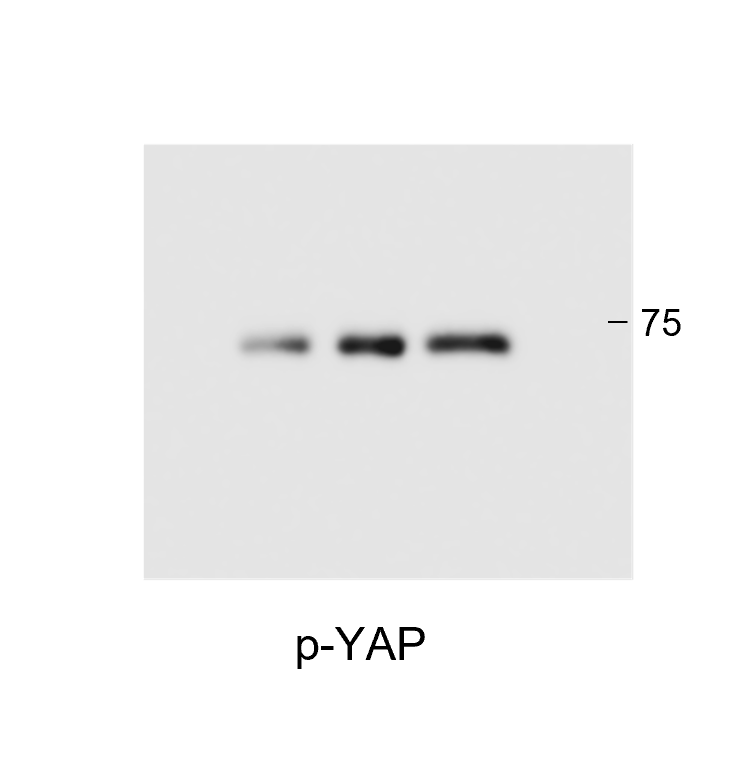

Supplement: Supplementary file 8 — Source data Fig. 6 [file 44319_2024_228_MOESM8_ESM.zip › Figure 6/Figure 6F/p-YAP.tif]

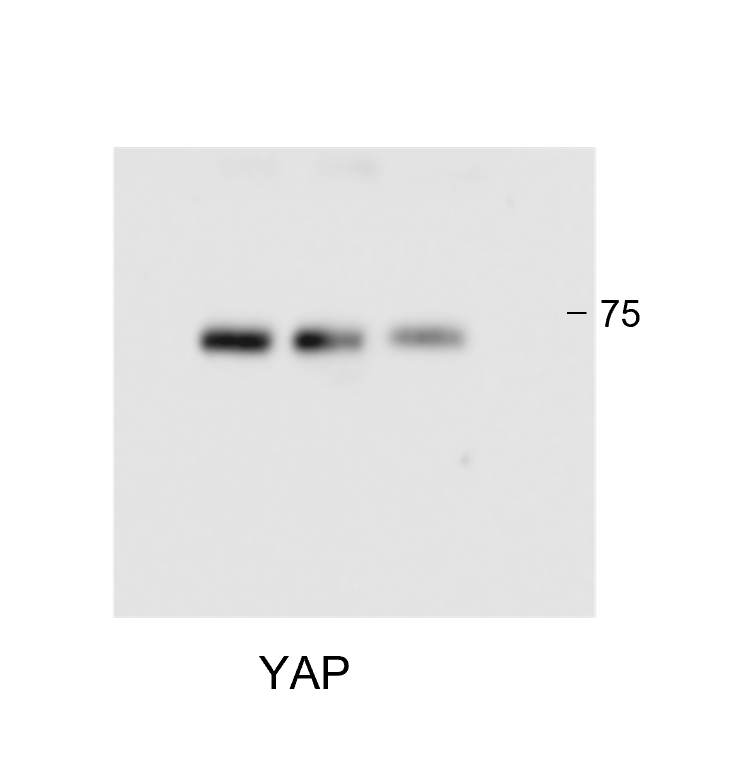

Supplement: Supplementary file 8 — Source data Fig. 6 [file 44319_2024_228_MOESM8_ESM.zip › Figure 6/Figure 6F/YAP.tif]

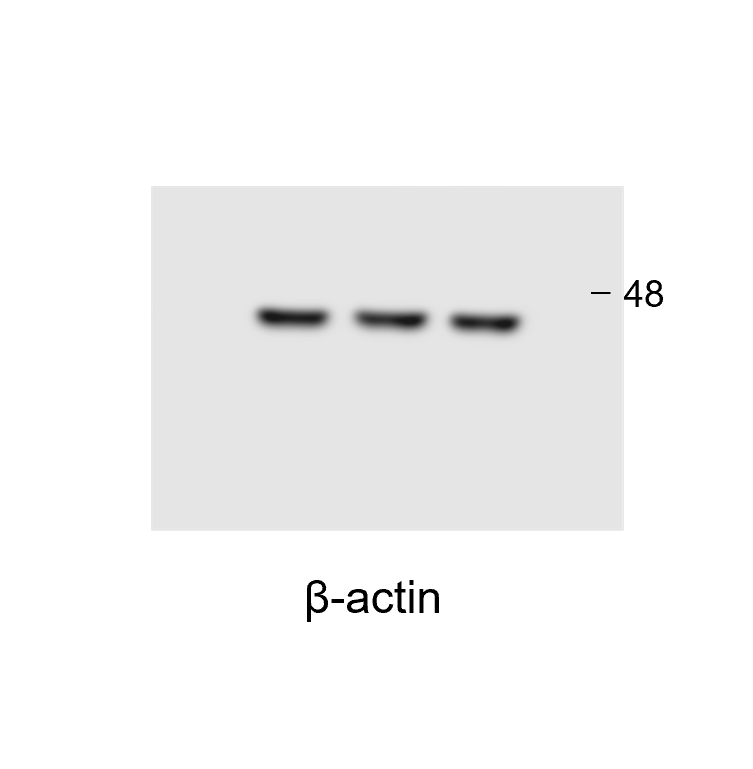

Supplement: Supplementary file 8 — Source data Fig. 6 [file 44319_2024_228_MOESM8_ESM.zip › Figure 6/Figure 6F/ÑΓ-actin.tif]

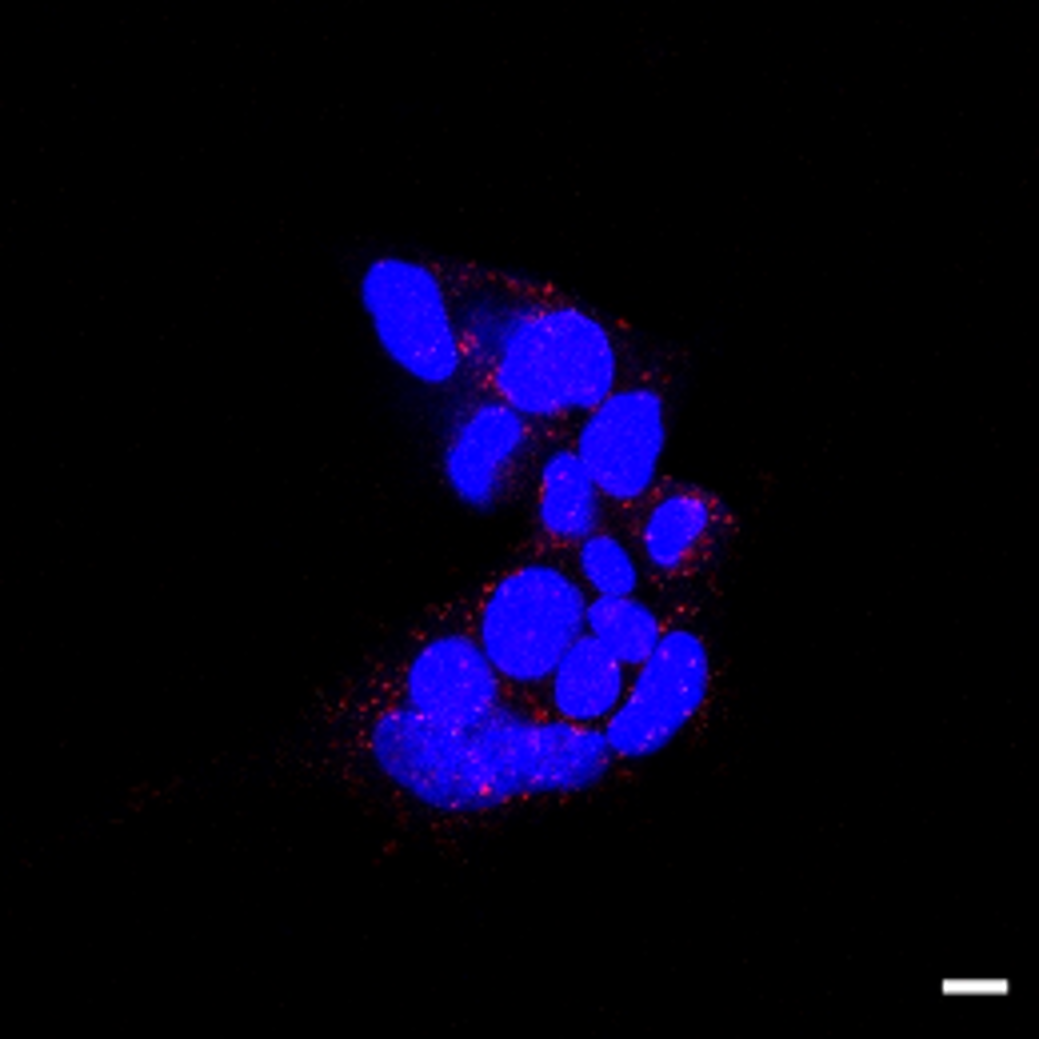

Supplement: Supplementary file 8 — Source data Fig. 6 [file 44319_2024_228_MOESM8_ESM.zip › Figure 6/Figure 6G/1 kPa.tif]

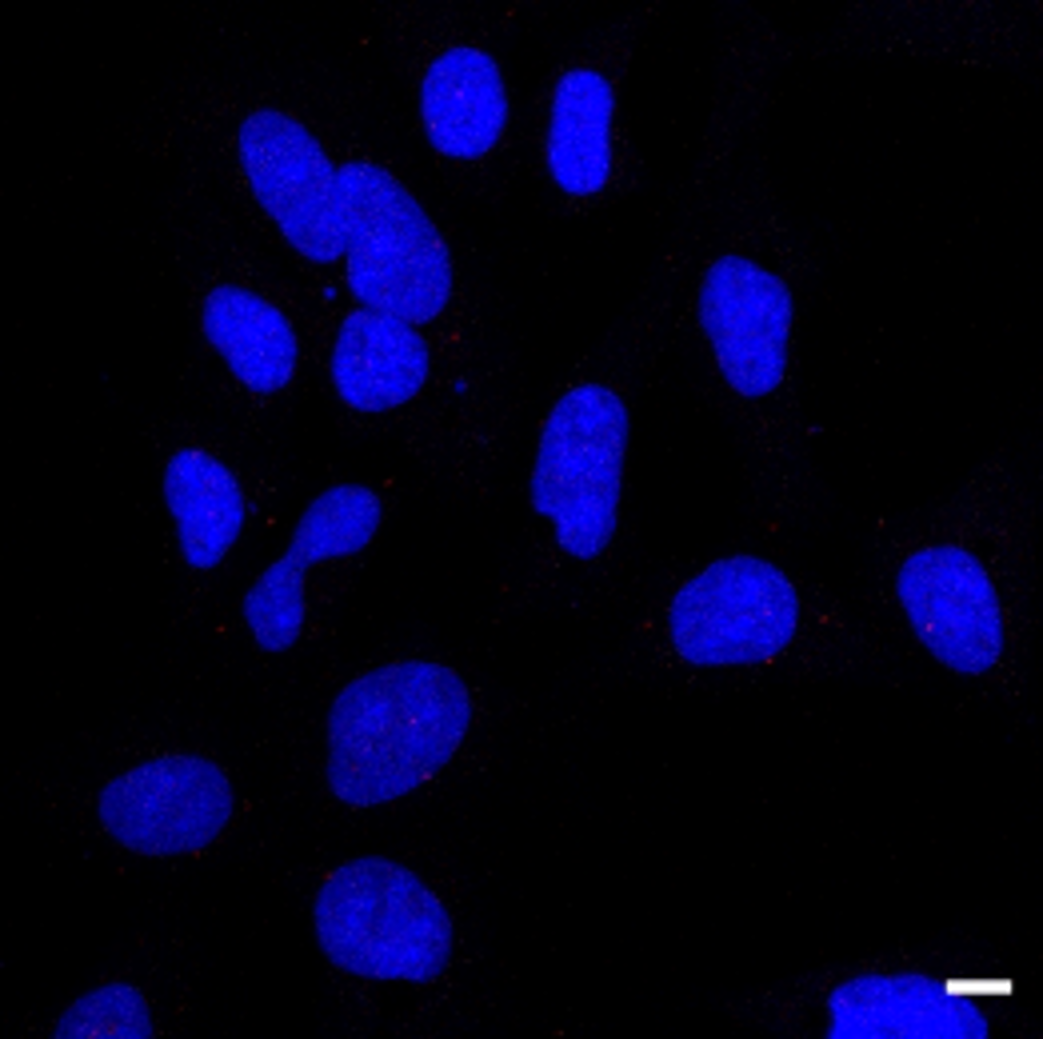

Supplement: Supplementary file 8 — Source data Fig. 6 [file 44319_2024_228_MOESM8_ESM.zip › Figure 6/Figure 6G/50 kPa.tif]

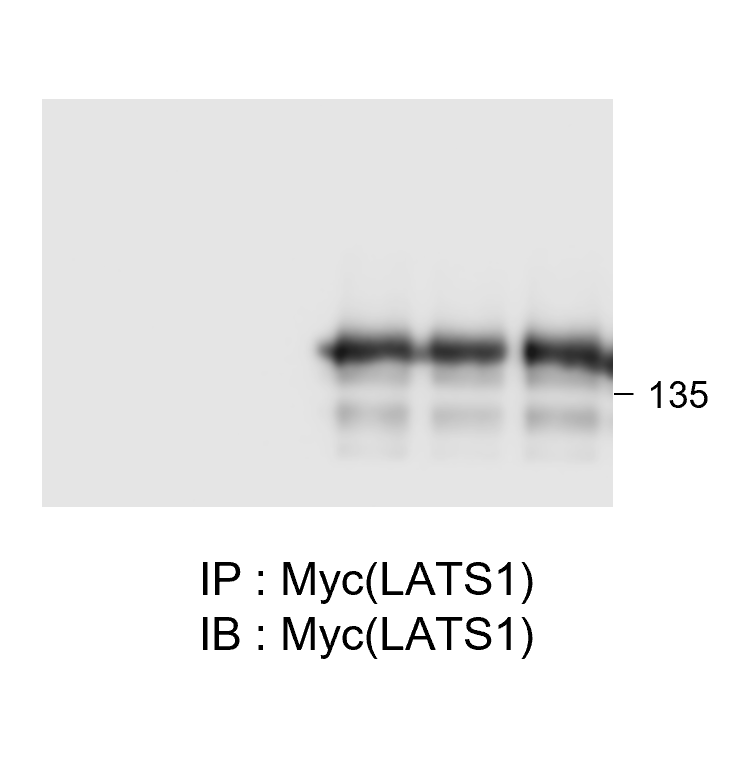

Supplement: Supplementary file 8 — Source data Fig. 6 [file 44319_2024_228_MOESM8_ESM.zip › Figure 6/Figure 6H/IP Myc(LATS1), IB Myc(LATS1).tif]

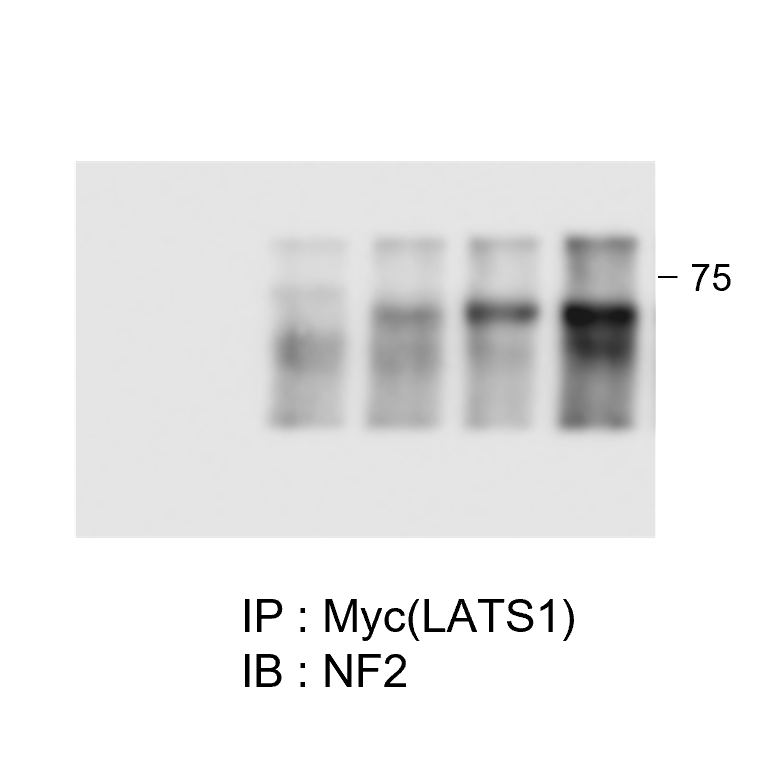

Supplement: Supplementary file 8 — Source data Fig. 6 [file 44319_2024_228_MOESM8_ESM.zip › Figure 6/Figure 6H/IP Myc(LATS1), IB NF2.tif]

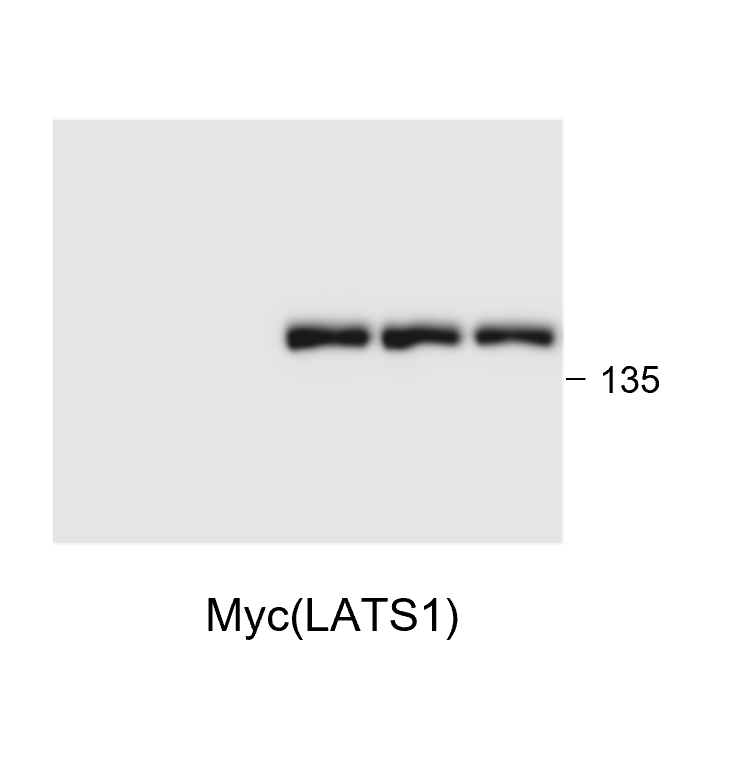

Supplement: Supplementary file 8 — Source data Fig. 6 [file 44319_2024_228_MOESM8_ESM.zip › Figure 6/Figure 6H/Myc(LATS1).tif]

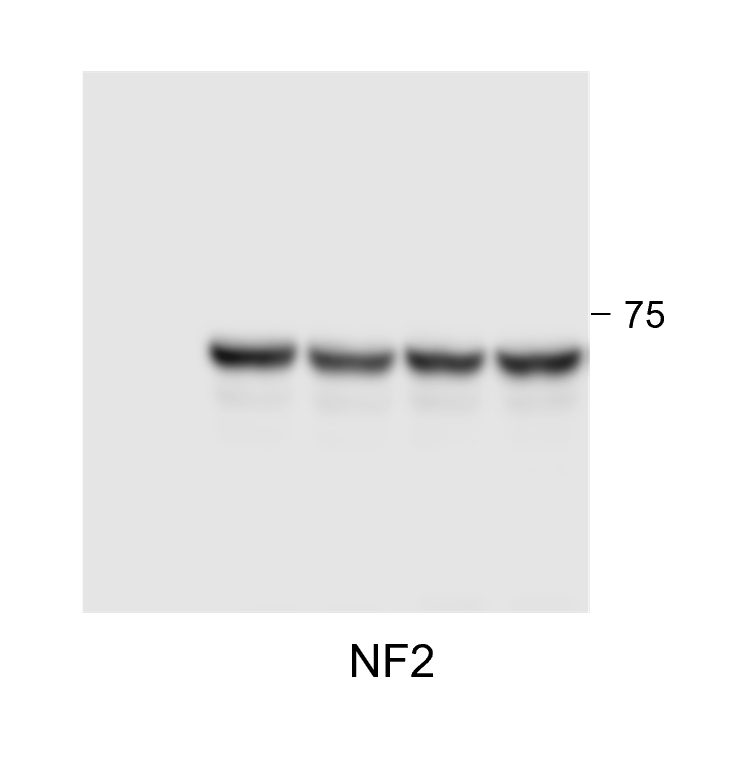

Supplement: Supplementary file 8 — Source data Fig. 6 [file 44319_2024_228_MOESM8_ESM.zip › Figure 6/Figure 6H/NF2.tif]

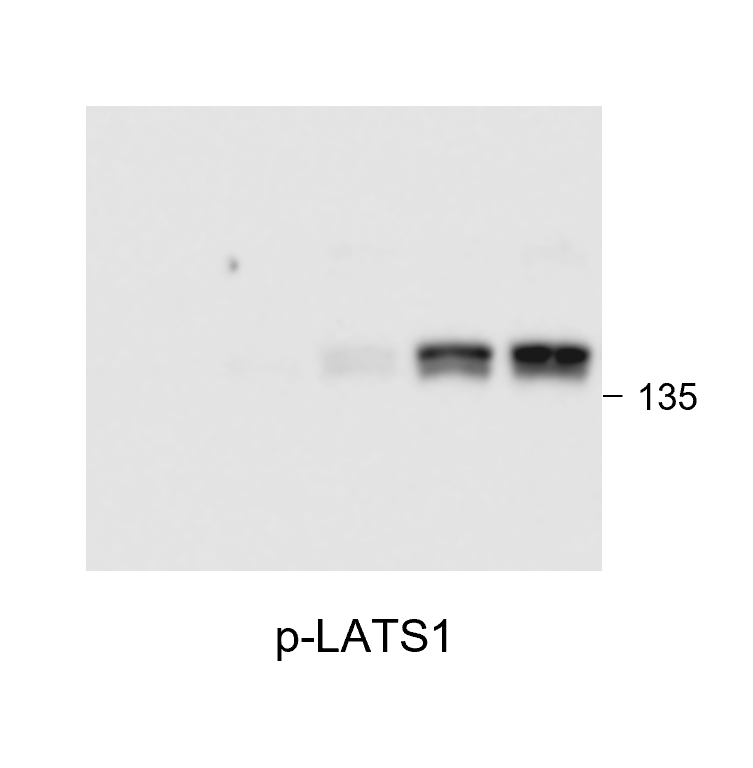

Supplement: Supplementary file 8 — Source data Fig. 6 [file 44319_2024_228_MOESM8_ESM.zip › Figure 6/Figure 6H/p-LATS1.tif]

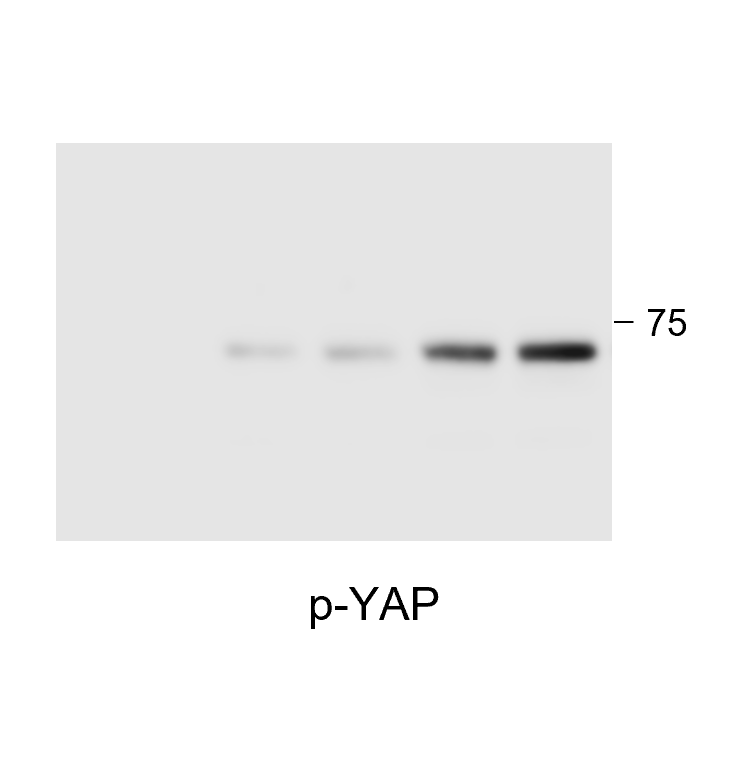

Supplement: Supplementary file 8 — Source data Fig. 6 [file 44319_2024_228_MOESM8_ESM.zip › Figure 6/Figure 6H/p-YAP.tif]

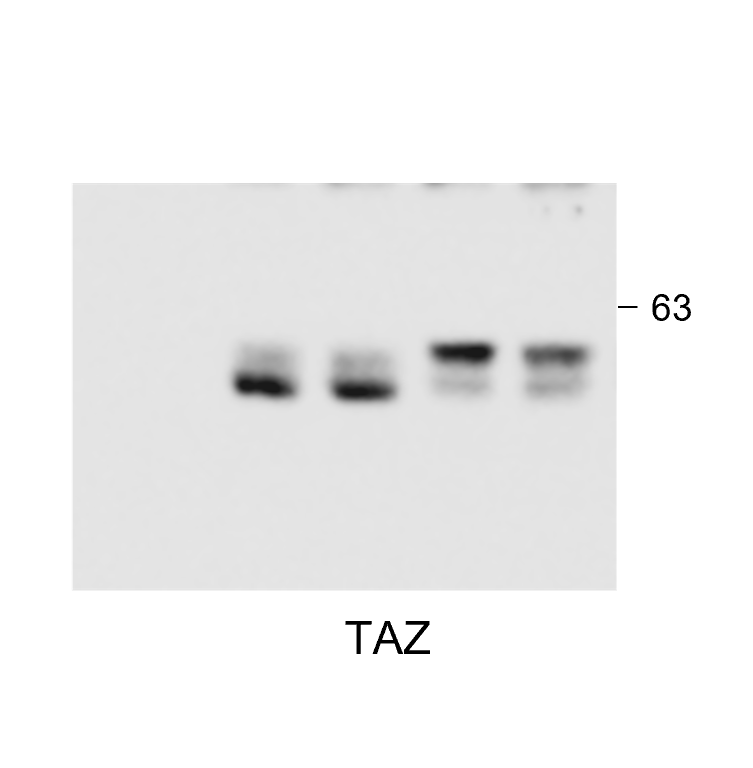

Supplement: Supplementary file 8 — Source data Fig. 6 [file 44319_2024_228_MOESM8_ESM.zip › Figure 6/Figure 6H/TAZ.tif]

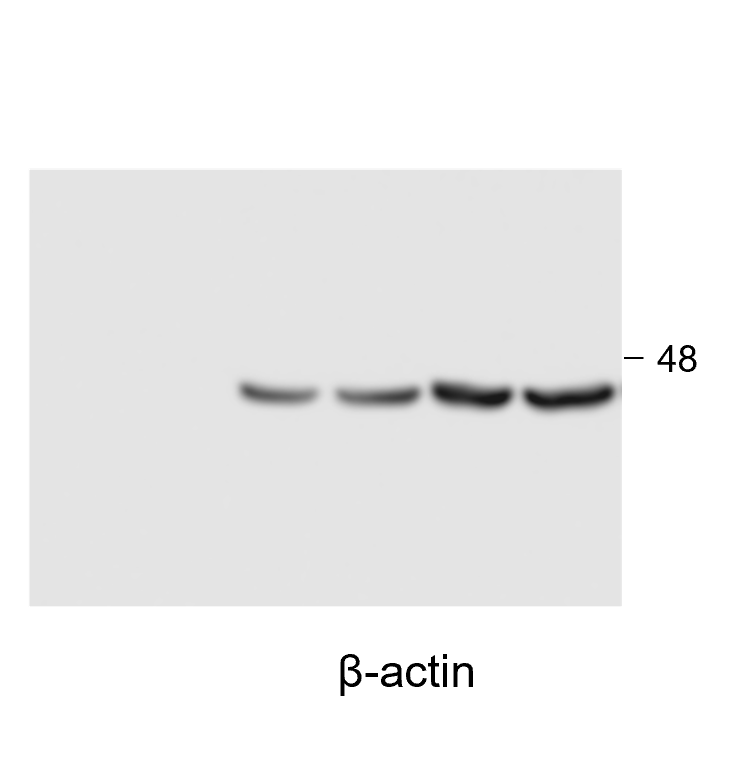

Supplement: Supplementary file 8 — Source data Fig. 6 [file 44319_2024_228_MOESM8_ESM.zip › Figure 6/Figure 6H/ÑΓ-actin.tif]

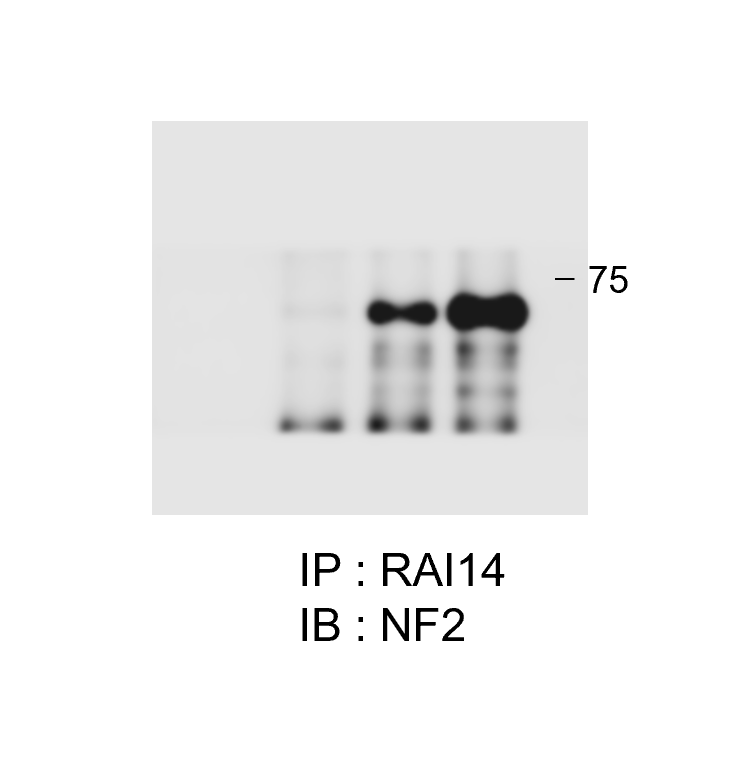

Supplement: Supplementary file 8 — Source data Fig. 6 [file 44319_2024_228_MOESM8_ESM.zip › Figure 6/Figure 6I/IP RAI14, IB NF2.tif]

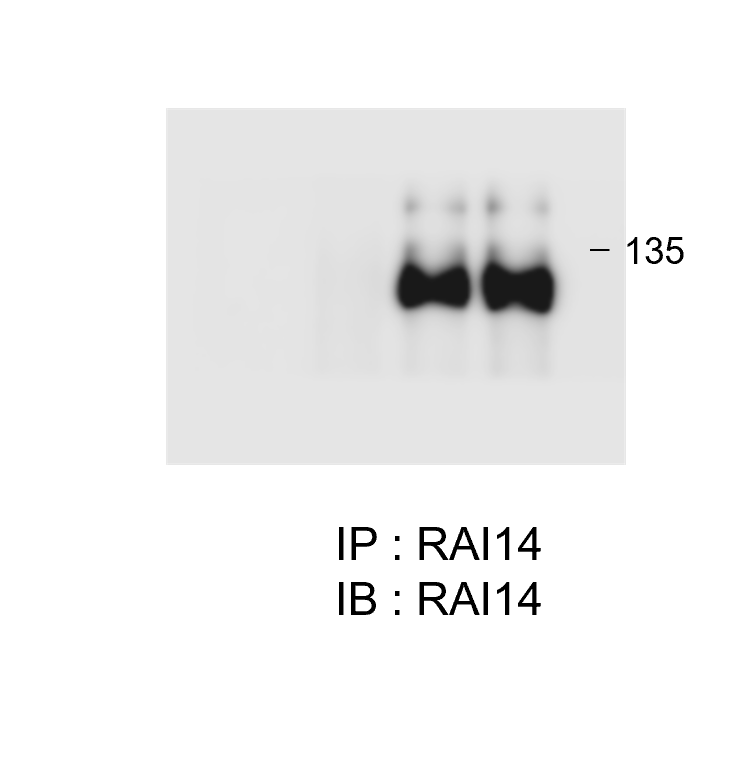

Supplement: Supplementary file 8 — Source data Fig. 6 [file 44319_2024_228_MOESM8_ESM.zip › Figure 6/Figure 6I/IP RAI14, IB RAI14.tif]

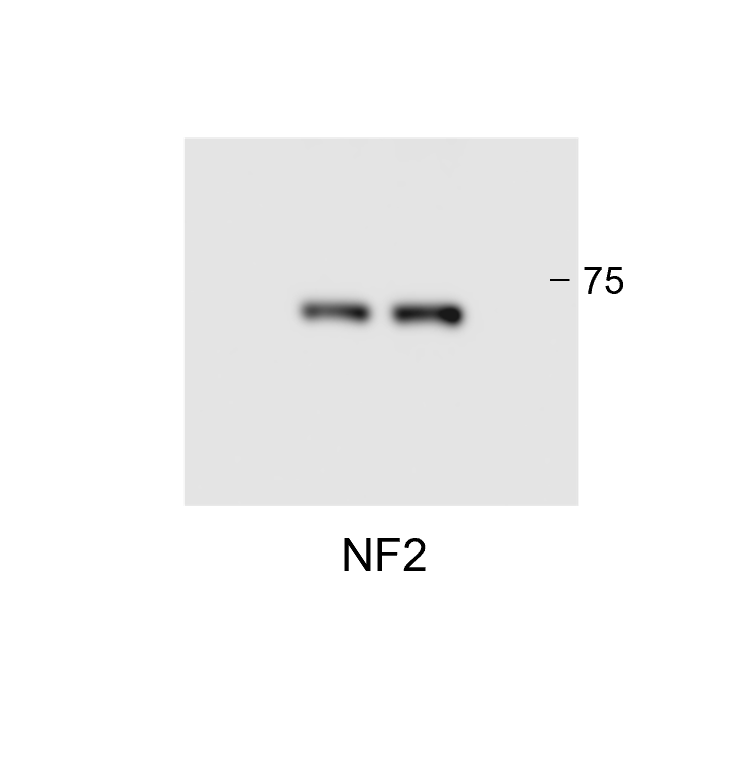

Supplement: Supplementary file 8 — Source data Fig. 6 [file 44319_2024_228_MOESM8_ESM.zip › Figure 6/Figure 6I/NF2.tif]

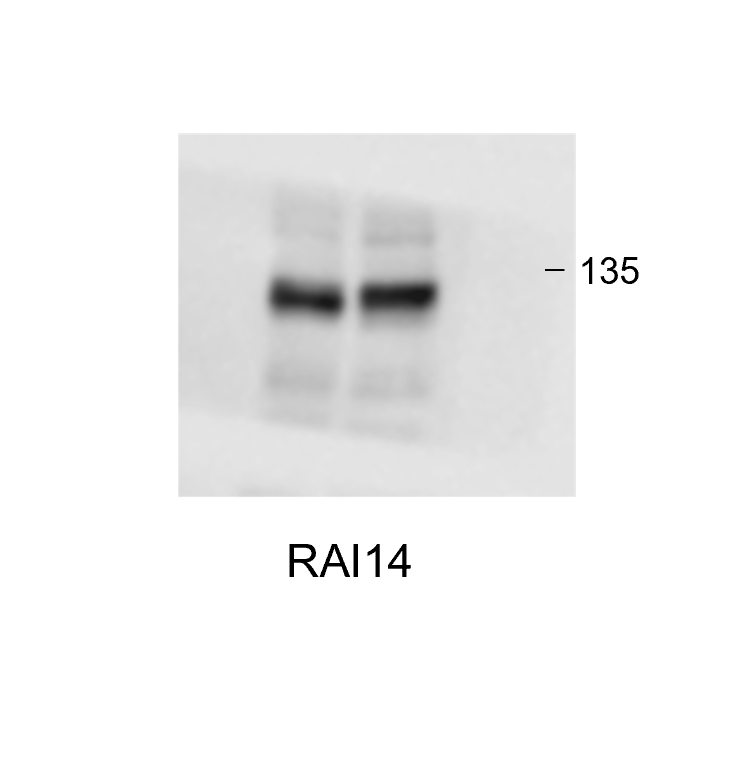

Supplement: Supplementary file 8 — Source data Fig. 6 [file 44319_2024_228_MOESM8_ESM.zip › Figure 6/Figure 6I/RAI14.tif]

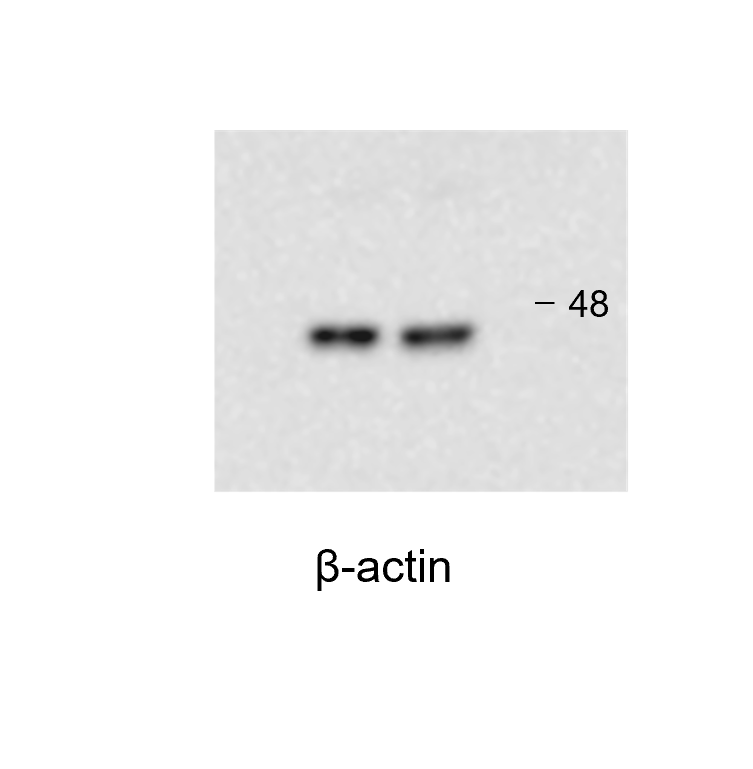

Supplement: Supplementary file 8 — Source data Fig. 6 [file 44319_2024_228_MOESM8_ESM.zip › Figure 6/Figure 6I/ÑΓ-actin.tif]

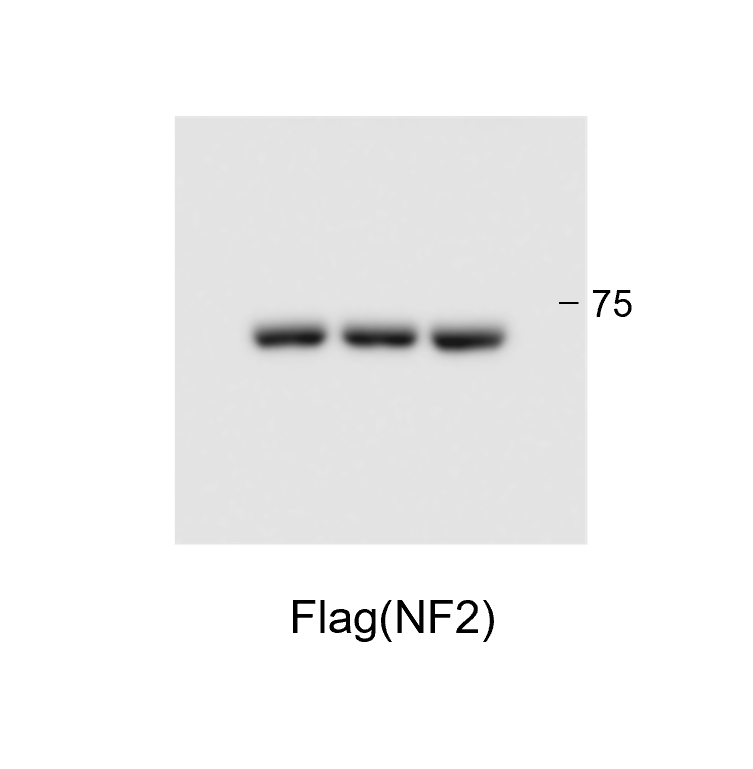

Supplement: Supplementary file 8 — Source data Fig. 6 [file 44319_2024_228_MOESM8_ESM.zip › Figure 6/Figure 6J/Flag(NF2).tif]

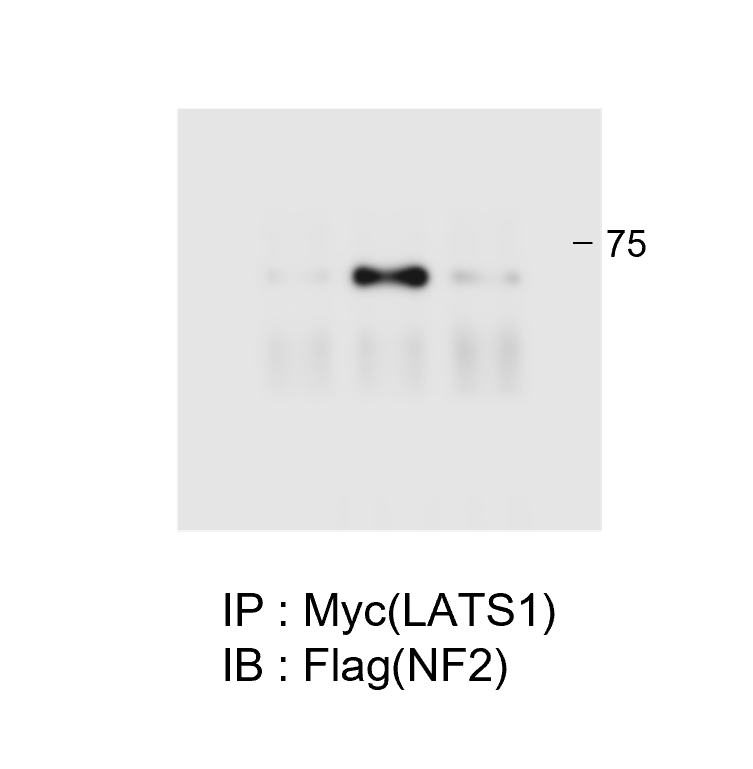

Supplement: Supplementary file 8 — Source data Fig. 6 [file 44319_2024_228_MOESM8_ESM.zip › Figure 6/Figure 6J/IP Myc(LATS1), IB Flag(NF2).tif]

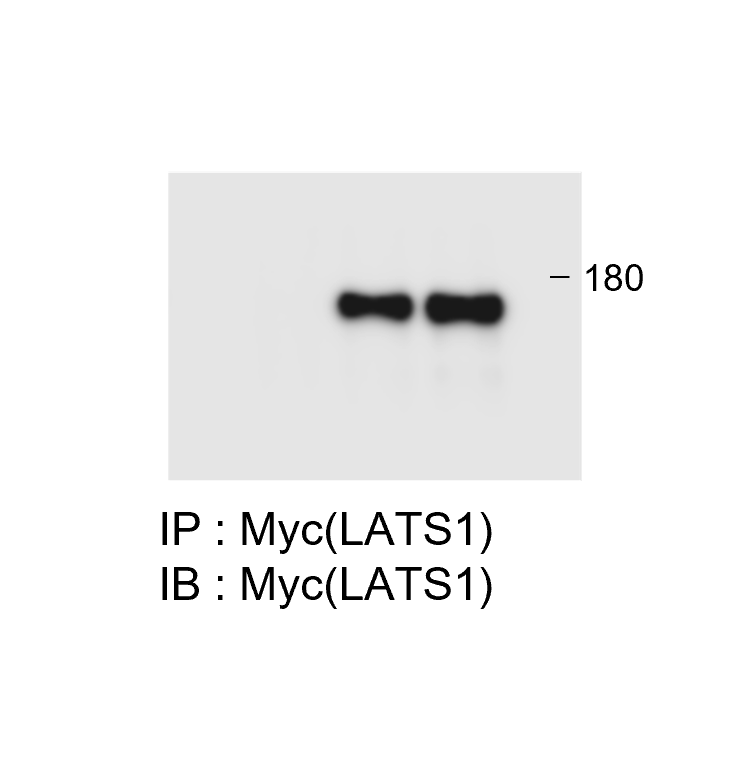

Supplement: Supplementary file 8 — Source data Fig. 6 [file 44319_2024_228_MOESM8_ESM.zip › Figure 6/Figure 6J/IP Myc(LATS1), IB Myc(LATS1).tif]

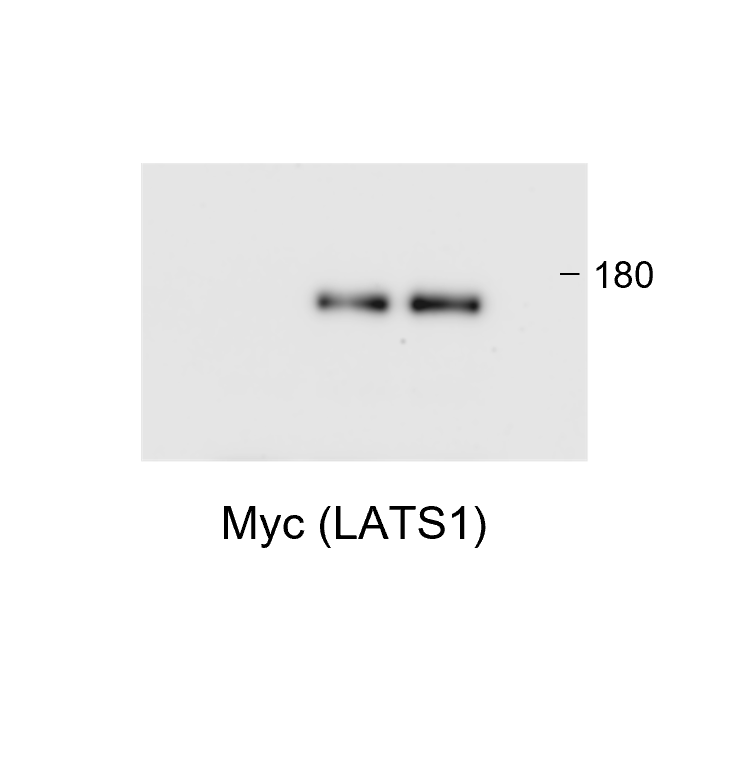

Supplement: Supplementary file 8 — Source data Fig. 6 [file 44319_2024_228_MOESM8_ESM.zip › Figure 6/Figure 6J/Myc(LATS1).tif]

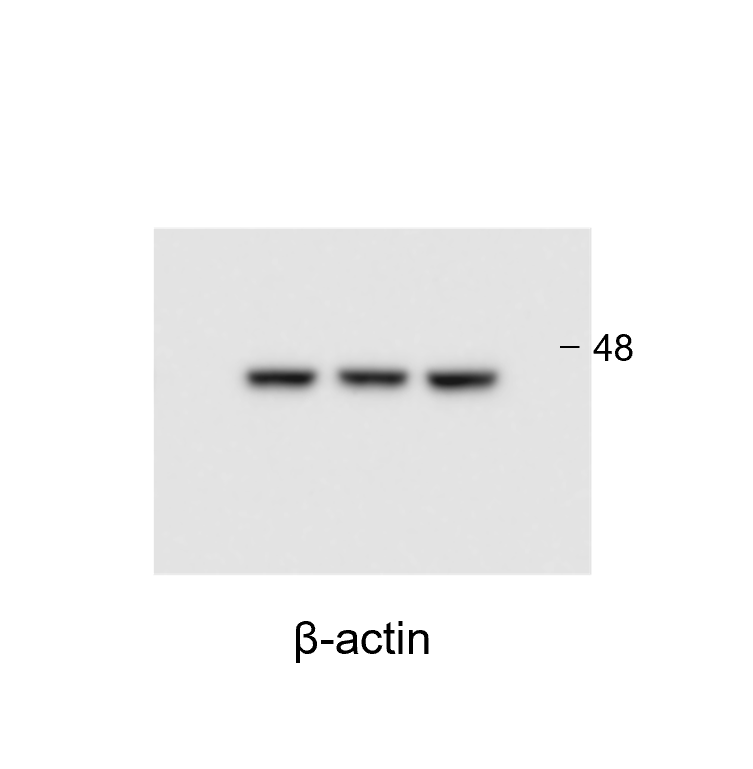

Supplement: Supplementary file 8 — Source data Fig. 6 [file 44319_2024_228_MOESM8_ESM.zip › Figure 6/Figure 6J/ÑΓ-actin.tif]

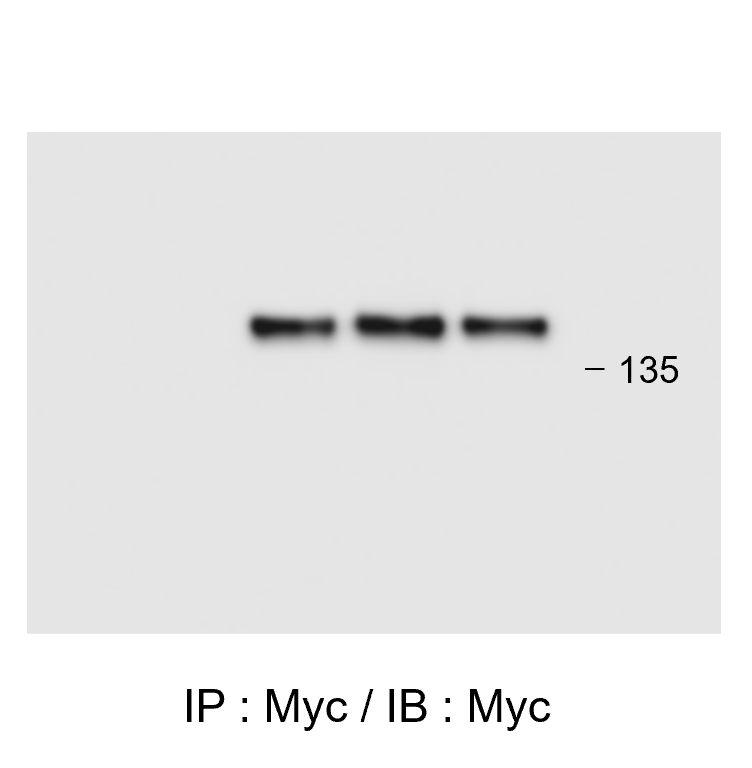

Supplement: Supplementary file 8 — Source data Fig. 6 [file 44319_2024_228_MOESM8_ESM.zip › Figure 6/Figure 6K/IP Myc(LATS1), IB Myc(LATS1).tif]

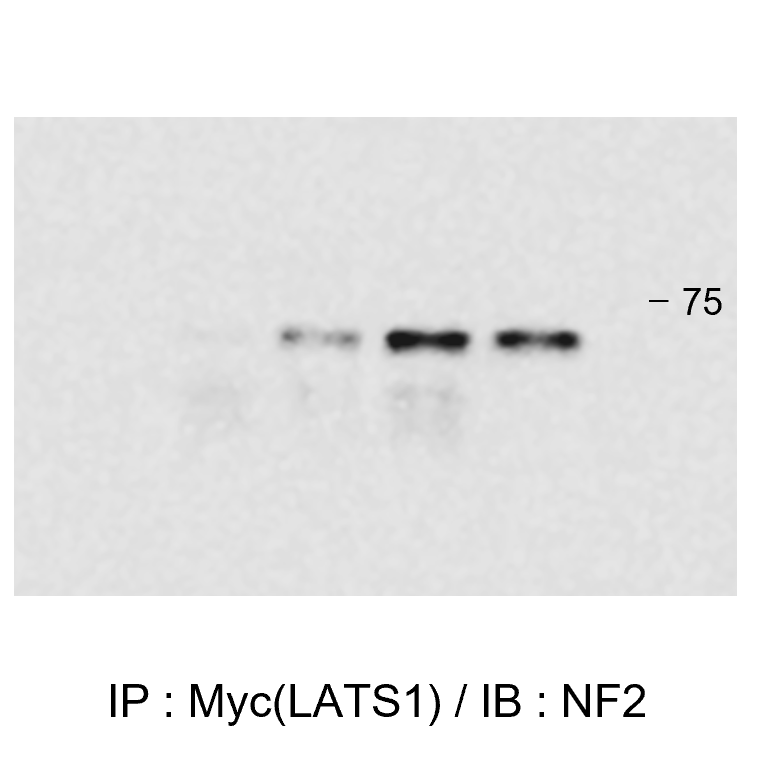

Supplement: Supplementary file 8 — Source data Fig. 6 [file 44319_2024_228_MOESM8_ESM.zip › Figure 6/Figure 6K/IP Myc(LATS1), IB NF2.tif]

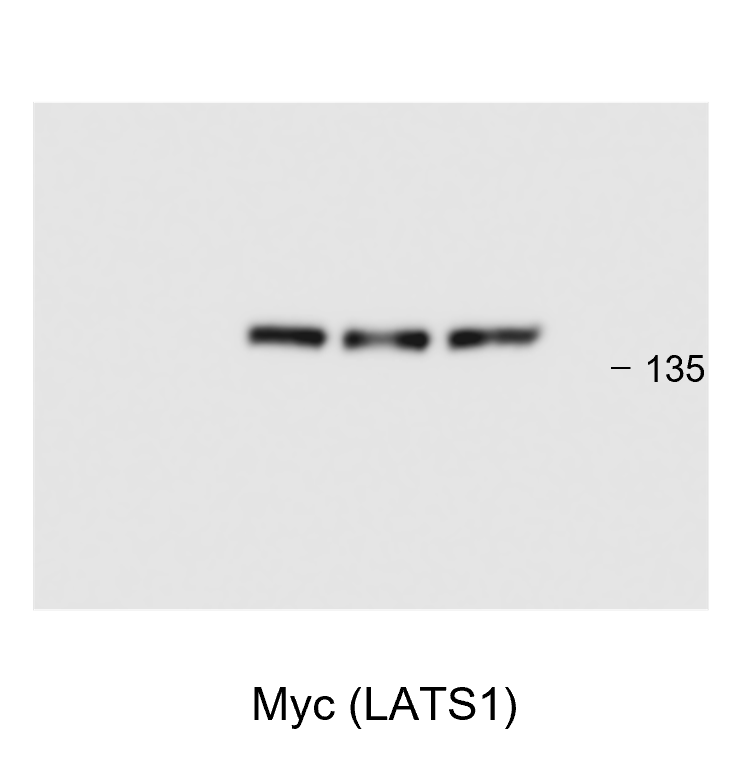

Supplement: Supplementary file 8 — Source data Fig. 6 [file 44319_2024_228_MOESM8_ESM.zip › Figure 6/Figure 6K/Myc(LATS1).tif]

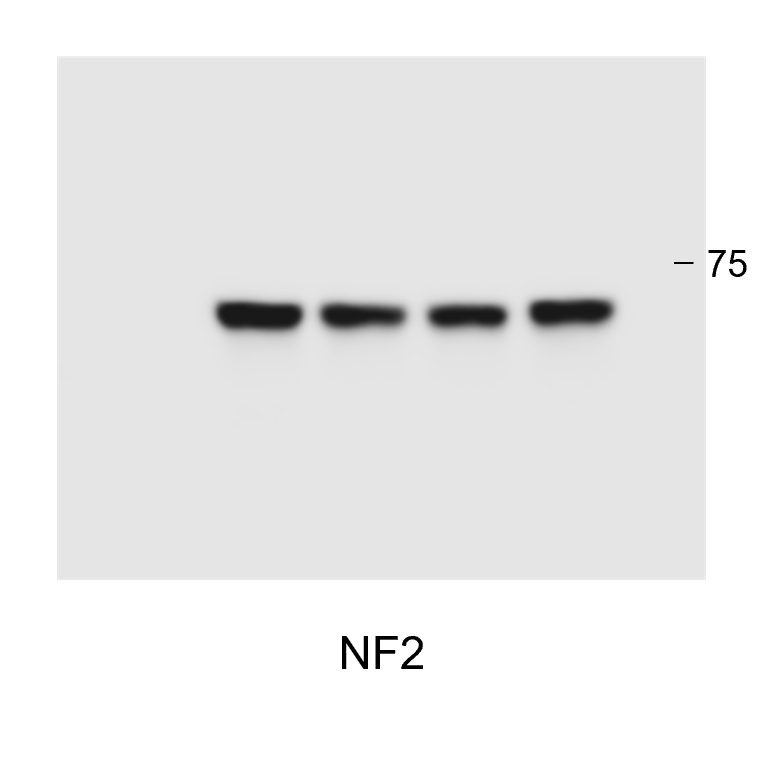

Supplement: Supplementary file 8 — Source data Fig. 6 [file 44319_2024_228_MOESM8_ESM.zip › Figure 6/Figure 6K/NF2.tif]

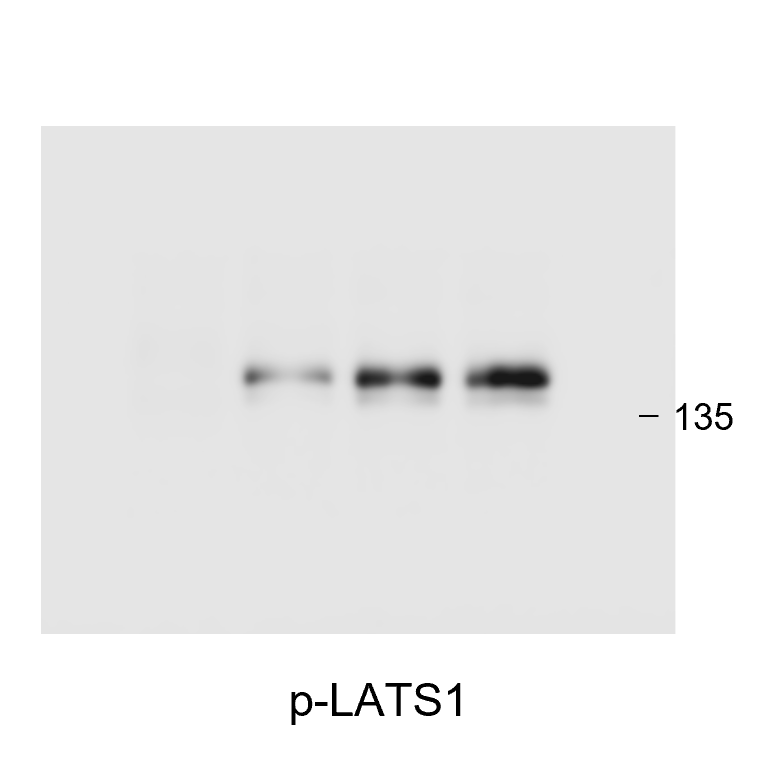

Supplement: Supplementary file 8 — Source data Fig. 6 [file 44319_2024_228_MOESM8_ESM.zip › Figure 6/Figure 6K/p-LATS1.tif]

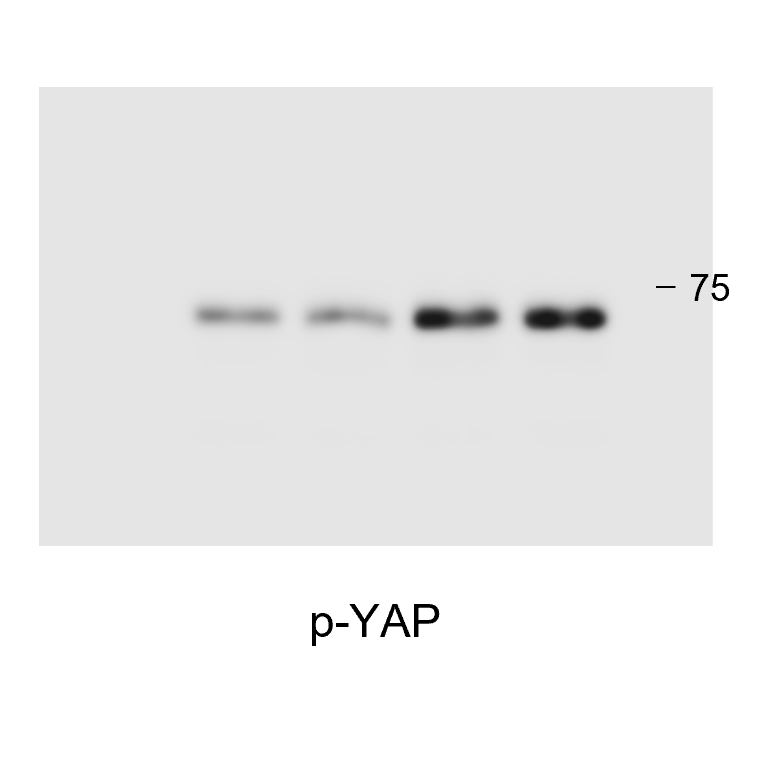

Supplement: Supplementary file 8 — Source data Fig. 6 [file 44319_2024_228_MOESM8_ESM.zip › Figure 6/Figure 6K/p-YAP.tif]
