# Supplementary figures and images for: Retinoic acid-induced protein 14 links mechanical forces to Hippo signaling (part 3 of 3)
Source: EMBO Rep. 2024 Aug 19;25(9):18. doi: 10.1038/s44319-024-00228-0 (PMC11387738; doi:10.1038/s44319-024-00228-0)

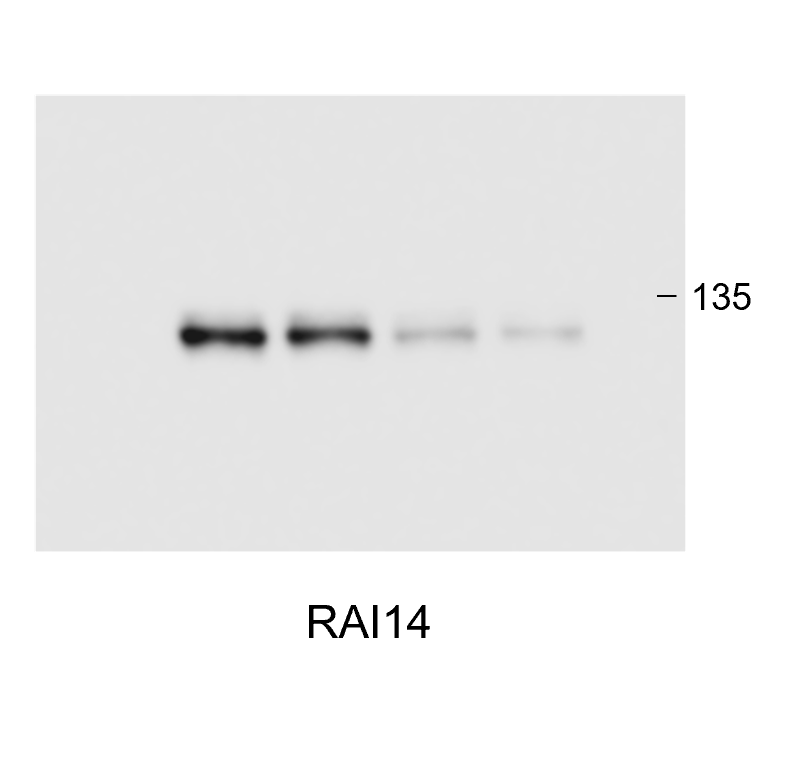

Supplement: Supplementary file 8 — Source data Fig. 6 [file 44319_2024_228_MOESM8_ESM.zip › Figure 6/Figure 6K/RAI14.tif]

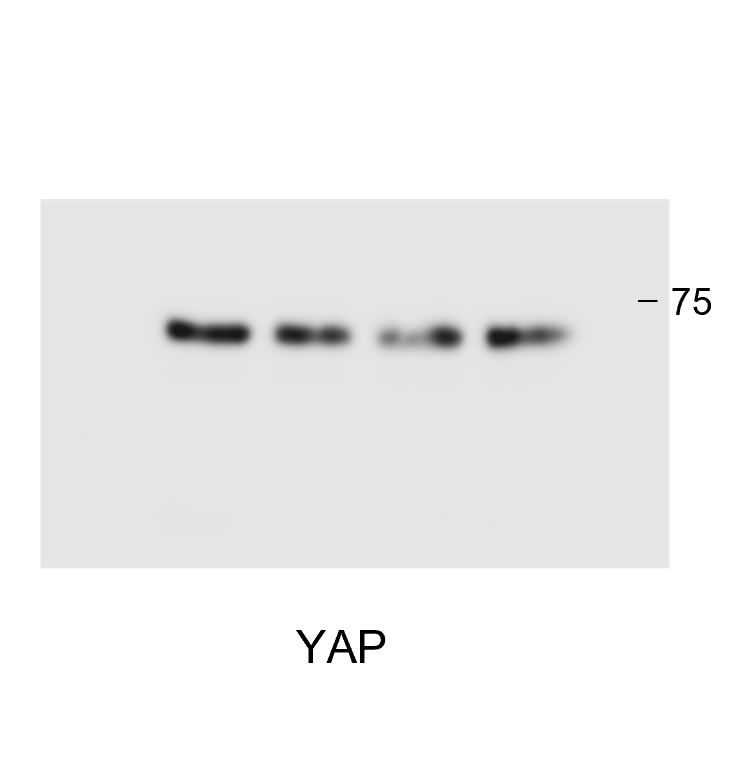

Supplement: Supplementary file 8 — Source data Fig. 6 [file 44319_2024_228_MOESM8_ESM.zip › Figure 6/Figure 6K/YAP.tif]

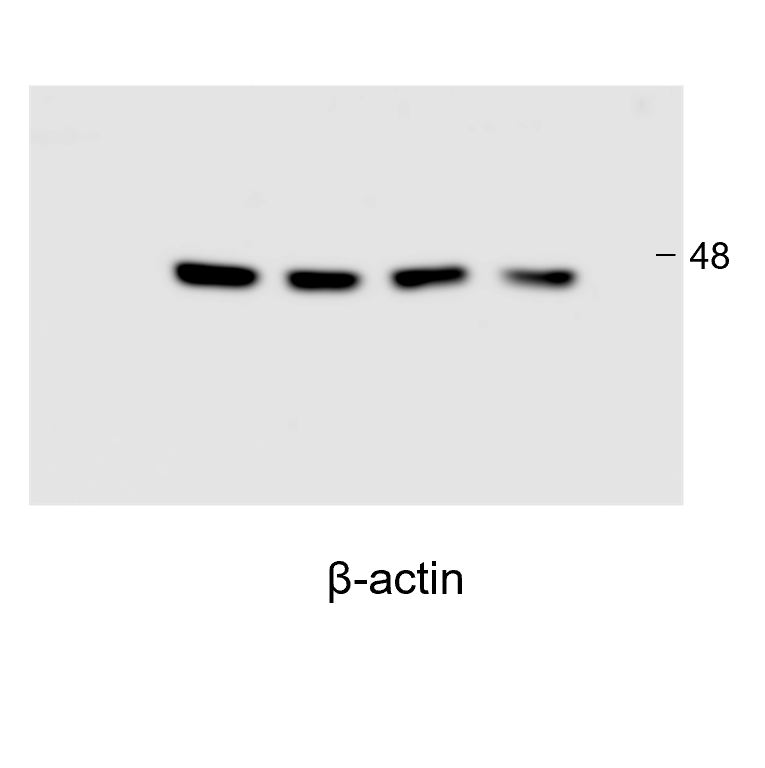

Supplement: Supplementary file 8 — Source data Fig. 6 [file 44319_2024_228_MOESM8_ESM.zip › Figure 6/Figure 6K/ÑΓ-actin.tif]

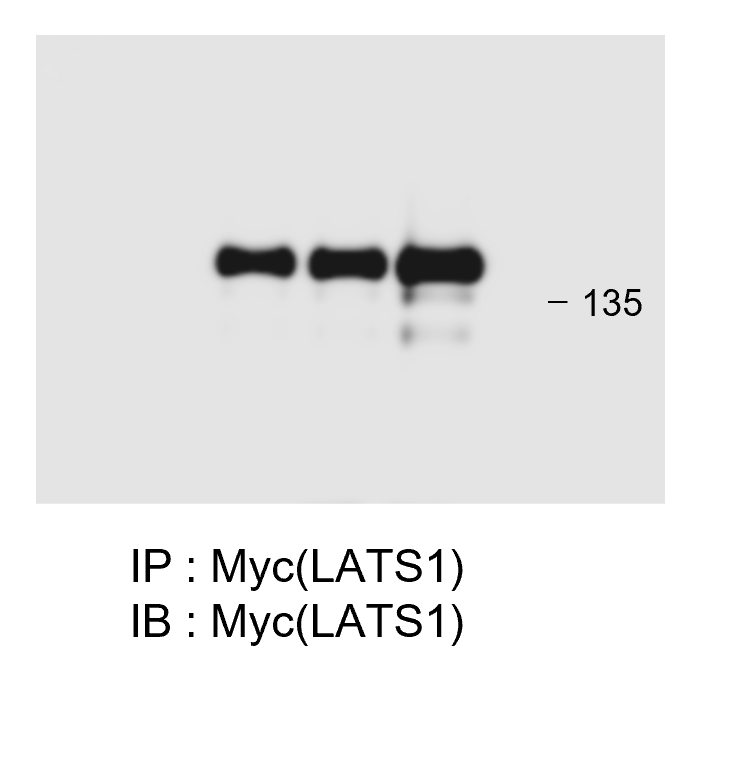

Supplement: Supplementary file 8 — Source data Fig. 6 [file 44319_2024_228_MOESM8_ESM.zip › Figure 6/Figure 6L/IP Myc(LATS1), IB Myc(LATS1).tif]

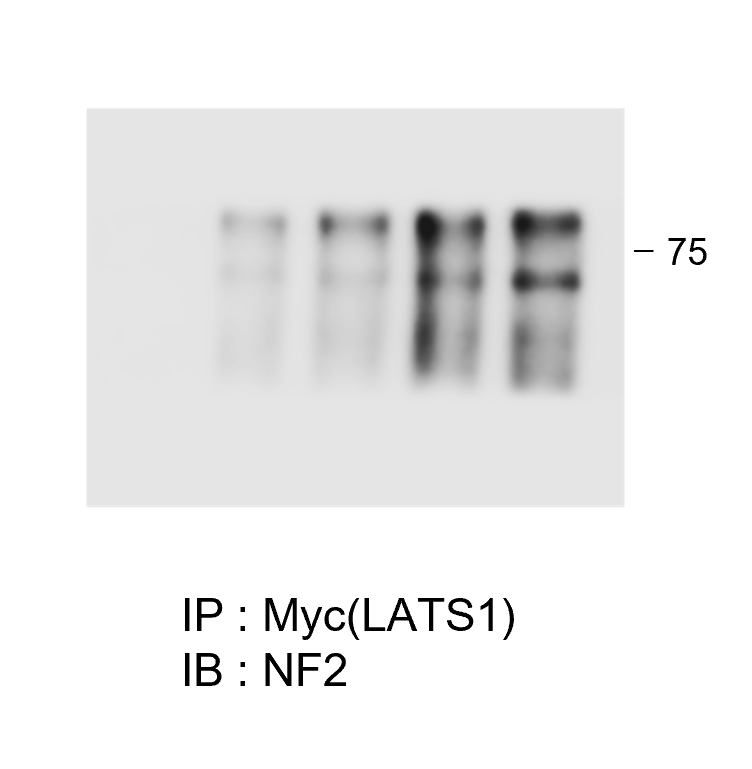

Supplement: Supplementary file 8 — Source data Fig. 6 [file 44319_2024_228_MOESM8_ESM.zip › Figure 6/Figure 6L/IP Myc(LATS1), IB NF2.tif]

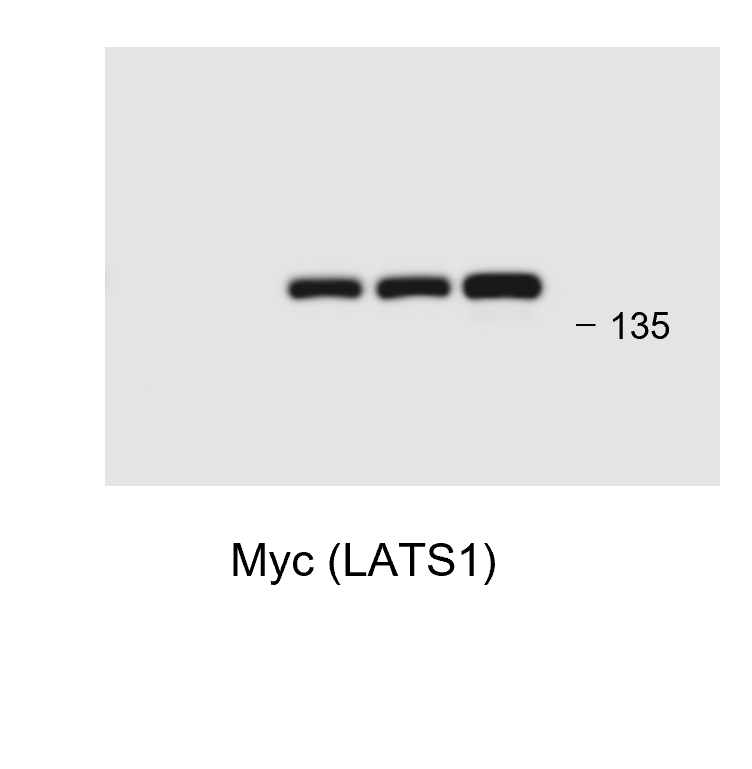

Supplement: Supplementary file 8 — Source data Fig. 6 [file 44319_2024_228_MOESM8_ESM.zip › Figure 6/Figure 6L/Myc(LATS1).tif]

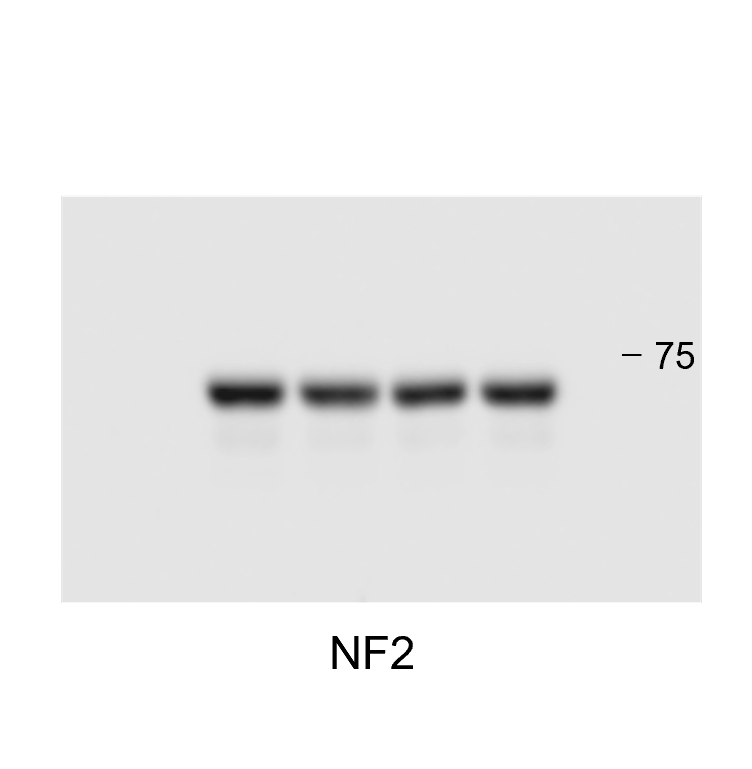

Supplement: Supplementary file 8 — Source data Fig. 6 [file 44319_2024_228_MOESM8_ESM.zip › Figure 6/Figure 6L/NF2.tif]

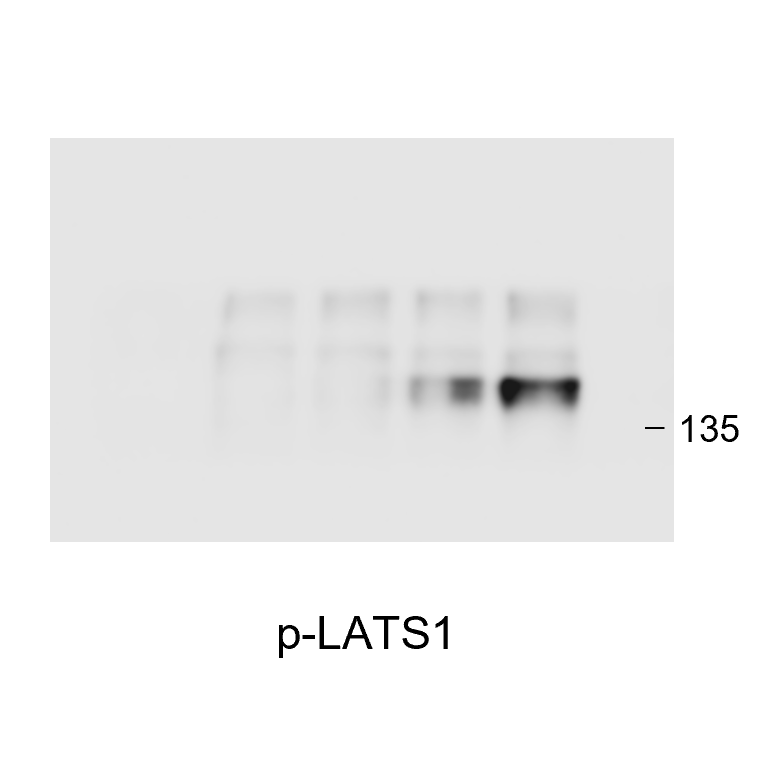

Supplement: Supplementary file 8 — Source data Fig. 6 [file 44319_2024_228_MOESM8_ESM.zip › Figure 6/Figure 6L/p-LATS1.tif]

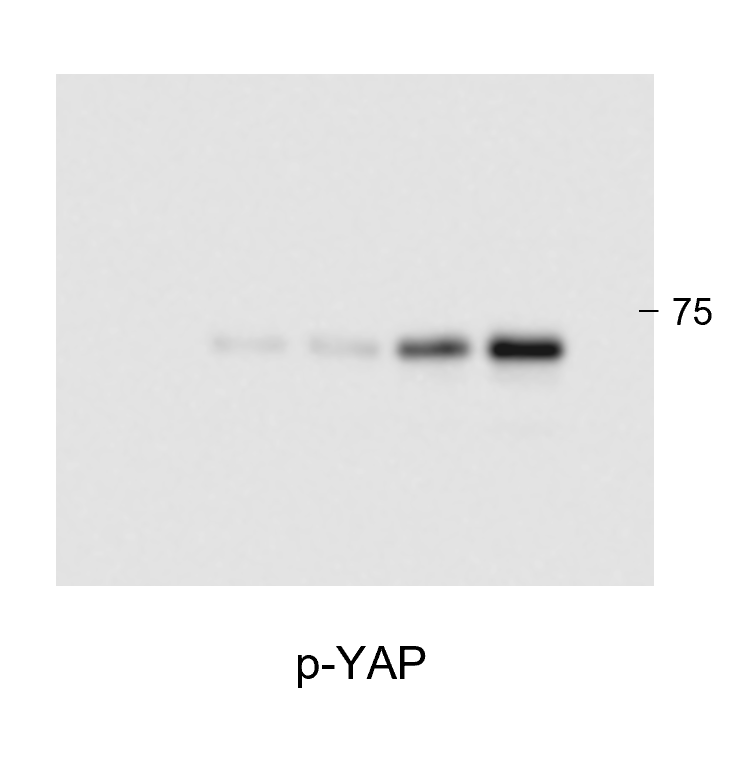

Supplement: Supplementary file 8 — Source data Fig. 6 [file 44319_2024_228_MOESM8_ESM.zip › Figure 6/Figure 6L/p-YAP.tif]

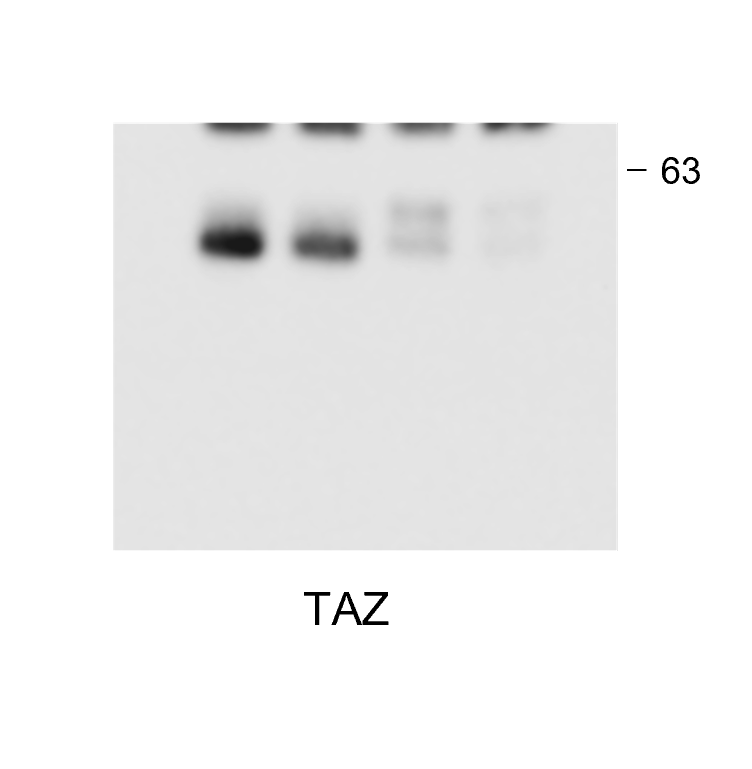

Supplement: Supplementary file 8 — Source data Fig. 6 [file 44319_2024_228_MOESM8_ESM.zip › Figure 6/Figure 6L/TAZ.tif]

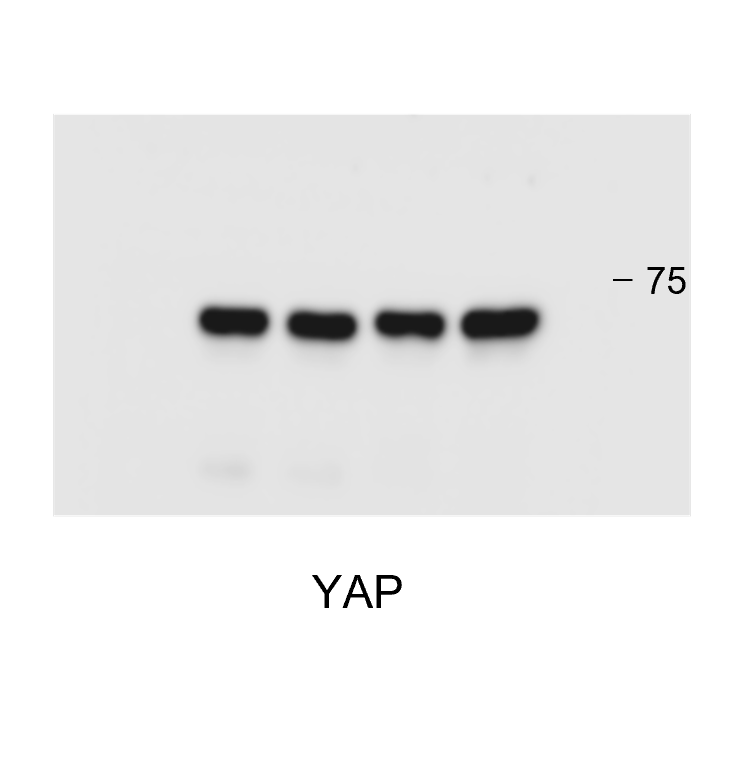

Supplement: Supplementary file 8 — Source data Fig. 6 [file 44319_2024_228_MOESM8_ESM.zip › Figure 6/Figure 6L/YAP.tif]

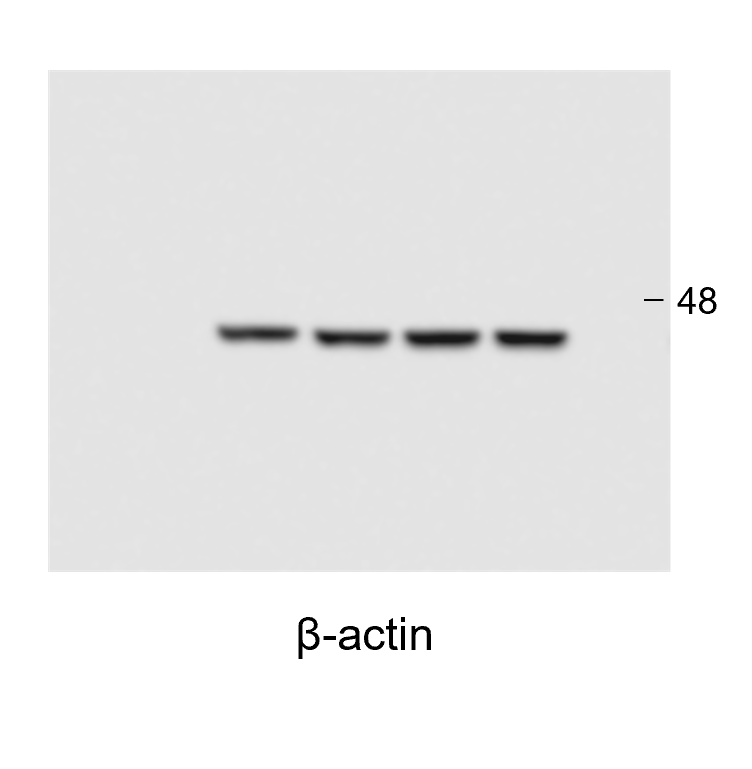

Supplement: Supplementary file 8 — Source data Fig. 6 [file 44319_2024_228_MOESM8_ESM.zip › Figure 6/Figure 6L/ÑΓ-actin.tif]

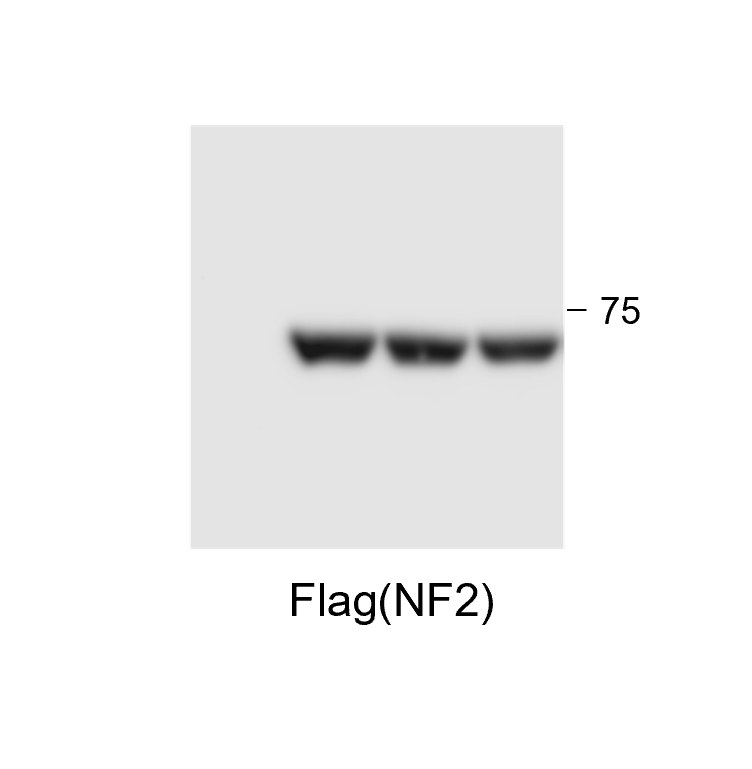

Supplement: Supplementary file 8 — Source data Fig. 6 [file 44319_2024_228_MOESM8_ESM.zip › Figure 6/Figure 6M/Flag(NF2).tif]

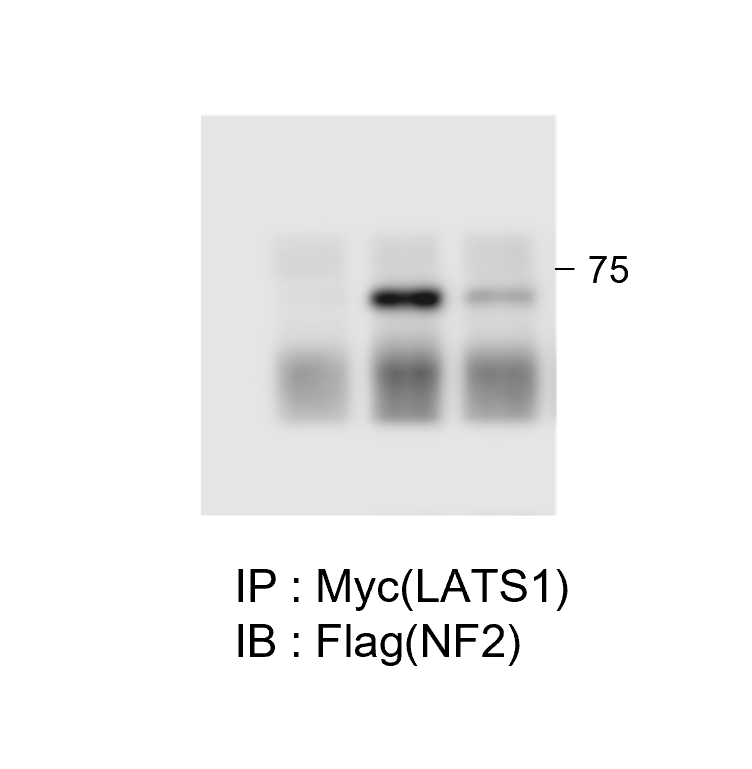

Supplement: Supplementary file 8 — Source data Fig. 6 [file 44319_2024_228_MOESM8_ESM.zip › Figure 6/Figure 6M/IP Myc(LATS1), IB Flag(NF2).tif]

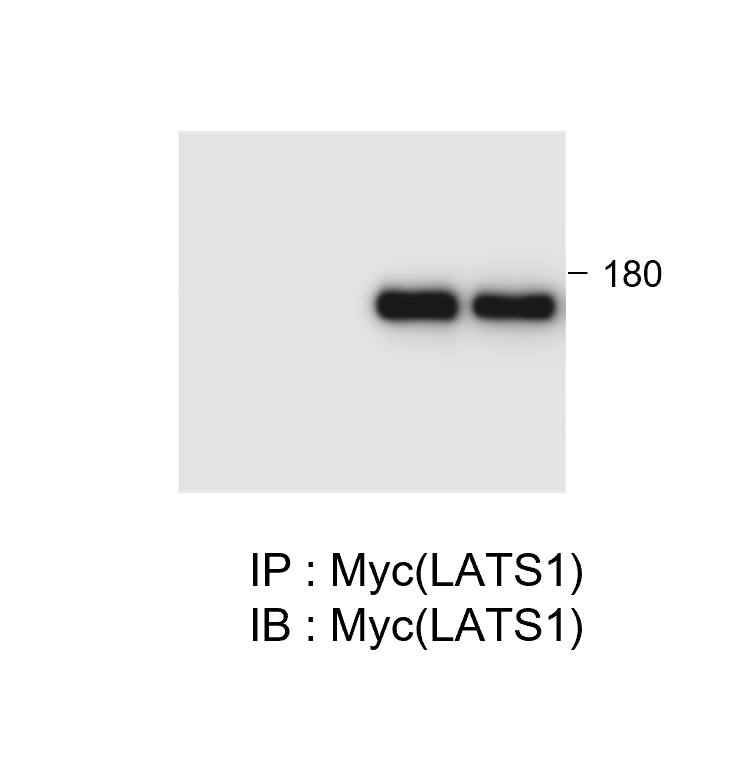

Supplement: Supplementary file 8 — Source data Fig. 6 [file 44319_2024_228_MOESM8_ESM.zip › Figure 6/Figure 6M/IP Myc(LATS1), IB Myc(LATS1).tif]

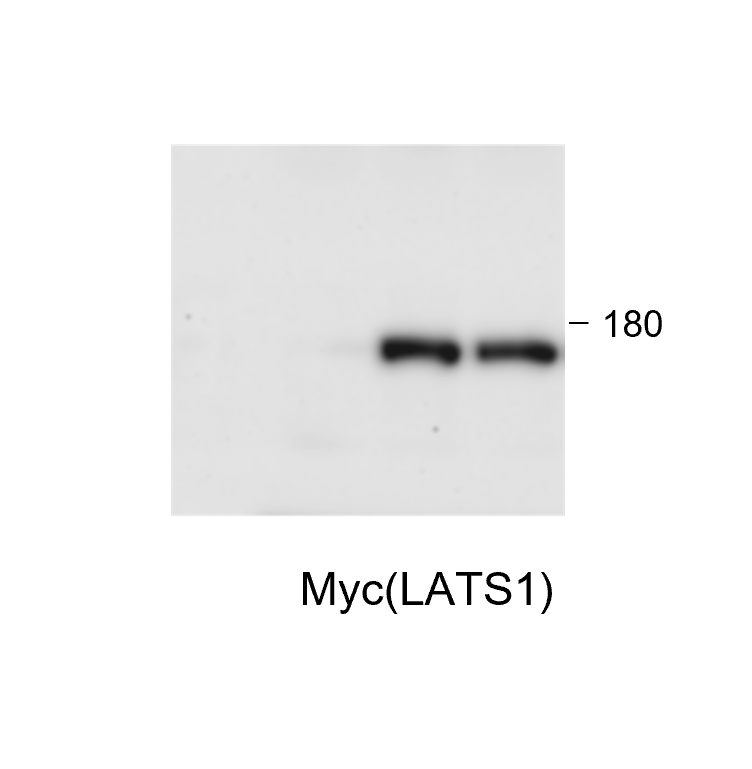

Supplement: Supplementary file 8 — Source data Fig. 6 [file 44319_2024_228_MOESM8_ESM.zip › Figure 6/Figure 6M/Myc(LATS1).tif]

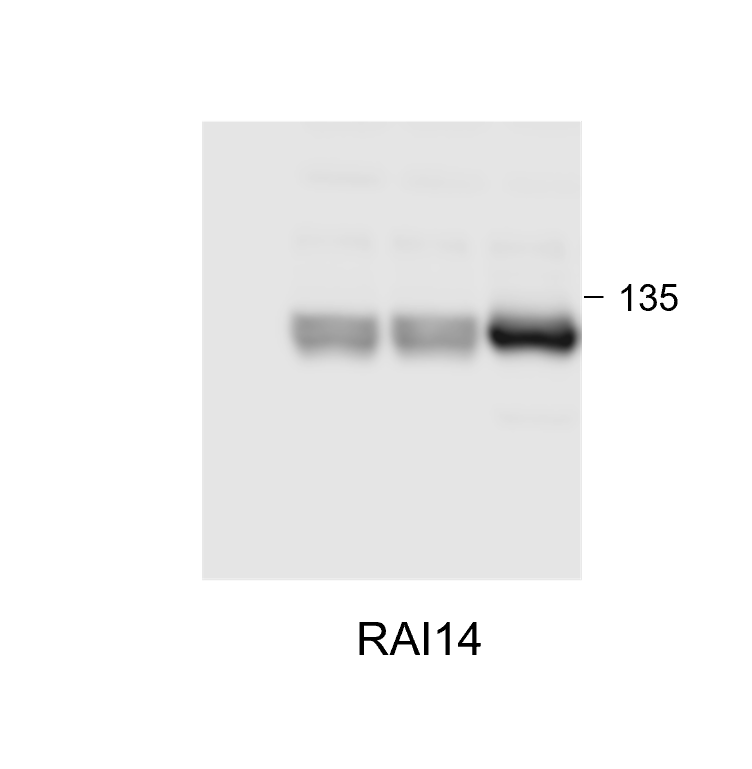

Supplement: Supplementary file 8 — Source data Fig. 6 [file 44319_2024_228_MOESM8_ESM.zip › Figure 6/Figure 6M/RAI14.tif]

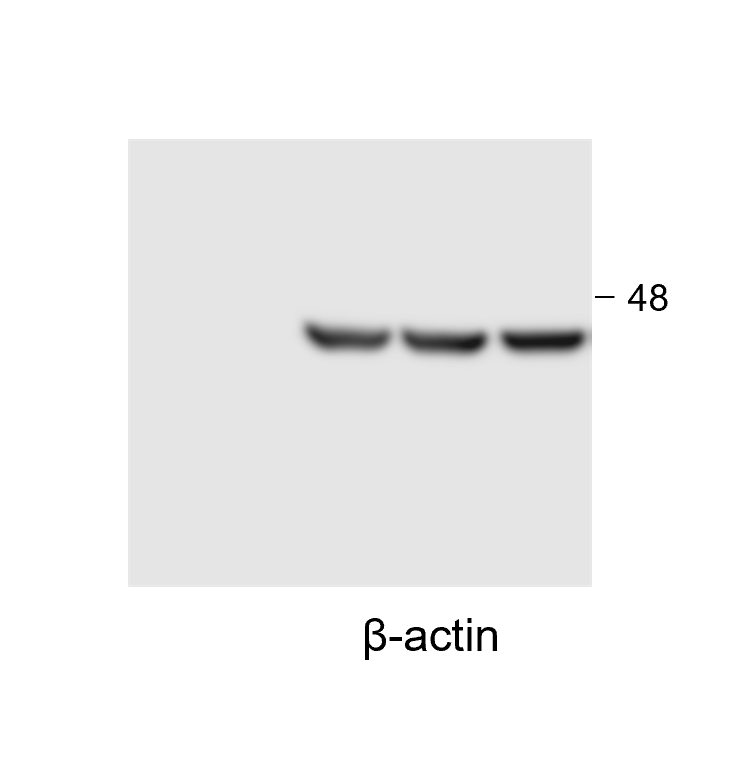

Supplement: Supplementary file 8 — Source data Fig. 6 [file 44319_2024_228_MOESM8_ESM.zip › Figure 6/Figure 6M/ÑΓ-actin.tif]

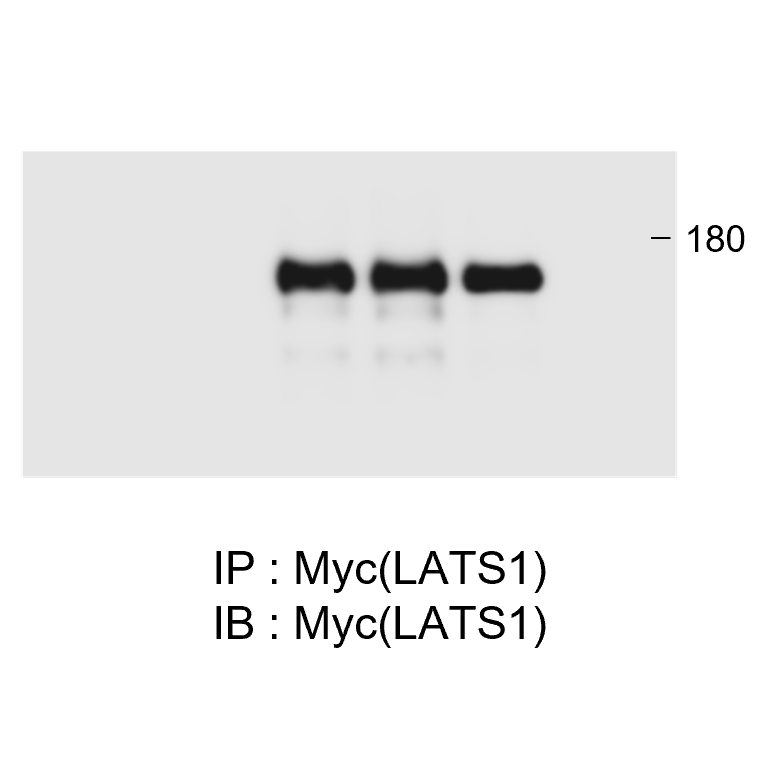

Supplement: Supplementary file 8 — Source data Fig. 6 [file 44319_2024_228_MOESM8_ESM.zip › Figure 6/Figure 6N/IP Myc(LATS1), IB Myc(LATS1).tif]

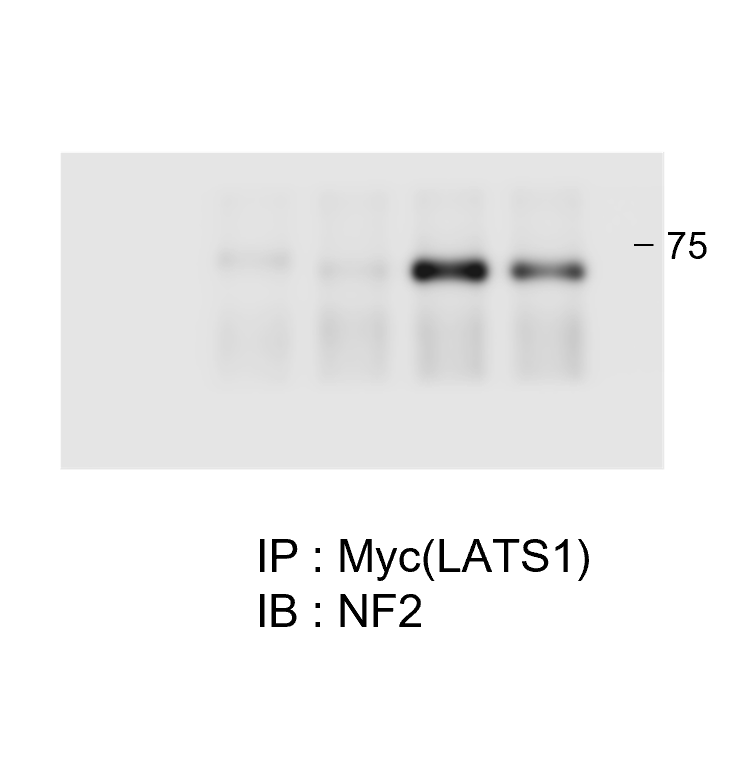

Supplement: Supplementary file 8 — Source data Fig. 6 [file 44319_2024_228_MOESM8_ESM.zip › Figure 6/Figure 6N/IP Myc(LATS1), IB NF2.tif]

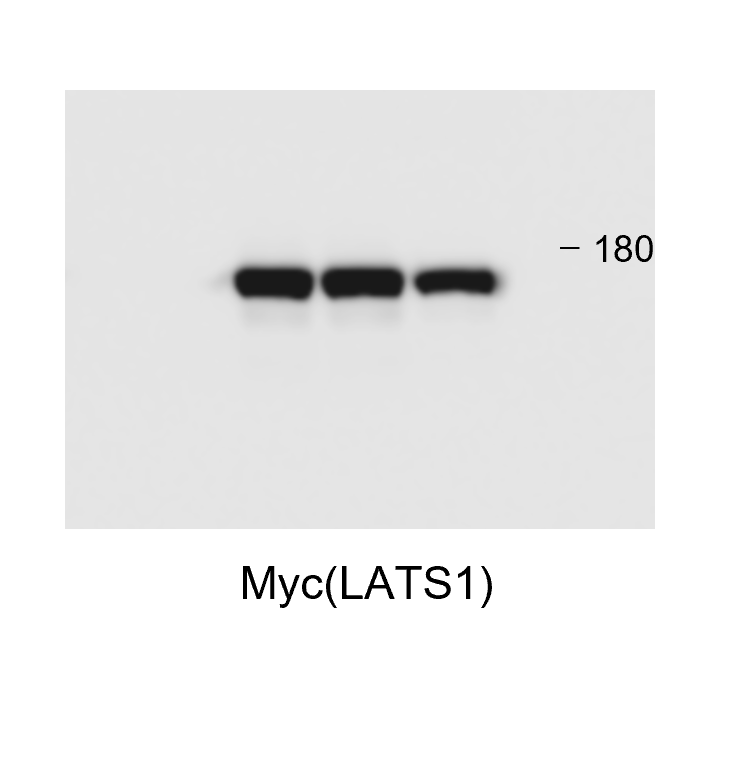

Supplement: Supplementary file 8 — Source data Fig. 6 [file 44319_2024_228_MOESM8_ESM.zip › Figure 6/Figure 6N/Myc(LATS1).tif]

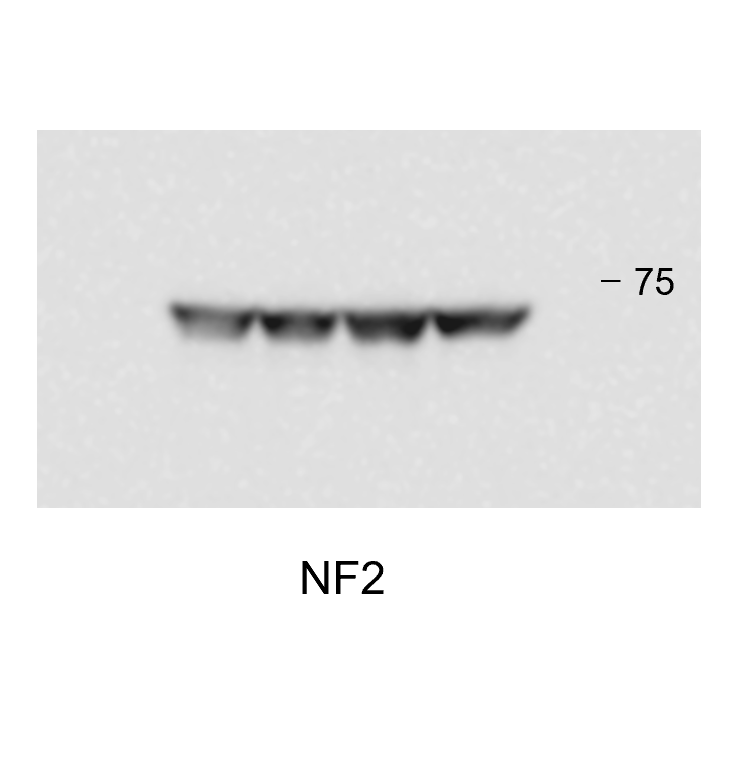

Supplement: Supplementary file 8 — Source data Fig. 6 [file 44319_2024_228_MOESM8_ESM.zip › Figure 6/Figure 6N/NF2.tif]

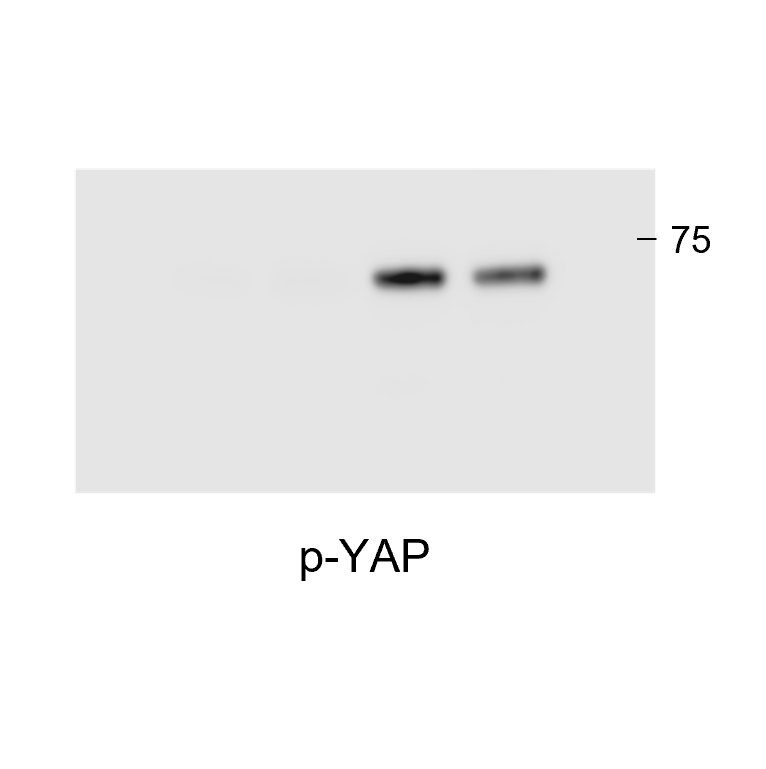

Supplement: Supplementary file 8 — Source data Fig. 6 [file 44319_2024_228_MOESM8_ESM.zip › Figure 6/Figure 6N/p-YAP.tif]

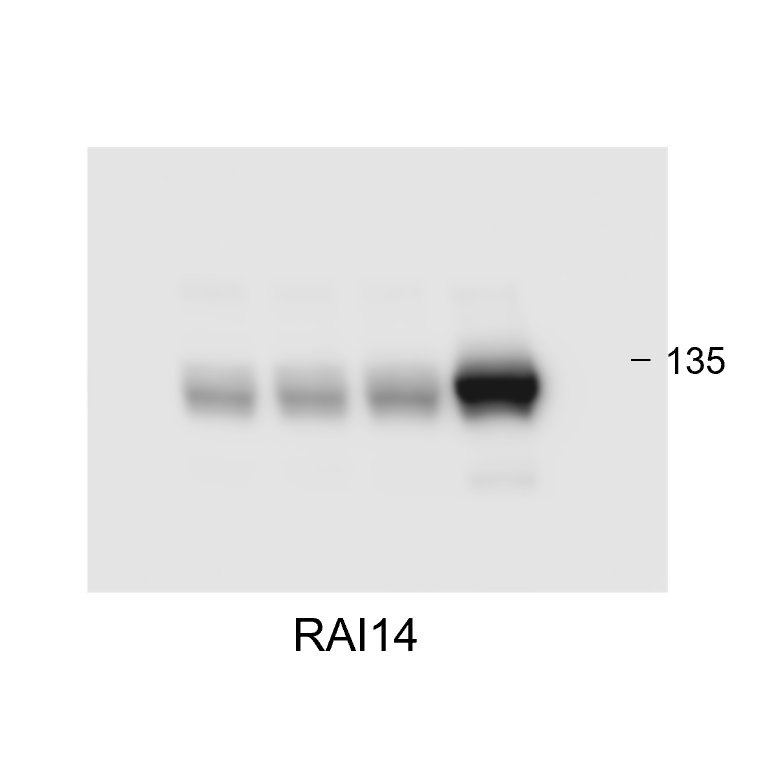

Supplement: Supplementary file 8 — Source data Fig. 6 [file 44319_2024_228_MOESM8_ESM.zip › Figure 6/Figure 6N/RAI14.tif]

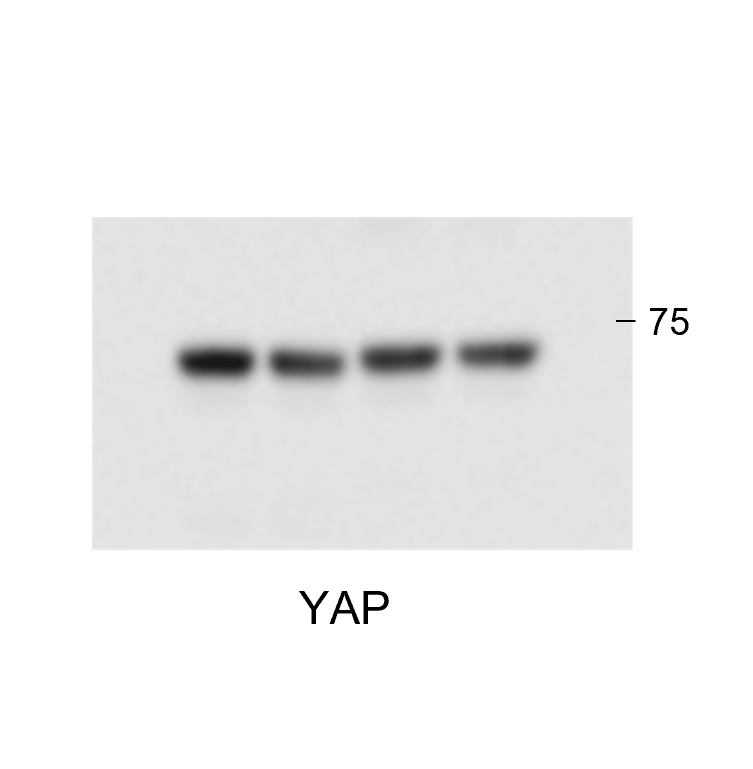

Supplement: Supplementary file 8 — Source data Fig. 6 [file 44319_2024_228_MOESM8_ESM.zip › Figure 6/Figure 6N/YAP.tif]

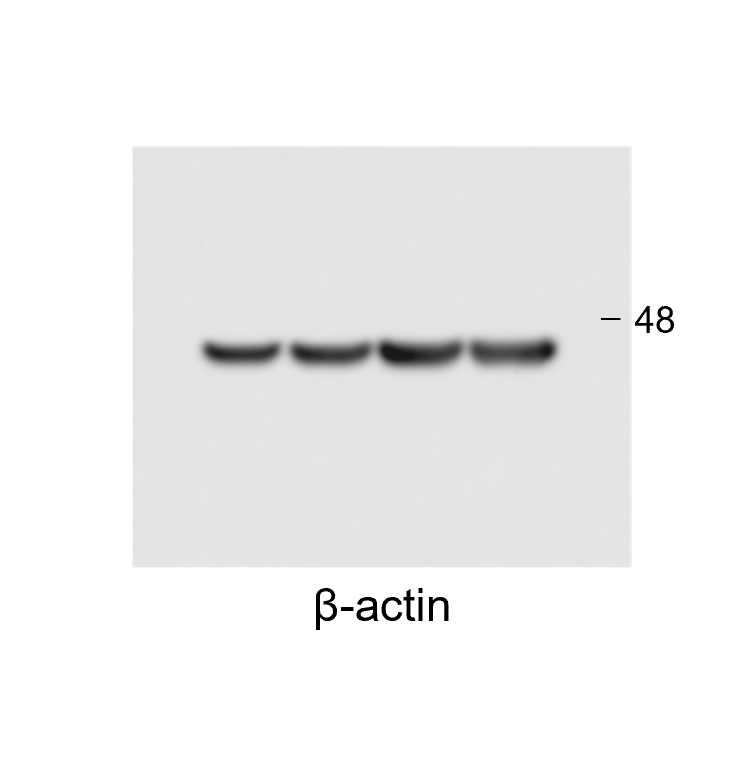

Supplement: Supplementary file 8 — Source data Fig. 6 [file 44319_2024_228_MOESM8_ESM.zip › Figure 6/Figure 6N/ÑΓ-actin.tif]

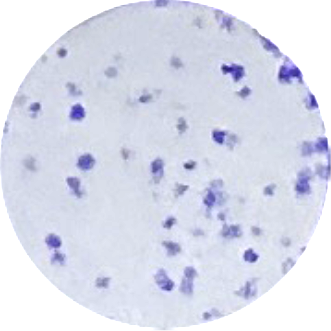

Supplement: Supplementary file 9 — Source data Fig. 7 [file 44319_2024_228_MOESM9_ESM.zip › Figure 7/Figure 7B/Ctrl+DMSO.tif]

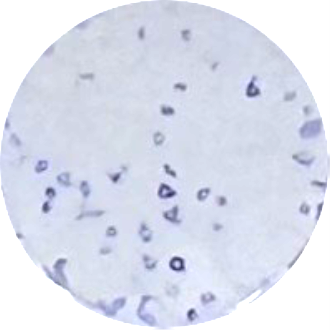

Supplement: Supplementary file 9 — Source data Fig. 7 [file 44319_2024_228_MOESM9_ESM.zip › Figure 7/Figure 7B/Ctrl+VP.tif]

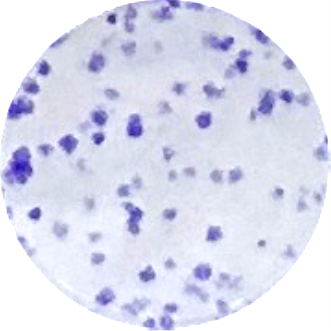

Supplement: Supplementary file 9 — Source data Fig. 7 [file 44319_2024_228_MOESM9_ESM.zip › Figure 7/Figure 7B/RAI14+DMSO.tif]

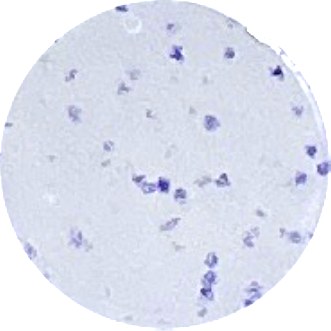

Supplement: Supplementary file 9 — Source data Fig. 7 [file 44319_2024_228_MOESM9_ESM.zip › Figure 7/Figure 7B/RAI14+VP.tif]

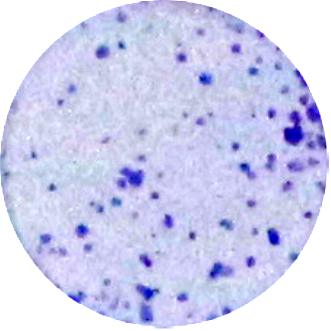

Supplement: Supplementary file 9 — Source data Fig. 7 [file 44319_2024_228_MOESM9_ESM.zip › Figure 7/Figure 7D/Ctrl+DMSO.tif]

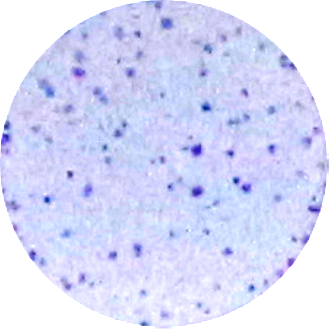

Supplement: Supplementary file 9 — Source data Fig. 7 [file 44319_2024_228_MOESM9_ESM.zip › Figure 7/Figure 7D/Ctrl+MGH-CP1.tif]

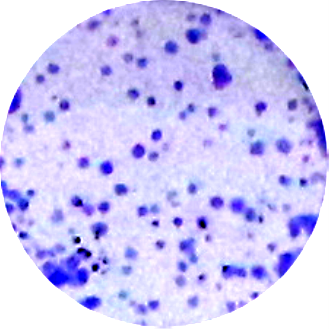

Supplement: Supplementary file 9 — Source data Fig. 7 [file 44319_2024_228_MOESM9_ESM.zip › Figure 7/Figure 7D/RAI14+DMSO.tif]

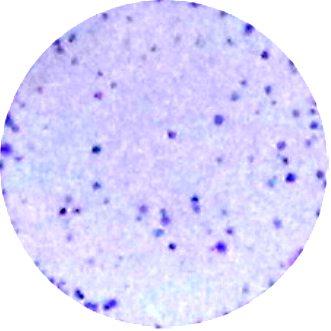

Supplement: Supplementary file 9 — Source data Fig. 7 [file 44319_2024_228_MOESM9_ESM.zip › Figure 7/Figure 7D/RAI14+MGH-CP1.tif]

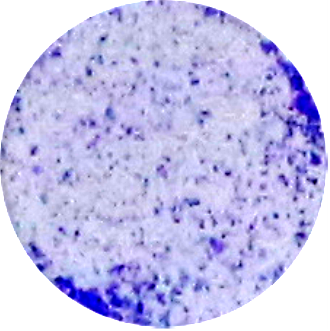

Supplement: Supplementary file 9 — Source data Fig. 7 [file 44319_2024_228_MOESM9_ESM.zip › Figure 7/Figure 7F/Ctrl+DMSO.tif]

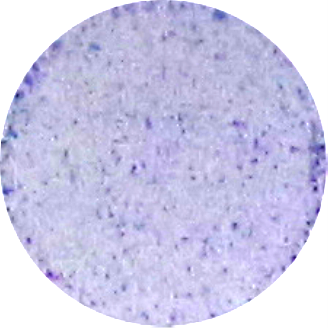

Supplement: Supplementary file 9 — Source data Fig. 7 [file 44319_2024_228_MOESM9_ESM.zip › Figure 7/Figure 7F/Ctrl+MGH-CP1.tif]

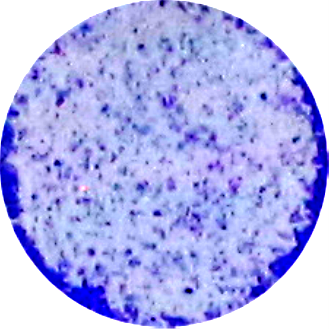

Supplement: Supplementary file 9 — Source data Fig. 7 [file 44319_2024_228_MOESM9_ESM.zip › Figure 7/Figure 7F/RAI14+DMSO.tif]

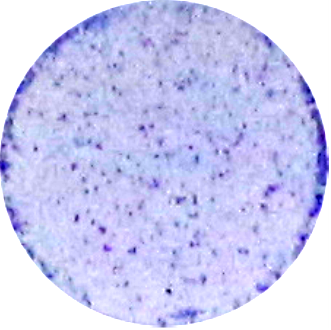

Supplement: Supplementary file 9 — Source data Fig. 7 [file 44319_2024_228_MOESM9_ESM.zip › Figure 7/Figure 7F/RAI14+MGH-CP1.tif]

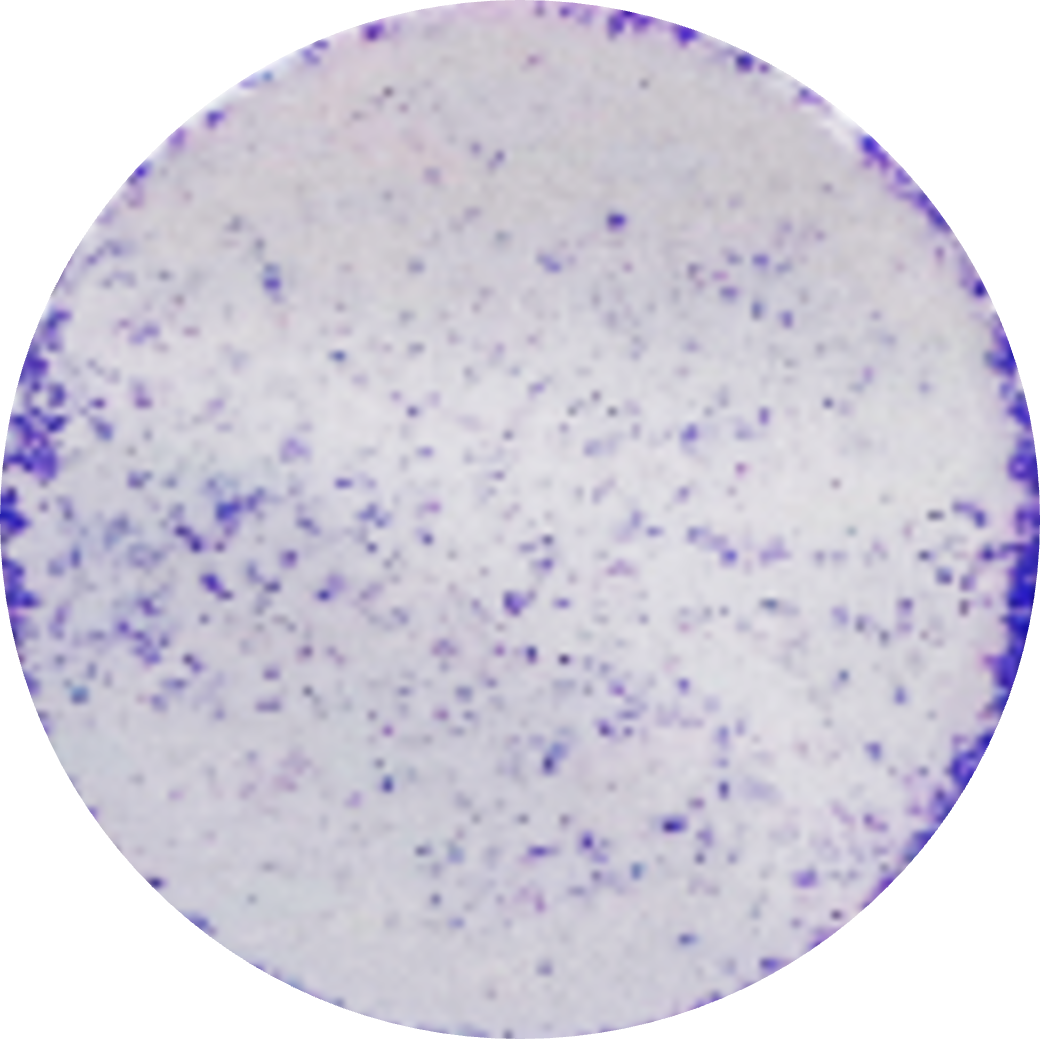

Supplement: Supplementary file 9 — Source data Fig. 7 [file 44319_2024_228_MOESM9_ESM.zip › Figure 7/Figure 7H/shCtrl+DMSO.tif]

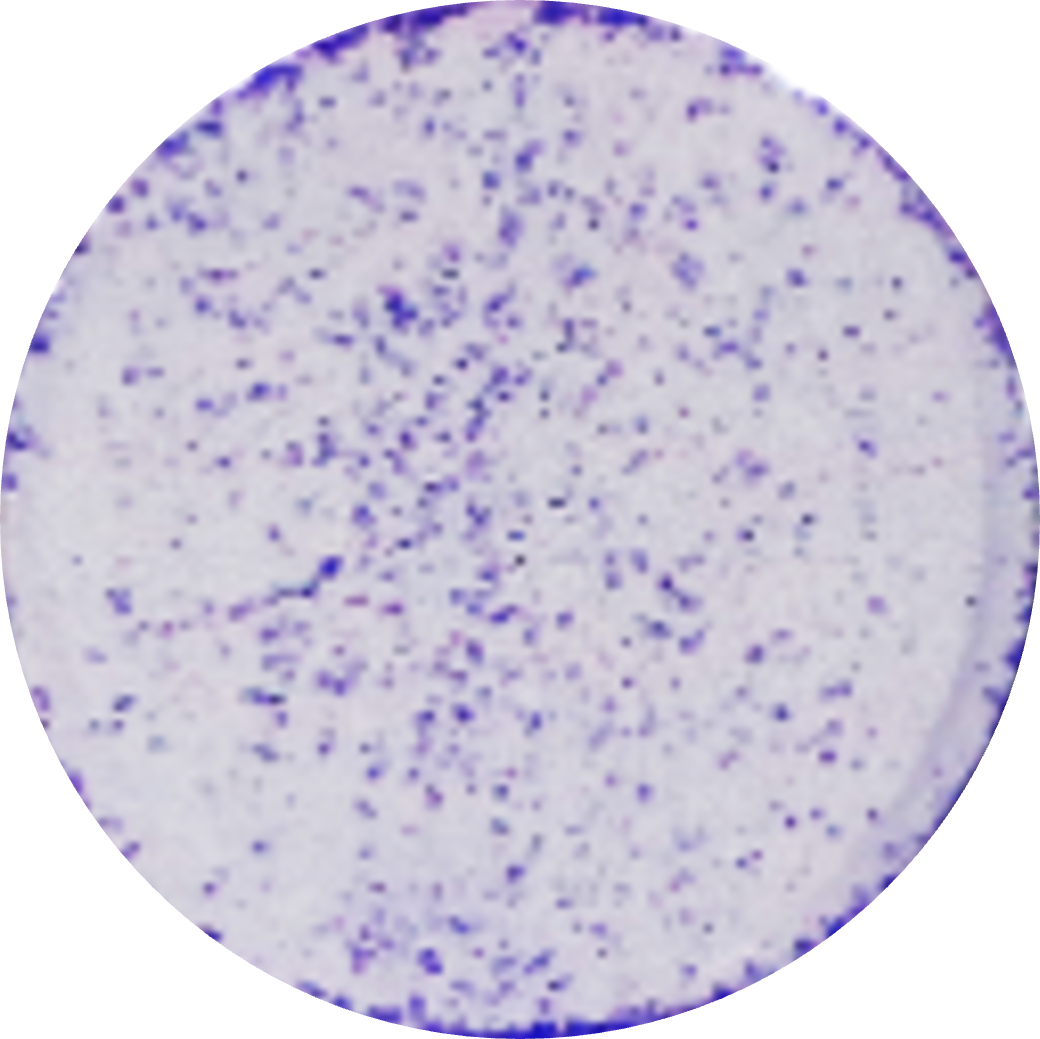

Supplement: Supplementary file 9 — Source data Fig. 7 [file 44319_2024_228_MOESM9_ESM.zip › Figure 7/Figure 7H/shCtrl+TRULI.tif]

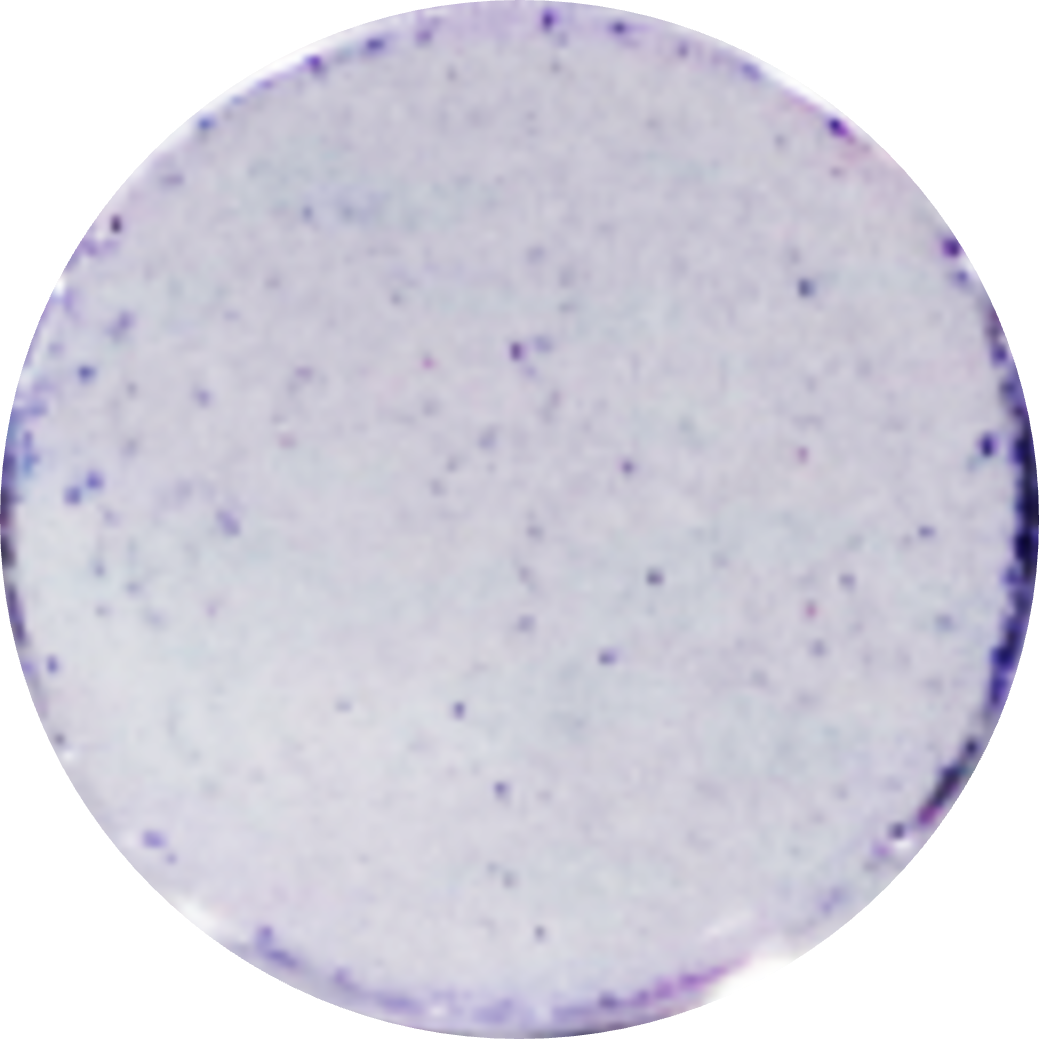

Supplement: Supplementary file 9 — Source data Fig. 7 [file 44319_2024_228_MOESM9_ESM.zip › Figure 7/Figure 7H/shRAI14#1+DMSO.tif]

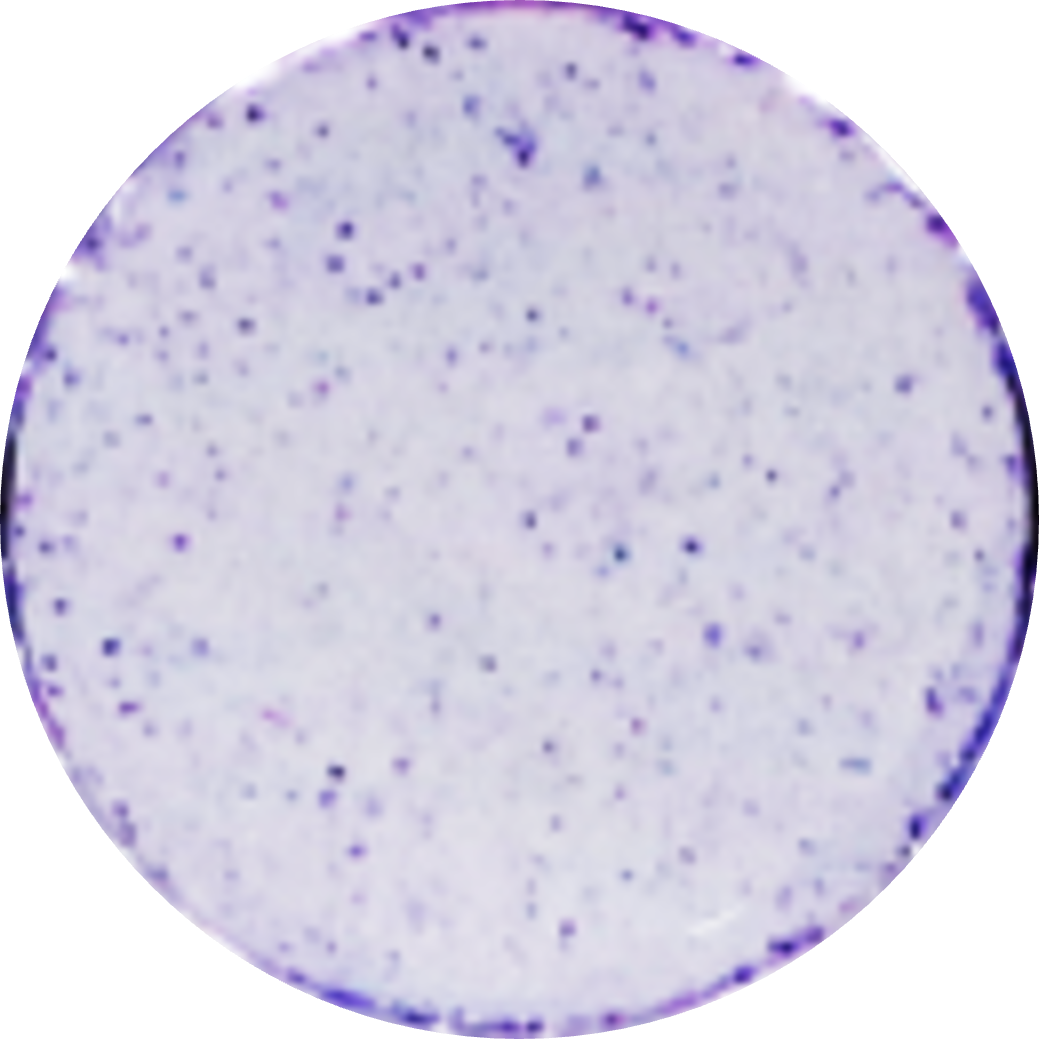

Supplement: Supplementary file 9 — Source data Fig. 7 [file 44319_2024_228_MOESM9_ESM.zip › Figure 7/Figure 7H/shRAI14#1+TRULI.tif]

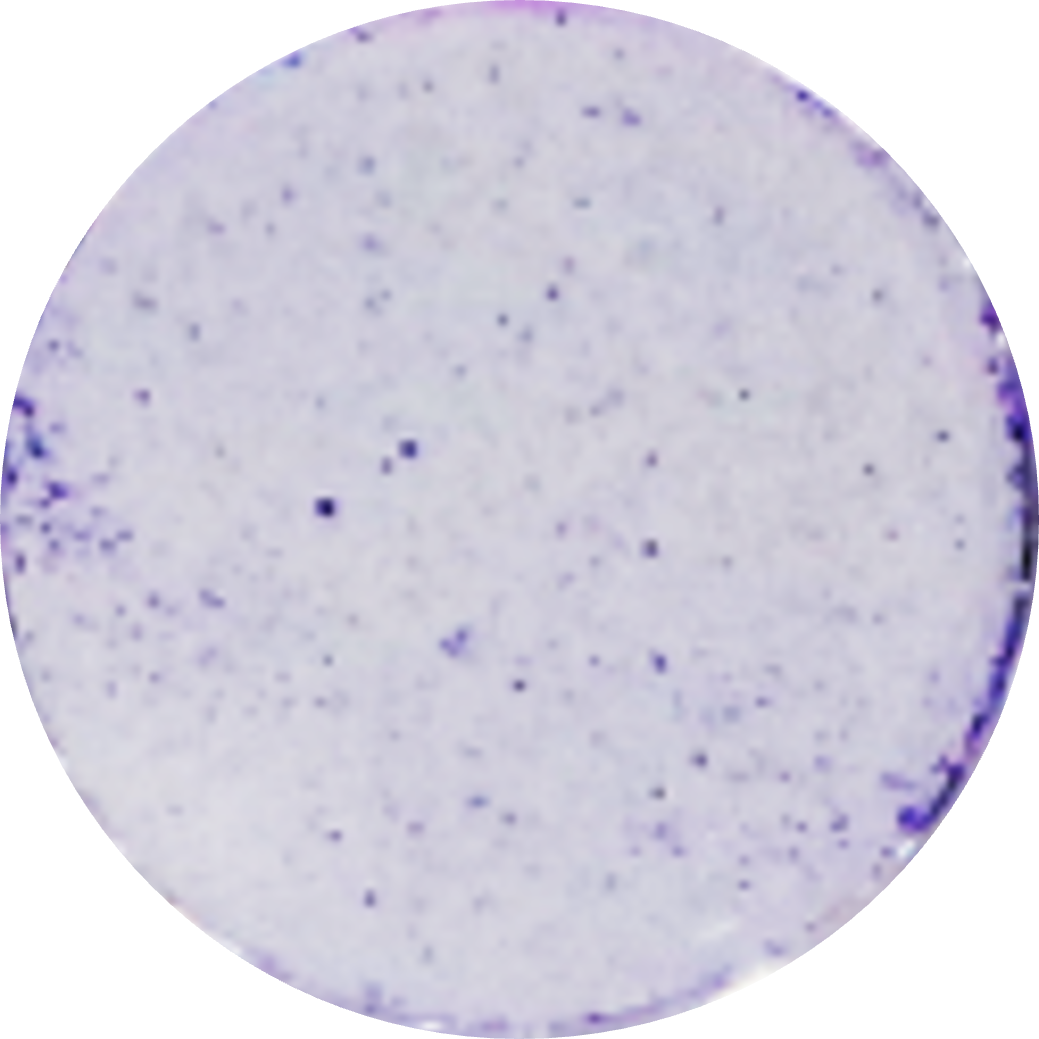

Supplement: Supplementary file 9 — Source data Fig. 7 [file 44319_2024_228_MOESM9_ESM.zip › Figure 7/Figure 7H/shRAI14#2+DMSO.tif]

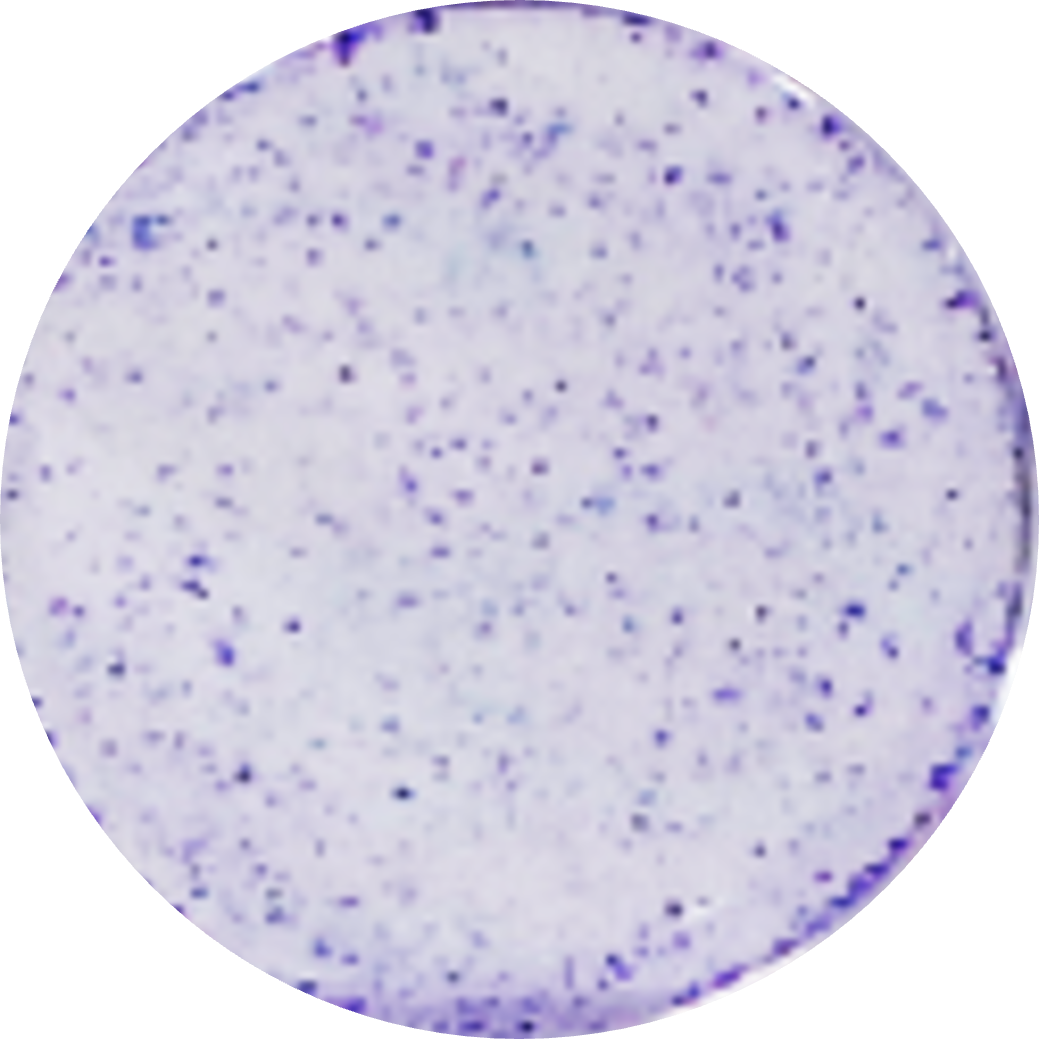

Supplement: Supplementary file 9 — Source data Fig. 7 [file 44319_2024_228_MOESM9_ESM.zip › Figure 7/Figure 7H/shRAI14#2+TRULI.tif]

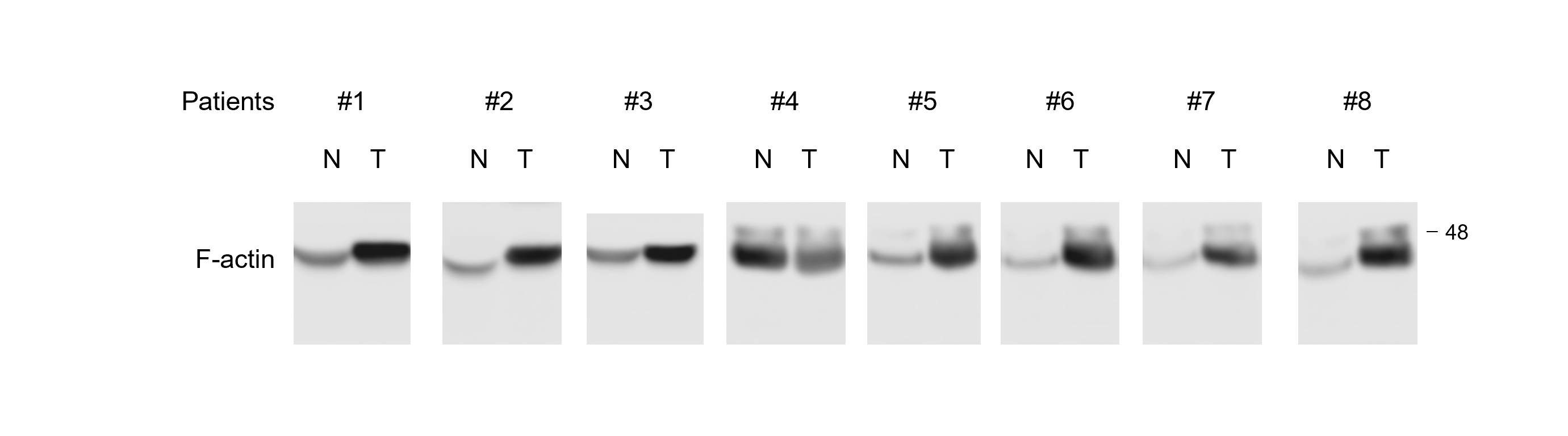

Supplement: Supplementary file 9 — Source data Fig. 7 [file 44319_2024_228_MOESM9_ESM.zip › Figure 7/Figure 7I/F-actin.tif]

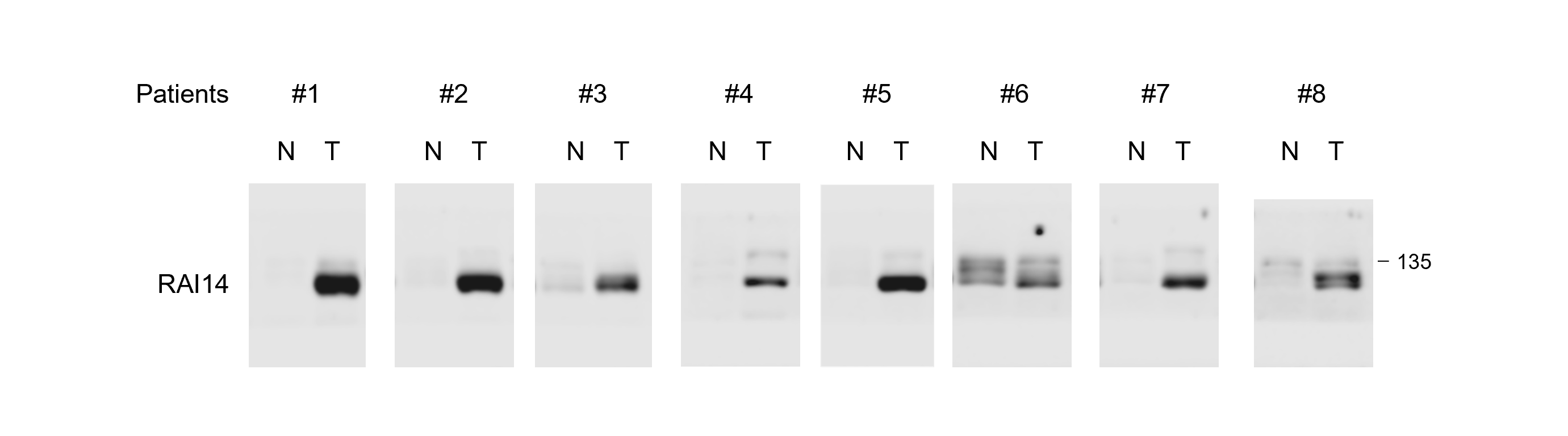

Supplement: Supplementary file 9 — Source data Fig. 7 [file 44319_2024_228_MOESM9_ESM.zip › Figure 7/Figure 7I/RAI14.tif]

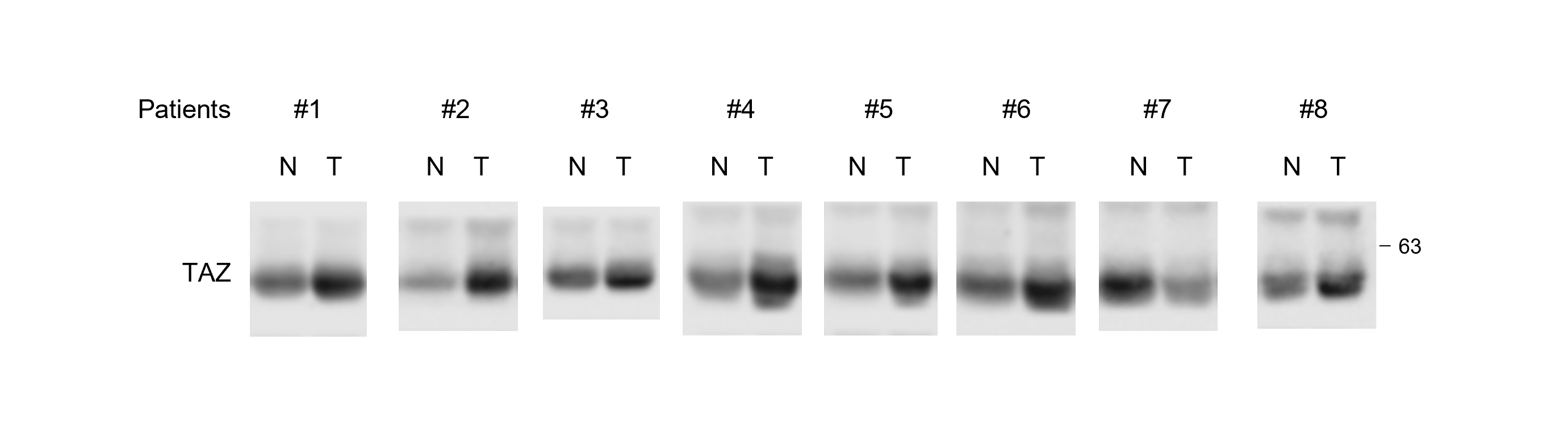

Supplement: Supplementary file 9 — Source data Fig. 7 [file 44319_2024_228_MOESM9_ESM.zip › Figure 7/Figure 7I/TAZ.tif]

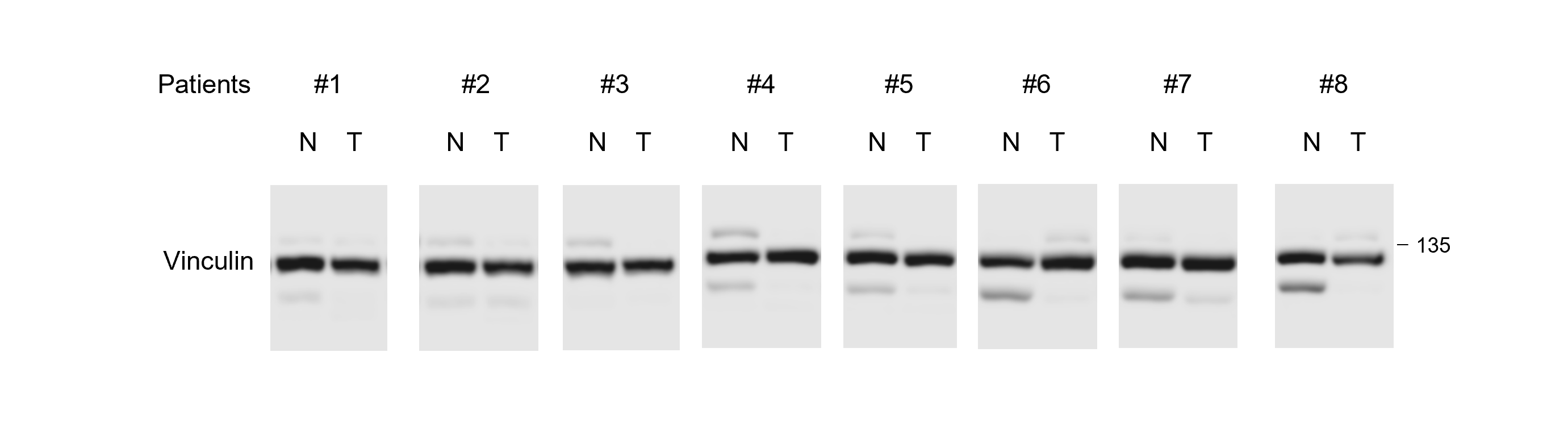

Supplement: Supplementary file 9 — Source data Fig. 7 [file 44319_2024_228_MOESM9_ESM.zip › Figure 7/Figure 7I/Vinculin.tif]

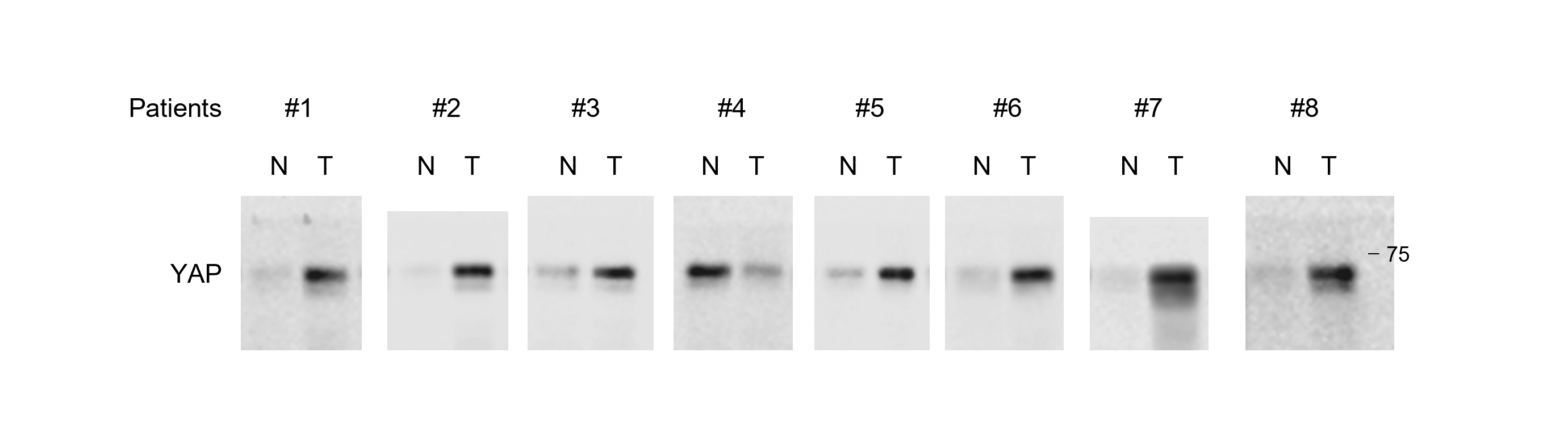

Supplement: Supplementary file 9 — Source data Fig. 7 [file 44319_2024_228_MOESM9_ESM.zip › Figure 7/Figure 7I/YAP.tif]

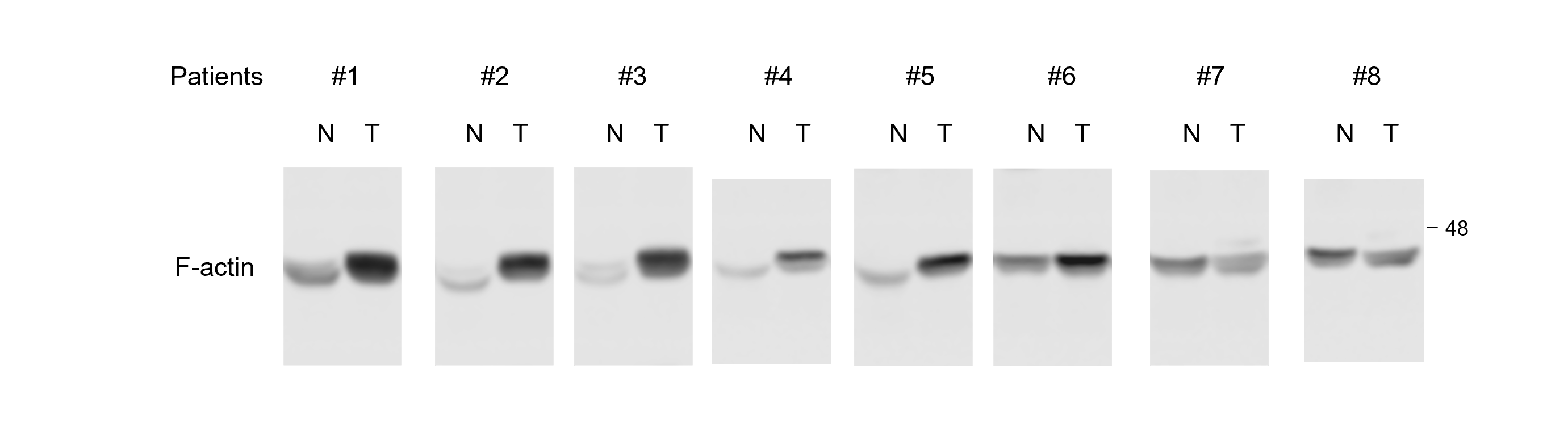

Supplement: Supplementary file 9 — Source data Fig. 7 [file 44319_2024_228_MOESM9_ESM.zip › Figure 7/Figure 7J/F-actin.tif]

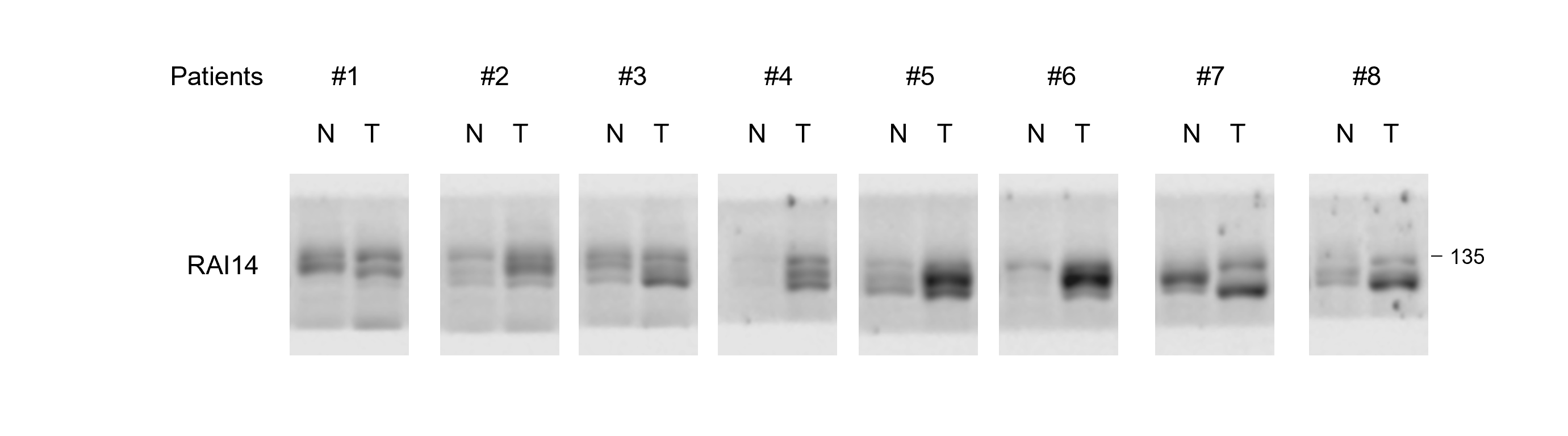

Supplement: Supplementary file 9 — Source data Fig. 7 [file 44319_2024_228_MOESM9_ESM.zip › Figure 7/Figure 7J/RAI14.tif]

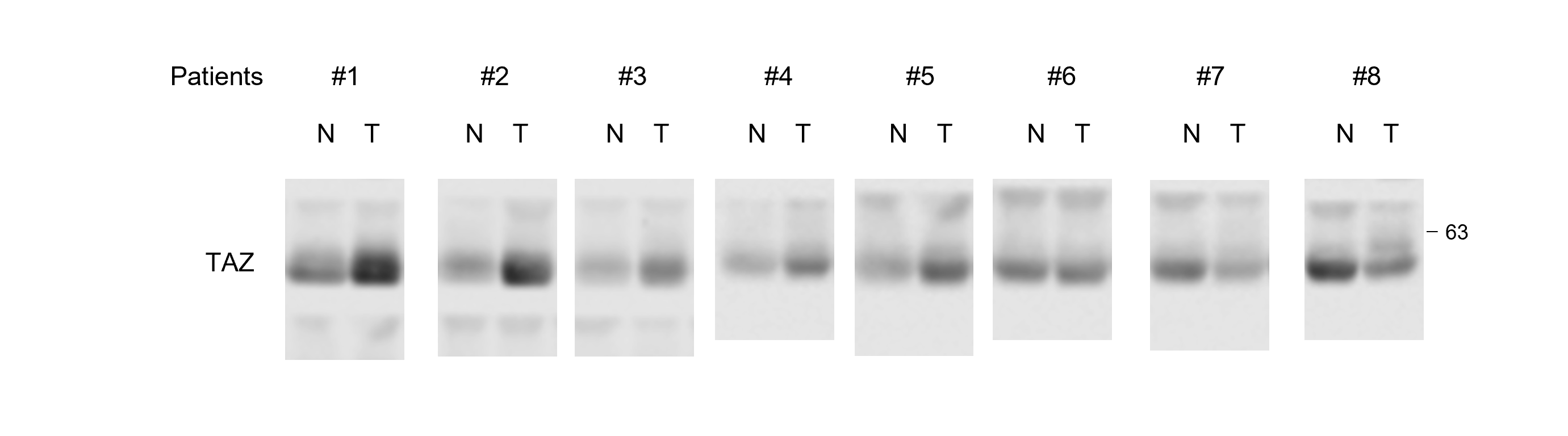

Supplement: Supplementary file 9 — Source data Fig. 7 [file 44319_2024_228_MOESM9_ESM.zip › Figure 7/Figure 7J/TAZ.tif]

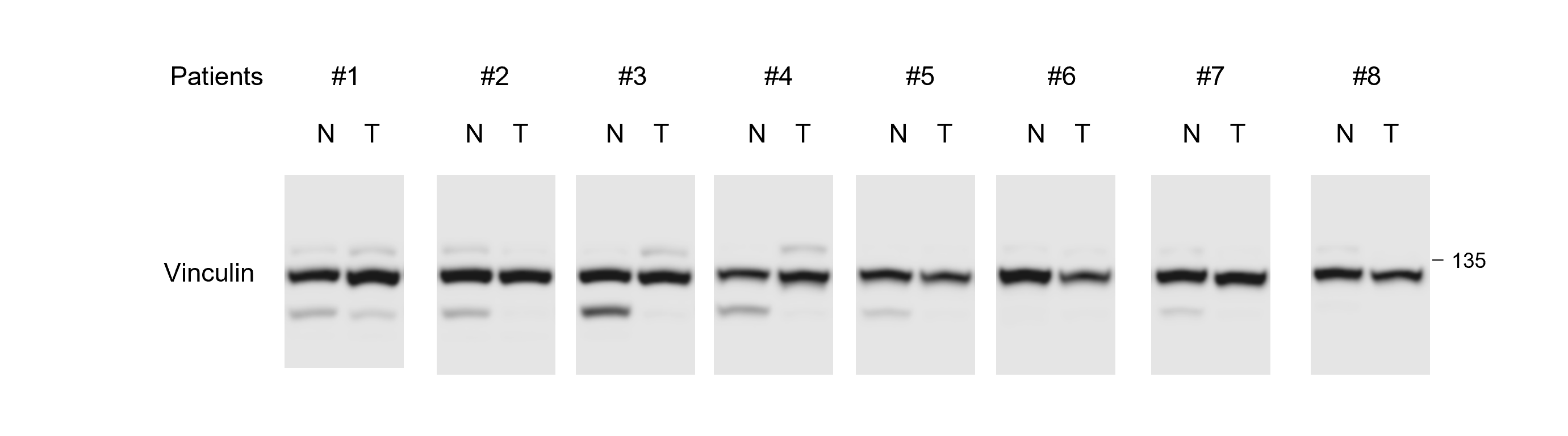

Supplement: Supplementary file 9 — Source data Fig. 7 [file 44319_2024_228_MOESM9_ESM.zip › Figure 7/Figure 7J/Vinculin.tif]

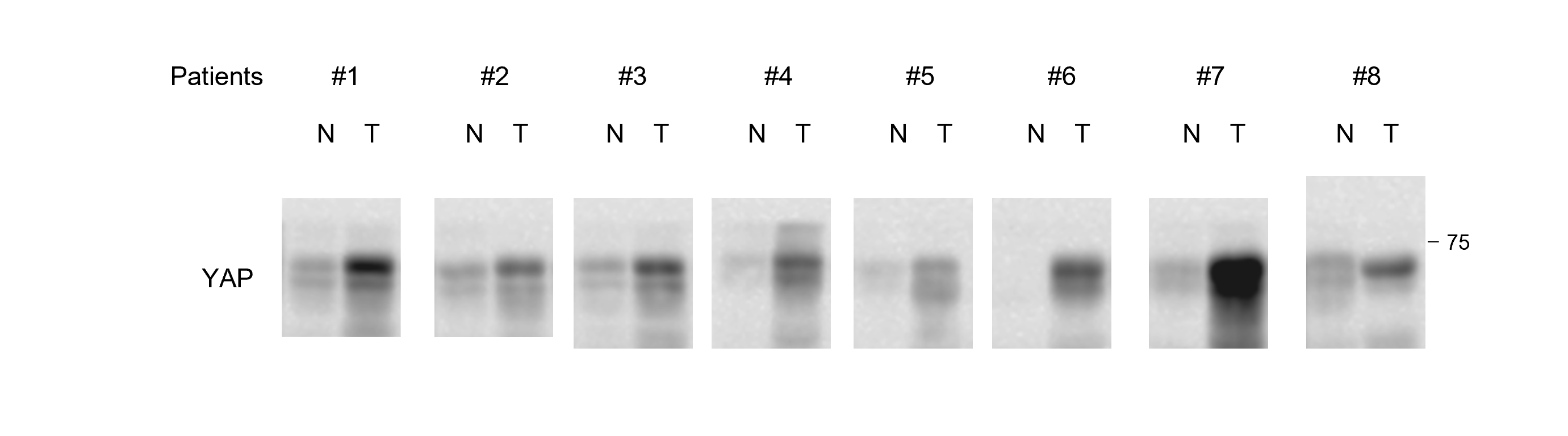

Supplement: Supplementary file 9 — Source data Fig. 7 [file 44319_2024_228_MOESM9_ESM.zip › Figure 7/Figure 7J/YAP.tif]
