# Supplementary material for: Retinoic acid-induced protein 14 links mechanical forces to Hippo signaling
Source: EMBO Rep. 2024 Aug 19;25(9):18. doi: 10.1038/s44319-024-00228-0 (PMC11387738; doi:10.1038/s44319-024-00228-0)
Supplement: Supplementary file 11 — Expanded View Figures [file 44319_2024_228_MOESM11_ESM.pdf]

Expanded View Figures

Figure EV1. Finding novel regulators of Hippo signaling by analyzing the TCGA database.

(**A**) List of the top fifteen differentially expressed genes with poor prognosis at high expression levels in stomach adenocarcinoma (STAD). Genes with a high correlation with YAP target genes (*CTGF* and *CYR61*) are highlighted in light yellow. Genes with increased expression in tumors are highlighted in apricot. Genes corresponding to both of the above conditions are highlighted in yellow. (**B**) Kaplan–Meier plots show that high expression of *RAI14* in gastric cancer patients is associated with poor prognosis. The upper 50th percentile ($n = 189$) and the lower 50th percentile ($n = 189$) were analyzed in gastric cancer patients. HR, hazard ratio. (**C**) The violin plot shows a high expression of RAI14 in gastric tumor samples. The Y-axis indicates the log base 2 of RAI14 expression. The analysis included normal samples from the GTEx database ($n = 172$) and the TCGA database ($n = 36$) as well as tumor samples from the TCGA database ($n = 414$). (**D**) The mRNA expression of gastric cancer patients ($n = 450$) from the TCGA database shows a positive correlation between *RAI14* and YAP-target genes (*CTGF* and *CYR61*). Data information: In (**B**), the hazard ratio (HR) was calculated using the Mantel–Haenszel method. In (**D**), the correlation coefficient (r) was calculated using Pearman's linear correlation. Statistical analysis was performed using log-rank test (**B**) and two-tailed unpaired *t* test (**C**), or calculating right-tailed F probability distribution (**D**), and exact *P* values are shown in each figure; $P < 0.05$, statistically significant. Source data are available online for this figure.

A

Top15 differential Survival Genes in STAD (gastric cancer)

RANK	Gene name	Gene ID	P-Value (Survival os)	Prognosis	Correlation with *CTGF* (Pearson correlation R)	Correlation with *CYR61* (Pearson correlation R)	Increased expression in tumor?
1	*GFAP*	ENSG00000131095.11	2.12×10^{-5}	unfavorable	0.23	0.2	
2	*RP11-497E19.1*	ENSG00000205562.2	2.33×10^{-5}	unfavorable	0.28	0.3	
3	*ASPA*	ENSG00000108381.10	2.69×10^{-5}	unfavorable	0.42	0.36	
4	*SERPINE1*	ENSG00000106366.8	3.41×10^{-5}	unfavorable	0.39	0.43	O
5	*ZNF883*	ENSG00000228623.3	3.59×10^{-5}	unfavorable	-0.026	-0.0087	
6	*AOC4P*	ENSG00000260105.6	3.86×10^{-5}	unfavorable	0.36	0.31	
7	*CBLN4*	ENSG00000054803.3	3.97×10^{-5}	unfavorable	0.11	0.15	
8	*NT5E*	ENSG00000135318.11	4.74×10^{-5}	unfavorable	0.082	0.044	O
9	*AC002480.3*	ENSG00000232759.1	6.37×10^{-5}	unfavorable	0.062	0.11	
10	*MEI4*	ENSG00000269964.2	6.39×10^{-5}	unfavorable	0.065	0.054	
11	*CTD-2054N24.2*	ENSG00000259363.5	6.93×10^{-5}	unfavorable	0.3	0.24	
12	*RP11-1069G10.2*	ENSG00000259727.1	9.09×10^{-5}	unfavorable	0.23	0.18	
13	*EMX2OS*	ENSG00000229847.8	9.24×10^{-5}	unfavorable	0.16	0.16	
14	*ZNF192P1*	ENSG00000226314.7	9.45×10^{-5}	unfavorable	0.023	-0.0096	
15	*RAI14*	ENSG00000039560.13	9.67×10^{-5}	unfavorable	0.49	0.4	O

Genes having a high correlation with *CTGF*, *CYR61* (R > 0.3)
Genes with increased expression in tumors
Genes having a high correlation with *CTGF*, *CYR61* and increased expression in tumors

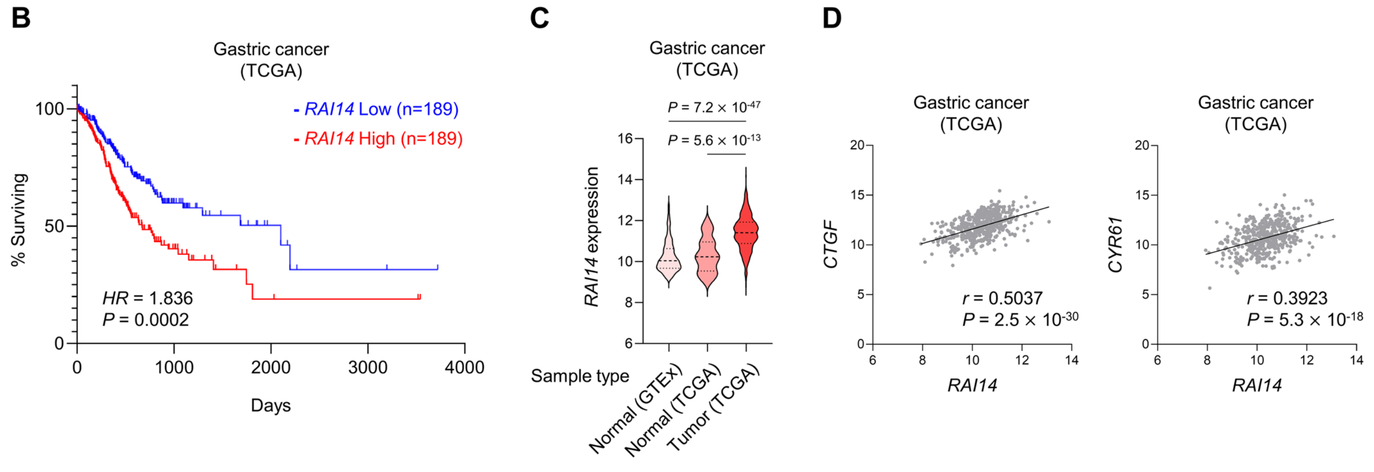

 © The Author(s)

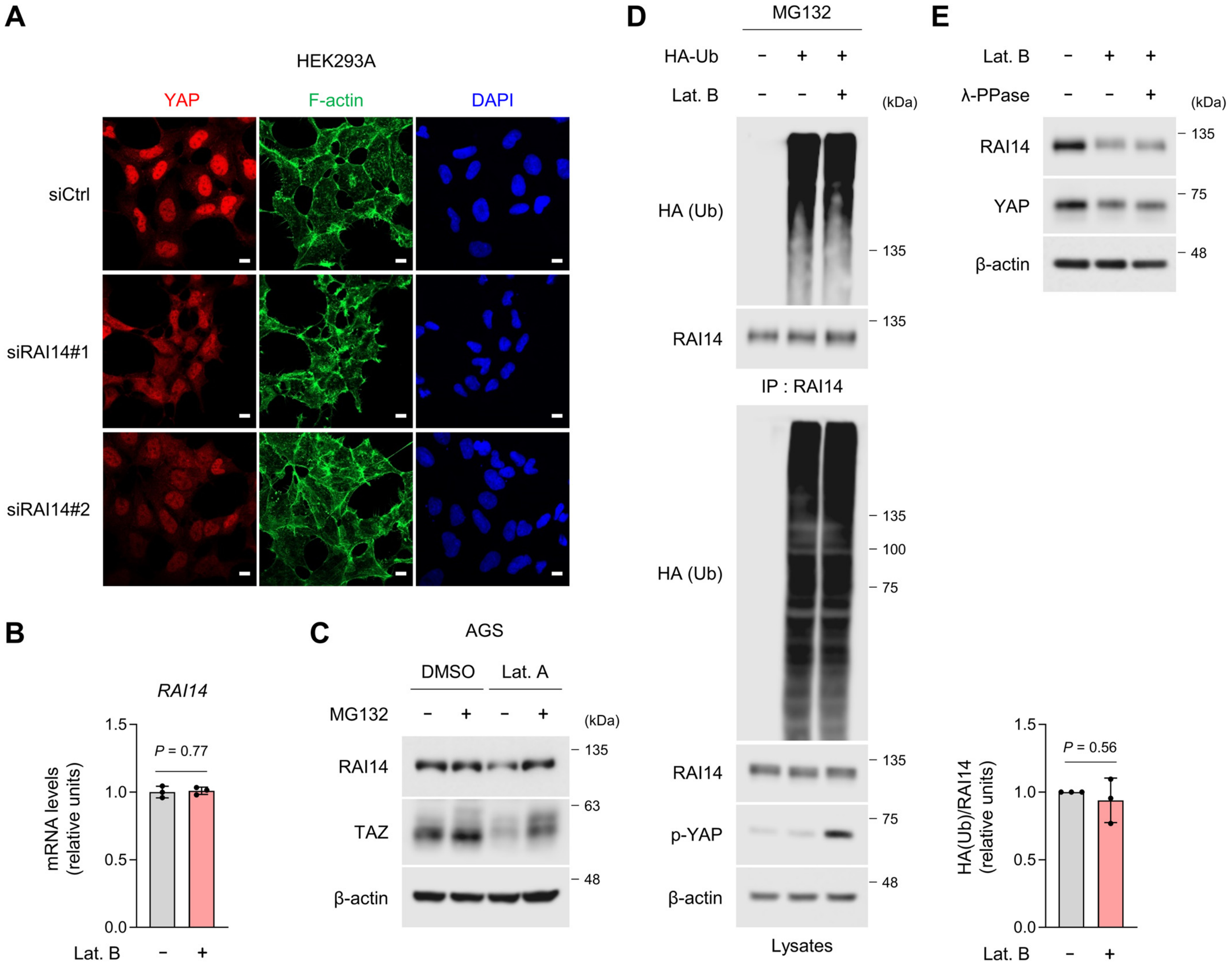

Figure EV2. Destabilization of F-actin induces proteasomal degradation of RAI14.

(**A**) Marginal effect of RAI14 knockdown on F-actin formation. HEK293A cells were used. For F-actin staining, phalloidin was used. Scale bars: 10 μm. (**B**) Destabilization of F-actin by Latrunculin B (Lat. B) does not reduce RAI14 mRNA expression. HEK293A cells were treated with DMSO or Lat. B (2 μM) for 6 h. (**C**) F-actin destabilization-mediated downregulation of RAI14 is dependent on the proteasomal degradation pathway. AGS cells were treated with Lat. A (2 μM) and MG132 (25 μM) for 6 h as indicated in the figure. (**D**) Destabilization of F-actin does not induce polyubiquitination of RAI14. HEK293A cells were transfected and treated with Lat. B (2 μM) and MG132 (25 μM) for 6 h as indicated in the figure. The HA(Ub)/RAI14 ratio from three immunoblot bands was quantified. (**E**) The mobility shift of RAI14 by F-actin destabilization is due to its phosphorylation. HEK293A cells were treated with Lat. B for 6 h and lambda phosphatase (λ-PPase) according to the manufacturer's instructions. Data information: In (**B**, **D**), the error bars indicate ± s.d. of triplicate measurements (**B** is technical replicates, and **D** is biological replicates). Statistical analysis was performed using two-tailed unpaired *t* test, and exact *P* values are shown in each figure; $P < 0.05$, statistically significant. Black dots on the graphs indicate individual measurements. Source data are available online for this figure.

© The Author(s)

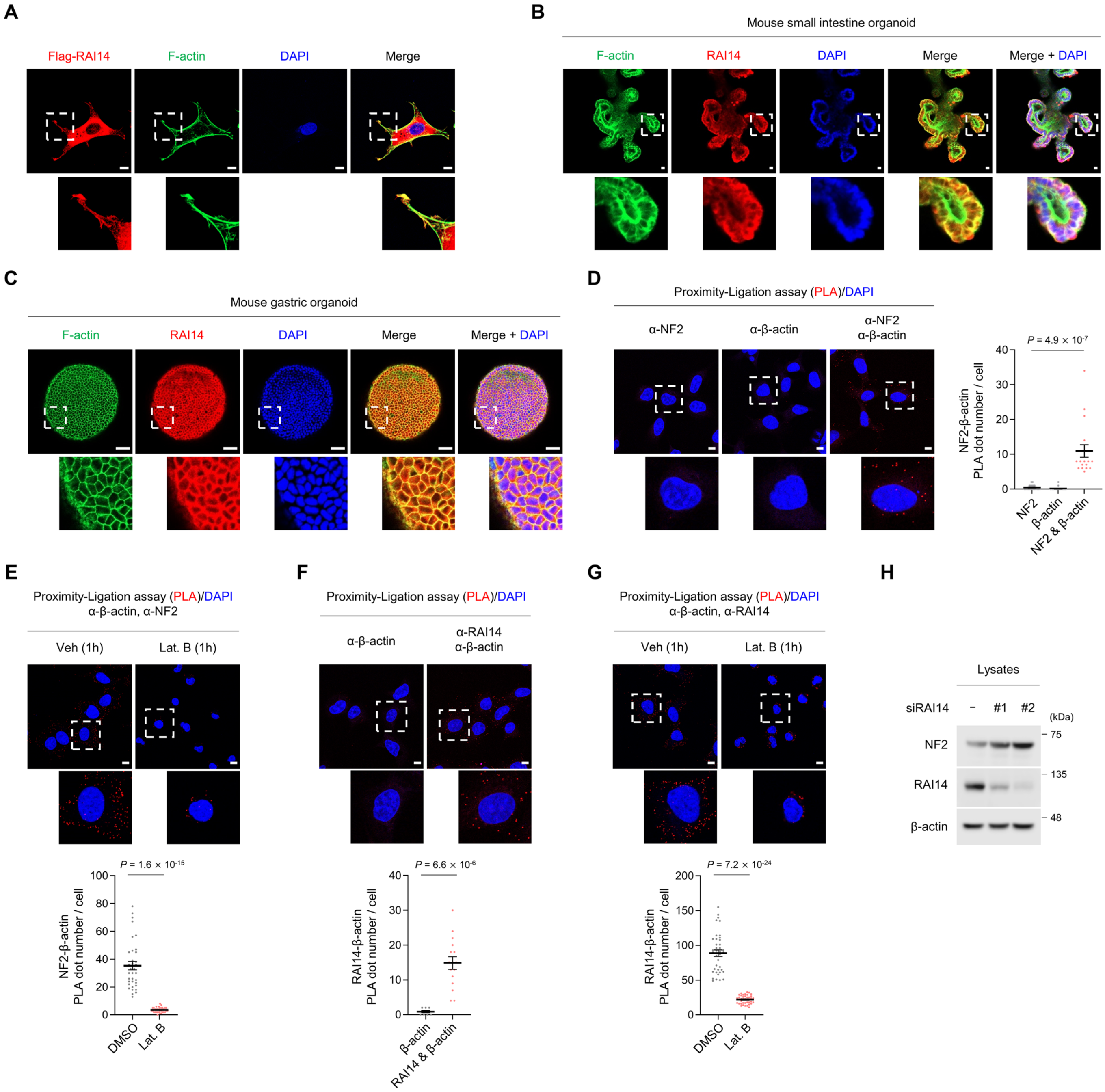

Figure EV3. RAI14 and NF2 interaction occurs on F-actin.

(**A**) Overexpressed Flag-RAI14 colocalizes with F-actin. HEK293A cells were used. Scale bars: 10 μm. (**B**, **C**) RAI14 colocalizes with F-actin at the endogenous level in organoid models. Mouse small intestine organoids (**B**, scale bars: 10 μm) or gastric organoids (**C**, scale bars: 50 μm) were used. (**D–G**) Endogenous NF2 and β-actin (**D**, **E**), and RAI14 and β-actin (**F**, **G**) are in proximity. HEK293A cells were treated with Lat. B (2 μM) for 1 h. The quantification of the number of dots in a single cell is shown. In (**D**), 20, 21, and 18 cells were used for NF2, β-actin, and NF2&β-actin, respectively. In (**E**), 35 and 33 cells were used for DMSO and Lat. B, respectively. In (**F**), 9 and 16 cells were used for β-actin and RAI14&β-actin, respectively. In (**G**), 40 cells were used for DMSO and Lat. B, respectively. Scale bars: 10 μm. (**H**) Immunoblot of lysates used for Fig. 5E. Data information: In (**D–G**), the error bars indicate ± s.e.m. of the measurements (technical replicates). Statistical analysis was performed using two-tailed unpaired t-test, and exact *P* values are shown in each figure; $P < 0.05$, statistically significant. Colored dots on the graphs indicate individual measurements. Source data are available online for this figure.

 © The Author(s)

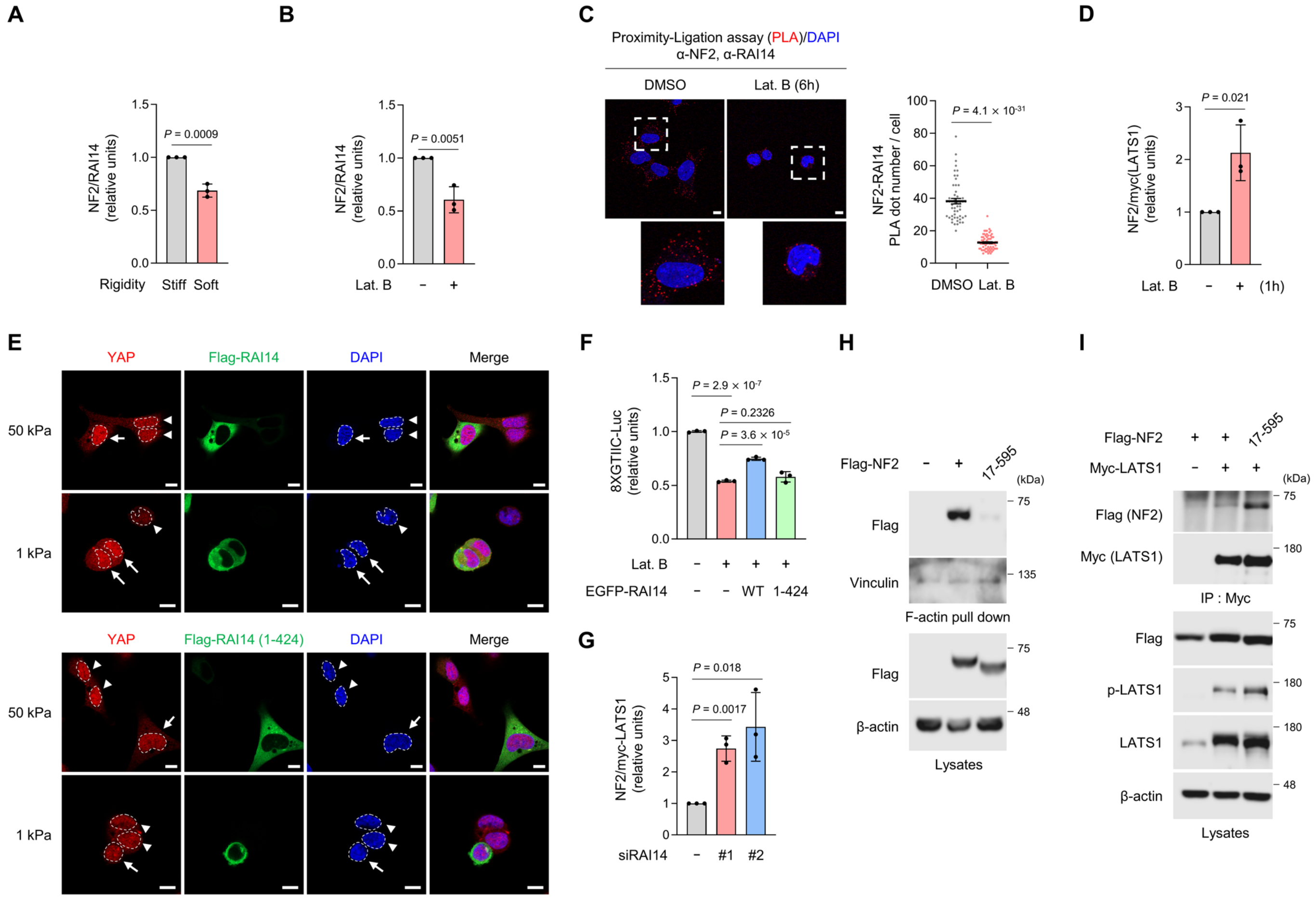

Figure EV4. ECM stiffness and F-actin integrity regulate the interaction between RAI14, NF2 and LATS.

(**A**, **B**) Quantification of immunoprecipitated NF2/RAI14 ratio from three immunoblot bands in Fig. 6B,C, respectively. (**C**) PLA assays show that the interaction between NF2 and RAI14 is inhibited by Lat. B treatment. HEK293A cells were treated with Lat. B (2 μM) and MG132 (25 μM) for 6 h. The quantification of the number of dots in a single cell is shown in the right panel. 52 and 70 cells were used for DMSO and Lat. B, respectively. Scale bars: 10 μm. (**D**) Quantification of immunoprecipitated NF2/Myc(LATS1) ratio from three immunoblot bands in Fig. 6H. (**E**) Overexpression of RAI14, but not the RAI14(1–424) mutant form, promotes nuclear localization of YAP under both hard and soft matrix conditions. HEK293A cells were transfected and incubated on a stiff (50 kPa) or soft (1 kPa) matrix for one day. Nuclei of Flag-RAI14 (upper panel) or Flag-RAI14(1–424) (lower panel) overexpressing cells are marked with a white arrow. Nuclei of un-transfected cells (upper and lower panel) are marked with a white arrowhead. Scale bars: 10 μm. (**F**) Overexpression of RAI14 partially rescues the YAP reporter activity but not in RAI14 (1–424) form. HEK293A cells were transfected and treated with Lat. B (2 μM) for 6 h. (**G**) Quantification of immunoprecipitated NF2/Myc(LATS1) ratio from three immunoblot bands in Fig. 6K. (**H**) The NF2 (17–595) form fails to bind F-actin. HEK293A cells were used. (**I**) The NF2 (17–595) form shows increased interaction with LATS1 and phosphorylation of LATS1 compared to wild-type NF2. HEK293A cells were used. Data information: In (**A**, **B**, **D**, **F**, **G**), the error bars indicate ± s.d. of triplicate measurements (biological replicates). In (**C**), the error bars indicate ± s.e.m. of the measurements (technical replicates). Statistical analysis was performed using two-tailed unpaired *t* test, and exact *P* values are shown in each figure; $P < 0.05$, statistically significant. Black or colored dots on the graphs indicate individual measurements. Source data are available online for this figure.

© The Author(s)

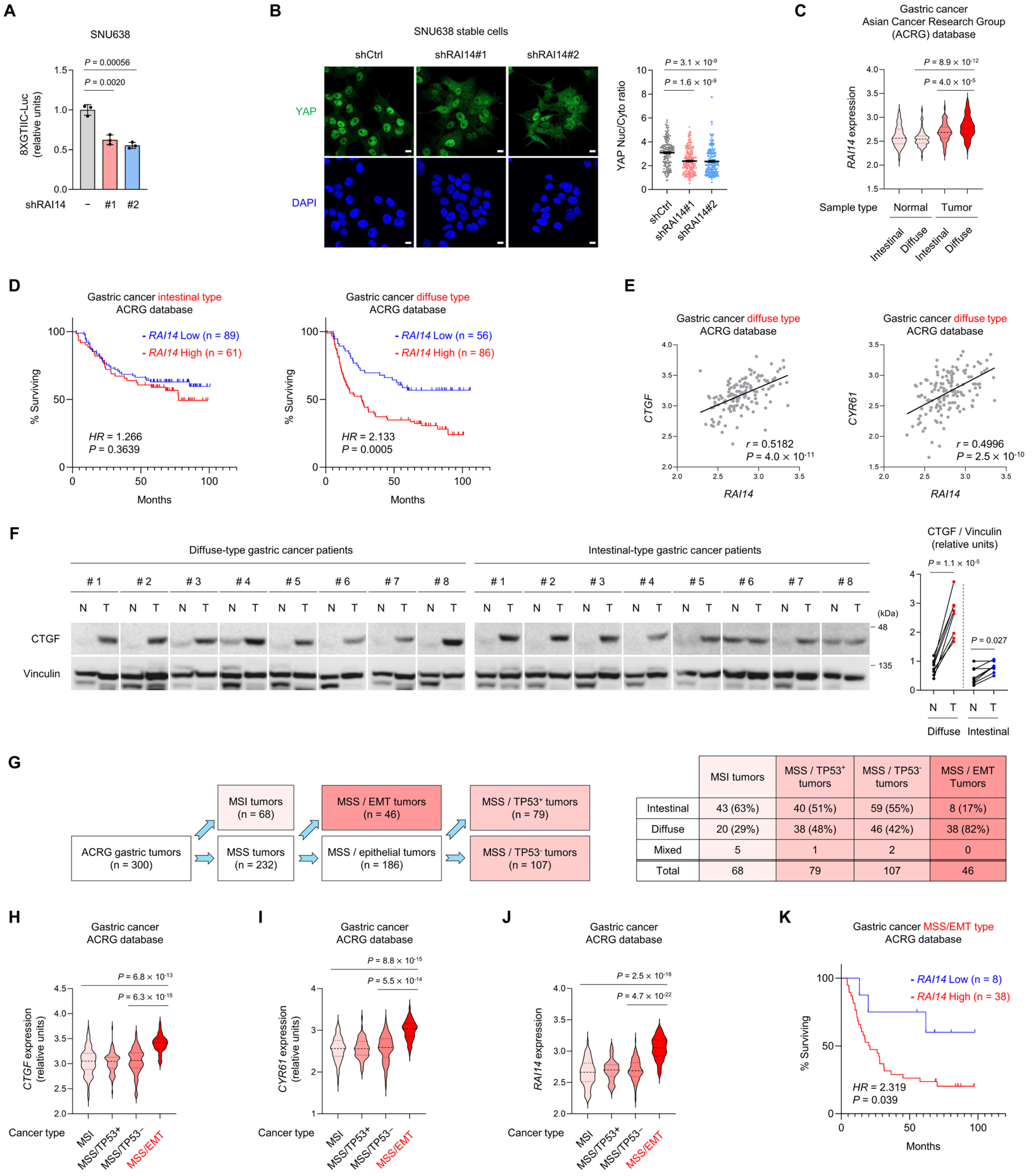

	MSI tumors	MSS / TP53+ tumors	MSS / TP53- tumors	MSS / EMT Tumors
Intestinal	43 (63%)	40 (51%)	59 (55%)	8 (17%)
Diffuse	20 (29%)	38 (48%)	46 (42%)	38 (82%)
Mixed	5	1	2	0
Total	68	79	107	46

 © The Author(s)

Figure EV5. RAI14-Hippo signaling promotes cell proliferation and growth, and is implicated in gastric cancer progression.

(**A**) Downregulation of RAI14 by shRNA reduces YAP-reporter activity. SNU638 cells were used. (**B**) Knockdown of RAI14 reduces the nuclear localization of YAP. SNU638 cells were used. Scale bars: 10 μm. Quantification of the nuclear/cytoplasmic ratio in a single cell is shown in the right panel. 170, 187, and 203 cells were used for shCtrl, shRAI14#1, and shRAI14#2, respectively. (**C**) The violin plot shows a high expression of *RAI14* in gastric tumor samples, especially in the diffuse-type. The y-axis indicates the log base 10 of *RAI14* expression. Adjacent normal tissue samples from intestinal-type ($n = 39$) and diffuse-type ($n = 58$) patients and tumor tissue samples from intestinal-type ($n = 150$) and diffuse-type ($n = 142$) patients were analyzed. (**D**) Kaplan–Meier plots show that high expression of *RAI14* in diffuse-type GC patients is associated with poor prognosis. The upper 41st ($n = 61$) and lower 59th ($n = 89$) percentiles were analyzed for intestinal-type GC patients, and the upper 61st ($n = 86$) and lower 39th ($n = 56$) percentiles were analyzed for diffuse-type GC patients. HR, hazard ratio. (**E**) The mRNA expression of diffuse-type GC ($n = 142$) from the ACRG database shows a positive correlation between *RAI14* and YAP-target genes (*CTGF* and *CYR61*). (**F**) CTGF level were high in GC tissues and significantly higher in DGC. Quantification of CTGF/Vinculin ratios from immunoblots was performed as in Fig. 7K. (**G**) Schematic representation of the molecular classification of GC from the ACRG database (left panel). Patients with MSS/EMT-type mainly belong to the diffuse-type (right panel). (**H–J**) The violin plot shows high expression of *CTGF*, *CYR61* and *RAI14* in MSS/EMT-type gastric tumor samples. The y-axis indicates the log base 10 of *RAI14* expression. Tissue samples from MSI ($n = 68$), MSS/TP53^{+} ($n = 79$), MSS/TP53^{-} ($n = 107$) and MSS/EMT-type patients ($n = 46$) were analyzed. (**K**) Kaplan–Meier plots show that high expression of *RAI14* in MSS/EMT-type GC patients is associated with a worse prognosis. The upper 17th percentile ($n = 8$) and the lower 83rd percentile ($n = 38$) were analyzed in patients with intestinal-type gastric cancer. HR, hazard ratio. Data information: In (**A**), the error bars indicate ± s.d. of triplicate measurements (biological replicates). In (**B**), the error bars indicate ± s.e.m. of the measurements (technical replicates). In (**D**, **K**), the hazard ratio (HR) was calculated using the Mantel–Haenszel method. In (**E**), the correlation coefficient (r) was calculated using Pearman's linear correlation. Statistical analysis was performed using two-tailed unpaired *t* test (**A–C**, **F**, **H–J**) and log-rank test (**D**, **K**), or calculating right-tailed F probability distribution (**E**), and exact *P* values are shown in each figure; $P < 0.05$, statistically significant. Black or colored dots on the graphs indicate individual measurements. Source data are available online for this figure.
